# An individual participant data meta-analysis of how physical activity relates to affective well-being in daily life

**DOI:** 10.1038/s41562-026-02427-2

**Published:** 2026-05-06

**Authors:** Johanna Rehder, Irina Timm, Gesa Berretz, Iris Reinhard, Andreas B. Neubauer, Onur Güntürkün, Keisuke Takano, Walter Bierbauer, Miriam Cabrita, Matthew Bourke, Joshua Smyth, Jinhyuk Kim, Johannes Michalak, Joshua Curtiss, Björn Pannicke, Jacob B. Gallagher, Ana M. Abrantes, Toru Nakamura, Yoshiharu Yamamoto, Paul Cook, Lena M. Wieland, Birte von Haaren-Mack, Bryan McCormick, Justin Hachenberger, Tomas Vetrovsky, Benajmin Henwood, Louise Poppe, Gorden Sudeck, Laura Hollands, Andrea B. Goldschmidt, Lynn Martire, Martina Kanning, Jaclyn P. Maher, Yu-Mei Li, Ulrich Reininghaus, Corina Berli, Caroline Seiferth, Derek J. Hevel, Kate Leger, Amanda E. Staiano, Almut Zeeck, Stefano Calza, Yue Liao, Geralyn R. Ruissen, CoCA Consortium, Andreas Reif, Oliver Grimm, Christine Freitag, Jutta Mayer, Toni Ramos-Quiroga, Christian Fadeuilhe, Jonna Kuntsi, Philip Asherson, Adam Pawley, Jan Buitelaar, Elena Suess, Ulrich Ebner-Priemer, Andreas R. Schwerdtfeger, Matthias Haucke, Loree T. Pham, Siwei Liu, Mark C. Thomas, Andreas Meyer-Lindenberg, Genevieve F. Dunton, Steriani Elavsky, Ulrich W. Ebner-Priemer, Marco Giurgiu, Julian Packheiser, Markus Reichert

**Affiliations:** 1 Department of Sport and Exercise Science, Faculty of Natural and Life Sciences, University of Salzburg, Salzburg, Austria; 2 Junior Researcher Group eHealth and Sports Analytics, Faculty of Sport Science, Ruhr University Bochum, Bochum, Germany; 3 Department of Psychiatry and Psychotherapy, Central Institute of Mental Health, Medical Faculty Mannheim, Heidelberg University, Mannheim, Germany; 4 Institute of Sports and Sports Science, Karlsruhe Institute of Technology, Karlsruhe, Germany; 5 Donders Institute for Brain, Cognition and Behaviour, Radboud Universiteit, Nijmegen, The Netherlands; 6 Core Facility Biostatistics, Central Institute of Mental Health, Medical Faculty Mannheim, Heidelberg University, Mannheim, Germany; 7 Institute of Psychology, RWTH Aachen University, Aachen, Germany; 8 Department of Biopsychology, Faculty of Psychology, Institute of Cognitive Neuroscience, Ruhr University Bochum, Bochum, Germany; 9 Research Center One Health Ruhr of the University Alliance Ruhr, Ruhr University Bochum, Bochum, Germany; 10 Department of Psychology, Ludwig-Maximilians-Universität München, Munich, Germany; 11 Applied Social and Health Psychology, University of Zurich, Zurich, Switzerland; 12 SHINE 2Europe, Coimbra, Portugal; 13 School of Human Movement and Nutrition Sciences, Faculty of Health and Behavioural Sciences, University of Queensland, Brisbane, Queensland Australia; 14 Department of Psychology, The Ohio State University, Columbus, OH USA; 15 Department of Informatics, Graduate School of Integrated Science and Technology, Shizuoka University, Shizuoka, Japan; 16 Department of Psychology and Psychotherapy, Witten/Herdecke University, Witten, Germany; 17 Department of Psychiatry, Depression Clinical and Research Program, Massachusetts General Hospital, Boston, MA USA; 18 Department of Psychology, Centre for Cognitive Neuroscience, Faculty of Natural and Life Sciences, University of Salzburg, Salzburg, Austria; 19 Department of Health and Human Physiology, The University of Iowa, Iowa City, IA USA; 20 Department of Kinesiology, Iowa State University, Ames, IA USA; 21 Department of Psychiatry and Human Behavior, Alpert Medical School of Brown University, Providence, RI USA; 22 Butler Hospital, Providence, RI USA; 23 Institute for Datability Science, Osaka University, Suita, Japan; 24 Graduate School of Education, The University of Tokyo, Bunkyo, Japan; 25 College of Nursing, University of Colorado Anschutz Medical Campus, Aurora, CO USA; 26 Leibniz Institute for Research and Information in Education, Frankfurt am Main, Germany; 27 Department of Health and Rehabilitation Sciences, Temple University, Philadelphia, PA USA; 28 Department of Psychology, Bielefeld University, Bielefeld, Germany; 29 Faculty of Physical Education and Sport, Charles University, Prague, Czech Republic; 30 USC Suzanne Dworak-Peck School of Social Work, University of Southern California, Los Angeles, CA USA; 31 Department of Public Health and Primary Care, Ghent University, Ghent, Belgium; 32 Institute of Sport Science, University of Tübingen, Tübingen, Germany; 33 College of Medicine and Health, St Luke’s Campus, University of Exeter, Exeter, UK; 34 Department of Psychiatry, University of Pittsburgh School of Medicine, Pittsburgh, PA USA; 35 Department of Human Development and Family Studies, The Pennsylvania State University, University Park, PA USA; 36 Department of Sports Science, University of Konstanz, Konstanz, Germany; 37 Department of Kinesiology, University of North Carolina Greensboro, Greensboro, NC USA; 38 Medical School OWL, Bielefeld University, Bielefeld, Germany; 39 Department of Public Mental Health, Central Institute of Mental Health, Medical Faculty Mannheim, Heidelberg University, Mannheim, Germany; 40 German Center for Mental Health (DZPG), partner site Mannheim-Heidelberg-Ulm, Mannheim, Germany; 41 Health Service and Population Research Department, Institute of Psychiatry, Psychology and Neuroscience, King’s College London, London, UK; 42 ESRC Centre for Society and Mental Health, King’s College London, London, UK; 43 Institute of Psychology, University of Bern, Bern, Switzerland; 44 Department of Clinical Psychology and Psychotherapy, University of Bamberg, Bamberg, Germany; 45 Department of Clinical Psychology and Psychotherapy, Freie Universität Berlin, Berlin, Germany; 46 Department of Psychology, The University of Kentucky, Lexington, KY USA; 47 Pennington Biomedical Research Center, Louisiana State University, Baton Rouge, LA USA; 48 Department of Psychosomatic Medicine and Psychotherapy, Center for Mental Health, Faculty of Medicine, University of Freiburg, Freiburg, Germany; 49 Unit of Biostatistics and Bioinformatics, Department of Molecular and Translational Medicine, University of Brescia, Brescia, Italy; 50 Department of Kinesiology, University of Texas at Arlington, Arlington, TX USA; 51 Faculty of Kinesiology, Sport, and Recreation, University of Alberta, Edmonton, Alberta Canada; 52 Department of Psychology, University of Graz, Graz, Austria; 53 Department of Psychiatry and Psychotherapy, Charité Universitätsmedizin Berlin, Charité Campus Mitte, Corporate member of Freie Universität Berlin, Humboldt Universität zu Berlin, Berlin Institute of Health, Berlin, Germany; 54 Chan Division of Occupational Science and Occupational Therapy, University of Southern California, Los Angeles, CA USA; 55 Department of Human Ecology, University of California at Davis, Davis, CA USA; 56 VISN 4 Mental Illness Research, Education and Clinical Center, VA Pittsburgh Healthcare System, Pittsburgh, PA USA; 57 Department of Population and Public Health Sciences, Keck School of Medicine, University of Southern California, Los Angeles, CA USA; 58 Department of Human Movement Studies, University of Ostrava, Ostrava, Czech Republic; 59 Institute of Movement Therapy and Movement-oriented Prevention and Rehabilitation, German Sport University Cologne, Cologne, Germany; 60 Department of Social Neuroscience, Faculty of Medicine, Ruhr University Bochum, Bochum, Germany; 61 Department of Psychiatry, Psychosomatic Medicine and Psychotherapy, University Hospital Frankfurt, Goethe University Frankfurt, Frankfurt am Main, Germany; 62 Department of Child and Adolescent Psychiatry, Psychosomatic Medicine and Psychotherapy, University Hospital Frankfurt, Goethe University Frankfurt, Frankfurt am Main, Germany; 63 Department of Psychiatry, Hospital Universitari Vall d’Hebron, Barcelona, Spain; 64 Group of Psychiatry, Mental Health and Addictions, Vall d’Hebron Research Institute, Barcelona, Spain; 65 Biomedical Network Research Centre on Mental Health, Barcelona, Spain; 66 Department of Psychiatry and Forensic Medicine, Universitat Autònoma de Barcelona, Barcelona, Spain; 67 Institute of Psychiatry, Psychology and Neuroscience, King’s College London, London, UK; 68 Radboud University Medical Center, Nijmegen, the Netherlands; 69 Department of Neuropediatrics and Muscle Disorders, Medical Center and Faculty of Medicine, University of Freiburg, Freiburg, Germany; 70 Institute of Sports and Sport Sciences, Karlsruhe Institute of Technology, Karlsruhe, Germany

**Keywords:** Human behaviour, Quality of life

## Abstract

Physical inactivity constitutes a pressing societal problem. To realize physical activity’s (PA) potential as a key health resource, mechanisms of PA engagement need to be understood. Laboratory and interventional studies documented that exercise relates to affective well-being (AWB) and suggested that AWB may shape PA behaviour. Digitalization enabled the investigation of how PA relates to AWB in everyday life, but findings from individual studies are ambiguous. Here we compiled 67 datasets (55.2% of eligible records) including 321,345 smartphone-based AWB ratings and nearly 1,000,000 h of accelerometer-measured PA (*N* = 8,223 participants) until December 2023 to clarify the nature and extent of PA–AWB associations. One- and two-stage individual participant data meta-analyses reveal that momentary AWB is associated with both prior (within, *r* = 0.05, 99.2% confidence intervals (CI) 0.03 to 0.06; between, *r* = 0.08, 99.2% CI 0.04 to 0.12) and subsequent (within, *r* = 0.04, 99.2% CI 0.03 to 0.05; between, *r* = 0.08, 99.2% CI 0.04 to 0.13) short-term PA in everyday life. Within persons, PA displays a positive association with energetic arousal, positive affective states and valence, yet a negative relation to calmness. The practical effect sizes are comparable to other daily life activities, with energetic arousal evincing the strongest relation to PA. Considerable heterogeneity in associations across individuals can be partially explained by sociodemographic moderators. Between participants, PA relates to positive affective states. The results document the critical relevance of PA–AWB relations in everyday life. They can contribute to the revision and development of health behaviour models and establish a starting point to approach behavioural, physiological and neuronal mechanisms underlying PA–AWB associations.

## Abstract (web summary)

In this individual participant data meta-analysis, and across 321,345 smartphone-ratings of affective well-being and nearly 1 million hours of physical activity measurement, Rehder et al. clarify the nature and extent of activity–well-being relations and document their relevance in humans’ everyday life.

## Main

Worldwide, many humans are insufficiently physically active^1–3^. If people meet current physical activity (PA) recommendations by the World Health Organization^4^, global gross domestic product is estimated to increase by US$314–446 billion per year^5^, mostly attributable to PA counteracting physical^6,7^ and mental illness^8–10^. Recent health behaviour theories consider affective well-being (AWB) as one of the most potent psychological facilitators between PA and health outcomes and suggest it to be critically involved in motivating, maintaining and reinforcing PA^11–17^. In general, AWB is increasingly recognized as a key driver of human behaviour and experience; for example, AWB is shown to be involved not only in PA engagement but also in attention, learning, memory and decision-making processes^18^.

Since the 1980s, cross-sectional studies employing retrospective questionnaires and laboratory experiments provided insights into associations of PA with AWB^19–21^. These findings, however, are constrained by low ecological validity owing to artificial settings, recall biases and a focus on differences between individuals^22–24^. Critically, between-person differences cannot be employed as a proxy for intra-individual processes^25^, a well-known limitation referred to as the ecological fallacy^26–31^. Yet, to promote PA in daily life, it is of crucial importance to understand the within-person associations of PA and AWB in everyday life. In general, variations in AWB in everyday life plausibly affect how humans act and think and are currently suggested to drive key health behaviours such as PA and diet^32^. Insights into these daily life processes can personalize and tailor prevention and treatment by identifying affective facilitators and barriers therewith contributing to public health strategies^32,33^.

To this end, for the last two decades, researchers have collected intensive longitudinal data on the relationship of AWB and PA in daily life^34^. In practice, such research employs wearables including accelerometers for PA tracking and repeated ratings of AWB on smartphone-based electronic diaries as participants go about their daily routines^33,35^. This procedure bypasses aforementioned limitations by assessing momentary instead of retrospective experience complemented by device-based measures and by collecting extensive within-person data for each individual^22,33,36,37^. Of note, it is not only experimental control in laboratory and interventional studies that contrasts with ecological validity of real-life studies but also the type of PA being researched, that is, structured exercise sessions in laboratory and interventional studies versus incidental PA in daily life studies, which comprises exercise but also low-threshold non-exercise activity as PA facets.

Evidence from daily life studies regarding the existence, strength, directionality and circumstances of PA–AWB associations is ambiguous. Liao et al. were the first trying to resolve these ambiguities by conducting a short review in 2015^38^. Differentiating three concepts of AWB (positive affect, negative affect and feeling states, that is, energy and fatigue), they found the strongest evidence for the bidirectional association of PA and positive affect, very weak evidence for PA and negative affect, and insufficient evidence for PA and feeling states. Similarly, in a systematic review on AWB–PA associations in children and adolescents from 2021, associations of PA and subsequent positive affect and energy emerged as most robust. There was some evidence for associations of valence and subsequent PA. Associations of PA with negative affect were not supported by literature^39^. Most recently, Timm et al.^34^ conducted a literature search, revealing that although the number of studies increased tremendously from 14 studies in 2015^38^ to >66 in 2023, evidence remains ambiguous. In line with prior reviews as well as a range of prominent laboratory studies^19–21^, Timm et al. reported stronger support for bidirectional associations between PA and positive affect compared with negative affect. Reciprocal associations of PA with energetic arousal were most consistently supported by literature. For valence, less than half of the studies found significant positive associations with preceding or subsequent PA. Associations between calmness and PA appeared the most heterogeneous, exhibiting both positive and negative associations with PA. Finally, the review reported a high degree of heterogeneity of PA and AWB assessment and statistical methods^34^.

In sum, although intensive longitudinal data on AWB–PA associations are rapidly increasing, evidence remains ambiguous. The concepts and terminology of AWB strongly differ between studies (for example, mood, emotions, affect and core affect) and there is little consistency regarding the items employed. To extensively summarize all evidence, we chose to focus on AWB as a superordinate term of affective constructs^18,21,40^. Further, the above-discussed systematic reviews were based on vote counting, that is, the sorting of studies according to their positive, negative or lack of statistical significance. This provides essential summaries of existing evidence. However, vote counting cannot quantify the existence, strength, directionality and circumstances of PA–AWB associations. Further, given that all studies are weighted equally, conclusions based on the descriptive method of vote counting are limited. Outweighing a significant effect in a study with a large sample size by a non-significant effect in a study with a small sample size is generally inappropriate^41,42^. In the PA–AWB field, matters are further complicated by the variety of methods and statistical models, which results in heterogeneous effect measures and prevents the extraction of effect sizes for traditional meta-analytic approaches. The gold-standard solution is individual participant data (IPD) meta-analysis^43,44^, in which data from publications are collected and uniformly analysed so that original statistical modelling does not affect results. In addition, IPD compiles all data necessary to model within-person processes across time. Here, we employ this method to investigate how PA covaries with AWB in humans’ everyday life.

For the IPD meta-analysis on PA–AWB, we collected 67 datasets from 14 countries worldwide. These datasets included data on 8,223 participants and 321,345 e-diary AWB ratings associated to nearly 1 million hours of PA measurement via accelerometry. The preregistered primary aims of this IPD meta-analysis were to (1) investigate the existence of PA–AWB associations for specific AWB concepts (positive affective states, negative affective states, valence, energetic arousal and calmness), (2) quantitatively assess the strength of PA–AWB associations and (3) compare effect sizes of PA–AWB associations in both directions, that is, AWB and preceding PA, referred to as the antecedent model, as well as AWB and subsequent PA, referred to as the consequent model. Further, we explored the heterogeneity of PA–AWB associations and investigated potential moderators (for example, age and weekday versus weekend).

## Results

### Studies and samples descriptives

**Fig. 1:**
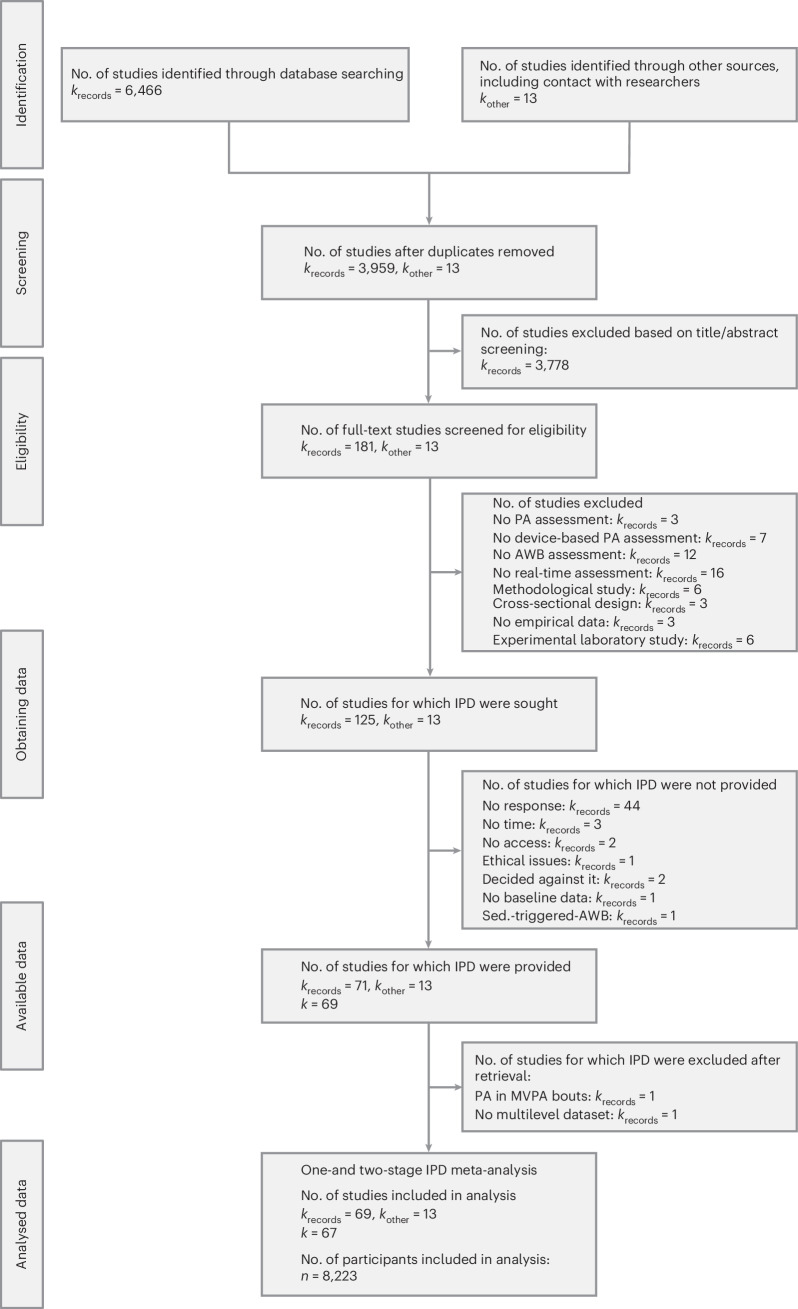
PRISMA flow diagram detailing the search and retrieval process. Illustration of the search and retrieval process for the IPD meta-analysis following the PRISMA guidelines. Sed.-triggered, e-diary prompts triggered when participants were sedentary; *k*_records_, numbers of records identified by database searching; *k*_other_, additional datasets that were not identified by database searching but by contacting the identified authors in the field; *k*, number of unique datasets when considering that several records relied on the same datasets, and several records combined datasets. See Supplementary Information Section 1 for details.

Overall, we combined 67 datasets including 8,223 participants (Fig. 1). Studies varied in observation duration (*M* = 17.72, median of 14, range 1–100 days), PA aggregation intervals (*M* = 159.44, median of 15, range 5–1,440 min) and AWB assessment frequency (*M* = 7.30, median of 5.50, range 1–25 prompts per day). See Supplementary Information Section 1 for details, including AWB and PA measurement as well as e-diary sampling procedures. See Table 1 for descriptives of participants.

**Table 1:** Descriptives of participants (*n* = 8,223) in the IPD meta-analysis

	*k*	*n*	Percent		
Gender/sex	67	8,190			
Female (%)			54.93		
Non-binary (%)^a^			0.06		
	*k*	*n*	*M*	s.d.	Range
Age (in years)	67	8,145	36.33	18.11	8–98
BMI for adults (kg m^−2^)	40	4,675	25.53	5.39	15.06–60.18
Antecedent PA (in study s.d.^b^)	66	7,974	1.03	0.78	0.00–5.48
Consequent PA (in study s.d.^b^)	37	5,127	0.88	0.79	0.00–5.47
Positive affective states (scaled to 1–4)	44	6,384	2.75	0.54	1.00–4.00
Negative affective states (scaled to 1–4)	46	6,711	1.42	0.41	1.00–3.99
Valence (scaled to 1–4)	22	2,036	3.15	0.44	1.06–4.00
Energetic arousal (scaled to 1–4)	19	1,810	2.82	0.41	1.39–4.00
Calmness (scaled to 1–4)	18	1,700	3.06	0.42	1.24–4.00

^a^A non-binary rating was only included in three studies^164–166^.

^b^The variety of methods for assessing PA made a standardization of PA values mandatory. In particular, we divided PA by the studies’ standard deviation of their respective PA measure. Hence, one unit in the standardized PA data summarized in the table corresponds to one standard deviation in the respective study.

### Effects, relevance and strength, and temporal directionality of PA–AWB associations in everyday life

**Fig. 2:**
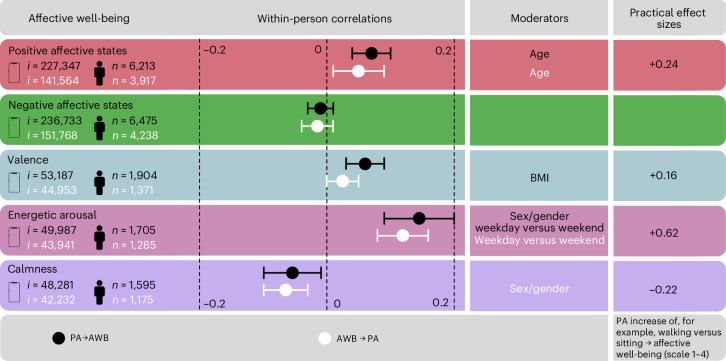
Graphical overview of findings. A summary of findings from all three analysis steps. Left: within-person correlations of AWB with prior (black, antecedent model) and subsequent (white, consequent) PA derived from two-stage IPD meta-analysis (Supplementary Information Section 2) are displayed. The dots represent the weighted mean effects across studies for each outcome. The whiskers represent the 99.2% CI. Middle: moderating influences on associations between AWB and PA derived from one-stage IPD (Supplementary Information Sections 3 and 12) models are illustrated. Right: practical effect sizes are depicted for significant associations of AWB and PA, which were derived from MAI one-stage IPD models (Supplementary Information Sections 3 and 6). Practical effect sizes are exemplified for a PA increase in the range of walking at 5.4 km h^−1^ versus sitting.

In our first series of analyses, we tackled the most prominent questions on PA–AWB associations^32,34,38,39^ including (1) how PA and AWB are associated with each other in everyday life (effects), (2) how practical effect sizes compare with the multitude of other influences in everyday life (relevance and strength) and (3) the temporal directionality of PA–AWB associations (AWB and preceding PA versus AWB and subsequent PA, which we operationalized in antecedent and consequent models). Towards these aims and to gain comprehensive and multifaceted insights on the basis of statistically robust analyses, we applied a three-step procedure. First, we conducted a (multivariate) meta-analysis to estimate an overall effect across AWB concepts and to compare standardized effect sizes derived from multilevel within- and between-person correlations *r* of different AWB concepts (two-stage IPD; Supplementary Information Section 2, analysis step 1). Second, we built a series of multilevel models to investigate the role of individual participant and measurement characteristics including standardized PA values and distinct AWB concepts, controlled for age and sex/gender (one-stage IPD; Supplementary Information Section 3, analysis step 2). Third, to calculate practical effect sizes, we built another series of multilevel models with the same structure (step 2) but restricted the data to studies containing measures of raw PA data (movement acceleration intensity (MAI), in milli-*g*) and added PA aggregation interval as a control variable (MAI one-stage IPD; Supplementary Information Section 3, analysis step 3). A comprehensive range of sensitivity and robustness analyses including ordinal multilevel regressions and bootstrapped confidence intervals (CIs) are presented in Supplementary Information Section 4. We adjusted alpha to 0.05/6 = 0.008, as each AWB concept was tested in three analysis steps and two directions. See Fig. 2 for a graphical overview of the findings.

#### PA predicting subsequent AWB: antecedent model

**Fig. 3:**
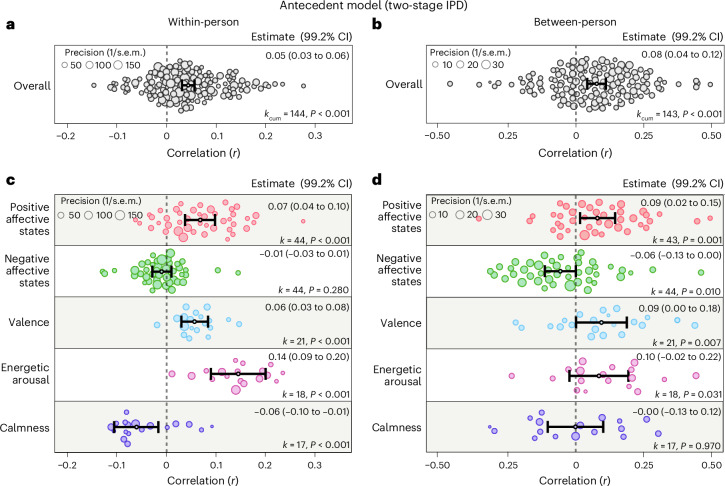
Orchard plot for the antecedent model, that is, PA preceding AWB. **a**,**b**, Within-person (**a**) (*P* = 0.0000000000005) and between-person associations (**b**) (*P* = 0.0000006) of PA with all AWB concepts subsumed in one category. A total of *k*_cum_ = 287 dependent effects from *k* = 65 independent studies serve as experimental units and are included in the analysis. For the overall effect, effects for negative affective states were inverted, as negative effects reflect positive associations with well-being. Black dots represent the weighted mean effect across studies for each outcome. The whiskers represent the 99.2% CI. The cumulative number of effects from all AWB concepts is reflected by *k*_cum_. Significance was assessed on the basis of an alpha threshold of 0.8%. Each dot represents an individual effect measured for that particular outcome and its size reflects the precision and weight in the analysis. Larger dots indicate higher precision as estimated by 1/s.e.m. Significance for the overall moderator effect was assessed using an omnibus *F* test, and the significance of individual moderator levels was determined via a *t*-test. All statistical tests were conducted two-sided. **c**,**d**, Plots are organized according to **a** and **b** but reflect the effects separately for all assessed concepts of AWB within-person (**c**), for example, positive affective states: *P* = 0.00000005; valence, *P* = 0.0000002; energetic arousal, *P* = 0.000000008; and calmness, *P* = 0.0005 and between-person (**d**). The whiskers represent the 99.2% CI. Significance for the overall moderator effect was assessed using an omnibus *F*-test, and the significance of individual moderator levels was determined via a *t*-test. All statistical tests were conducted two-sided.

First, we examined how PA is related to subsequent AWB. To test if there is an overarching association between PA and AWB, we computed a meta-analytic model. In this model, all AWB concepts were subsumed in one larger category, and distinct AWB signs were rectified. This analysis revealed significant within- and between-person associations (two-stage IPD; within, *t*(63) = 9.07, *P* < 0.001, *r* = 0.05, 99.2% CI 0.03 to 0.06; between, *t*(63) = 5.56, *P* < 0.001, *r* = 0.08, 99.2% CI 0.04 to 0.12; Fig. 3a,b). This evidences that overall AWB is indeed positively associated with preceding PA. That is, when a participant is more active than their own average, their AWB in the subsequent AWB assessment is higher compared with assessments with lower preceding PA (within-person association), and on average, more active participants exhibit higher AWB compared with less active participants (between-person association).

**Fig. 4:**
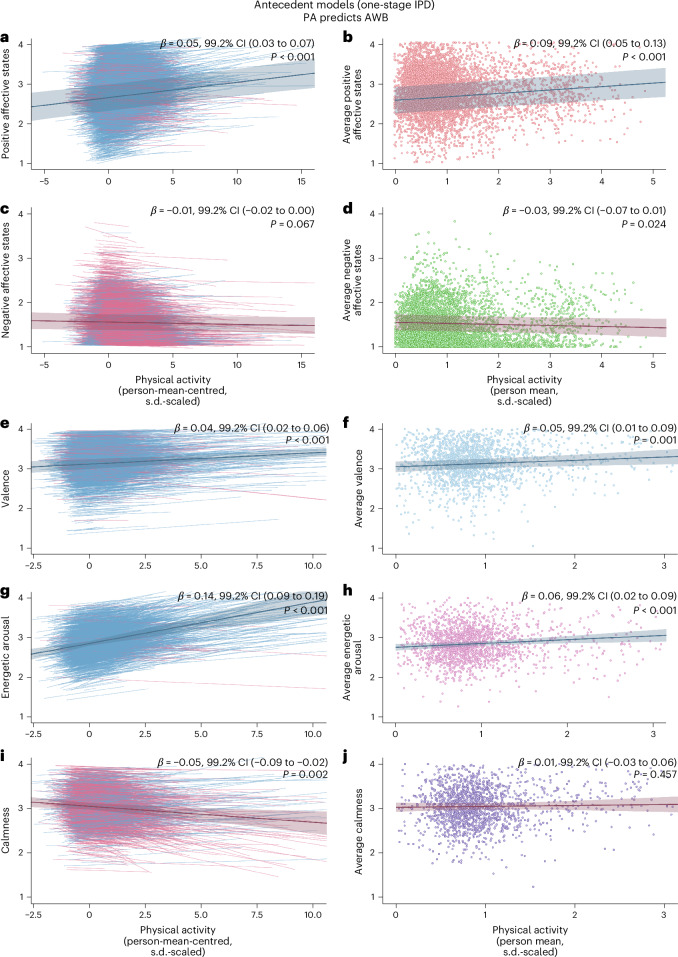
Spaghetti and scatter plots for the antecedent one-stage IPD models, that is, PA preceding AWB. **a**,**c**,**e**,**g**,**i**, The within-person associations of PA and AWB for positive affective states (**a**) (*P* = 0.00000002, *i* = 225,932), negative affective states (**c**) (*i* = 235,537), valence (**e**) (*P* = 0.0000246, *i* = 53,274), energetic arousal (**g**) (*P* = 0.0000008, *i* = 50,105) and calmness (**i**) (*i* = 48,399). The individual regression lines (thin lines) of participants are shown in red (negative slope) and blue (positive slope). The bold line and associated interval represent the conditional fixed effect of person-mean-centred PA with a 99.2% CI. **b**,**d**,**f**,**h**,**j**, The between-person associations of PA and AWB for positive affective states (**b**) (*P* = 0.000000003, *n* = 6,159), negative affective states (**d**) (*n* = 6,429), valence (**f**) (*n* = 1,947), energetic arousal (**h**) (*P* = 0.0001, *n* = 1,751) and calmness (**j**) (*n* = 1,641). Each dot represents one participant’s average PA (*x* axis) and average AWB (*y* axis). The bold line and associated interval represent the conditional fixed effect of person-mean PA with a 99.2% CI. AWB was regressed on person-mean-centred and person-mean PA. Person-mean-centred PA was nested in individuals, which were nested in studies. Multilevel models were controlled for gender/sex and age, and regression coefficients were standardized. Significance was derived using two-sided *t*-tests of regression coefficients, which were adjusted for multiple comparisons (*P* < 0.008). *n* indicates the number of participants and *i* the number of ratings.

We next investigated associations of PA with distinct AWB concepts. Here, we differentiated between two unipolar AWB concepts (positive affective states and negative affective states) and three bipolar AWB concepts (valence, energetic arousal and calmness). We found evidence for differential associations (two-stage IPD; *F*(9, 55) = 19.38, *P* < 0.001). At the within-person level, PA showed the strongest positive associations with energetic arousal, that is, feeling energized and awake^45^, compared with all other AWB measures (two-stage IPD, *t*(55) = 6.79, *P* < 0.001, *r* = 0.14, 99.2% CI 0.09 to 0.20; Fig. 3c and Supplementary Information Section 2). One-stage IPD analysis robustly supports this finding (*t*(17.35) = 7.50, *P* < 0.001, *β* = 0.14, 99.2% CI 0.09 to 0.19, Fig. 4g and Supplementary Table 5.4). Effects translate to an increase in energetic arousal of 0.62 points on a 1 to 4 scale when a participant was walking (499 milli-*g*)^46,47^ versus sitting (20 milli-*g* (refs. ^46,47^); MAI one-stage IPD, *t*(13.83) = 7.32, *P* < 0.001; Supplementary Table 6.4 and Supplementary Fig. 6). Associations of PA with positive affective states, for example, feeling happy, cheerful or active^48^, and valence (that is, feeling well and content)^45^ were smaller but also significantly positive across all analysis steps (positive affective states: two-stage IPD, *t*(55) = 6.31, *P* < 0.001, *r* = 0.09, 99.2% CI 0.02 to 0.15, one-stage IPD, *t*(35.25) = 7.30, *P* < 0.001, *β* = 0.05, 99.2% CI 0.03 to 0.07; Fig. 4a and Supplementary Table 5.1; valence: two-stage IPD, *t*(55) = 5.90, *P* < 0.001, one-stage IPD, *t*(14.16) = 6.14, *P* < 0.001, *β* = 0.04, 99.2% CI 0.02 to 0.06; Fig. 4e and Supplementary Table 5.3). Translated to practice, participants’ positive affective states increased by 0.24 (MAI one-stage IPD, *t*(3.70) = 7.51, *P* = 0.002; Supplementary Table 6.1 and Supplementary Fig. 6) and their valence increased by 0.16 (MAI one-stage IPD, *t*(13.88) = 6.84, *P* < 0.001; Supplementary Table 6.3 and Supplementary Fig. 6) points on a 1–4 scale when they were walking versus sitting. Conversely, we found a significant negative association with PA and subsequent calmness, that is, feeling relaxed and calm^45^, across all analysis steps (two-stage IPD, *t*(55) = 3.70, *P* < 0.001, *r* = −0.06, 99.2% CI −0.10 to −0.01; one-stage IPD, *t*(13.26) = −3.95, *P* = 0.002, *β* = −0.05, 99.2% CI −0.09 to −0.02; Fig. 4i and Supplementary Table 5.5). The typical participant’s calmness decreased by −0.22 points on a 1–4 scale when they were walking versus sitting in their everyday life (MAI one-stage IPD, *t*(9.36) = −4.56, *P* = 0.001; Supplementary Table 6.5 and Supplementary Fig. 6). Negative affective states, that is, feeling sad, anxious or angry^48^, did not reveal a robust significant association with preceding PA on the within-person level (two-stage IPD, *t*(55) = 1.09, *P* = 0.280, *r* = −0.01, 99.2% CI −0.03 to 0.01; Fig. 3c; one-stage IPD, *t*(22.23) = −1.93, *P* = 0.067, *β* = −0.01, 99.2% CI −0.02 to 0.00; Fig. 4c and Supplementary Table 5.2; MAI one-stage IPD, *t*(2.85) = −2.04, *P* = 0.140; Supplementary Table 6.2).

For between-person associations, we found significant associations between PA and positive affective states (two-stage IPD, *t*(55) = 3.46, *P* = 0.001, *r* = 0.09, 99.2% CI 0.02 to 0.15; Fig. 3d; one-stage IPD, *t*(4,304.66) = 5.95, *P* < 0.001, *β* = 0.09, 99.2% CI 0.05 to 0.13; Fig. 4b and Supplementary Table 5.1; MAI one-stage IPD, *t*(496.20) = 3.78, *P* < 0.001; Supplementary Table 6.1) as well as valence (two-stage IPD, *t*(55) = 2.80, *P* = 0.007, *r* = 0.09, 99.2% CI 0.00 to 0.19; Fig. 3d; one-stage IPD, *t*(1,647.42) = 3.24, *P* = 0.001, *β* = 0.05, 99.2% CI 0.01 to 0.09; Fig. 4f and Supplementary Table 5.3; except for MAI one-stage IPD, *t*(1,372.52) = 2.01, *P* = 0.045; Supplementary Table 6.3). Hence, participants who engaged in more PA in general appeared to also experience higher positive affective states and valence than the average less active participant. Evidence for a between-person association of preceding PA and energetic arousal was mixed across analysis steps (two-stage IPD, *t*(55) = 2.22, *P* = 0.031, *r* = 0.10, 99.2% CI −0.02 to 0.22; Fig. 3d; one-stage IPD, *t*(350.13) = 3.87, *P* < 0.001, *β* = 0.06, 99.2% CI 0.02 to 0.09; Fig. 4h and Supplementary Table 5.4; MAI one-stage IPD, *t*(341.36) = 2.33, *P* = 0.020; Supplementary Table 6.4).

#### AWB and subsequent PA: consequent model

**Fig. 5:**
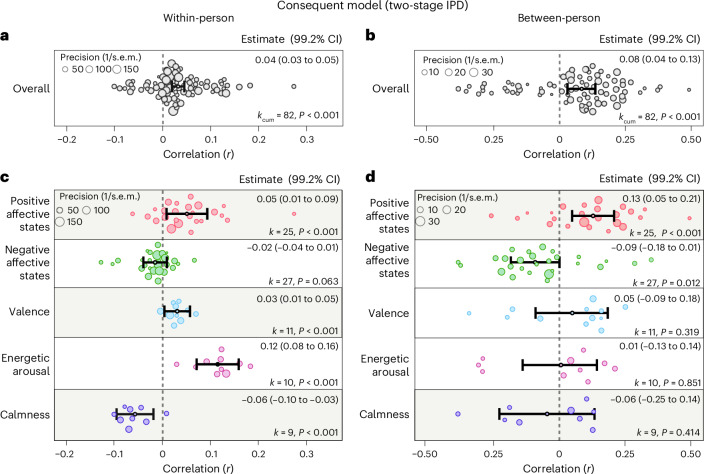
Orchard plot for the consequent model, that is, PA subsequent to AWB. **a**,**b**, Within-person (**a**) (*P* = 0.0003) and (**b**) between-person associations (*P* = 0.00000001) of PA with all AWB concepts subsumed in one category. A total of *k*_cum_ = 164 dependent effects from *k* = 37 independent studies serve as experimental units and are included in the analysis. For the overall effect, effects for negative affective states were inverted, as negative effects reflect positive associations with well-being. The black dots represent the weighted mean effect across studies for each outcome. The whiskers represent the 99.2% CI. The cumulative number of effects from all AWB concepts is reflected by *k*_cum_. Significance was assessed on the basis of an alpha threshold of 0.8%. Each dot represents an individual effect measured for that particular outcome and its size reflects the precision and weight in the analysis. Larger dots indicate higher precision as estimated by 1/s.e.m. Significance for the overall moderator effect was assessed using an omnibus *F*-test, and the significance of individual moderator levels was determined via a *t*-test. All statistical tests were conducted two-sided. **c**,**d**, Plots are organized according to **a** and **b** but reflect the effects separately for all assessed concepts of AWB within-person (**c**), for example, positive affective states, *P* = 0.0006; valence, *P* = 0.0005; energetic arousal, *P* = 0.000000006; and calmness, *P* = 0.00001; and between-person (**d**), for example, positive affective states: *P* = 0.00009. The whiskers represent the 99.2% CI. Significance for the overall moderator effect was assessed using an omnibus *F* test, and the significance of individual moderator levels was determined via a *t*-test. All statistical tests were two sided.

Next, we investigated associations of momentary AWB with subsequent PA in everyday life. Our two-stage IPD across all AWB concepts revealed significant within- and between-person associations (within, *t*(35) = 7.43, *P* < 0.001, *r* = 0.04, 99.2% CI 0.03 to 0.05; between, *t*(35) = 4.01, *P* < 0.001, *r* = 0.08, 99.2% CI 0.04 to 0.13; Fig. 5a,b). That is, when a participant exhibited higher AWB than their own average, their subsequent PA was higher compared with assessments with lower prior AWB (within-person association), and on average, participants with higher AWB showed higher overall levels of PA compared with participants with lower AWB (between-person association).

**Fig. 6:**
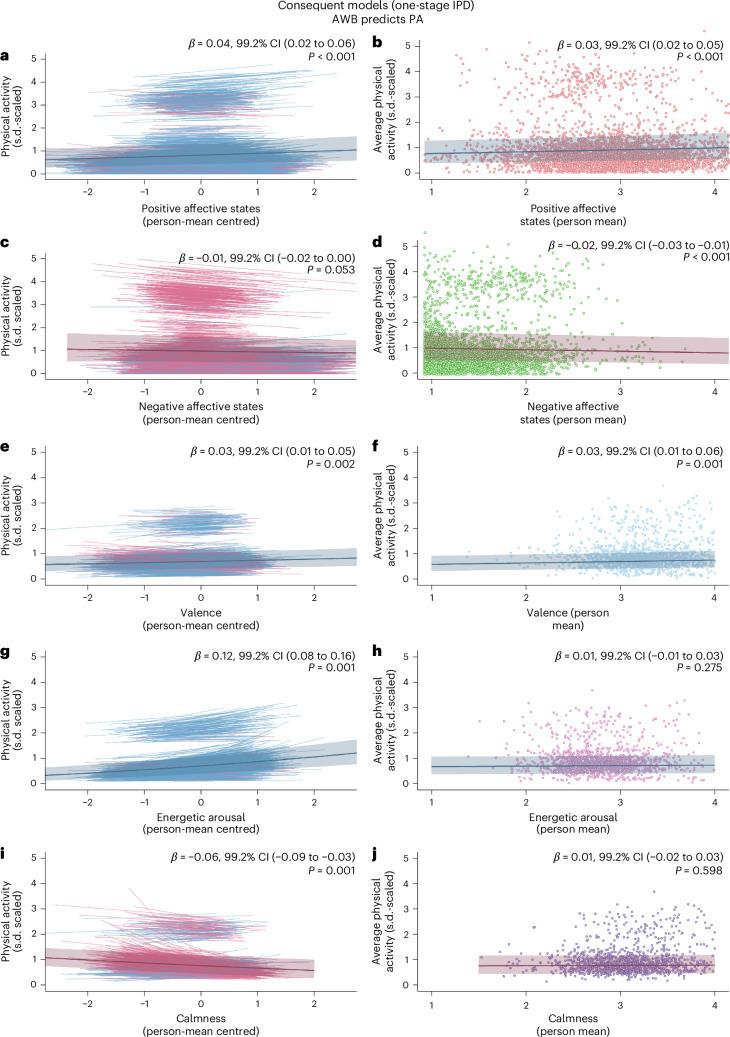
Spaghetti and scatter plots for the consequent one-stage IPD models, that is, PA subsequent to AWB. **a**,**c**,**e**,**g**,**i**, The within-person associations of AWB and PA for positive affective states (**a**) (*P* = 0.0001, *i* = 140,170), negative affective states (**c**) (*i* = 150,118), valence (**e**) (*i* = 44,251), energetic arousal (**g**) (*P* = 0.00005, *i* = 43,270) and calmness (**i**) (*i* = 41,561). The individual regression lines (thin lines) of participants are shown in red (negative slope) and blue (positive slope). The bold line and associated interval represent the conditional fixed effect of person-mean-centred AWB with a 99.2% CI. **b**,**d**,**f**,**h**,**j**, The between-person associations of AWB and PA for positive affective states (**b**) (*P* = 0.0000000000007, *n* = 3,864), negative affective states (**d**) (*P* = 0.00006, *n* = 4,179), valence (**f**) (*n* = 1,354), energetic arousal (**h**) (*n* = 1,271) and calmness (**j**) (*n* = 1,161). Each dot represents one participant’s average AWB (*x* axis) and predicted average PA (*y* axis). The bold line and associated interval represent the conditional fixed effect of person-mean AWB with a 99.2% CI. Of note, in *k* = 5 studies included, the intercept of the outcome is systematically larger owing to the limited variability or the higher average of the outcome, leading to larger values on the standardized PA score. This can be seen in all panels where several lines/dots are clustered above the fitted lines. Removing these data points (see Supplementary Information Section 4.4 for sensitivity analysis) did not alter the conclusions drawn, suggesting that these between-study differences in mean outcome levels did not bias our results. PA was square-root transformed and regressed on person-mean-centred and person-mean AWB. Person-mean-centred AWB was nested in individuals, which were nested in studies. Multilevel models were controlled for gender/sex and age, and regression coefficients were standardized. Significance was derived using two-sided *t*-tests of regression coefficients, which were adjusted for multiple comparisons (*P* < 0.008). *n* indicates the number of participants and *i* the number of ratings. For visualization, predictions were back-transformed to the original response scale (instead of square-root transformed scale). Standard errors remain on the square-root-transformed scale.

The AWB concepts showed distinct associations with subsequent PA (two-stage IPD, *F*(9, 27) = 29.71, *P* < 0.001). At the within-person level, energetic arousal showed stronger positive associations with subsequent PA compared with all other measures of AWB (two-stage IPD, *t*(27) = 8.35, *P* < 0.001, *r* = 0.12, 99.2% CI 0.08 to 0.16; Fig. 5c and Supplementary Information Section 2). Strong associations of energetic arousal and subsequent PA were fully supported by one-stage IPD analyses (*t*(7.24) = 8.47, *P* < 0.001, *β* = 0.12, 99.2% CI 0.08 to 0.16; Fig. 6g and Supplementary Table 7.4; MAI one-stage IPD, *t*(614.55) = 21.10, *P* < 0.001; Supplementary Table 8.4). Moreover, positive affective states (two-stage IPD, t(27) = 3.89, *P* < 0.001, *r* = 0.05, 99.2% CI 0.01 to 0.09; Fig. 5c; one-stage IPD, *t*(18.26) = 4.87, *P* < 0.001, *β* = 0.04, 99.2% CI 0.02 to 0.06; Fig. 6a and Supplementary Table 7.1; except for MAI one-stage IPD, *t*(2.87) = 5.63, *P* = 0.013; Supplementary Table 8.1) and valence (two-stage IPD, *t*(27) = 3.98, *P* < 0.001, *r* = 0.03, 99.2% CI 0.01 to 0.05; Fig. 5c; one-stage IPD, *t*(8.74) = 4.20, *P* = 0.002, *β* = 0.03, 99.2% CI 0.01 to 0.05; Fig. 6e and Supplementary Table 7.3; except for MAI one-stage IPD, *t*(6.03) = 3.38, *P* = 0.015; Supplementary Table 8.3) demonstrated significant positive and calmness (two-stage IPD, *t*(27) = 5.30, *P* < 0.001, *r* = −0.06, 99.2% CI −0.10 to −0.03; Fig. 5c; one-stage IPD, *t*(5.96) = −5.71, *P* = 0.001, *β* = −0.06, 99.2% CI −0.09 to −0.03; Fig. 6i and Supplementary Table 7.5; MAI one-stage IPD, *t*(616.34) = −10.48, *P* < 0.001; Supplementary Table 8.5) significant negative associations with subsequent PA. Negative affective states showed no robust significant association with subsequent PA in any analysis step (two-stage IPD, *t*(27) = 1.94, *P* = 0.063, *r* = −0.02, 99.2% CI −0.04 to 0.01; Fig. 5c; one-stage IPD, *t*(12.42) = −2.13, *P* = 0.053, *β* = −0.01, 99.2% CI −0.02 to 0.00; Fig. 6c and Supplementary Table 7.2; MAI one-stage-IPD: *t*(257.20) = −2.48, *P* = 0.014; Supplementary Table 8.2).

At the between-person level, only positive affective states consistently showed a positive association with subsequent PA across all analysis steps (two-stage IPD, *t*(27) = 4.60, *P* < 0.001, *r* = 0.13, 99.2% CI 0.05 to 0.21; Fig. 5d; one-stage IPD, *t*(3,697.86) = 7.20, *P* < 0.001, *β* = 0.03, 99.2% CI 0.02 to 0.05; Fig. 6b and Supplementary Table 7.1; MAI one-stage IPD, *t*(619.35) = 3.27, *P* = 0.001; Supplementary Table 8.1). Whether participants with on average lower negative affective states (two-stage IPD, *t*(27) = 2.68, *P* = 0.012, *r* = −0.09, 99.2% CI −0.18 to 0.01; Fig. 5d; one-stage IPD, *t*(3,861.76) = −4.04, *P* < 0.001, *β* = −0.02, 99.2% CI −0.03 to −0.01; Fig. 6d and Supplementary Table 7.2; one-stage MAI IPD: *t*(604.85) = −1.17, *P* = 0.242; Supplementary Table 8.2) or higher valence (two-stage IPD, t(27) = 1.02, *P* = 0.319, *r* = 0.05, 99.2% CI −0.09 to 0.18; Fig. 5d; one-stage IPD, *t*(1,188.32) = 3.21, *P* = 0.001, *β* = 0.03, 99.2% CI 0.01 to 0.06; Fig. 6f and Supplementary Table 7.3; one-stage MAI IPD: *t*(794.75) = 2.12, *P* = 0.035; Supplementary Table 8.3) also exhibited higher PA was mixed across analysis steps and robustness analyses; therefore, we assume no association.

Drawing on a conceptual framework from affective psychology^49^, we conducted sensitivity analyses for AWB operationalizations. For bipolar conceptualizations of AWB, we jointly analysed studies (*k*_ant_ = 17, *k*_con_ = 8) assessing valence, energetic arousal and calmness with the psychometrically evaluated short-version of the Multidimensional Mood Questionnaire^45^. The short-version of the Multidimensional Mood Questionnaire has been referenced as a measure of core affect^50–54^. For unipolar conceptualizations of AWB, there is no similarly established and psychometrically evaluated measure in daily life research and the collected data. We concentrated our analysis on all studies that followed the Positive and Negative Affect Schedule (PANAS)^48^ and summarized high-activation items in positive and negative affect (*k*_*ant*_ = 10, *k*_*con*_ = 4). It needs to be kept in mind that this operationalization is not undisputed (see, for example, refs. ^55,56^ for a critical discussion of the PANAS). The sensitivity analyses support our main findings. See Supplementary Information Sections 4.5, 4.6 and 9 for a more detailed description of the theoretical considerations and the resulting analyses.

#### Effect directions

In summary, this IPD meta-analysis on PA–AWB associations in everyday life consistently shows PA to be associated with subsequent valence, calmness, energetic arousal and positive affective states but not with subsequent negative affective states on a within-person level. For the reverse time-order, we consistently found within-person associations of positive affective states, valence, energetic arousal and calmness with subsequent PA but not for negative affective states. Between participants, PA was robustly associated with subsequent positive affective states and valence but with none of the other AWB concepts (calmness, energetic arousal and negative affective states). For between-associations of AWB and subsequent PA, we found only positive affective states to be consistently associated with subsequent PA.

#### Relevance and strength

When translating these effect sizes into practice to estimate their relevance in humans’ everyday life, their magnitude is comparable to other daily life activities. Changing one’s activity in the range of, for example, from sitting to walking is associated with an increase in AWB from 0.16 points (scale 1–4) for valence (the weakest yet significant effect) to 0.62 points (scale 1–4) for energetic arousal (the strongest effect). Comparing PA–AWB effects to an existing and prominent large-scale daily life study^57^ makes their relevance and strength tangible: overall, our data across five AWB concepts are in line with the Killingsworth and Gilbert study on happiness during self-reported activities (17,775 ratings)^57^, in which taking a walk was related to an increase in happiness of approximately 0.21 points compared with a person’s average happiness and exercising to an increase of approximately 0.36 points when transferring effects to a scale from 1–4 (Supplementary Fig. 6). In the Killingsworth and Gilbert study, other daily life activities such as listening to music, playing and talking exhibited effects of similar size; watching television, reading, shopping and relaxing were related to a smaller increase in happiness of approximately 0.06 points.

#### Temporal directionality

We compiled evidence on the temporal directionality of PA–AWB versus AWB–PA associations. Our analyses revealed significant within-person associations of both preceding and subsequent PA with positive affective states, valence, energetic arousal and calmness but no within-person associations with negative affective states. To statistically test for temporal directionality, we conducted a two-stage IPD meta-analysis considering both temporal directions in one model (Supplementary Information Section 10). Post-hoc comparisons revealed no significant differences in effects between both time-orders for all PA–AWB associations. Hence, there was no evidence that the PA–AWB association is larger in one or the other temporal direction.

### Heterogeneity of PA–AWB associations

Variation in the direction and strength of PA–AWB associations between individuals has not been representatively studied to date but promises to come with high relevance for precision medicine and therefore remains under intensive discussion^58–62^. Employing the full strength of the IPD meta-analysis, we here found considerable differences in the direction and strength of PA–AWB associations between individuals (Figs. 4a,c,e,g,i and 6a,c,e,g,i). This offers a promising treatment target for individualized and thus expedient interventions. In particular, the association between PA and subsequent negative affective states appears the most heterogeneous (40.22% positive and 59.78% negative) across participants, whereas we found the most homogeneous effects for PA and energetic arousal. Nearly all participants (>95%) felt more energetically aroused when PA prior or subsequent to the e-diary prompt was higher.

### Moderators of PA–AWB associations

The evidence on the heterogeneity of PA–AWB associations motivated us to inspect potential correlates of person and measurement characteristics with the within-person associations of AWB and PA (for moderator analyses for study characteristics see Supplementary Information Section 11). Although the range of possible moderators appears immense, we focused our analyses on four epidemiologically potent moderators^63,64^: age (person-level), gender/sex (person-level), body mass index (BMI; person-level) and weekday (relative to weekend; measurement-level). Moderating associations emerged, which differed depending on the AWB concept (only significant moderation analyses are presented; see Supplementary Information Section 12 for a comprehensive report of moderator analyses).

#### Age

**Fig. 7:**
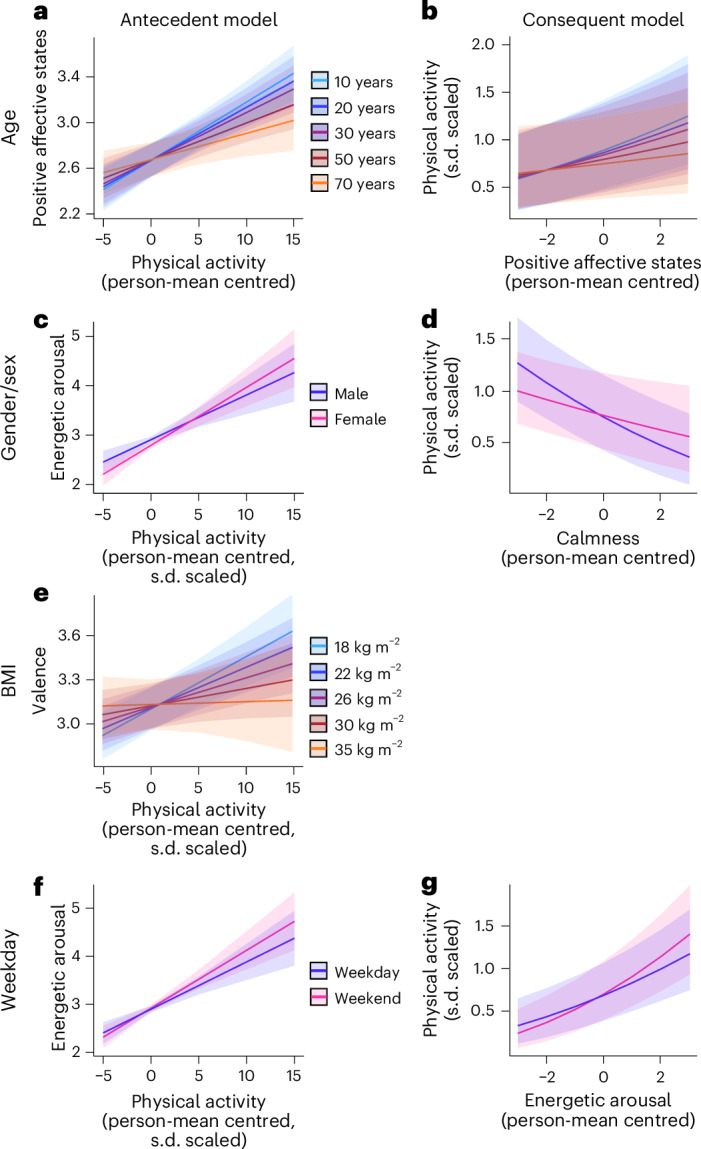
Plots of predicted moderation effects of PA and AWB associations in antecedent (left) and consequent (right) one-stage IPD models with 99.2% CIs. **a**,**b**, The moderating influence of age on the association of positive affective states with PA. Younger individuals engaged in more PA before (**a**) and after (**b**) reporting high positive affective states compared with older individuals, for whom the bidirectional association of positive affective states and PA was less pronounced. **c**,**d**, The moderating influence of sex/gender on the association of energetic arousal and calmness with PA; women felt more energetically aroused after being physically active than men (**c**); men were more physically active than women when feeling less calm than usual; however, they were less physically active than women when reporting more calmness than usual (**d**). **e**, The moderating influence of BMI on the association of valence with preceding PA. Individuals with a lower BMI reported higher valence after being more physically active than usual compared with individuals with a higher BMI. Given that height and weight-based BMI values are, in general, inappropriate for underaged individuals, this moderator analysis was conducted for adults only. **f**,**g**, The moderating influence of weekday versus weekend on the association of energetic arousal and PA. On weekends, the bidirectional association of PA and energetic arousal was stronger than on weekdays.

Younger individuals engaged in more PA before (*t*(720.63) = −3.74, *P* < 0.001, *β* = −0.01, 99.2% CI −0.02 to −0.00; Fig. 7a and Supplementary Table 12.1a) and after (*t*(179.58) = −3.01, *P* = 0.003, *β* = −0.02, 99.2% CI −0.04 to −0.00; Fig. 7b and Supplementary Table 12.2a) reporting high positive affective states compared with older individuals, for whom the bidirectional association of positive affective states and PA was less pronounced.

#### Gender/sex

Women felt more energetically aroused after being physically active than men (*t*(808.61) = 3.18, *P* = 0.002, *β* = 0.04, 99.2% CI 0.01 to 0.07; Fig. 7c and Supplementary Table 12.3d). Men were more physically active than women when feeling less calm than usual. However, they were less physically active than women when reporting more calmness than usual (*t*(630.44) = 3.52, *P* < 0.001, *β* = 0.10, 99.2% CI 0.02 to 0.18; Fig. 7d and Supplementary Table 12.4e).

#### BMI

Adults with a lower BMI reported higher valence after being more physically active than usual compared with adults with a higher BMI (*t*(620.19) = −2.87, *P* = 0.004, *β* = −0.02, 99.2% CI −0.03 to −0.00; Fig. 7e and Supplementary Table 12.5c).

#### Weekday versus weekend

On weekends, the bidirectional association of PA and energetic arousal was stronger than on weekdays (antecedent model, *t*(17,918.03) = −3.36, *P* < 0.001, *β* = −0.02, 99.2% CI −0.03 to −0.00; Fig. 7f and Supplementary Table 12.7d; consequent model, *t*(35,430.89) = −4.04, *P* < 0.001, *β* = −0.03, 99.2% CI −0.05 to −0.01; Fig. 7g and Supplementary Table 12.8d).

Finally, given the urgent need to further research heterogeneity in PA–AWB associations between participants towards precision medicine approaches, we explored potential low-base effects. We plotted participants’ individual slopes and intercepts against each other and explored the random effects correlations within the one-stage IPD multilevel models (Supplementary Information Section 13). The data suggest that individuals exhibiting higher average negative affective states show stronger reductions in their momentary negative affective states after engaging in more PA than usual compared with individuals with low negative affective states (*r* = −0.13, 99.2% CI −0.23 to −0.04). For positive affective states, the data suggest that individuals exhibiting lower average positive affective states show stronger increases in their momentary positive affective states after engaging in more PA than usual compared with individuals with high positive affective states (*r* = −0.09, 99.2% CI −0.17 to −0.01).

## Discussion

This preregistered IPD meta-analysis provides evidence on directions and strength, relevance, temporal directionality, heterogeneity and moderators of PA–AWB relations in humans’ everyday life. Associations vary depending on AWB concepts and level of analysis: Within-persons, PA is consistently and positively associated with positive affective states, valence and energetic arousal. Yet, PA is negatively associated with calmness and, overall, not significantly linked to negative affective states. Between-persons, PA only robustly associates with positive affective states. The practical effect sizes are comparable to other daily life activities, with energetic arousal evincing the strongest within-person association with PA. Associations substantially vary in direction and strength between individuals, but age, gender/sex, BMI and weekday/weekend partially explain PA–AWB association differences between persons.

Overall, the data show that PA is robustly associated with AWB as humans go about their daily routines. The evidence on pronounced within-person PA–AWB relations substantiates the general sentiment of cutting-edge health behaviour theory development. Traditional health behaviour theories emphasizing the rational and cognitive determination of health behaviour^65–69^ show limited explanatory power^11,14,17,70^. To tackle the vital issue of health behaviour decision-making and public health, novel models of health behaviour build on hedonic theories of behaviour^11–14,71–76^ and take into account that affective, impulsive processes shape health behaviours, including PA engagement. These affective, impulsive processes contrast against rational, reflective processes and regained attention subsequent to Kahnemann’s ‘thinking slow, thinking fast’ proposal^77^. Put simply, novel health behaviour models emphasize that it is not only the knowledge on positive health effects that drives PA engagement but actual experiences and affective feelings related to PA.

This large-scale IPD provides empirical evidence to understand the micro-temporal processes of PA–AWB relations. This is crucial for future research on precision medicine approaches such as personalized micro-interventions in everyday life based on real-time data (so-called ecological momentary interventions or just-in-time adaptive interventions)^78^. Ultimately, the micro-temporal processes promise to allow for individually and context-tailored real-time support to prevent and intervene on a broad range of non-communicable diseases and thereby contribute to public health. As variations in AWB are not only thought to be linked to PA engagement but also to human experiences and behaviours in general, it is tempting to speculate that PA may indirectly also influence other aspects of daily life, such as attention, memory, decision-making or learning, via fluctuations in AWB. General models of decision-making, for example, postulate that incidental factors, such as lingering moods and emotions, can impact decisions^79,80^. Hence, PA-related changes in AWB unrelated to the object of decisions may nevertheless subtly affect how individuals make decisions in their daily lives^79^. However, these implications need to be considered against the background of the observational nature of the data meta-analysed and the resulting potential for residual confounding. Although this meta-analysis on intensive longitudinal data of daily life studies on PA and AWB comes with highest ecological validity, the designs of PA–AWB studies included are observational. That is, they do not include experimental manipulation to control the impact of confounding variables (that is, variables impacting the PA–AWB associations). As a result, unmeasured (time-varying) confounding variables (for example, weather conditions) could have affected the findings. Ideally, future Ecological Momentary Assessment (EMA) studies consider the impact of these variables more thoroughly. For studies focusing on the PA–AWB association, a quasi-experimental design could be adopted^81,82^. For studies examining the AWB–PA association, potential confounding variables should be systematically identified and accounted for in the EMA design and the analyses^83,84^.

The data reveal an especially pronounced covariation of PA with feelings of energy across time, with PA–energetic arousal associations yielding considerably higher effect sizes than PA–calmness and PA–valence associations. The PA–positive affective states association strength positions itself in between. Accordingly, PA–energetic arousal and PA–positive affective states relations present themselves as particularly salient targets to shape theories of health behaviour change and develop future precision medicine approaches. For example, PA–energy associations can be especially valuable in the prevention and intervention of fatigue symptomatology^85–87^ and in the treatment of affective disorders^8,36,88^. Overall, the practical effect sizes of PA–AWB relations derived from our IPD meta-analysis are comparable to other daily life activities (for example, reading, listening to music and eating) that were investigated in a large-scale study by Killingsworth and Gilbert^57^. For feelings of energy, they appear considerable. It has to be taken into account, however, that the Killingsworth and Gilbert^57^ study investigated happiness, whereas we present different concepts of AWB.

Our IPD meta-analytic findings can guide future research towards individualized, tailored and expedient interventions utilizing PA–AWB relations. First, given its considerable effect size, interactions of PA with energetic arousal appear as important future research targets. Considering the overall differential AWB associations documented, it can critically contribute to existing health behaviour theories and even bring up novel health behaviour models. Of note, our meta-analysis revealed a pronounced variability between studies in the instruments applied for the assessment of AWB, as well as a limited coverage of certain AWB dimensions. This appears as a challenge to daily life studies. We conducted sensitivity analyses revealing that our main analyses are robust against assignments of AWB constructs to positive affective states, negative affective states, valence, energetic arousal and calmness (Supplementary Information Sections 4.5, 4.6 and 9). We recommend future studies to provide clear conceptual rationales and rely on psychometrically evaluated measures when conducting a study on PA–AWB associations in daily life, for example, guided by a framework from affective psychology^49^. The development of formalized and testable psychological theories^89–91^ as well as item pools (for example, the Experience Sampling Method Item Repository^92^) for daily life research can further contribute to greater consensus. Second, evidence is in favour of temporally bidirectional PA–AWB associations. However, aggregated data are not suited to evidence causal conclusions. Dynamic modelling approaches such as continuous-time analyses^93,94^ enriched by experimental manipulation in everyday life are essential to approach a future PA–AWB Frequency, Intensity, Time and Type framework^95^.

Third, our data clearly show PA–AWB relations to vary in direction and strength between individuals and accumulate evidence for sex/gender-, BMI- and weekday-dependent relationships. Although we received no clear evidence for uniform moderation patterns across AWB concepts, distinct interactions emerged. Younger and low-BMI individuals exhibit stronger associations of PA with subsequent valence or positive affective states. In light of motivational and particularly hedonic theories, which postulate that individuals strive to maximize their well-being, the less positive affective response to PA in high-BMI and older individuals plausibly fits epidemiological observations of reduced PA in these groups^2,96–98^. High-BMI and older adults may have weaker associations between PA and subsequent positive affective states and valence because PA may trigger more feelings of discomfort, pain, heat, stain or other unpleasant physiological states in these groups. To enhance PA engagement, it thus appears especially important to identify putative catalysts of the PA–AWB associations in vulnerable groups^99^: Sociodemographic, biological and contextual factors may facilitate effects of PA on pleasure to reinforce future PA engagement and lower the threshold for AWB to evoke PA. This may comprise aspects such as walkability^100,101^, green spaces^102–104^ or the social context^105–107^. Given neurobiological^108^ and laboratory^109–111^ findings of blunted PA–AWB responses in high-BMI individuals, it is also tempting to speculate that the intensity of PA differentially affects neurobiological processes and, thus, constitutes a candidate for precision intervention^111,112^. Next, for men, calmness is more strongly associated with subsequent PA than for women, yet women feel more energetically aroused after being physically active than men. Although the assessment of gender/sex differed between studies and only few studies included a non-binary gender option, this differential pattern of PA–AWB associations may relate to distinct autonomic nervous system properties seen in people with female versus male biology, such as differences in hormones^113^ or heart rate variability^114,115^. If substantiated in future proposals, this suggests that biologically informed prevention and treatment approaches may be particularly salient for PA engagement and AWB. Finally, on weekends, the bidirectional association of PA and energetic arousal is stronger than on weekdays, which partially matches findings on differences in work-related versus leisure-time PA impacts on both mental^116,117^ and physical^118^ health. Particularly, studies have found that although leisure-time PA is consistently linked with reduced cardiovascular risk and depression, occupational PA appears to have negative health effects. Hence, accounting for the day-of-week context appears of particular importance for future precision medicine approaches. Intriguingly, the data also reveal that high-negative affective states individuals can exhibit substantial benefit from PA. Thus, this work sets the basis for differential investigations within specific individuals, subgroups and contexts for leveraging diverse personal or situational characteristics of the ideographic AWB–PA relationship in follow-up randomized clinical trials. Following recent criticism on the exclusive focus on social and contextual determinants to the detriment of biological and genetic determinants of PA engagement^119,120^, future research can gain extensive knowledge on the idiographic associations between PA and AWB by focusing not only on a wide range of person–situation–environment characteristics but also through studying biological and genetic factors as well.

Several limitations need to be taken into account. First, although we searched the most prominent literature databases and asked all authors for additional unpublished data, we cannot rule out that representative datasets were overlooked by our literature search. However, we received and compiled a total of 67 datasets across 14 countries comprising a highly representative sample. Second, although a major strength of this IPD meta-analysis is the sole inclusion of device-based assessed PA, studies vary in measurement methods. To rule out residual confounding influences, we adjusted to these differences by means of (1) statistical standardization, (2) testing for potential moderation and (3) securing findings by introducing a third analysis step only applying streamlined raw PA data. Findings remained robust across all analysis steps and regardless of PA assessment procedures. Third, studies varied in the AWB concepts investigated and, within concepts, the specific items employed. We streamlined our analyses across unipolar (positive affective states and negative affective states) and bipolar (valence, calmness and energetic arousal) AWB concepts. Despite the heterogeneity of AWB assessments, our meta-analysis reveals that PA can explain meaningful variance across AWB concepts.

In conclusion, this work shows the critical relevance of PA–AWB associations within humans and extends the state of knowledge on how PA relates to AWB in everyday life. It supports the general sentiment of ‘affectivism’^18^ and of motivational theories that highlight the relevance of affective processes. The findings argue for a dominant role of momentary AWB fluctuations within humans to shape behaviour and experiences. The heterogeneity of PA–AWB associations underlines the importance of considering contextual factors, especially in vulnerable groups. Considering aspects of the social and physical environment of individuals, such as walkability or urban green spaces, appears of utmost importance for human decision-making and public health strategies. The collected data provide an evidence base for precision medicine interventions that may ultimately alleviate the inactivity pandemic and enhance human health around the globe.

## Methods

This IPD meta-analysis followed established procedures (PRISMA-IPD; Supplementary Information Section 14) and was preregistered on 21 March 2022 (CRD42022303509; https://www.crd.york.ac.uk/prospero/display_record.php?ID=CRD42022303509). The present IPD meta-analysis complies with all relevant ethical regulations. An ethics exemption was obtained from the ethics committee at the Faculty of Sport Science, Ruhr University Bochum, Germany, based on the ethics approvals of individual studies included in the meta-analysis (reference: EKS S 03/2022).

All preprocessing, harmonization, checking, merging and analysing of data was performed using R (version 4.4.0 and 4.5.2^121^), the interface RStudio^122^ and various R packages^123–140^.

### Data sources and study selection

Records were considered for inclusion if (1) PA was quantified using technical devices (for example, accelerometers) to reduce distortions (for example, cognitive heuristics^35,141,142^); (2) AWB was self-reported (for example, electronic diaries on smartphones) to comply with state-of-the-art procedures for reliable and ecologically valid assessments of psychological states^142^; (3) assessments were conducted for at least 1 day to minimize (diurnal) confounds^143^; and (4) aggregated time frames did not exceed 24 h to reduce well-known recall-bias^142^. Records studying people of all ages with and without mental or physical diseases were included to provide a summary of PA–AWB associations across populations.

We excluded records if (1) PA measurements were conducted under controlled or artificial conditions (for example, laboratory or research settings, interventions and in-patient treatments); (2) records employed retrospective questionnaires on PA and AWB; and (3) data collection occurred one time only.

We searched the databases Web of Science, PubMed, Scopus, SPORTDiscus and PsycINFO until December 2023 using the search terms ‘ecological momentary assessment’, ‘physical activity’ and ‘sedentary behavior’ as well as their synonyms (see Supplementary Information Section 15 for the adapted Boolean operators) and search terms for the umbrella term AWB; these comprise ‘mood’ or ‘emotion’ or ‘affect’. The search scope was confined to articles published in English, without restrictions on publication year. Records were first selected on the basis of a screening of titles. Abstracts and full-texts were independently reviewed for eligibility (M.G., I.T.; in case of non-correspondence: M.R. as third reviewer; Fig. 1).

### Data synthesis

Corresponding authors of selected studies were contacted to retrieve IPD and also asked for additional unpublished IPD meeting the inclusion criteria. The deadline for all data transfers was set to 31 October 2024. During data collection, two retrieved datasets were excluded. In one dataset, PA and AWB were only available for exercise bouts, not for time slots preceding or following the e-diary prompt^144^. The dataset of another study only included one aggregate measure of PA per participant so that the within-associations of PA and AWB could not be investigated^145^. Variables to be harmonized were chosen upon intensive discussion in the IPD-coordinating team (J.R., I.T., G.B., M.G., J.P. and M.R.).

### Outcomes

In the antecedent models, AWB served as the primary outcome. There is an ongoing debate on how to conceptualize, define and operationalize AWB^18,55,76,146–152^. A variety of terms such as core affect, feelings, emotions and moods, exists, which are, to date, not consistently used (see^18^ and Supplementary Information Section 9 for an extensive discussion). Prominent models of AWB include Russell’s^153^ circumplex model, Watson et al.’s two-dimensional approach operationalized within the PANAS^48^ and Thayer’s concept of activation comprising tension and wakefulness^146,154,155^. Recently, three-dimensional theories of AWB, which include the dimensions of valence, calmness and energetic arousal^45,156^, have gained increasing interest. To extensively summarize the heterogeneous literature on PA and AWB, we focused on five constructs from two- and three-dimensional models of AWB that were frequently assessed in real-life studies on AWB–PA associations^34^: positive affective states, negative affective states, valence, energetic arousal and calmness. We differentiated positive and negative affective states (as unipolar AWB concepts) from bipolar AWB concepts (that is, valence, energetic arousal and calmness). If studies included none of the predefined AWB dimensions, the assessed AWB measures were assigned to positive affective states, negative affective states, valence, energetic arousal and calmness by five researchers independently (M.R., M.G., G.B., I.T. and J.R.); a unipolar item of ‘fatigue’ was, for example, assigned to negative affective states, a unipolar item of ‘pleasure’ (that is, ‘not at all pleasurable’ to ‘totally pleasurable’) to positive affective states. ‘Mood’ was depending on whether it was assessed unipolarly (for example, DABS Mood^157^) or bipolarly (that is, ‘very unpleasant’ to ‘very pleasant’, ‘sad’ to ‘happy’ and ‘very negative’ to ‘very positive’) assigned to positive affective states and negative affective states (unipolar) or to valence (bipolar).

In the consequent models, PA was the primary outcome. If multiple PA measures were available, we followed a predefined procedure. To support the investigation of momentary PA–AWB associations, we preferred PA aggregated over the smallest available time interval. If multiple PA operationalizations were included in a dataset, we preferred (1) measures comprising the full PA intensity spectrum (MAI, counts, steps, MET > moderate-to-vigorous PA (MVPA), light PA (LIPA) and time stepping), (2) streamlined measures involving less preprocessing (MAI > counts, steps) and (3) measures capturing more frequent activities (LIPA > time stepping > MVPA). The full hierarchy is: MAI > counts > steps > MET > LIPA > time stepping > MVPA.

**Extended Data Fig. 1:**
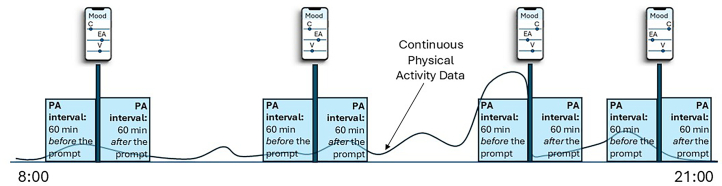
Study design of ambulatory assessment studies investigating the relation of affective well-being (AWB) and physical activity (PA) in daily life. AWB is assessed at multiple discrete time points across a day/multiple days. PA is measured continuously and aggregated over specific time intervals preceding or following the AWB rating. We have taken the chronological order into account by separately computing antecedent (PA preceding the AWB rating) and consequent (PA following the AWB rating) models.

All studies assessed AWB at multiple discrete time points. PA was measured continuously in all studies. To analyse the relation of AWB and PA, researchers aggregated PA in certain time intervals, for example, 60 min preceding or following the AWB rating. Thus, antecedent models incorporated PA aggregated before the AWB rating. Consequent models incorporated PA aggregated following the AWB rating. See Extended Data Fig. 1 for a rough visualization of the study design across studies.

To increase robustness of our findings, we included three main effect measures: First, we chose a two-stage IPD approach, in which multilevel correlations served as effect measures. In the second and third analysis step, we chose a one-stage IPD approach and set up separate multilevel models for each AWB dimension. Here, beta coefficients served as effect measures (see Figs. 4 and 6 for standardized and Supplementary Information Sections 5–8 for unstandardized effects).

### Analysis

We tackled the most prominent questions on PA–AWB associations^32–34,38,39^ on (1) how PA and AWB are associated to each other in everyday life (effects), (2) how practical effect sizes compare with the multitude of other influences in human everyday life (relevance and strength) and (3) the temporal directionality of PA–AWB associations (PA and subsequent AWB versus AWB and subsequent PA). We calculated a two-stage and two one-stage IPD analyses for both antecedent and consequent models: First, we conducted a (multivariate) meta-analysis to estimate an overall effect across AWB dimensions and compared standardized effect sizes derived from multilevel within- and between-person correlations *r* and their respective 99.2% CIs of different AWB dimensions (two-stage IPD). Second, we built a series of three-level models to investigate the role of individual participant and measurement characteristics including standardized PA values and distinct AWB dimensions, controlling for age and sex/gender (one-stage IPD). Third, to calculate practical effect sizes, we built an additional series of multilevel models with the same structure (step 2) but restricted the data to studies containing measures of raw PA data (MAI, in milli-*g*) and added PA aggregation interval as a control variable (MAI one-stage IPD). The decision to employ MAI for the deduction of practical effect sizes (instead of, for example, counts or MVPA) was data-driven: we compared PA data distributions and statistics across studies and decided on the most homogeneous PA measure. Finally, we explored variations in effects based on study-level, participant-level and measurement-level characteristics and conducted moderator analyses. A detailed description of all analysis steps can be found in Supplementary Information Sections 2, 3, 11 and 12.

### Risk of bias

To assess and account for risk of bias between studies, we calculated small study bias in the two-stage IPD analysis and visually inspected funnel plots. We used the precision-effect test and precision-effect estimate with standard errors to apply a small study bias correction by using either the standard error and the effect size variance as regressors^158^. Influential studies were quantified using Cook’s *D*. A threshold of *D* > 0.5 was used to qualify a study as influential^159^. Heterogeneity was assessed using Cochran’s *Q*, which indicates if the extracted effect sizes estimate a common population effect size.

To assess risk of bias in individual studies, we applied the ROBINS-E tool^160^. Ratings were conducted and discussed by three researchers (I.T., J.R. and M.G.) and are presented in Supplementary Information Section 16. Further, we also employed the modified quality assessment (QA) tool developed by Timm et al.^34^ as a QA tool specifically developed for ambulatory assessment studies on PA and AWB. The modified QA tool includes categories such as accelerometer technology employed, e-diary sampling schema applied and compliance rates received. It consists of 16 questions (that is, the total score equals 16). For the current meta-analysis, all included records’ quality was assessed independently by two researchers (I.T. and M.G.) and divergent ratings were rated by a third author (M.R.). Ratings can be found in Supplementary Information Section 17.

### Additional analyses

In the two-stage IPD analyses, sensitivity analyses were carried out with values of *ρ* = 0, *ρ* = 0.25, *ρ* = 0.75 and *ρ* = 1.0 (Supplementary Information Section 18).

To ensure robustness of findings against violated assumptions in one-stage IPD analyses, we followed established procedures^161,162^ and calculated residual/wild bootstrapped CIs using the package lmeresampler^131^. In addition, as there is an increasing call for ordinal instead of metric models to analyse Likert-scaled data^163^, for the antecedent hypothesis, we also calculated ordinal multilevel regression models using the package ordinal^130^. In most cases, findings aligned (Supplementary Information Section 4).

Moreover, to ensure robustness of AWB operationalizations, we conducted sensitivity analyses in the one-stage IPD analysis. These analyses followed the same procedure as our main analysis but were restricted to a subset of studies that followed a narrowly defined assessment of AWB (Supplementary Information Sections 4.5, 4.6 and 9).

Finally, we set up a logistic regression model and predicted data provision by study characteristics to explore systematic differences between studies that provided data and studies that did not provide data for our meta-analysis (Supplementary Information Section 19). In the studies included, PA was more often investigated before AWB than in studies that did not provide data (*P* = 0.01).

### Deviations from preregistration

In contrast to preregistration, the synthesized data did not allow to investigate the preregistered research questions RQ6 (does the context of physical behaviour influence the effect size, for example, gym versus home?) and RQ8 (how long does the effect on well-being last?) as the information was missing in individual datasets. Further, RQ4 (does the type of physical behaviour influence the effect size?) and RQ5 (does the duration of physical behaviour influence the effect size?) could only be approximated by two-stage IPD moderator analyses (that is, moderation of PA type and aggregation interval; Supplementary Information Section 11). In addition, our meta-analysis is based on peer-reviewed records identified by Timm et al.^34^ and also includes records identified by an updated literature search and unpublished/unrequested data. Data harmonization and merging was not conducted by two researchers separately but by J.R. using a standardized R script. The R script underwent independent code review by J.P. As effect measures in two-stage IPD analyses, we employed multilevel correlations instead of standardized beta coefficients as correlations allow for easier interpretations and are comparable to beta coefficients in case of no included covariates. Standardized betas were used in one-stage IPD analyses. We forwent imputations of missing data due to the lack of consistent predictors of missing data across individual datasets. Finally, influential studies in two-stage IPD analysis were identified using Cook’s *D* instead of bootstrapping to follow established procedures.

### Reporting summary

Further information on research design is available in the Nature Portfolio Reporting Summary linked to this article.

## Supplementary information

Supplementary Information Supplementary Information Sections 1–19, which contain the respective Supplementary Tables and Figures (for example, Supplementary Information Section 4 contains Supplementary Table 4.1a–Supplementary Table 4.6e; Supplementary Information Section 6 contains Supplementary Fig. 6).

Reporting Summary

Peer Review File

## Data Availability

For licence and ethical reasons, IPD cannot be made publicly available. Data from the individual studies need to be requested from the respective data contributor. To facilitate data requests, a list of included datasets is publicly available (https://osf.io/2tn8u). Furthermore, we are happy to assist in establishing contact with the data contributors.

## Code availability

The analysis code of our analyses is publicly available (https://osf.io/2tn8u).
