# Supplementary material for: An individual participant data meta-analysis of how physical activity relates to affective well-being in daily life
Source: Nat Hum Behav. 2026 May 6;10(7):1297–315. doi: 10.1038/s41562-026-02427-2 (PMC13388107; doi:10.1038/s41562-026-02427-2)
Supplement: Supplementary file 1 — Supplementary Information Sections 1–19, which contain the respective Supplementary Tables and Figures (for example, Supplementary Information Section 4 contains Supplementary Table 4.1a–Supplementary Table 4.6e; Supplementary Information Section 6 contains Supplementary Fig. 6). [file 41562_2026_2427_MOESM1_ESM.pdf]

nature human behaviour

Supplementary information

https://doi.org/10.1038/s41562-026-02427-2

An individual participant data meta-analysis of how physical activity relates to affective well-being in daily life

In the format provided by the authors and unedited

Table of Contents

S1: Data Extraction Table and Preprocessing

S2: Two-stage Individual Participant Data meta-analysis

S3: One-stage (Movement Acceleration Intensity) Individual Participant Data meta-analysis

S4: Sensitivity and Robustness Analyses of one-stage Individual Participant Data models

S5: Results of antecedent one-stage Individual Participant Data models

S6: Results of antecedent one-stage Movement Acceleration Intensity Individual Participant Data models

S7: Results of consequent one-stage Individual Participant Data models

S8: Results of consequent one-stage Movement Acceleration Intensity Individual Participant Data models

S9: Assessing affective well-being in daily life

S10: Two-stage Individual Participant Data meta-analysis of antecedent vs. consequent model

S11: Results of moderation analysis in two-stage Individual Participant Data models

S12: Results of moderation analysis in one-stage Individual Participant Data models

S13: Low Base effects in one-stage Individual Participant Data models

S14: PRISMA Checklist

S15: Comprehensive search term

S16: Risk of Bias assessment of individual studies using ROBINS-E

S17: Quality Assessment of individual studies

S18: Sensitivity analyses of two-stage Individual Participant Data models

S19: Predicting data provision by study characteristics

S1: Data Extraction Table and Preprocessing

To extract variables on study level, a data extraction template was custom-developed to summarise data on: authors, country, detailed participant characteristics, AWB assessment, PA assessment, assessment duration (number of days participants were instructed to wear accelerometers and fill out e-diaries), and assessment frequency (number of e-diary prompts sent per day). The independent extraction files of two researchers (IT, MG) were merged. If discrepancies could not be resolved, a third reviewer (MR) was consulted to reach a decision.

For participant and measurement characteristics, all contributed data files were inspected individually by JR. Relevant variables were renamed and recoded if necessary in order to harmonise all data sets following a predefined data mask. The data mask comprised the following variables. From the data extraction template: authors, country, participants characteristics (short sample description, e.g., "depressed adults", "healthy children"), PA assessment (placement of accelerometer, measure of PA), AWB assessment (AWB concept), assessment duration, assessment frequency. From individual datasets: gender/sex, age, BMI, date, time, study day number, prompt number per day, prompt number across days, weekday, PA aggregation interval, PA measure preceding prompt, PA measure subsequent to prompt, and AWB concept scores. If no AWB scale mean was provided, scale means were calculated from item scores. All AWB scores were recoded to the scale of 1 to 4 (the smallest available scale in contributed data sets). If PA scores were only available centered around the EMA prompt, the centered score was included as both PA preceding and PA following the EMA prompt. For daily PA aggregates (1,440 minutes), if the e-diary was filled out at the beginning of the day PA was included as PA following the EMA prompt, and if the e-diary was filled out at the end of the day, PA was included as PA preceding the EMA prompt. PA scores were rescaled to minute-level (i.e., sum scores were divided by the aggregation interval) for one-stage MAI IPD analyses and standardised by dividing by the standard deviation of the respective study in order to control for different metrics and accelerometer placements for the one-stage IPD analyses. Missing design variables (e.g., indicators for day, beep, observation) were added based on time and date. Weekday vs. weekend was also inferred from date.

Following recent and established guidelines for ESM data[1], all data sets were individually and jointly checked for inconsistencies, such as missing/duplicated design variables, time-variant demographic variables, and implausible descriptive statistics/out-of-range values. Inconsistencies were discussed with the respective data contributor; if no explanation or solution (in case of technical errors) was found, data were set to missing, resulting in i = 206 additional missing rows. Implausible PA values (i.e., MET < 1 or MVPA minutes exceeding the PA aggregation interval) were set to missing, resulting in i = 3,140 induced missing values. Further, we excluded all rows of data with neither PA nor AWB data, reducing the dataset from i = 380,349 to i = 369,828. We also excluded participants with a standard deviation of 0 in all AWB scores which we used as an indicator of careless responding to EMA prompts. This led to the exclusion of 27 participants (0.33%)/477 measurements (0.13%). These steps resulted in a final dataset containing 8,223 participants and 369,351 e-diary prompts with 321,345 e-diary ratings.

Publication	Country[a]	n[b]	days[b]	prompts[b]	Female[b] (%)	Age[b] (mean, range)	Participants characteristics[a] sample; (study name)	Affect assessment[a, b] AWB items → assigned to (questionnaire; software, device); prompts/day	Physical behaviour assessment[a, b] (unit; time frame; direction; device; placement)	Assessment duration; sampling design[a, b] (random, fixed, event-based, mixed)
Berli et al. (2021) [2]	CH	122	37.88	4,621	50.00%	44.23 (22-72)	Adults (inactive, overweight couples, DYACTIC); only control groups in analysis	Positive affect, negative affect (Positive and Negative Affect Schedule, PANAS, Thompson, 2007[3]; NR); 1 prompt/day (end of the day)	PA (counts); 1440 min; before; ActiGraph GT3X+; hip	28 (T1) + 14 (T3) days, fixed
Bermudez et al. (2021) [4]	CH	127	19.63	2,493	16.54%	61.92 (24-83)	Adults (cardiac disease patients, CAMP)	Positive affect, negative affect; (PANAS, no reference; NR, Tablet); 1 prompt/day (end of the day)	PA (LPA); 1440 min; before; ActiGraph GT3X; hip	21 days, fixed
Bossmann et al. (2013) [5]	GER	62	1.60	807	14.52%	21.40 (19-30)	Adults (students)	Valence, energetic arousal, calmness (MDMQ, Wilhelm & Schoebi, 2007[6]; MyExperience, study smartphone); Every hour after waking up (approx. 13 prompts/day)	PA (MAI milli-*g*); 10 min; before; Movisens Move I; chest	1 day, fixed
Bourke et al. (2021) [7]<br>Bourke et al. (2022) [8]<br>Bourke et al. (2023) [9]	AU	119	3.87	2,016	46.22%	14.65 (13-17)	Adolescents	Valence, energetic arousal, tense arousal/calmness (MDMQ, Wilhelm & Schoebi, 2007[6]; Qualtrics, participants' smartphone); 5 (weekdays) or 9 (weekend days) prompts/day	PA (LPA); 60 min; before and after; ActiGraph GT3X+; dominant wrist	4 days, fixed with random component
Cabrita et al. (2017) [10]	NL	10	32.00	2,301	60.00%	68.70 (65-83)	Elders	Pleasure → positive affective states (no reference; Activity Coach, study smartphone); 12 prompts/day	PA (IMA); 10 min; centered → before and after (Activity Coach; hip)	30 days, fixed
Cook et al. (2022) [11]	US	54	25.63	1,384	16.67%	45.78 (26-69)	Adults (HIV positive)	DABS mood → positive affective states, negative affective states (DABS Mood, Kamarck et al., 1998[12]; REDCap, participants' smartphone); 1 prompt/day	PA (steps); 1440 min; random time → before and after; Fitbit Alta HR; wrist	30 days, random
Curtiss et al. (2022) [13]	US	34	13.12	1,567	73.53%; 2.94% non-binary	28.97 (18-55)	Adults (MDD, anxiety)	Positive affect, negative affect (Positive and Negative Affect Items, Leemput et al., 2014[14]; Ethica App, participants' smartphone); 5 prompts/day	PA (counts); 60 min; before; Ethica App smartphone; on person	14 days, fixed
Dickman et al. (2020) [15]	US	491	4.25	27,778	52.34%	42.78 (30-54)	Adults (AHAB-II)	Positive affect, negative affect (no reference; Palm Z22); hourly during waking hours (approx. 14 per day)	PA (MET); 10 min; before; SenseWear Pro3; wrist	4 days, fixed
Dunton et al. (2011) [16]<br>Dunton et al. (2014) [19]	US	120	3.74	1,714	48.33%	11.02 (9-13)	Children (Healthy PLACES)	Positive affect, negative affect (PANAS for Children, Ebesutani et al., 2012[17], Laurent et al., 1999[18]; MyExperience, study smartphone); 3 (weekdays) -7 (weekend days) prompts/day	PA (counts); 15 min; before and after; ActiGraph GT2M; right hip	8 days (2 waves a 4 days), fixed with random component

Elavsky et al. (2016) [20]	US	117	13.75	5,084	100.00%	51.68 (40-60)	Adults	Positive affect, negative affect (PANAS, Thompson, 2007[3]; Purdue Momentary Assessment Tool, PDA); 4 prompts/day	PA (counts); 180 min; before; ActiGraph GT1M; nondominant hip	15 days, mixed
Elavsky et al. (2021) [21]	CZ	30	22.83	1,931	46.67%	61.20 (50-74)	Adults, Elders	Positive affect, negative affect (no reference; Survey app developed for the study, Android smartphones); 3 prompts/day	PA (steps); 15 min; before and after; Fitbit Charge 2; wrist	24 days, 3 waves á 8 days, mixed
Elavsky et al. (2024) [22]	CZ	1,310	13.88	71,253	46.41%	38.09 (18-65)	Adults (runners and inactive controls; 4HAIE)	Positive affect, negative affect; (no reference; Survey app developed for the study; participants' smartphones); 4 prompts/day	PA (LPA); 15 min; before and after; Fitbit Charge 3; wrist	14 days, fixed with random component
Gallagher & Carr (2021) [23]	US	113	3.93	1,528	68.14%	39.35 (21-57)	Adults (full-time employed)	Mood → Valence (Brief Mood Introspection Scale, Mayer & Gaschke, 1988[24]; Boomerang, NR); 4 prompts/day	PA (steps); 60 min; before and after; ActivPAL3: front mid-thigh	4 days, fixed
Giurgiu et al. (2019) [25]	AU; GER	92	4.83	3,722	65.22%	33.73 (22-62)	Adults (university employee)	Valence, energetic arousal, calmness (MDMQ, Wilhelm & Schoebi, 2007[6]; movisensXS, study smartphone); 8-21 prompts/day	PA (MAI milli-*g*); 15 min; before and after; Movisens Move 3 + EcgMove 3; chest, hip, thigh	5 days, mixed
Giurgiu et al. (2020) [26]										
Giurgiu et al. (2020) [27]										
Giurgiu et al. (2022) [28]	GER	110	4.95	3,828	54.55%	23.28 (17-57)	Adults (students)	Valence, energetic arousal, calmness (MDMQ, Wilhelm & Schoebi, 2007[6]; movisensXS, study smartphone); 6 prompts/day	PA (MAI milli-*g*); 15 min; before and after; Movisens Move 4; wrist, hip, thigh	5 days, mixed
Giurgiu et al. (2023) [29]										
Timm et al. (2023) [30]										
von Haaren et al. (2013) [31]	GER	59	2.00	938	0.00%	21.42 (19-27)	Adults (students)	Valence, energetic arousal, calmness (MDMQ, Wilhelm & Schoebi, 2007[6]; My Experience, PDA); 8 prompts/day	PA (MAI milli-*g*); 15 min; before and after; Movisens Move II; chest	2 days, fixed with random component
Hachenberger et al. (2023) [32]	GER	50	9.62	844	78.00%; 2.00% non-binary	23.61 (19-34)	Adults (students), exam-period	Positive affect, negative affect (items following Das-Friebel et al., 2020[33]; movisensXS, study or participants' smartphone); 2 prompts/day	PA (ENMO milli-*g*); 90 min; before; GENEActiv; (non-dominant) wrist	10 days, fixed
Haucke et al. (2022) [34]	GER	260	7.00	12,031	67.69%; 1.15% non-binary	30.83 (18-72)	Adults, lonely during COVID pandemic	Stress, lonelieness → negative affective states (no reference; movisensXS; participants' smartphone); 8 prompts/day	PA (ENMO milli-*g*); 15 min; before and after; GENEActive; (left) wrist	7 days, fixed with a random component
Hevel et al. (2021) [35]	US	103	10.00	5,924	62.14%	72.25 (60-98)	Adults, Elders	Positive affect, negative affect (no reference; movisensXS, study smartphone); 6 prompts/day	PA (Time Stepping); 15 min; before and after; ActivPAL; thigh	10 days, fixed with random component
Hollands et al. (2020) [36]	UK	42	4.90	381	83.33%	37.62 (19-63)	Adults with MDD (eMotion)	Mood → valence (no reference; text local, participant's smartphones); 2 prompts/day	PA (LPA); 60 min; before; GENEActive; (non-dominant) wrist	5 days, fixed
Jeckel & Sudeck (2016) [37]	GER	46	6.26	489	60.87%	31.11 (20-59)	Adults	Valence, energetic arousal, calmness (MDMQ, Wilhelm & Schoebi, 2007[6]; MyExperience, study smartphone); 2 prompts/day	PA (MET); 15 min; before; Movisens EcgMove; chest	7 days, mixed

Jeckel & Sudeck (2018) [38]										
Kanning (2009)	GER	93	1.60	733	41.94%	37.82 (20-74)	Adults	Valence, energetic arousal, calmness (MDMQ, Wilhelm & Schoebi, 2007[6]; NR); up to 13 prompts/day, median 5 prompts/day	PA (MAI milli-*g*); 10 min.; before; Becker Meditech Varioport-e; hip	1 day, mixed
Kanning (2010)	GER	54	1.46	320	35.19%	58.02 (50-84)	Adults, Elders	Valence, energetic arousal, calmness (MDMQ, Wilhelm & Schoebi, 2007[6]; NR); up to 10 prompts/day, median 4 prompts/day	PA (MAI milli-*g*); 10 min.; before; Becker Meditech Varioport-e; hip	1 day, mixed
Kanning et al. (2012)* [39]	GER	98	1.91	1,811	55.10%	27.14 (18-68)	Adults (students)	Valence, energetic arousal, calmness (MDMQ, Wilhelm & Schoebi, 2007[6]; Izybuilder, PDA); 19 prompts/day	PA (MAI milli-*g*); 10 min; before; Becker Meditech Varioport-e; hip	1 day, fixed with random component
Kanning (2013)* [40]										
Kanning & Schoebi (2016)* [41]										
Kanning et al. (2015)* [42]	GER	69	2.77	1,331	49.28%	60.17 (49-74)	Adults, Elders	Valence, energetic arousal, calmness (MDMQ, Wilhelm & Schoebi, 2007[6]; MyExperience, study smartphone); activity-triggered prompts; up to 17 prompts/day, median 6-7 prompts/day	PA (MAI milli-*g*); 10 min; before; Becker Meditech Varioport-e; hip	3 days, mixed
Kanning & Hansen, (2017)* [43]										
Kim et al. (2013) [44]	JP	31	2.68	541	19.35%	21.58 (20-32)	Adults, undergraduates	Positive mood, negative mood → positive affective states, negative affective states (DAMS, Fukui, 1997[45]; NR, wristwatch); 10 prompts/day	PA (counts); 60 min; centered → before and after; wristwatch computer (ECOLOG, Ruputer); (nondominant) wrist	2 days, mixed
		30	10.17	1,319	86.67%	13.57 (13-14)	Adolescents	Positive mood, negative mood → positive affective states, negative affective states (DAMS, Fukui, 1997[45]; NR, wristwatch); 5 prompts/day	PA (counts); 60 min; centered → before and after; wristwatch computer (ECOLOG, Ruputer); (nondominant) wrist	7 days, mixed
		24	7.92	976	0%	41.04 (24-58)	Adults, office workers	Positive mood, negative mood → positive affective states, negative affective states (DAMS, Fukui, 1997[45]; NR, wristwatch); 6 prompts/day	PA (counts); 60 min; centered → before and after; wristwatch computer (ECOLOG, Ruputer); (nondominant) wrist	7 days, mixed
Kim et al. (2020) [46]	US	110	2.95	1,868	76.36%	41.30 (19-63)	Adults, full-time employed (Work and Daily Life)	Valence, energetic arousal (no reference; NR, PDA); 6 prompts/day	PA (counts); 5min; before and after; ActiHeart; chest	3 days, fixed with random component
Kim et al. (2021) [47]	JP	35	13.17	1,866	40.00%	21.69 (19-26)	Adults (students)	Positive affect, negative affect (no reference; smartphone); 6 prompts/day	PA (counts); 10 min; before and after; ActiGraph wGT3X; wrist	14 days, fixed with random component

Koch et al. (2018) [48]<br>Koch et al. (2020) [50]	GER	198	6.88	7,808	43.43%	15.02 (11-18)	Adolescents (URGENY)	Valence, energetic arousal, calmness, positive affect, negative affect (items following Leonhardt et al., 2016[49]; MovisensXS, study smartphone); 4-7 (weekdays) or 8-17 (weekend days) prompts/day	PA (MAI milli-*g*), 15 min; before and after; Movisens Move 2 + Move 3; (right) hip	7 days, mixed
Koch et al. (2022) [51]	GER; NL; ESP; UK	191	3.96	8,887	52.88%	24.72 (14-44)	Adolescents, adults (with and without ADHD)	Positive and negative affect (items following Myin-Germeys et al., 2003[52]; movisens XS, NR); 12 prompts/day	PA (MAI milli-*g*); 10 min; before; Movisens LightMove 3; (non-dominant) wrist	4 days, random
Kracht et al. (2021) [53]	US	315	6.72	6,660	53.02%	12.61 (10-16)	Adolescents (TIGER Kids study)	Positive affect, negative affect (PANAS for Children, Ebesutani et al., 2011[54]; LifeData Corporation App, study device, or participants' smartphone); 2 (weekdays) or 6 (weekend days) prompts/day	PA (counts); 10 min; before and after; ActiGraph GT3X+; hip	7 days, fixed with random component
Kuehnhausen et al. (2013) [55]	GER	58	17.74	1,029	43.10%	9.19 (8-11)	Children (FLUX)	Pleasantness, unpleasantness → positive affective states, negative affective states (items following Leonhardt et al., 2016[49]; NR, study smartphone); 4 prompts/day	PA (MVPA); 1440 min; daily average → before and after; ActiGraph GT3X+; hip	28 days, fixed
Leger et al. (2023) [56]	US	307	5.31	6,165	55.05%	74.00 (65-90)	Elders (Daily Experiences and Well-being Study)	Positive affect, negative affect (items following Fingerman et al., 2016[57]; NR, Android smartphone); 4 prompts/day	PA (LPA); 180 min; before; Phillips Respironics Actical Z; wrist	6 days, fixed
Li et al. (2022) [58]	UK	78	14.05	4,168	73.08%	25.46 (17-43)	Adults	Positive affect, negative affect, (items taken from PHQ-9[59], the Zung Self-Rating Depression Scale[60], the Hospital Anxiety and Depression Scale[61], and the Depression Anxiety Stress Scales[62]; movisensXS or Qumi, participants' smartphone); 5 prompts/day	PA (MAI milli-*g*); 31 min; before and after; Movisens EcgMove 3; chest	14 days, fixed with random component
Liao et al. (2017) [63]<br>Liao et al. (2017) [64]<br>Maher et al. (2017) [65]	US	116	10.37	7,651	71.55%	40.37 (27-73)	Adults, low-active (MOBILE)	Positive affect, negative affect, (no reference; MyExperience, study smartphone); 8 prompts/day	PA (counts); 15 min; before and after; ActiGraph GT2 M; hip	4 days, fixed with random component
Madden et al. (2020) [66]	US	20	3.85	520	75.00%	47.30 (21-63)	Adults (severe mental illness: MDD, bipolar, schizophrenia)	Positive affect, negative affect (no reference; custom software phone application, study smartphone); 7 prompts/day	PA (counts); 15 min.; before and after; Actigraph wGT3x-BT; (right) hip	4 days, fixed with random component
Maher (2022)	US	90	8.00	4,146	77.78%	70.20 (60-89)	Adults, Elders (racially minoritised)	Positive affect, negative affect (no reference; movisensXS, study smartphone); 6 prompts/day	PA (Time Stepping); 15 min; before and after; ActivePAL; thigh	8 days, fixed with random component
McCormick et al. (2008) [67]	SRB/US	20	7.75	933	55.00%	39.75 (19-59)	Adults (with severe mental illness)	Positive affect, negative affect (no reference; Casio Data Bank 50 telememo watch); 7 prompts/day	PA (counts); 10 min; before; MTI 7164; (right) hip	7 days, random

Michalak et al. (2022) [68]	GER	71	2.00	1,816	60.56%	39.31 (18-60)	Adults (MDD, healthy)	Mood → valence (no reference; Palm Tungsten T3, NR); 14 prompts/day	PA (MAI milli-*g*); 60 min; before; Vitamove; trunk, (right) thigh	2 days, fixed
Olfermann et al. (2024) [69]	GER	66	6.94	6,662	100.00%	25.39 (19-57)	Adults, adolescents (with eating disorder, healthy)	Valence, energetic arousal, calmness (MDMQ, Wilhelm & Schoebi, 2007[6]; movisensXS, study smartphones); 9-22 prompts/day; average 13.15 prompts/day	PA (MAI in milli-*g*); 15 min; before and after; Movisens Move 3; hip	7 days, mixed
Pannicke et al. (2020) [70]	AT	37	6.97	1,506	75.68%	23.51 (19-28)	Adults	Positive affect, negative affect (German Version of the PANAS, Breyer & Bluemke, 2016[71], and additional items without reference; NR, participants' smartphone); 6 prompts/day	PA (MET); 150 min; before; Actiheart; chest	7 days, fixed
Pham et al. (2023) [72]	US	196	13.69	13,409	54.59%	39.55 (18-75)	Adults (type I diabetes)	Positive affect, negative affect, (items following Broderick et al., 2009[73], Crawford & Henry, 2004[74], Dunton et al., 2008[75], Laurenceau, 2013[76], Merwin et al., 2015[77], Scott et al., 2017[78]; Ilumivu, study smartphone); 5-6 prompts/day	PA (steps); 180 min; before; ActiGraph wGT3X-BT; (non-dominant) wrist	14 days, fixed
Poppe et al. (2021) [79]	BE	51	9.63	491	39.22%	62.73 (46-81)	Adults (with type 2 diabetes mellitus)	Stress, sadness, fatigue → negative affective states (no reference; LimeSurvey, participants' computer or tablet); 1 prompt/day	PA (LPA); 1440 min; after; ActiGraph GT3X+; (right) hip	10 days, event
Reichert et al. (2016) [80]<br>Reichert at al. (2017) [81]	GER	351	6.91	27,490	56.13%	23.05 (18-28)	Adults (URGENCY)	Valence, energetic arousal, calmness, positive affect, negative affect (MDMQ, Wilhelm & Schoebi, 2007[6], no reference for positive and negative affect items; movisensXS, study smartphone); 9-22 prompts/day	PA (MAI milli-*g*); 15 min; before and after; Movisens Move 2; hip	7 days, mixed
Reichert et al. (2018) [82]	GER	62	2.95	1,836	41.94%	25.79 (18-55)	Adults, depressed and healthy students/employees	Valence, energetic arousal, calmness (MDMQ, Wilhelm & Schoebi, 2007[6]; movisensXS, study smartphone); approx. 10 prompts/day	PA (MAI milli-*g*); 15 min; before and after; Movisens Move 3; hip	3 days, mixed
Reininghaus et al. (2023) [83]	GER	76	8.45	4,601	75.00%	21.63 (14-25)	Adolescents, Adults (distressed; at risk of first episode of a severe mental disorder)	Positive affect, negative affect (no reference; movisensXS, study smartphone); up to 10 prompts/day	PA (MAI milli-*g*); 15 min; before and after; Movisens EcgMove 4; chest	7 days, fixed with random component

Ruissen et al. (2022) [84]	USA	126	14.28	8,330	48.41%	27.71 (18-40)	Adults	Positive affect, negative affect (items selected from PANAS(-X), Watson & Tellegan, 1988[85], Watson & Clark, 1999[86] following Sperry et al., 2021[87]; MetricWire, participants' smartphone); 6 prompts/day	PA (MVPA); 60 min; before and after; Fitbit Blaze; wrist	14 days, fixed with random component
Schwerdtfeger & Mai, (2009) [88]<br>Schwerdtfeger et al. (2015) [89]	GER; AT	60	1.17	958	51.67%	29.15 (20-40)	Adults	Positive affect, negative affect (no reference; Palm Zire3, PDA); 15 prompts/day	PA (root integral milli-*g*); 5 min; before; Varioport-b, uni- and triaxial accelerometer Becker Meditech; thigh and chest	1 day, fixed with random component
Schwerdtfeger et al. (2010) [90]	GER	124	1.00	1,447	51.61%	31.67 (18-73)	Adults	Positive affect, negative affect (no reference; DialogPad, PDA); 13 prompts/day	PA (counts); 5 min; before and after; ActiGraph GT1M; ankle	1 day, fixed with random component
Schwerdtfeger & Scheel (2012) [91]	GER; AT	84	1.25	1,275	59.52%	26.36 (20-39)	Adults	Positive affect, negative affect (no reference; Palm Zire31PDA); approx. 15 prompts/day	PA (root integral milli-*g*); 5 min; before; Varioport-b, Becker Meditec; thigh and chest	1 day, fixed with random component
Schwerdtfeger & Gerteis (2014) [92]	AT	122	2.97	4,030	55.74%	27.80 (20-40)	Adults, natural interaction dyads	Positive affect, negative affect (German version of the PANAS, Krohne et al., 1996[93]; iPod Touch); approx. 13 prompts/day	PA (milli-*g*); 5 min; before; Hidalgo Equivital EQ-Q1 LifeMonitor; chest	3 days, fixed with random component
Schwerdtfeger & Rathner (2016) [94]	AT	114	1.97	2,814	47.37%	37.30 (30-60)	Adults	Positive affect, negative affect (no reference; iDialogPad App, iPod Touch); approx. 25 prompts/day	PA (root integral milli-*g*); 5 min; before; Varioport-b, Becker Meditec; thigh and chest	1 day, fixed with random component
Schwerdtfeger & Dick (2019) [95]	GER	38	1.97	610	0.00%	32.71 (25-53)	Adults, firefighters	Negative affect, resilience → positive affective states, (Adapted items from resilience scale, RS-25, Wagnild & Young, 1993[96], and PANAS, Watson et al., 1988[85]; movisensXS); 16 prompts/day	PA (energy expenditure); 6 min; before; Movisens EcgMove 3; chest	1 day, fixed with random component
Seiferth et al. (2023) [97]	CH	157	6.79	4,807	68.15%	49.60 (21-74)	Adults, obese	Valence, energetic arousal, calmness (MDMQ, Wilhelm & Schoebi, 2007[6]; I-GENDO App); 8 prompts/day	PA (counts); 15 min.; before and after; ActiGraph wGT3X-BT; hip	7 days, mixed
Smith et al. (2020) [98]	US	16	13.81	221	56.25%	11.28 (8-14)	Children (with overweight/obesity)	Positive affect, negative affect (PANAS, Watson et al., 1999[99]; ReTAINE, smartphone NR); 4-6 prompts/day and after meals	PA (MVPA); 1020 min; daily average → before and after; Philips Actiwatch 2; wrist	14 days, mixed
Stevenson et al. (2022) [100]	US	25	18.56	1,475	56.00%	39.76 (22-60)	Adults (alcohol use disorder)	Positive affect, negative affect (PANAS-X, Watson et al., 1988[85]; ilumivu, participants' smartphone); 4 prompts/day	PA (steps); 60 min; before; Fitbit Charge 3; wrist	21 days, fixed with random component

Sudeck et al. (2018) [101]	GER	64	3.94	780	57.81%	35.47 (20-63)	Adults	Valence, energetic arousal, calmness (MDMQ, Wilhelm & Schoebi, 2007[6]; movisensXS, study smartphone); 4 prompts/day	PA (MAI milli-*g*); 15 min; before; Movisens Move 3; hip	4 days, fixed with random component
Takano et al. (2013) [102]	JP	47	7.00	2,571	25.53%	19.38 (18-23)	Adults (undergraduate students)	Positive affect, negative affect (items from PANAS, Watson, et al., 1988[85]; NR, participants' smartphone); 8 prompts/day	PA (counts); 15 min; before; Respironics Actiwatch; wrist	7 days, fixed with random component
Vetrovsky et al. (2021) [103]	CZ	27	19.78	534	74.07%	68.22 (60-91)	Adults, Elders	Fatigue → negative affective states (single-item from Brief Fatigue Inventory, Mendoza et al., 1999[104]; NR, participants' smartphone); 1 prompt/day	PA (ENMO milli-*g*); 720 min; centered → before and after; ActiGraph wGT3X-BT; (right) hip	21 days, fixed
Williams et al. (2020) [105]	US	194	89.53	17,368	71.13%	40.72 (20-74)	Adults	Positive affect, negative affect (PANAS, Watson et al., 1988[85]; online survey, NR); 1 prompt/day	PA (steps); 1440 min; before; Fitbit Charge; wrist	100 days, fixed
Yang et al. (2020) [106]<br>Wen et al. (2018) [107]<br>Dunton et al. (2022) [108]<br>Kanning et al. (2020) [109]	US	395	30.63	39,997	75.44%	25.19 (9-56)	Children, adults (MATCH)	Positive affect, negative affect (PANAS, child and parent shortened versions, Ebesutani et al., 2021[17], Laurent et al., 1999[18]; NR, participants' or study smartphone); mothers: 4 (weekdays) or 8 (weekend days) prompts/day; children: 3 (weekdays) or 7 (weekend days) prompts/day	PA (counts); 15 min; before and after; ActiGraph GT3X or WGT3X-BT; (right) hip	42 days, fixed with random component
Zarbo et al. (2023) [110]	IT	167	6.76	6,766	41.92%	40.75 (18-56)	Adults, schizophrenia and controls (DiAPAson)	Positive affect, negative affect (Experience Sampling Method Item Repository, https://osf.io/kg376/; custom-developed App, smartphone); 8 prompts/day	PA (counts); 15 min; before and after; Actigraph GT9X; wrist	7 days, fixed with random component
Zhaoyang & Martire (2019) [111]	US	145	21.65	6,191	57.24%	65.70 (50-95)	Elders (knee osteoarthritis)	Positive affect, negative affect (items following Thomas & Diener, 1990[112]; NR, hand held computer); 1 prompts/day	PA (LPA); 1440 min; before and after; ActiGraph GT1M or GT3X; hip	22 days, fixed

Note. [*]for a better overview, two datasets were summarised in one row of the data extraction table as multiple records relied on these two datasets; this affects the total number of datasets in the data extraction table which is accordingly 65 instead of 67 [a] Information extracted from publication, [b] Information derived from dataset; Abbreviations: After = affective well-being prior to physical activity; Before = physical activity prior to affective well-being; Centered = physical activity centered around affective well-being; ENMO = Euclidean Norm Minus One; Event = self-initiated prompts; Fixed with random component = random prompts within pre-established intervals or semi-random prompts; Fixed = fixed prompts; IMA = Integral Module of Acceleration; LPA = light physical activity; MAI = Movement Acceleration Intensity; MDD = major depression disorder; min = minutes; Mixed = e.g., activity- or sedentary-triggered prompt, combined with random prompts; MVPA = moderate to vigorous physical activity; NR = not reported; PDA = personal digital assistant; Random = random prompt

S2: Two-stage Individual Participant Data meta-analysis

For the two-stage IPD multivariate multilevel approach, correlation coefficients and their variance (squared standard error) were extracted from the standardised raw data of each original study both on the within- and between-person level using lavaan[113]. We used maximum likelihood estimation with robust (Huber-White) standard errors and the nlminb optimiser when estimating multilevel correlations (data was clustered in participants) using the sem-function. Two studies needed to be excluded from two-stage IPD analysis due to sampling errors. We then used the rma.mv function from the metafor package[114] to calculate an overall effect across all studies and outcomes. As less negative affective states is indicative of increased AWB, this concept was inverted for the overall analysis. To account for the hierarchical structure of the data, we used a multilevel structure with random effects at the study and effects level. Furthermore, we calculated the variance-covariance matrix that provides a better estimation of effect size variance when multiple effect sizes come from the same study. The variance-covariance matrix was calculated by default with an assumed correlation of effect sizes within each cohort of rho = 0.5. In addition to these procedures, we used robust-variance estimation with cluster-robust inference at the study level. This step is recommended to more accurately determine the confidence intervals in complex multivariate models[115].

In the next step, we calculated a model with the factors outcome (valence, calmness, energetic arousal, positive affective states, negative affective states) and association level (within- or between-person associations) as predictors of the obtained effect sizes. This model was computed separately for the antecedent and consequent effects. Data of two-stage IPD meta-analysis are reported via correlation coefficients and their respective 99.2% confidence intervals (CI). Data were visualized using orchard plots[116] as well as forest plots.

Post hoc tests comparing AWB outcomes with each other for the antecedent model revealed significant differences at the within-level between energetic arousal and valence ($r\Delta = 0.09$, $t_{(55)} = 5.46$, $p < .001$), positive affective states ($r\Delta = 0.07$, $t_{(55)} = 3.02$, $p = .004$), negative affective states ($r\Delta = 0.15$, $t_{(55)} = 6.64$, $p < .001$) as well as calmness ($r\Delta = 0.20$, $t_{(55)} = 7.81$, $p < .001$). Post hoc tests comparing AWB outcomes with each other for the consequent model revealed significant differences at the within-level between energetic arousal and valence ($r\Delta = 0.09$, $t_{(27)} = 5.90$, $p < .001$), positive affective states ($r\Delta = 0.07$, $t_{(27)} = 3.64$, $p = .001$), negative affective states ($r\Delta = 0.13$, $t_{(27)} = 7.89$, $p < .001$) as well as calmness ($r\Delta = 0.18$, $t_{(27)} = 9.81$, $p < .001$).

S3: One-stage (Movement Acceleration Intensity) Individual Participant Data meta-analysis

We calculated two separate one-stage IPD models comprising different amounts of datasets: The first included all available datasets, i.e. 67 datasets, irrespective of their diversity in PA metrics[117,118]. The second only contained datasets with raw accelerometry data, i.e. MAI (in milli-*g*; k = 19 datasets). Apart from the different number of datasets, analyses were conducted in parallel (however, aggregation interval of PA was added as control variable in the latter set of analyses), so that we waive on outlining the analyses separately.

To analyze within-person associations between PA and AWB, we set up three-level models for analyzing hierarchically structured intensive longitudinal data[119]. In particular, we calculated a random intercept random slope model with repeated measurements (level 1) nested within participants (level 2) nested within studies (level 3). Models were conducted in a bi-directional way with PA as an antecedent of AWB outcomes (i.e., positive affective states, negative affective states, valence, energetic arousal, and calmness; antecedent model), as well as PA as a consequence of AWB ratings (consequent model). In total, we conducted ten different multilevel models, which were replicated in the next analysis step with a restriction to studies containing raw PA data (MAI in milli-*g*). Intraclass correlations (ICCs) were estimated using unconditional models, including AWB (antecedent model) or PA (consequent model) as outcome. In all PA–AWB antecedent models, PA was person-mean-centered; both average and person-mean-centered PA were used as main predictors. In all PA–AWB consequent models, participants' average AWB and person-mean-centered AWB were used as main predictors. Models were controlled for age [years], gender/sex [male vs. female (vs. non-binary)], and, in one-stage MAI IPD models, the PA aggregation interval [min]. We included random effects for the intercept and the main predictor (person-mean-centered PA/AWB). The final models are presented in the equation below:

Antecedent model:
Level 1 equation:

$$AWB_{ijk} = \beta_{0jk} + \beta_{1jk} * meancenteredPA_{ijk} + \varepsilon_{ijk}$$

Level 2 equation:

$$\beta_{0jk} = \gamma_{00k} + \gamma_{01} * Age_{jk} + \gamma_{02} * Gender/Sex_{jk} + \gamma_{03} * personmeanPA_{jk} + u_{0jk}$$
$$\beta_{1jk} = \gamma_{10k} + u_{1jk}$$

Level 3 equation:

$$\gamma_{00k} = \delta_{000} + v_{00k}$$
$$\gamma_{10k} = \delta_{100} + v_{10k}$$

Consequent model:
Level 1 equation:

$$\sqrt{PA_{ijk}} = \beta_{0jk} + \beta_{1jk} * meancenteredAWB_{ijk} + \varepsilon_{ijk}$$

Level 2 equation:

$$\beta_{0jk} = \gamma_{00k} + \gamma_{01} * Age_{jk} + \gamma_{02} * Gender/Sex_{jk} + \gamma_{03} * personmeanAWB_{jk} + u_{0jk}$$
$$\beta_{1jk} = \gamma_{10k} + u_{1jk}$$

Level 3 equation:

$$\gamma_{00k} = \delta_{000} + v_{00k}$$
$$\gamma_{10k} = \delta_{100} + v_{10k}$$

To account for the known skewness of PA data, we square-root-transformed PA as outcome. We calculated post-hoc standardised beta coefficients (standardised β) following established procedures[120]; standardize_parameters function[121]). We used restricted maximum-likelihood (REML) estimation and the bobyqa optimiser for better convergence. However, variance on the study level emerged as very small, resulting for some models in singularity. In case of singularity, we switched to using a two-level model (measurements nested in participants) and included the study as a fixed effects control variable. We inspected residuals of multilevel models to check modeling assumptions (i.e., normality and homoscedasticity of residuals). We visualised person averages using scatter plots as well as individual slopes using scatter and spaghetti plots[122,123].

S4: Sensitivity and Robustness Analyses of one-stage Individual Participant Data models

As there is an increasing call for ordinal instead of metric models to analyze Likert-scaled data[124], we calculated ordinal multilevel regression models using the package ordinal[125] as robustness analyses for antecedent one-stage individual participant data (IPD) models. Note that the ordinal model for negative affective states never converged, so that we do not present the results.

Further, we included bootstrapped confidence intervals[126] of both antecedent and consequent one-stage IPD models to ensure robustness in case of violated statistical assumptions. For bootstrapping, we used the R package lmeresampler[127].

Table S4.1a: Antecedent ordinal model positive affective states

	Ordinal positive affective states		
Predictors	*Odds Ratios*	*CI*	*p*
1|2	0.01	0.01 – 0.01	**<.001**
2|3	0.35	0.30 – 0.40	**<.001**
3|4	11.90	10.32 – 13.72	**<.001**
person-mean PA (SD-scaled)	0.89	0.83 – 0.95	**.001**
person-mean-centered PA (SD-scaled)	1.13	1.12 – 1.15	**<.001**
age	1.00	1.00 – 1.01	**.002**
sex [female]	0.70	0.63 – 0.78	**<.001**
sex [non-binary]	0.13	0.01 – 2.44	.172
**Random Effects**			
σ^2	3.29		
$\tau_{00\ id}$	4.31		
ICC	0.57		
N_{id}	6,159		
Observations	225,932		

Note. To achieve convergence, we first removed the random intercept of study and controlled for study as a fixed effect. In this model, the variance-covariance matrix of the parameters was not defined. Hence, we completely omitted the predictor study from the ordinal model predicting positive affective states.

Table S4.1b: Antecedent ordinal model negative affective states

Not presented due to the lack of convergence even after removing the random intercept of study. Parameters could not be uniquely determined, and while the absolute convergence criterion was met, the relative criterion was not. When omitting the fixed effect of study, the convergence criteria were met but the variance-covariance matrix of the parameters was not defined.

Table S4.1c: Antecedent ordinal model valence

	Ordinal valence		
Predictors	*Odds Ratios*	*99.2% CI*	*p*
1|2	0.01	0.00 – 0.01	**<.001**
2|3	0.19	0.11 – 0.32	**<.001**
3|4	5.08	2.96 – 8.71	**<.001**
person-mean PA (SD-scaled)	1.50	1.20 – 1.87	**<.001**
person-mean-centered PA (SD-scaled)	1.11	1.08 – 1.13	**<.001**
age	1.01	1.00 – 1.02	.036
sex [female]	0.80	0.67 – 0.95	.013
**Random Effects**			
σ^2	3.29		
$\tau_{00\ id}$	3.06		
$\tau_{00\ study}$	0.72		
ICC	0.53		
N_{id}	1,947		
N_{study}	22		
Observations	53,274		

Table S4.1d: Antecedent ordinal model energetic arousal

	ordinal energetic arousal		
Predictors	*Odds Ratios*	*99.2% CI*	*p*
1|2	0.03	0.02 – 0.04	**<.001**
2|3	0.76	0.56 – 1.02	.068
3|4	12.44	9.19 – 16.85	**<.001**
person-mean PA (SD-scaled)	1.44	1.22 – 1.71	**<.001**
person-mean-centered PA (SD-scaled)	1.31	1.29 – 1.34	**<.001**
age	1.02	1.01 – 1.03	**<.001**
sex [female]	0.67	0.59 – 0.77	**<.001**
**Random Effects**			
σ^2	3.29		
$\tau_{00\ id}$	1.64		
$\tau_{00\ study}$	0.05		
ICC	0.34		
N_{id}	1,751		
N_{study}	19		
Observations	50,105		

Table S4.1e: Antecedent ordinal model calmness

	ordinal calmness		
Predictors	*Odds Ratios*	*99.2% CI*	*p*
1|2	0.01	0.00 – 0.01	**<.001**
2|3	0.17	0.11 – 0.27	**<.001**
3|4	4.59	2.93 – 7.18	**<.001**
person-mean PA (SD-scaled)	1.16	0.91 – 1.46	.225
person-mean-centered PA (SD-scaled)	0.90	0.88 – 0.92	**<.001**
age	1.00	0.99 – 1.01	.392
sex [female]	0.76	0.64 – 0.90	**.002**
Random Effects			
σ^2	3.29		
$\tau_{00\ id}$	2.62		
$\tau_{00\ study}$	0.20		
ICC	0.46		
N_{id}	1,641		
N_{study}	18		
Observations	48,399		

Table S4.2a: Antecedent bootstrapped confidence intervals positive affective states

	positive affective states	
Predictors	*Estimates*	*99.2% CI*
(Intercept)	2.59	2.51 – 2.67
person-mean PA (SD-scaled)	0.09	0.04 – 0.12
person-mean-centered PA (SD-scaled)	0.04	0.03 – 0.05
age	0.00	-0.00 – 0.00
sex [female]	-0.04	-0.08 – -0.01
sex [non-binary]	-0.06	-0.87 – 0.69

Note. Instead of the wild bootstrap a residual bootstrap was employed due to computational limits.

Table S4.2b: Antecedent bootstrapped confidence intervals negative affective states

	negative affective states	
Predictors	*Estimates*	*99.2% CI*
(Intercept)	1.56	1.51 – 1.62
person-mean PA (SD-scaled)	-0.02	-0.05 – 0.00
person-mean-centered PA (SD-scaled)	-0.01	-0.01 – -0.00
age	-0.00	-0.00 – -0.00
sex [female]	0.02	-0.00 – 0.05
sex [non-binary]	0.14	-0.18 – 0.49

Note. Instead of the wild bootstrap a residual bootstrap was employed due to computational limits.

Table S4.2c: Antecedent bootstrapped confidence intervals valence

	valence	
Predictors	*Estimates*	*99.2% CI*
(Intercept)	3.02	2.92 – 3.12
person-mean PA (SD-scaled)	0.08	0.03 – 0.14
person-mean-centered PA (SD-scaled)	0.03	0.02 – 0.04
age	0.00	-0.00 – 0.00
sex [female]	-0.05	-0.10 – 0.00

Table S4.2d: Antecedent bootstrapped confidence intervals energetic arousal

	energetic arousal	
Predictors	*Estimates*	*99.2% CI*
(Intercept)	2.65	2.56 – 2.74
person-mean PA (SD-scaled)	0.10	0.03 – 0.16
person-mean-centered PA (SD-scaled)	0.10	0.09 – 0.11
age	0.01	0.00 – 0.01
sex [female]	-0.12	-0.17 – -0.07

Table S4.2e: Antecedent bootstrapped confidence intervals calmness

	calmness	
Predictors	*Estimates*	*99.2% CI*
(Intercept)	3.03	2.93 – 3.12
person-mean PA (SD-scaled)	0.02	-0.04 – 0.09
person-mean-centered PA (SD-scaled)	-0.04	-0.05 – -0.02
age	0.00	-0.00 – 0.00
sex [female]	-0.07	-0.12 – -0.01

Table S4.3a: Consequent bootstrapped confidence intervals positive affective states

	Physical Activity (SD-scaled)	
Predictors	*Estimates*	*99.2% CI*
(Intercept)	0.83	0.80 – 0.88
person-mean positive affective states	0.04	0.03 – 0.06
person-mean-centered positive affective states	0.04	0.03 – 0.06
age	-0.00	-0.00 – -0.00
sex [female]	-0.01	-0.02 – 0.00

Note. Instead of the wild bootstrap a residual bootstrap was employed due to computational limits.

Table S4.3b: Consequent bootstrapped confidence intervals negative affective states

	Physical Activity (SD-scaled)	
Predictors	*Estimates*	*99.2% CI*
(Intercept)	1.05	1.00 – 1.09
person-mean negative affective states	-0.03	-0.05 – -0.01
person-mean-centered negative affective states	-0.02	-0.03 – -0.00
age	-0.00	-0.00 – -0.00
sex [female]	-0.01	-0.03 – 0.00
sex [non-binary]	0.03	-0.20 – 0.26

Note. Instead of the wild bootstrap a residual bootstrap was employed due to computational limits.

Table S4.3c: Consequent bootstrapped confidence intervals valence

	Physical Activity (SD-scaled)	
Predictors	*Estimates*	*99.2% CI*
(Intercept)	0.75	0.61 – 0.90
person-mean valence	0.03	-0.01 – 0.08
person-mean-centered valence	0.03	0.01 – 0.05
age	-0.00	-0.00 – 0.00
sex [female]	0.01	-0.03 – 0.04

Table S4.3d: Consequent bootstrapped confidence intervals energetic arousal

	Physical Activity (SD-scaled)	
Predictors	*Estimates*	*99.2% CI*
(Intercept)	0.82	0.65 – 0.99
person-mean energetic arousal	0.01	-0.05 – 0.07
person-mean-centered energetic arousal	0.10	0.08 – 0.11
age	-0.00	-0.00 – 0.00
sex [female]	0.01	-0.03 – 0.05

Table S4.3e: Consequent bootstrapped confidence intervals calmness

	Physical Activity (SD-scaled)	
Predictors	*Estimates*	*99.2% CI*
(Intercept)	0.85	0.70 – 1.00
person-mean calmness	0.01	-0.04 – 0.05
person-mean-centered calmness	-0.06	-0.07 – -0.04
age	-0.00	-0.00 – 0.00
sex [female]	0.01	-0.02 – 0.04

Table S4.4a: Consequent one-stage IPD model positive affective states without four studies with systematically larger intercepts

	PA (SD-scaled, square-root-transformed)		
Predictors	*Estimates*	*99.2% CI*	*p*
(Intercept)	0.65	0.50 – 0.81	**<.001**
person-mean positive affective states	0.04	0.03 – 0.06	**<.001**
person-mean-centered positive affective states	0.04	0.01 – 0.07	**.001**
age	-0.00	-0.00 – -0.00	**<.001**
sex [female]	-0.01	-0.03 – 0.00	.043
Random Effects			
σ^2	0.22		
$\tau_{00\ id:study}$	0.02		
$\tau_{00\ study}$	0.05		
$\tau_{11\ id:study.mc\_posaff}$	0.01		
$\tau_{11\ study.mc\_posaff}$	0.00		
$\rho_{01\ id:study}$	0.15		
$\rho_{01\ study}$	-0.03		
ICC	0.26		
N_{id}	3,612		
N_{study}	21		
Observations	131,942		

Table S4.4b: Consequent one-stage IPD model negative affective states without four studies with systematically larger intercepts

	PA (SD-scaled, square-root-transformed)		
Predictors	*Estimates*	*99.2% CI*	*p*
(Intercept)	0.89	0.68 – 1.11	**<.001**
person-mean negative affective states	-0.03	-0.05 – -0.01	**<.001**
person-mean-centered negative affective states	-0.01	-0.04 – 0.02	.176
age	-0.00	-0.00 – -0.00	**<.001**
sex [female]	-0.01	-0.03 – 0.00	.029
sex [non-binary]	0.03	-0.23 – 0.28	.765
Random Effects			
σ^2	0.22		
$\tau_{00\ id:study}$	0.02		
$\tau_{00\ study}$	0.12		
$\tau_{11\ id:study.mc\_negaff}$	0.01		
$\tau_{11\ study.mc\_negaff}$	0.00		
$\rho_{01\ id:study}$	-0.05		
$\rho_{01\ study}$	-0.50		
ICC	0.39		
N_{id}	3,927		
N_{study}	23		
Observations	141,890		

Table S4.4c: Consequent one-stage IPD model valence without one study with systematically larger intercepts

	PA (SD-scaled, square-root-transformed)		
Predictors	*Estimates*	*99.2% CI*	*p*
(Intercept)	0.70	0.56 – 0.85	<.001
person-mean valence	0.03	-0.00 – 0.06	**.008**
person-mean-centered valence	0.03	0.00 – 0.05	**.005**
age	-0.00	-0.00 – 0.00	.123
sex [female]	-0.00	-0.03 – 0.02	.671
**Random Effects**			
σ^2	0.16		
$\tau_{00\ id:study}$	0.02		
$\tau_{00\ study}$	0.01		
$\tau_{11\ id:study.mc\ valence}$	0.01		
$\tau_{11\ study.mc\ valence}$	0.00		
$\rho_{01\ id:study}$	-0.05		
$\rho_{01\ study}$	-0.56		
ICC	0.15		
N_{id}	1235		
N_{study}	10		
Observations	42,578		

Table S4.4d: Consequent one-stage IPD model energetic arousal without one study with systematically larger intercepts

	PA (SD-scaled, square-root-transformed)		
Predictors	*Estimates*	*99.2% CI*	*p*
(Intercept)	0.77	0.62 – 0.92	**<.001**
person-mean energetic arousal	0.01	-0.02 – 0.04	.641
person-mean-centered energetic arousal	0.10	0.06 – 0.14	**<.001**
age	-0.00	-0.00 – 0.00	.266
sex [female]	-0.00	-0.03 – 0.02	.816
**Random Effects**			
σ^2	0.16		
$\tau_{00\ id:study}$	0.01		
$\tau_{00\ study}$	0.01		
$\tau_{11\ id:study.mc\_energetic}$	0.01		
$\tau_{11\ study.mc\_energetic}$	0.00		
$\rho_{01\ id:study}$	0.24		
$\rho_{01\ study}$	0.90		
ICC	0.16		
N_{id}	1,152		
N_{study}	9		
Observations	41,595		

Table S4.4e: Consequent one-stage IPD model calmness without one study with systematically larger intercepts

	PA (SD-scaled, square-root-transformed)		
Predictors	*Estimates*	*99.2% CI*	*p*
(Intercept)	0.77	0.66 – 0.88	**<.001**
person-mean calmness	0.00	-0.03 – 0.03	.759
person-mean-centered calmness	-0.07	-0.08 – -0.05	**<.001**
age	0.00	-0.00 – 0.00	.702
sex [female]	0.00	-0.02 – 0.03	.741
controlled for: study			
Random Effects			
σ^2	0.16		
$\tau_{00\ id}$	0.01		
$\tau_{11\ id.mc\_calmness}$	0.02		
$\rho_{01\ id}$	-0.20		
ICC	0.10		
N_{id}	1,042		
Observations	39,886		

Note. Due to singularity in the three-level model (measurements nested in participants nested in studies), the third level (study) was dropped and a two-level model (measurements nested in participants) employed. We introduced study as a fixed effect control variable.

Table S4.5a: Antecedent one-stage IPD model positive affective states following PANAS

	Positive affective states		
Predictors	*Estimates*	*99.2% CI*	*p*
(Intercept)	2.26	1.92 – 2.60	**<.001**
person-mean PA (SD-scaled)	0.10	0.02 – 0.18	**.001**
person-mean-centered PA (SD-scaled)	0.07	0.04 – 0.10	**<.001**
age	0.01	0.00 – 0.01	**.001**
sex [female]	-0.03	-0.12 – 0.06	.383
**Random Effects**			
σ^2	0.19		
$\tau_{00\ id:study}$	0.30		
$\tau_{00\ study}$	0.08		
$\tau_{11\ id:study.mc\_PA\_bp\_zwm}$	0.01		
$\tau_{11\ study.mc\_PA\_bp\_zwm}$	0.00		
$\rho_{01\ id:study}$	-0.10		
$\rho_{01\ study}$	0.02		
ICC	0.68		
N $_{id}$	1,335		
N $_{study}$	10		
Observations	51,649		

Table S4.5b: Antecedent one-stage IPD model negative affective states following PANAS

	Negative affective states		
Predictors	*Estimates*	*99.2% CI*	*p*
(Intercept)	1.40	1.17 – 1.63	**<.001**
person-mean PA (SD-scaled)	0.01	-0.04 – 0.05	.756
person-mean-centered PA (SD-scaled)	-0.00	-0.02 – 0.01	.326
age	-0.00	-0.01 – -0.00	.004
sex [female]	0.04	-0.02 – 0.09	.072
**Random Effects**			
σ^2	0.10		
$\tau_{00\ id:study}$	0.11		
$\tau_{00\ study}$	0.04		
$\tau_{11\ id:study.mc\_PA\_bp\_zwm}$	0.00		
$\tau_{11\ study.mc\_PA\_bp\_zwm}$	0.00		
$\rho_{01\ id:study}$	-0.18		
$\rho_{01\ study}$	-0.00		
ICC	0.60		
N $_{id}$	1,336		
N $_{study}$	10		
Observations	51,655		

Table S4.5c: Antecedent one-stage IPD model valence using the short-version of the MDMQ

	Valence		
Predictors	*Estimates*	*99.2% CI*	*p*
(Intercept)	2.96	2.81 – 3.12	**<.001**
person-mean PA (SD-scaled)	0.10	0.02 – 0.18	**.001**
person-mean-centered PA (SD-scaled)	0.03	0.01 – 0.04	**<.001**
age	0.00	-0.00 – 0.01	.013
sex [female]	-0.03	-0.09 – 0.03	.191
**Random Effects**			
σ^2	0.22		
$\tau_{00\ id:study}$	0.16		
$\tau_{00\ study}$	0.01		
$\tau_{11\ id:study.mc\_PA\_bp\_zwm}$	0.00		
$\tau_{11\ study.mc\_PA\_bp\_zwm}$	0.00		
$\rho_{01\ id:study}$	-0.10		
$\rho_{01\ study}$	-0.50		
ICC	0.44		
N_{id}	1,465		
N_{study}	17		
Observations	43,824		

Table S4.5d: Antecedent one-stage IPD model energetic arousal using the short-version of the MDMQ

	energetic		
Predictors	*Estimates*	*99.2% CI*	*p*
(Intercept)	2.61	2.47 – 2.75	**<.001**
person-mean PA (SD-scaled)	0.11	0.04 – 0.19	**<.001**
person-mean-centered PA (SD-scaled)	0.12	0.08 – 0.16	**<.001**
age	0.01	0.00 – 0.01	**<.001**
sex [female]	-0.12	-0.17 – -0.06	**<.001**
**Random Effects**			
σ^2	0.34		
$\tau_{00\ id:study}$	0.14		
$\tau_{00\ study}$	0.01		
$\tau_{11\ id:study.mc\_PA\_bp\_zwm}$	0.01		
$\tau_{11\ study.mc\_PA\_bp\_zwm}$	0.00		
$\rho_{01\ id:study}$	-0.06		
$\rho_{01\ study}$	-0.18		
ICC	0.31		
N_{id}	1,465		
N_{study}	17		
Observations	43,835		

Table S4.5e: Antecedent one-stage IPD model calmness using the short-version of the MDMQ

	Calmness		
Predictors	*Estimates*	*99.2% CI*	*p*
(Intercept)	3.01	2.86 – 3.17	<.001
person-mean PA (SD-scaled)	0.03	-0.05 – 0.11	.267
person-mean-centered PA (SD-scaled)	-0.04	-0.07 – -0.00	**.003**
age	0.00	-0.00 – 0.00	.324
sex [female]	-0.07	-0.13 – -0.01	.004
**Random Effects**			
σ^2	0.24		
$\tau_{00\ id:study}$	0.16		
$\tau_{00\ study}$	0.01		
$\tau_{11\ id:study.mc\_PA\_bp\_zwm}$	0.01		
$\tau_{11\ study.mc\_PA\_bp\_zwm}$	0.00		
$\rho_{01\ id:study}$	0.02		
$\rho_{01\ study}$	-0.06		
ICC	0.42		
N id	1,465		
N study	17		
Observations	43,824		

Table S4.6a: Consequent one-stage IPD model positive affective states following PANAS

	PA (SD-scaled, square-root-transformed)		
Predictors	*Estimates*	*CI*	*p*
(Intercept)	0.63	0.49 – 0.76	**<.001**
person-mean positive affective states	0.04	0.01 – 0.08	**.001**
person-mean-centered positive affective states	0.04	0.02 – 0.06	**<.001**
age	-0.01	-0.01 – -0.00	**<.001**
sex [female]	-0.02	-0.06 – 0.02	.227
controlled for: study			
**Random Effects**			
σ^2	0.16		
$\tau_{00\ id}$	0.02		
$\tau_{11\ id.mc\_posaff}$	0.00		
$\rho_{01\ id}$	0.70		
ICC	0.13		
N id	551		
Observations	13,190		

Note. Due to singularity in the three-level model (measurements nested in participants nested in studies), the third level (study) was dropped and a two-level model (measurements nested in participants) employed. We introduced study as a fixed effect control variable.

Table S4.6b: Consequent one-stage IPD model negative affective states following PANAS
Not presented due to the lack of convergence even after removing the random intercept of study. Only four studies were included in this analysis.

Table S4.6c: Consequent one-stage IPD model valence using the short-version of the MDMQ

	PA (SD-scaled, square-root-transformed)		
Predictors	*Estimates*	*99.2% CI*	*p*
(Intercept)	0.75	0.44 – 1.07	**<.001**
person-mean valence	0.04	0.01 – 0.06	**.001**
person-mean-centered valence	0.02	-0.01 – 0.06	.022
age	0.00	-0.00 – 0.00	.678
sex [female]	0.02	-0.00 – 0.05	.011
**Random Effects**			
σ^2	0.16		
$\tau_{00\ id:study}$	0.01		
$\tau_{00\ study}$	0.06		
$\tau_{11\ id:study.mc\ valence}$	0.01		
$\tau_{11\ study.mc\ valence}$	0.00		
$\rho_{01\ id:study}$	-0.06		
$\rho_{01\ study}$	0.15		
ICC	0.32		
N_{id}	984		
N_{study}	8		
Observations	36,945		

Table S4.6d: Consequent one-stage IPD model energetic arousal using the short-version of the MDMQ

	PA (SD-scaled, square-root-transformed)		
Predictors	*Estimates*	*99.2% CI*	*p*
(Intercept)	0.78	0.46 – 1.10	<.001
person-mean energetic arousal	0.03	-0.00 – 0.06	.011
person-mean-centered energetic arousal	0.10	0.05 – 0.15	**.001**
age	0.00	-0.00 – 0.00	.702
sex [female]	0.03	0.00 – 0.05	**.004**
**Random Effects**			
σ^2	0.15		
$\tau_{00\ id:study}$	0.01		
$\tau_{00\ study}$	0.06		
$\tau_{11\ id:study.mc\ energetic}$	0.01		
$\tau_{11\ study.mc\ energetic}$	0.00		
$\rho_{01\ id:study}$	0.22		
$\rho_{01\ study}$	-0.84		
ICC	0.33		
N id	984		
N study	8		
Observations	36,954		

Table S4.6e: Consequent one-stage IPD model calmness using the short-version of the MDMQ

	PA (SD-scaled, square-root-transformed)		
Predictors	*Estimates*	*99.2% CI*	*p*
(Intercept)	0.82	0.50 – 1.14	**<.001**
person-mean calmness	0.01	-0.01 – 0.04	.200
person-mean-centered calmness	-0.06	-0.11 – -0.01	**.003**
age	0.00	-0.00 – 0.00	.654
sex [female]	0.03	0.00 – 0.05	**.007**
**Random Effects**			
σ^2	0.16		
$\tau_{00\ id:study}$	0.01		
$\tau_{00\ study}$	0.06		
$\tau_{11\ id:study.mc\ calmness}$	0.02		
$\tau_{11\ study.mc\ calmness}$	0.00		
$\rho_{01\ id:study}$	-0.17		
$\rho_{01\ study}$	0.75		
ICC	0.33		
N id	984		
N study	8		
Observations	36,945		

S5: Results of antecedent one-stage Individual Participant Data models

In all antecedent one-stage IPD models, Physical Activity (PA) was person-mean-centered; both participants' average and mean-centered PA were used as predictors. We included a random intercept and a random slope for mean-centered PA. With respect to the hierarchical structure of the data, we applied three-level models (measurements nested in participants nested in studies) using restricted maximum-likelihood (REML) estimation and the bobyqa optimiser for better convergence. We controlled for participants' age and sex/gender.

Table S5.1: Antecedent one-stage IPD model positive affective states

	positive affective states			positive affective states		
Predictors	*Estimates*	*99.2% CI*	*p*	*Estimates*	*99.2% CI*	*p*
(Intercept)	2.67	2.53 – 2.81	**<.001**	2.59	2.42 – 2.75	**<.001**
person-mean PA (SD-scaled)				0.09	0.05 – 0.12	**<.001**
person-mean-centered PA (SD-scaled)				0.04	0.02 – 0.05	**<.001**
age				-0.00	-0.00 – 0.00	.948
sex [female]				-0.04	-0.08 – -0.01	**.001**
sex [non-binary]				-0.06	-0.96 – 0.85	.871
Random Effects						
σ^2	0.25			0.25		
τ_{00}	0.22 $_{id:study}$			0.22 $_{id:study}$		
	0.11 $_{study}$			0.12 $_{study}$		
τ_{11}				0.00 $_{id:study.mc\_PA\_bp\_zwm}$		
				0.00 $_{study.mc\_PA\_bp\_zwm}$		
ρ_{01}				-0.09 $_{id:study}$		
				-0.42 $_{study}$		
ICC	0.56			0.58		
N	6,159 $_{id}$			6,159 $_{id}$		
	44 $_{study}$			44 $_{study}$		
Observations	225,932			225,932		

Table S5.2: Antecedent one-stage IPD model negative affective states

	negative affective states			negative affective states		
Predictors	*Estimates*	*99.2% CI*	*p*	*Estimates*	*99.2% CI*	*p*
(Intercept)	1.48	1.37 – 1.59	**<.001**	1.56	1.44 – 1.69	**<.001**
person-mean PA (SD-scaled)				-0.02	-0.05 – 0.00	.024
person-mean-centered PA (SD-scaled)				-0.01	-0.01 – 0.00	.067
age				-0.00	-0.00 – -0.00	**<.001**
sex [female]				0.02	-0.00 – 0.04	.026
sex [non-binary]				0.14	-0.28 – 0.55	.387
Random Effects						
σ^2	0.13			0.13		
τ_{00}	0.12 $_{id:study}$			0.12 $_{id:study}$		
	0.07 $_{study}$			0.07 $_{study}$		
τ_{11}				0.00 $_{id:study.mc\_PA\_bp\_zwm}$		
				0.00 $_{study.mc\_PA\_bp\_zwm}$		
ρ_{01}				-0.13 $_{id:study}$		
				-0.39 $_{study}$		
ICC	0.58			0.59		
N	6,429 $_{id}$			6,429 $_{id}$		
	45 $_{study}$			45 $_{study}$		
Observations	235,537			235,537		

Table S5.3: Antecedent one-stage IPD model valence

	valence			valence		
Predictors	*Estimates*	*99.2% CI*	*p*	*Estimates*	*99.2% CI*	*p*
(Intercept)	3.13	3.00 – 3.27	**<.001**	3.02	2.85 – 3.19	**<.001**
person-mean PA (SD-scaled)				0.08	0.01 – 0.15	**.001**
person-mean-centered PA (SD-scaled)				0.03	0.01 – 0.04	**<.001**
age				0.00	-0.00 – 0.00	.082
sex [female]				-0.05	-0.10 – 0.00	.012
Random Effects						
σ^2	0.22			0.22		
τ_{00}	0.15 $_{id:study}$			0.15 $_{id:study}$		
	0.04 $_{study}$			0.04 $_{study}$		
τ_{11}				0.00 $_{id:study.mc\_PA\_bp\_zwm}$		
				0.00 $_{study.mc\_PA\_bp\_zwm}$		
ρ_{01}				-0.10 $_{id:study}$		
				-0.90 $_{study}$		
ICC	0.47			0.48		
N	1,947 $_{id}$			1,947 $_{id}$		
	22 $_{study}$			22 $_{study}$		
Observations	53,274			53,274		

Table S5.4: Antecedent one-stage IPD model energetic arousal

	energetic arousal			energetic arousal		
Predictors	*Estimates*	*99.2% CI*	*p*	*Estimates*	*99.2% CI*	*p*
(Intercept)	2.87	2.78 – 2.96	**<.001**	2.65	2.53 – 2.77	**<.001**
person-mean PA (SD-scaled)				0.10	0.03 – 0.16	**<.001**
person-mean-centered PA (SD-scaled)				0.10	0.06 – 0.15	**<.001**
age				0.01	0.00 – 0.01	**<.001**
sex [female]				-0.12	-0.17 – -0.07	**<.001**
Random Effects						
σ^2	0.33			0.32		
τ_{00}	0.14 id:study			0.14 id:study		
	0.01 study			0.01 study		
τ_{11}				0.01 id:study.mc_PA_bp_zwm		
				0.00 study.mc_PA_bp_zwm		
ρ_{01}				-0.06 id:study		
				-0.41 study		
ICC	0.32			0.32		
N	1,751 id			1,751 id		
	19 study			19 study		
Observations	50,105			50,105		

Table S5.5: Antecedent one-stage IPD model calmness

	calmness			calmness		
Predictors	*Estimates*	*99.2% CI*	*p*	*Estimates*	*99.2% CI*	*p*
(Intercept)	3.05	2.96 – 3.14	**<.001**	3.03	2.89 – 3.18	**<.001**
person-mean PA (SD-scaled)				0.02	-0.05 – 0.09	.457
person-mean-centered PA (SD-scaled)				-0.04	-0.06– -0.01	**.002**
age				0.00	-0.00 – 0.00	.394
sex [female]				-0.07	-0.12 – -0.01	**.002**
Random Effects						
σ^2	0.24			0.24		
τ_{00}	0.15 $_{id:study}$			0.15 $_{id:study}$		
	0.01 $_{study}$			0.01 $_{study}$		
τ_{11}				0.01 $_{id:study.mc\_PA\_bp\_zwm}$		
				0.00 $_{study.mc\_PA\_bp\_zwm}$		
ρ_{01}				0.01 $_{id:study}$		
				-0.07 $_{study}$		
ICC	0.41			0.42		
N	1,641 $_{id}$			1,641 $_{id}$		
	18 $_{study}$			18 $_{study}$		
Observations	48,399			48,399		

S6: Results of antecedent one-stage Movement Acceleration Intensity Individual Participant Data models

Next, we restricted analyses to datasets including raw accelerometry data as Movement Acceleration Intensity (MAI; in milli-*g*). Thereby, we aim at ensuring the robustness of effects in light of the wide range of diverse PA metrics jointly analyzed in the one-stage IPD analyses (S3, S5). Also, for the antecedent models, we used one-stage MAI IPD analyses to derive practical effect sizes. In all antecedent one-stage MAI IPD models, PA was person-mean-centered; both participants' average and mean-centered PA were used as predictors. We included a random intercept and a random slope for mean-centered PA. With respect to the hierarchical structure of the data, we applied three-level models (measurements nested in participants nested in studies) using restricted maximum-likelihood (REML) estimation and the bobyqa optimiser for better convergence. We also controlled for participants' age and sex/gender as well as the PA aggregation interval (in minutes).

Table S6.1: Antecedent one-stage MAI IPD model positive affective states

	positive affective states		
Predictors	*Estimates*	*99.2% CI*	*p*
(Intercept)	2.67	1.76 – 3.59	**<.001**
person-mean MAI (in deci-*g*)	0.16	0.05 – 0.28	**<.001**
person-mean-centered MAI (in deci-*g*)	0.05	0.02 – 0.08	**.002**
age	-0.01	-0.02 – -0.00	**<.001**
sex [female]	-0.10	-0.18 – -0.02	**.001**
PA aggregation interval (in min.)	0.02	-0.09 – 0.13	.161
Random Effects			
σ^2	0.18		
$\tau_{00\ id:study}$	0.17		
$\tau_{00\ study}$	0.02		
$\tau_{11\ id:study.mc\_mai\_bp\_sc100}$	0.00		
$\tau_{11\ study.mc\_mai\_bp\_sc100}$	0.00		
$\rho_{01\ id:study}$	-0.12		
$\rho_{01\ study}$	-0.81		
ICC	0.51		
N_{id}	839		
N_{study}	5		
Observations	33,651		

Note. For better convergence, MAI was included in deci-*g* (1 deci-$g = \frac{1}{10}\,g$).

Table S6.2: Antecedent one-stage MAI IPD model negative affective states

	negative affective states		
Predictors	*Estimates*	*99.2% CI*	*p*
(Intercept)	1.28	0.59 – 1.97	**<.001**
person-mean MAI (in deci-*g*)	-0.02	-0.11 – 0.08	.645
person-mean-centered MAI (in deci-*g*)	-0.01	-0.05 – 0.03	.140
age	0.00	-0.01 – 0.01	.898
sex [female]	0.04	-0.02 – 0.11	.064
PA aggregation interval (in min.)	0.01	-0.02 – 0.04	.335
Random Effects			
σ^2	0.08		
$\tau_{00\ id:study}$	0.11		
$\tau_{00\ study}$	0.05		
$\tau_{11\ id:study.mc\_mai\_bp\_sc100}$	0.00		
$\tau_{11\ study.mc\_mai\_bp\_sc100}$	0.00		
$\rho_{01\ id:study}$	-0.30		
$\rho_{01\ study}$	-0.96		
ICC	0.66		
N_{id}	839		
N_{study}	5		
Observations	33,638		

Note. For better convergence, MAI was included in deci-*g* (1 deci-*g* = $\frac{1}{10}$ *g*).

Table S6.3: Antecedent one-stage MAI IPD model valence

	valence		
Predictors	*Estimates*	*99.2% CI*	*p*
(Intercept)	3.11	2.90 – 3.32	**<.001**
person-mean MAI (in deci-*g*)	0.06	-0.02 – 0.15	.045
person-mean-centered MAI (in deci-*g*)	0.03	0.02 – 0.05	**<.001**
age	0.00	-0.00 – 0.00	.440
sex [female]	-0.02	-0.09 – 0.04	.291
PA aggregation interval (in min.)	-0.00	-0.01 – 0.00	.178
Random Effects			
σ^2	0.20		
$\tau_{00\ id:study}$	0.16		
$\tau_{00\ study}$	0.02		
$\tau_{11\ id:study.mc\_mai\_bp\_sc100}$	0.00		
$\tau_{11\ study.mc\_mai\_bp\_sc100}$	0.00		
$\rho_{01\ id:study}$	-0.12		
$\rho_{01\ study}$	-0.77		
ICC	0.46		
N_{id}	1,393		
N_{study}	16		
Observations	43,361		

Note. For better convergence, MAI was included in deci-*g* (1 deci-*g* = $\frac{1}{10}$ *g*).

Table S6.4: Antecedent one-stage MAI IPD model energetic arousal

	energetic arousal		
Predictors	*Estimates*	*99.2% CI*	*p*
(Intercept)	2.62	2.28 – 2.96	**<.001**
person-mean MAI (in deci-*g*)	0.07	-0.01 – 0.15	.020
person-mean-centered MAI (in deci-*g*)	0.13	0.08 – 0.19	**<.001**
age	0.01	0.00 – 0.01	**<.001**
sex [female]	-0.09	-0.15 – -0.03	**<.001**
PA aggregation interval (in min.)	0.00	-0.02 – 0.03	.744
Random Effects			
σ^2	0.30		
$\tau_{00\ id:study}$	0.13		
$\tau_{00\ study}$	0.00		
$\tau_{11\ id:study.mc\_mai\_bp\_sc100}$	0.01		
$\tau_{11\ study.mc\_mai\_bp\_sc100}$	0.00		
$\rho_{01\ id:study}$	-0.06		
$\rho_{01\ study}$	-0.77		
ICC	0.32		
N $_{id}$	1,322		
N $_{study}$	15		
Observations	41,561		

Note. For better convergence, MAI was included in deci-*g* (1 deci-*g* = $\frac{1}{10}$ *g*).

Table S6.5: Antecedent one-stage MAI IPD model calmness

	calmness		
Predictors	*Estimates*	*99.2% CI*	*p*
(Intercept)	3.17	2.69 – 3.66	**<.001**
person-mean MAI (in deci-*g*)	-0.02	-0.10 – 0.07	.596
person-mean-centered MAI (in deci-*g*)	-0.05	-0.08 – -0.01	**.001**
age	0.00	-0.00 – 0.00	.747
sex [female]	-0.05	-0.11 – 0.01	.026
PA aggregation interval (in min.)	-0.01	-0.04 – 0.03	.449
Random Effects			
σ^2	0.22		
$\tau_{00\ \text{id:study}}$	0.15		
$\tau_{00\ \text{study}}$	0.01		
$\tau_{11\ \text{id:study.mc\_mai\_bp\_sc100}}$	0.01		
$\tau_{11\ \text{study.mc\_mai\_bp\_sc100}}$	0.00		
$\rho_{01\ \text{id:study}}$	0.03		
$\rho_{01\ \text{study}}$	-0.58		
ICC	0.43		
N_{id}	1,322		
N_{study}	15		
Observations	41,551		

Note. For better convergence, MAI was included in deci-*g* (1 deci-*g* = $\frac{1}{10}$ *g*).

Figure S6: Practical Effect sizes (one-stage MAI IPD analysis) of the intensity of Physical Activity (PA) on Affective Well-Being (AWB) exemplified for walking (5.4 km/h) in MAI [milli-*g*]

To present practical effect sizes, we inserted MAI values for walking at 5.4 km/h (4.99 deci-*g*; see Figure)[117]; into the regression formulas of the MAI one-stage IPD models. To enable comparisons of the effects with other daily life activities (e.g., with relaxing, eating, or conversation[128]), we transferred effects reported by Killingsworth and Gilbert[128] to a scale from 1 to 4. To this end, we divided the effect by 100 and multiplied it by 3 (the ranges of the respective scales). For example, in Killingsworth and Gilbert's study, taking a walk was related to an increase in happiness of 7 points on a scale from 0 to 100. This translates to an increase of 0.21 points on a scale from 1 to 4.

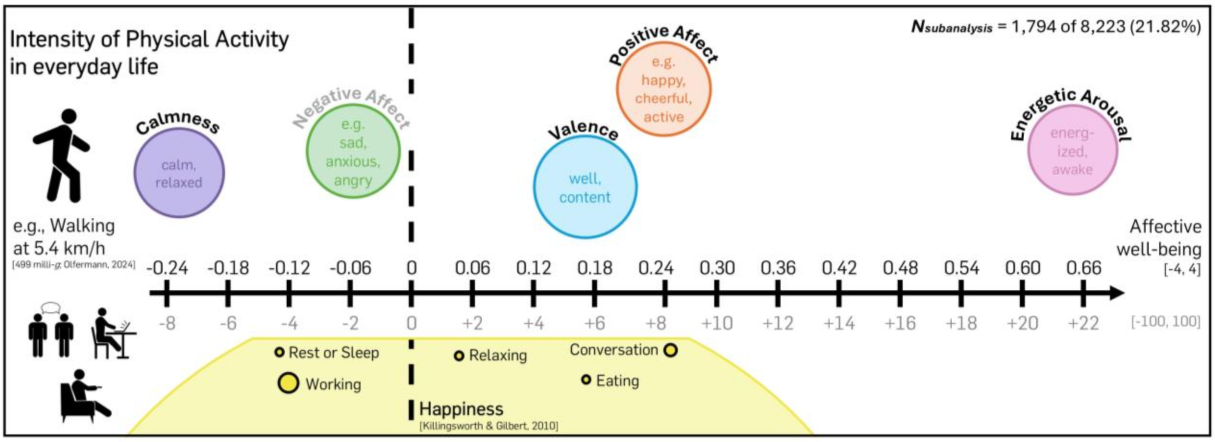

Note. MAI One-stage IPD analyses are based on a subset of data (28.36% of datasets, 21.82% of participants, 20.99% of e-diary prompts). *n* indicates the number of participants and *i* the number of ratings. Practical effect sizes of person-mean-centered physical activity on positive affective states ($t(3.70) = 7.51$, $p = .002$, $n = 839$, $i = 33{,}651$), negative affective states ($t(2.85) = -2.04$, $p = .140$, $n = 839$, $i = 33{,}638$), valence $t(13.88) = 6.84$, $p = .000008$, $n = 1{,}393$, $i = 43{,}361$), calmness ($t(9.36) = -4.56$, $p = .001$, $n = 1{,}322$, $i = 41{,}551$), and energetic arousal ($t(13.83) = 7.32$, $p = .000004$, $n = 1{,}322$, $i = 41{,}561$) were derived from MAI one-stage multilevel models (regression coefficients multiplied by MAI values for walking at 5.4 km/h). Practical effect sizes on affective well-being (x-axis) are compared to other daily life activities reported in Killingsworth & Gilbert[128] in the lower part of the Figure. The bubble size represents the number of datapoints/e-diary prompts. Km/h = kilometers per hour.

S7: Results of consequent one-stage Individual Participant Data models

In order to test whether Affective Well-Being (AWB) is associated with subsequent PA, we again employed three-level models (measurements nested in participants nested in studies; REML, bobyqa optimiser). This time, PA functioned as outcome and we used participants' average AWB and mean-centered AWB as predictors. We included a random intercept and a random slope for mean-centered AWB. To account for the known skewness of PA data, we square-root-transformed PA. We controlled for participants' age and sex/gender.

Table S7.1: Consequent one-stage IPD model positive affective states

	PA (SD-scaled, square-root-transformed)			PA (SD-scaled, square-root-transformed)		
Predictors	*Estimates*	*99.2% CI*	*p*	*Estimates*	*99.2% CI*	*p*
(Intercept)	0.90	0.63 – 1.17	**<.001**	0.83	0.56 – 1.11	**<.001**
person-mean positive affective states				0.04	0.03 – 0.06	**<.001**
person-mean-centered positive affective states				0.04	0.02 – 0.07	**<.001**
age				-0.00	-0.00 – -0.00	**<.001**
sex [female]				-0.01	-0.03 – 0.00	.067
Random Effects						
σ^2	0.22			0.21		
τ_{00}	0.02 $_{id:study}$			0.02 $_{id:study}$		
	0.22 $_{study}$			0.22 $_{study}$		
τ_{11}				0.01 $_{id:study.mc\_posaff}$		
				0.00 $_{study.mc\_posaff}$		
ρ_{01}				0.13 $_{id:study}$		
				0.01 $_{study}$		
ICC	0.53			0.53		
N	3,864 $_{id}$			3,864 $_{id}$		
	25 $_{study}$			25 $_{study}$		
Observations	140,170			140,170		

Table S7.2: Consequent one-stage IPD model negative affective states

	PA (SD-scaled, square-root-transformed)			PA (SD-scaled, square-root-transformed)		
Predictors	*Estimates*	*99.2% CI*	*p*	*Estimates*	*99.2% CI*	*p*
(Intercept)	0.95	0.68 – 1.22	**<.001**	1.05	0.77 – 1.32	**<.001**
person-mean negative affective states				-0.03	-0.05 – -0.01	**<.001**
person-mean-centered negative affective states				-0.02	-0.04 – 0.01	.053
age				-0.00	-0.00 – -0.00	**<.001**
sex [female]				-0.01	-0.03 – 0.00	.043
sex [non-binary]				0.03	-0.22 – 0.28	.755
Random Effects						
σ^2	0.21			0.21		
τ_{00}	0.02 $_{id:study}$			0.02 $_{id:study}$		
	0.24 $_{study}$			0.24 $_{study}$		
τ_{11}				0.01 $_{id:study.mc\_negaff}$		
				0.00 $_{study.mc\_negaff}$		
ρ_{01}				-0.04 $_{id:study}$		
				-0.60 $_{study}$		
ICC	0.55			0.56		
N	4,179 $_{id}$			4,179 $_{id}$		
	27 $_{study}$			27 $_{study}$		
Observations	150,118			150,118		

Table S7.3: Consequent one-stage IPD model valence

	PA (SD-scaled, square-root-transformed)			PA (SD-scaled, square-root-transformed)		
Predictors	*Estimates*	*99.2% CI*	*p*	*Estimates*	*99.2% CI*	*p*
(Intercept)	0.83	0.60 – 1.07	**<.001**	0.75	0.51 – 0.99	**<.001**
person-mean valence				0.03	0.01 – 0.06	**.001**
person-mean-centered valence				0.03	0.01 – 0.05	**.002**
age				-0.00	-0.00 – 0.00	.091
sex [female]				0.01	-0.02 – 0.03	.534
Random Effects						
σ^2	0.16			0.16		
τ_{00}	0.02 $_{\text{id:study}}$			0.02 $_{\text{id:study}}$		
	0.06 $_{\text{study}}$			0.05 $_{\text{study}}$		
τ_{11}				0.01 $_{\text{id:study.mc\_valence}}$		
				0.00 $_{\text{study.mc\_valence}}$		
ρ_{01}				-0.06 $_{\text{id:study}}$		
				0.01 $_{\text{study}}$		
ICC	0.31			0.31		
N	1,354 $_{\text{id}}$			1,354 $_{\text{id}}$		
	11 $_{\text{study}}$			11 $_{\text{study}}$		
Observations	44,251			44,251		

Table S7.4: Consequent one-stage IPD model energetic arousal

	PA (SD-scaled, square-root-transformed)			PA (SD-scaled, square-root-transformed)		
Predictors	*Estimates*	*99.2% CI*	*p*	*Estimates*	*99.2% CI*	*p*
(Intercept)	0.84	0.57 – 1.10	**<.001**	0.82	0.55 – 1.09	**<.001**
person-mean energetic arousal				0.01	-0.02 – 0.04	.275
person-mean-centered energetic arousal				0.10	0.05 – 0.14	**<.001**
age				-0.00	-0.00 – 0.00	.162
sex [female]				0.01	-0.02 – 0.03	.341
Random Effects						
σ^2	0.16			0.15		
τ_{00}	0.01 $_{id:study}$			0.01 $_{id:study}$		
	0.06 $_{study}$			0.06 $_{study}$		
τ_{11}				0.01 $_{id:study.mc\_energetic}$		
				0.00 $_{study.mc\_energetic}$		
ρ_{01}				0.25 $_{id:study}$		
				-0.04 $_{study}$		
ICC	0.32			0.33		
N	1,271 $_{id}$			1,271 $_{id}$		
	10 $_{study}$			10 $_{study}$		
Observations	43,270			43,270		

Table S7.5: Consequent one-stage IPD model calmness

	PA (SD-scaled, square-root-transformed)			PA (SD-scaled, square-root-transformed)		
Predictors	*Estimates*	*99.2% CI*	*p*	*Estimates*	*99.2% CI*	*p*
(Intercept)	0.88	0.61 – 1.15	**<.001**	0.85	0.58 – 1.12	**<.001**
person-mean calmness				0.01	-0.02 – 0.03	.598
person-mean-centered calmness				-0.06	-0.10 – -0.02	**.001**
age				-0.00	-0.00 – 0.00	.982
sex [female]				0.01	-0.01 – 0.03	.204
Random Effects						
σ^2	0.16			0.16		
τ_{00}	0.01 id:study			0.01 id:study		
	0.05 study			0.05 study		
τ_{11}				0.02 id:study.mc_calmness		
				0.00 study.mc_calmness		
ρ_{01}				-0.20 id:study		
				0.80 study		
ICC	0.29			0.31		
N	1161 id			1,161 id		
	9 study			9 study		
Observations	41,561			41,561		

S8: Results of consequent one-stage Movement Acceleration Intensity Individual Participant Data models

Next, we again restricted analyses to datasets including raw accelerometry data as Movement Acceleration Intensity (MAI in milli-*g*). Thereby, we aim at ensuring the robustness of effects in light of the wide range of diverse PA metrics jointly analyzed in the one-stage IPD analyses (S3, S7). PA constituted the outcome in one-stage MAI IPD analyses. As PA data needed to be square-root transformed due to skewness, it was not possible to derive practical effect sizes for consequent one-stage IPD models. We used participants' average AWB and mean-centered AWB as predictors. We included a random intercept and a random slope for mean-centered AWB (REML, bobyqa optimiser). As some of the three-level models predicting PA based on AWB were singular, we used two-level models with study as a fixed effects control variable instead. We controlled for participants' age and sex/gender as well as the PA aggregation interval (in minutes).

Table S8.1: Consequent one-stage MAI IPD model positive affective states

	PA (milli-g, square-root-transformed)		
Predictors	*Estimates*	*99.2% CI*	*p*
(Intercept)	6.32	2.01 – 10.63	**.002**
person-mean positive affective states	0.40	0.08 – 0.73	**.001**
person-mean centered positive affective states	0.61	-0.11 – 1.34	.013
age	-0.03	-0.07 – 0.01	.045
sex [female]	-0.06	-0.33 – 0.21	.529
PA interval [minutes]	0.03	-0.29 – 0.35	.469
Random Effects			
σ^2	11.92		
$\tau_{00\ id:study}$	1.17		
$\tau_{00\ study}$	0.44		
$\tau_{11\ id:study.mc\_posaff}$	0.78		
$\tau_{11\ study.mc\_posaff}$	0.03		
$\rho_{01\ id:study}$	-0.05		
$\rho_{01\ study}$	0.94		
ICC	0.13		
N_{id}	654		
N_{study}	4		
Observations	26,920		

Table S8.2: Consequent one-stage MAI IPD model negative affective states

	PA (milli-*g*, square-root-transformed)		
Predictors	*Estimates*	*99.2% CI*	*p*
(Intercept)	8.78	7.86 – 9.70	**<.001**
person-mean negative affective states	-0.19	-0.62 – 0.24	.242
person-mean-centered negative affective states	-0.21	-0.43 – 0.02	.014
age	-0.03	-0.07 – 0.01	.069
sex [female]	-0.07	-0.34 – 0.20	.497
controlled for: study			
Random Effects			
σ^2	12.09		
$\tau_{00\ id}$	1.20		
$\tau_{11\ id.mc\_negaff}$	0.45		
$\rho_{01\ id}$	0.46		
ICC	0.09		
N_{id}	654		
Observations	26,911		

Note. Due to singularity in the three-level model (measurements nested in participants nested in studies), the third level (study) was dropped and a two-level model (measurements nested in participants) employed. We introduced study as a fixed effect control variable. As the fixed-effect matrix of the model including the PA aggregation interval as a control variable was rank deficient, the control variable was dropped.

Table S8.3: Consequent one-stage MAI IPD model valence

	PA (milli-g, square-root-transformed)		
Predictors	*Estimates*	*99.2% CI*	*p*
(Intercept)	6.41	4.97 – 7.85	**<.001**
person-mean valence	0.23	-0.06 – 0.52	.035
person-mean-centered valence	0.28	-0.04 – 0.61	.015
age	0.00	-0.02 – 0.03	.631
sex [female]	0.06	-0.18 – 0.29	.510
Random Effects			
σ^2	13.58		
$\tau_{00\ id:study}$	1.03		
$\tau_{00\ study}$	0.74		
$\tau_{11\ id:study.mc\_valence}$	0.54		
$\tau_{11\ study.mc\_valence}$	0.02		
$\rho_{01\ id:study}$	-0.20		
$\rho_{01\ study}$	-0.50		
ICC	0.12		
N_{id}	885		
N_{study}	7		
Observations	35,623		

Note. As the fixed-effect matrix of the model including the PA aggregation interval as a control variable was rank deficient, the control variable was dropped.

Table S8.4: Consequent one-stage MAI IPD model energetic arousal

	PA (milli-*g*, square-root-transformed)		
Predictors	*Estimates*	*99.2% CI*	*p*
(Intercept)	6.20	5.02 – 7.38	**<.001**
person-mean energetic arousal	0.06	-0.24 – 0.37	.579
person-mean-centered energetic arousal	1.01	0.89 – 1.14	**<.001**
age	0.00	-0.02 – 0.03	.634
sex [female]	0.06	-0.17 – 0.30	.480
controlled for: study			
Random Effects			
σ^2	13.20		
$\tau_{00\ id}$	1.01		
$\tau_{11\ id.mc\_energetic}$	0.61		
$\rho_{01\ id}$	0.29		
ICC	0.08		
N_{id}	885		
Observations	35,630		

Note. Due to singularity in the three-level model (measurements nested in participants nested in studies), the third level (study) was dropped and a two-level model (measurements nested in participants) employed. We introduced study as a fixed effect control variable. As the fixed-effect matrix of the model including the PA aggregation interval as a control variable was rank deficient, the control variable was dropped.

Table S8.5: Consequent one-stage MAI IPD model calmness

	PA (milli-*g*, square-root-transformed)		
Predictors	*Estimates*	*99.2% CI*	*p*
(Intercept)	6.47	5.28 – 7.65	**<.001**
person-mean calmness	-0.04	-0.31 – 0.24	.725
person-mean-centered calmness	-0.67	-0.84 – -0.50	**<.001**
age	0.01	-0.02 – 0.03	.519
sex [female]	0.07	-0.16 – 0.31	.406
controlled for: study			
Random Effects			
σ^2	13.32		
$\tau_{00\ id}$	1.03		
$\tau_{11\ id.mc\_calmness}$	1.41		
$\rho_{01\ id}$	-0.28		
ICC	0.09		
N_{id}	885		
Observations	35,621		

Note. Due to singularity in the three-level model (measurements nested in participants nested in studies), the third level (study) was dropped and a two-level model (measurements nested in participants) employed. We introduced study as a fixed effect control variable. As the fixed-effect matrix of the model including the PA aggregation interval as a control variable was rank deficient, the control variable was dropped.

S9: Assessing affective well-being in daily life

There is an ongoing debate on how to conceptualise, define, and operationalise affective phenomena[129–137]. Despite various efforts to standardise affective terminology (e.g.,[131,133,138]), terms such as core affect, feelings, emotions, and moods remain inconsistently used[131,133]. We use the term affective well-being (AWB) as a superordinate term that comprises all types of affective phenomena[139,140].

A prominent approach to standardise affective terminology differentiates between core affect, emotions, and mood (e.g., [133,138,141]). Russell and Barrett[138] define core affect as "the most elementary consciously accessible affective feelings". Russell[141] argues that concepts such as Thayer's[142] *activation*, Watson's and Tellegen's[143] *affect*, Morris'[144] *mood*, and the commonly used term *feeling* also tap into core affect, although they employ different terminology. Core affect is a continuous and dimensional phenomenon, which is most apparent in moods and emotions[141]. It has been modelled both as a two-dimensional (e.g.,[142,143,145]) and a three-dimensional concept (e.g.,[146–148]). For both there is empirical evidence[6,148]. Emotions, on the other hand, involve stimulus-related changes in multiple components (e.g., facial expressions, action tendencies, physiology, core affect) and cognitive processes, such as appraisal or constructionism[131,133,135]. Various emotion theories persist such as Ekman's[149] basic emotion theory, Lange's[150] network theory, Scherer's[151] appraisal theory, Moors's[152] goal-directed theory, Barrett and Russell's[153] psychological construction theories, and Mesquita and Parkinson's[154] social constructionist theories (see Scarantino[137] for an overview). Finally, definitions of mood appear to exhibit the largest heterogeneity. Russell[141] describes mood as "prolonged core affect" and a free-floating "fuzzy [concept] because neither duration nor degree of stability is defined". Ekkekakis[133] summarises that mood further involves appraisal processes, evolving around more existential topics compared to emotions. Other conceptualization of mood, however, appear similar to core affect (e.g.,[6–9,30,97,144]). Further, it needs to be noted that mood and core affect are sometimes not easily separated as changes in core affect may condense in changes in mood ratings[133].

Given the ongoing debate on basic affective terminology, several procedures for assessing AWB in daily life are currently in use: Some researchers employ self-developed items tailored to their research questions but without psychometric evaluations. Others select items from established questionnaires from cross-sectional studies, such as the Positive and Negative Affect Schedule (PANAS[85,3]; for a critical discussion of the PANAS see, e.g.,[133,155]). Both approaches commonly result in a list of affective states (e.g., content, happy, calm, excited, sad, angry, upset, anxious), which is summarised in scores for positive and negative affect. In many studies the composition of the item list varies and is not theoretically justified[156,157]. The third approach are questionnaires based on a solid theoretical framework that have demonstrated good psychometric properties in daily life[6,133,157]. To date, there is only a limited number of affective measures meeting these criteria[157], with the short-version of the MDMQ[6] being a prominent example.

To test for the robustness of our findings in the current meta-analysis against different AWB operationalizations, we draw on a theoretical framework from affective psychology[158].

Different affective phenomena, such as core affect, mood, or emotions, may be of relevance for PA in daily life. All studies identified in our literature search assessed PA continuously. Hence, all kinds of PA in everyday life were assessed. For our sensitivity analysis, we assume that the majority of physical activities in daily life (e.g., walking to the bus station) are not perceived as events but occurs incidentally without any conscious reflection on the PA involved[50,159]. Hence, we do not presume that PA in daily life elicits emotions that, per definition, are reactions to specific events or objects and comprise psychological construction[160] or appraisal[131] processes. Whether PA in daily life may be linked to core affect or mood is more difficult to theorise. As outlined above, the predominant differentiation between mood and core affect appears to lie in the time course (with mood being transient and lasting for hours, days, or weeks and core affect being a continuous phenomenon) and the presumed underlying appraisal processes of mood. Following Ekkekakis'[133] line of argument that "brief interventions (i.e., those lasting for just a few minutes) may not be long or powerful enough to change mood per se. What probably changes in such cases is core affect and it is the core affect that is reflected in self-reports of mood." (p. 51), we chose to focus our sensitivity analysis on short-term associations of PA and core affect.

We assume PA as an integral part of daily life to be associated with the global domain of core affect rather than specific affective states. Therefore, we adopt a dimensional approach to core affect instead of relying on distinct states. Both two-dimensional and three-dimensional models of core affect exist. The most prominent theoretical model of core affect are different versions of a circumplex model[142,143,145,155,161]. Russell[145] explains core affect by the two dimensions of valence and activation. Specific affective states can be arranged along those two dimensions. Watson and Tellegen[143] identified two dimensions that combine valence and activation: positive affect/activation, which ranges from low activated negative affect to high activated positive affect, and negative affect/activation, which ranges from low activated positive affect to high activated negative affect.

Three-dimensional models of core affect[146–148], on the other hand, use a different terminology than the circumplex model. Instead of referring to core affect, three-dimensional models refer to mood, which is defined as "a current inner experience and inner perception of an individual [that] is not bound to specific objects (persons, things, events, institutions) or situations and is not tied to specific, experientially evident causes"[162] and, therefore, appears to largely overlap with Russell's core affect. Three-dimensional models of core affect differentiate between the dimensions of valence or hedonic tone, tense arousal or calmness (its opposite), and energetic arousal[146,147]. Thus, they differentiate two dimensions of arousal. With regard to PA, studies adapting a three-dimensional approach, have suggested differential effects of PA on valence, calmness, and energetic arousal[5,7,37,50,80].

Direct psychometric comparisons of two-dimensional and three-dimensional models appear to favour three-dimensional models of core affect[148], at least within-persons[6]. Since we are predominantly interested in within-person associations, we therefore adopt a three-dimensional model of core affect for our sensitivity analysis.

Three-dimensional measures of core affect[146–148] exhibited good psychometric properties in cross-sectional studies. However, the psychometric quality cannot be directly transferred from cross-sectional to daily life studies[6]. Thus, Wilhelm and Schoebi[6] developed a short-version (6 items) of the Multidimensional Mood Questionnaire (MDMQ[147]) explicitly for the application in daily life studies. The short version of the MDMQ demonstrated acceptable within- and between-person reliability and sensibility to change in daily life[6]. Hence, to demonstrate robustness of findings for a narrowly defined AWB operationalization, we will jointly analyse only those studies using the short-version of the MDMQ in the following sensitivity analysis.

As a second sensitivity analysis, we adopted the PANAS conceptualization of positive and negative affect. Thus, we jointly analysed all studies that used items from the PANAS to derive scores for positive and negative activation, i.e., that used high-activation items only. A sensitivity analysis based on the circumplex model would also be desirable. However, there are only few daily life studies that directly assess the dimensions of the circumplex model to date (e.g.,[46]), making it impossible to conduct a sensitivity analysis on the dimensions of valence and activation.

S10: Two-stage Individual Participant Data meta-analysis of antecedent vs. consequent model

We statistically compared effect sizes supporting the antecedent hypothesis to effect sizes supporting the consequent hypothesis. Hence, we conducted a complimentary two-stage IPD meta-analysis considering both PA-AWB correlations and AWB-PA correlations in one model and introduced an interaction term. Post-hoc comparisons revealed no significant differences in effects between both time-orders for all PA–AWB associations when adjusting for multiple testing (.05/6 = .008).

Table S10: Two-stage Individual Participant Data meta-analysis of PA-AWB correlations vs. AWB-PA correlations

Model	Outcome	*t*	*p*
Within	positive affective states	2.16	.035
Within	negative affective states	0.76	.452
Within	valence	2.21	.032
Within	energetic arousal	1.69	.097
Within	calmness	0.29	.772
Between	positive affective states	2.16	.035
Between	negative affective states	0.94	.351
Between	valence	1.14	.261
Between	energetic arousal	2.16	.035
Between	calmness	1.02	.314

S11: Results of moderation analysis in two-stage Individual Participant Data models

In the antecedent overall model subsuming all AWB concepts into one larger category, we conducted moderator analyses as power for the individual AWB concepts would have been too low to detect meaningful effects. The following moderators were assessed:

1. PA type: We differentiated between measures of intensity (e.g., steps or MAI) and measures of time (e.g., MVPA or time stepping).
2. Age group: We divided samples into three categories, i.e., children and adolescents, young adults and older adults (age > 60) to ascertain if age moderates the relationship between PA and AWB.
3. Study location: To ascertain if there are cross-cultural differences, we used the study location as a proxy. In accordance with other meta-analyses[163], we differentiated by continental grouping. Overall, studies from Asia, North America, Europe, and Oceania were collected,
4. Position of the sensor: We used a binary classification for sensors located proximally on the body (e.g., on the hip or chest) or sensors located distally on the body (e.g., wrist or ankle).
5. Number of prompts: The absolute number of prompts per day sent to each participant was used as a moderator to assess the impact of the frequency of measurements.
6. Days of sampling: Similar to the number of prompts, it was documented over how many days the sampling took place to identify if the sampling period moderates the association between PA and AWB.
7. Short and long aggregation interval of PA: Studies varied with respect to the time interval over which PA was aggregated preceding or following an e-diary prompt. We thus used the time interval (in minutes) as continuous moderator in our analysis.

For the type of PA (time vs. intensity), we found a significant moderation effect ($F(3,61) = 25.44$, $p < .001$). Activity types classified as time (e.g., MVPA) showed weaker associations ($\beta = -0.01$, 95% CI = [-0.02;0.01]) compared to activity types classified as intensity (e.g., MAI in milli-g, $\beta = 0.05$, 95% CI = [0.04;0.07]) on the within-participant level ($t(61) = 7.87$, $p <.001$, Figure S11.1A). The position of the measurement device (proximal vs. distal) did not significantly affect the associations between PA and AWB ($F(3,60) = 1.51$, $p = .220$, Figure S11.2A). Similarly, the moderation effect of age group ($F(5,57) = 3.06$, $p = .016$, Figure S11.3A) and sampling region ($F(5,57) = 3.04$, $p = .017$, Figure S11.4A) did not reach significance using a more conservative alpha threshold of $p < .008$. For continuous moderators, neither short ($t(38) = 1.25$, $p = .220$) nor long PA intervals ($t(41) = 1.38$, $p = .175$) had any effect on associations between PA and AWB. Similarly, neither the number of prompts per day ($t(61) = 0.85$, $p = .401$) nor the days of sampling ($t(61) = 0.71$, $p = .478$) significantly moderated the association between PA and AWB. To test for risk of bias, we assessed PET- and PEESE-corrected models of the overall effect estimate by using the standard error and variance as moderators, respectively. For within-participant associations, we found no evidence of small study bias in either the PET ($F(1,63) = 0.02$, p .899) or PEESE ($F(1,63) = 0.80$, $p = .207$) analysis. For between-participant associations, the PET analysis revealed a significant negative moderation effect suggestion that effects decrease with increasing error ($F(1,62) = 508$, $p = .028$). This effect, however, did not meet the more conservative statistical threshold of $p < .008$. There was no effect for PEESE ($F(1,62) = 3.02$, $p = .087$).

We repeated the moderation analyses for the consequent model as well. For the type of PA (time vs. intensity), we again found a significant moderation effect ($F(3,33) = 17.84$, $p < .001$). Activity types classified as time (e.g., MVPA) showed weaker associations ($\beta = -0.01$, 95% CI = [-0.02;0.01]) compared to activity types classified as intensity (e.g., millig, $\beta = 0.04$, 95% CI = [0.03;0.06]) on the within-participant level ($t(33) = 6.59$, $p <.001$, Figure S11.1B). The position of the measurement device (proximal vs. distal) did not significantly affect the associations between PA and AWB ($F(3,33) = 2.44$, $p = .082$, Figure S11.2B). The moderation effect of age group ($F(5,31) = 4.45$, $p = .004$, Figure S11.3B) reached significance. Post hoc tests revealed significantly stronger within-participant associations in adults ($\beta = 0.04$, 95% CI = [0.02;0.06]) compared to older adults ($\beta = 0.00$, 95% CI = [-0.03;0.04], $p = .003$). The sampling region significantly moderated the association between PA and AWB (($F(5,29) = 8.82$, $p < .001$, Figure S11.4B). Within-participant associations in samples from Asian countries showed stronger associations ($\beta = 0.11$, 95% CI = [0.06;0.15]) compared to European ($\beta = 0.03$, 95% CI = [0.02;0.05], $p < .001$) or North American cohorts ($\beta = 0.03$, 95% CI = [-0.01;0.06] , $p < .001$). For continuous moderators, neither short ($t(21) = 1.09$, $p = .288$) nor long PA intervals ($t(28) = 0.24$, $p = .809$) had any effect on associations between PA and AWB. The same was true for the number of prompts per day ($t(33) = 1.46$, $p = .153$). However, a higher number of sampling days was linked to stronger associations between PA and AWB in the consequent model ($t(33) = 3.84$, $p < .001$). As for the antecedent model, risk of bias was assessed via PET- and PEESE-corrected models of the overall effect estimate by using the standard error and variance as moderators, respectively. We found no evidence of small study bias in either the PET ($F(1,35) = 1.86$, p .182) or PEESE ($F(1,35) = 1.46$, $p = .235$) analysis on the within-participant level. For between-participant associations, we found a negative association for PET ($F(1,35) = 5.09$, $p = .030$) that did not meet the more conservative statistical threshold. The PEESE model also did not reach significance according to this threshold ($F(1,35) = 4.51$, $p = .041$).

Figure S11.1

Forest plot illustrating the influence of PA type (intensity vs time) on overall AWB

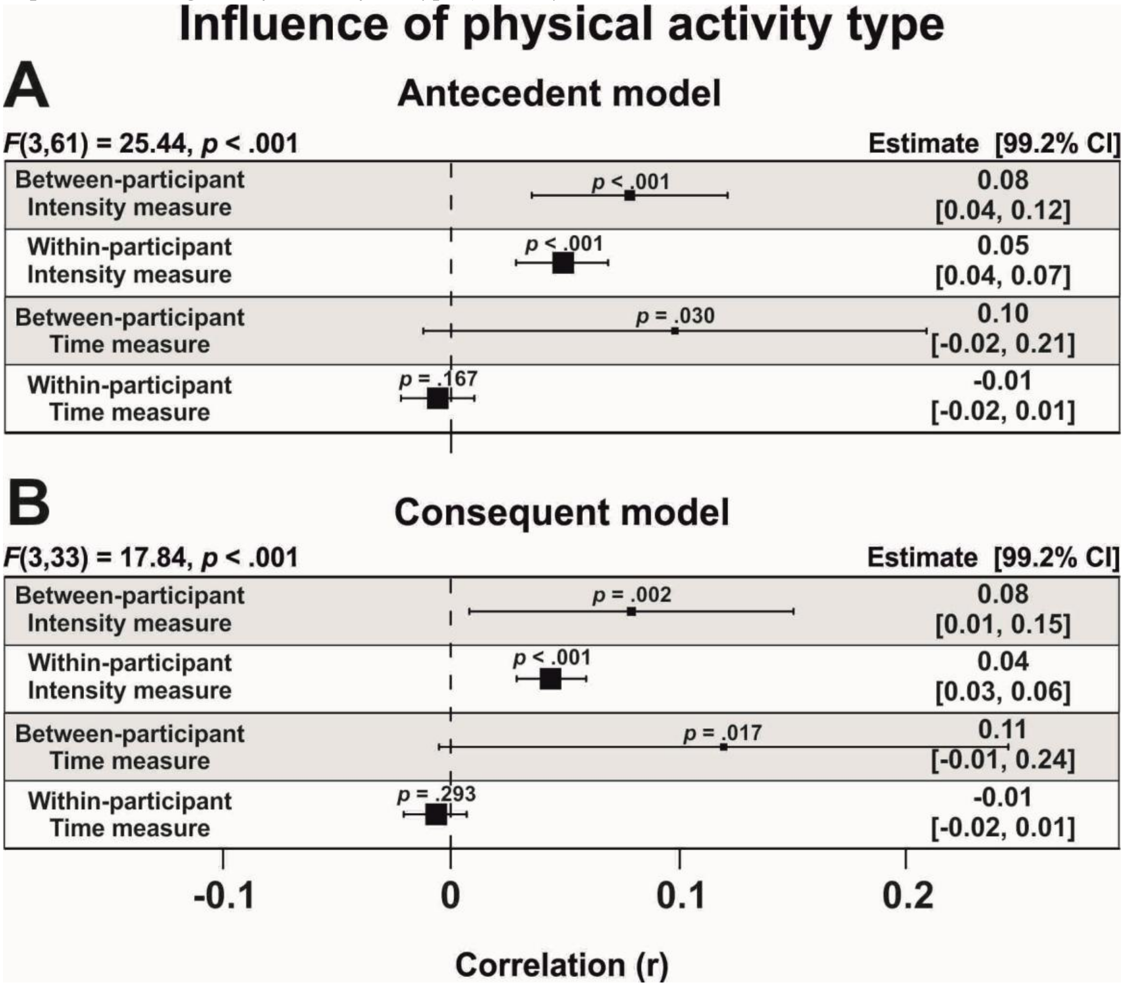

Note. PA type as a moderator reached significance for the A) antecedent and for the B) consequent model suggesting that the association between PA and AWB differs between types of PA. A total of k_{cum} = 287 dependent effects from k = 65 independent studies serve as experimental units and are included in the analysis for the antecedent model. A total of k_{cum} = 164 dependent effects from k = 37 independent studies serve as experimental units and are included in the analysis for the consequent model. Significance was assessed based on an alpha threshold of 0.8%. The whiskers represent the 99.2% confidence interval (CI). Significance for the overall moderator effect was assessed using an omnibus F-test and the significance of individual moderator levels was determined via a t-test. All statistical tests were conducted two-sided.

Figure S11.2

Forest plot illustrating the influence of sensor position (proximal vs distal) on overall AWB

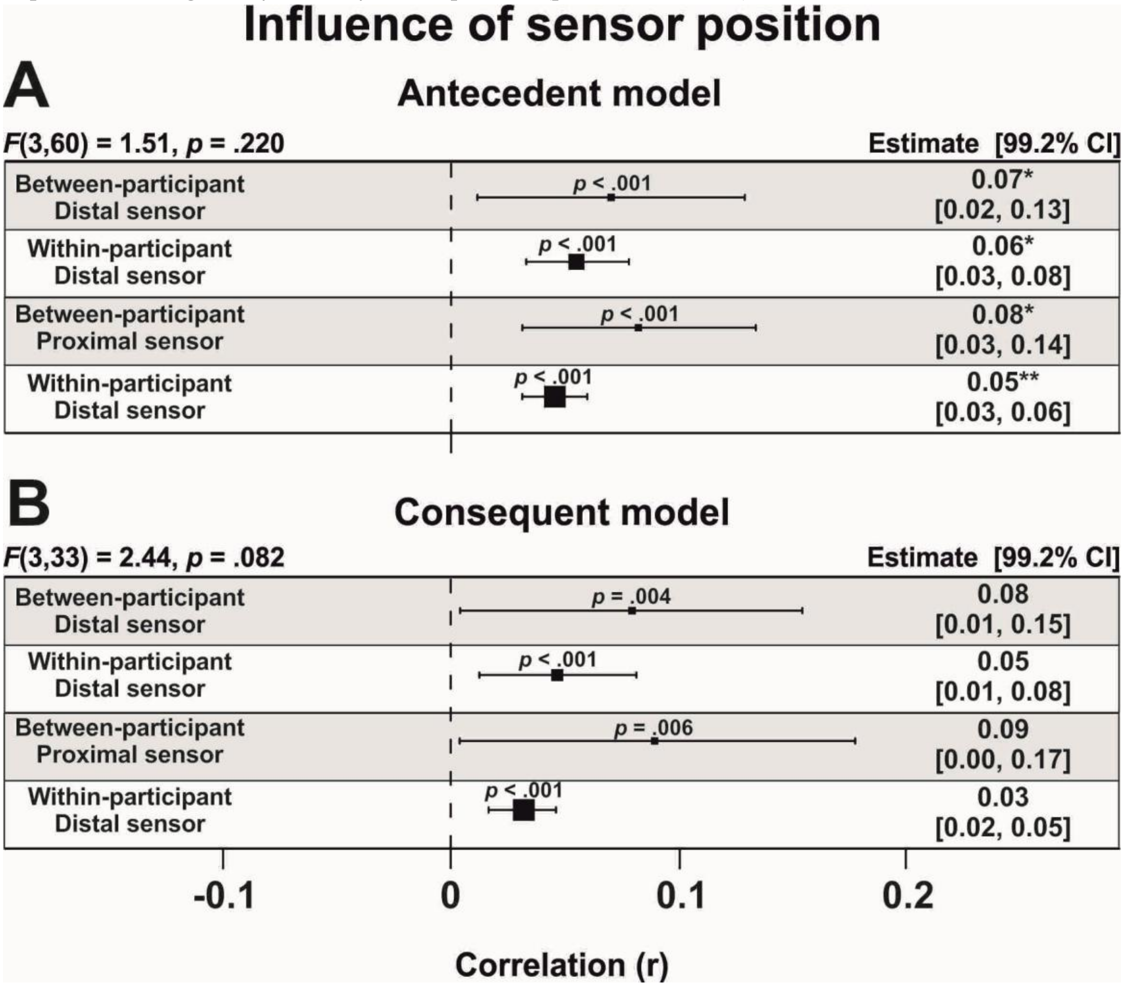

Note. Sensor position as a moderator did not reach significance for the A) antecedent nor for the B) consequent model suggesting that sensor position is not affecting the association between PA and AWB. A total of k_{cum} = 283 dependent effects from k = 64 independent studies serve as experimental units and are included in the analysis for the antecedent model. A total of k_{cum} = 164 dependent effects from k = 37 independent studies serve as experimental units and are included in the analysis for the consequent model. Significance was assessed based on an alpha threshold of 0.8%. The whiskers represent the 99.2% confidence interval (CI). Significance for the overall moderator effect was assessed using an omnibus F-test and the significance of individual moderator levels was determined via a t-test. All statistical tests were conducted two-sided.

Figure S11.3

Forest plot illustrating the influence of age group (adults vs. non-adults vs. older adults) on overall AWB

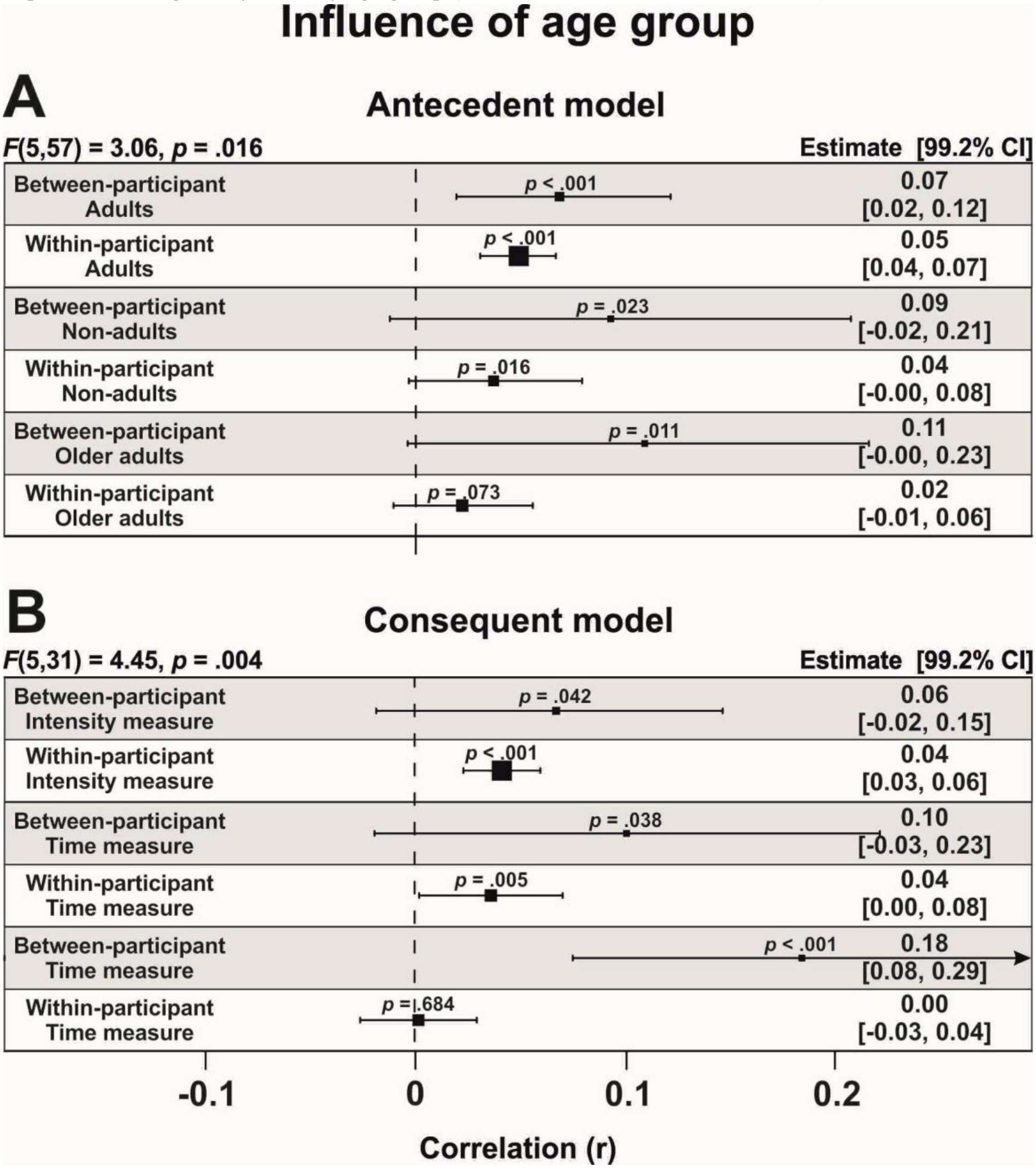

Note. Age group as a moderator did not reach significance for the A) antecedent using a more conservative significance threshold. However, it did reach significance for the B) consequent model. A total of k_{cum} = 277 dependent effects from k = 63 independent studies serve as experimental units and are included in the analysis for the antecedent model. A total of k_{cum} = 164 dependent effects from k = 37 independent studies serve as experimental units and are included in the analysis for the consequent model. Significance was assessed based on an alpha threshold of 0.8%. The whiskers represent the 99.2% confidence interval (CI). Significance for the overall moderator effect was assessed using an omnibus F-test and the significance of individual moderator levels was determined via a t-test. All statistical tests were conducted two-sided.

Figure S11.4

Forest plot illustrating the influence of sampling region (Asia vs. Europe vs. North American) on overall AWB

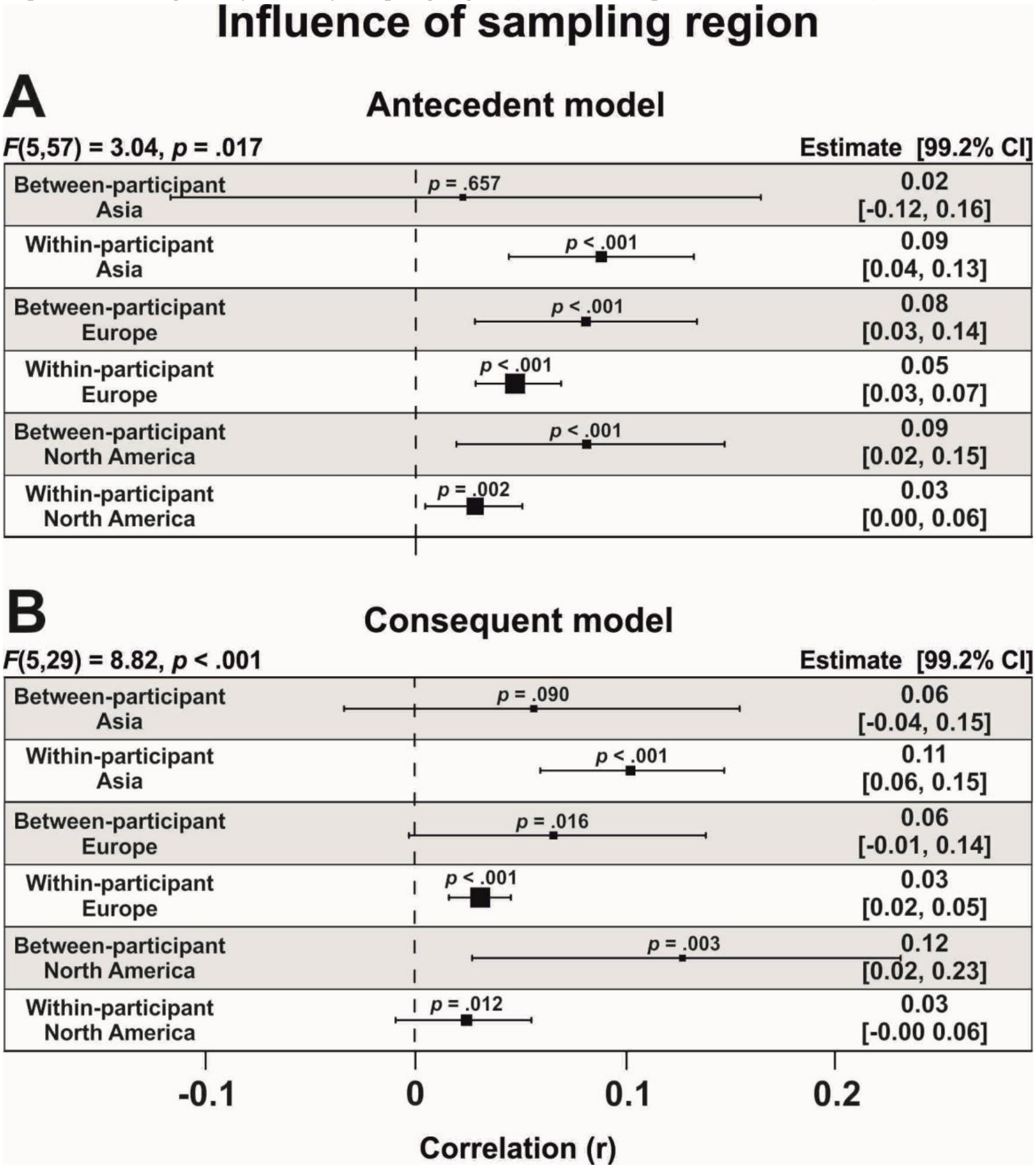

Note. Sampling region as a moderator did not reach significance for the A) antecedent but reached significance in the B) consequent model suggesting that the association between PA and AWB might be culturally dependent. A total of k_{cum} = 275 dependent effects from k = 63 independent studies serve as experimental units and are included in the analysis for the antecedent model. A total of k_{cum} = 152 dependent effects from k = 35 independent studies serve as experimental units and are included in the analysis for the consequent model. Significance was assessed based on an alpha threshold of 0.8%. The whiskers represent the 99.2% confidence interval (CI). Significance for the overall moderator effect was assessed using an omnibus F-test and the significance of individual moderator levels was determined via a t-test. All statistical tests were conducted two-sided.

S12: Results of moderation analysis in one-stage Individual Participant Data models

We investigated potential impacts of person and measurement characteristics onto the within-person associations of AWB and PA for both antecedent and consequent one-stage IPD models. Hence, we explored whether participant or measurement characteristics explained variations in effects. We set up a series of multilevel models including an interaction term of the following moderators with person-mean-centered PA (antecedent models) or AWB (consequent models) into our main one-stage IPD models (S3, S5, S7):

1. Age: Age of participants in years
2. Gender/sex: Gender (if available) or sex of participants; included categories were male, female, and non-binary
3. BMI: Body mass index of adult participants
4. Weekday vs. Weekend: Weekday of e-diary prompt; Mondays, Tuesdays, Wednesdays, Thursdays, and Fridays were coded as weekdays and Saturdays and Sundays as weekend days

Table S12.1a: Antecedent moderation analysis of age for positive affective states

	positive affective states		
Predictors	*Estimates*	*99.2% CI*	*p*
(Intercept)	2.58	2.41 – 2.74	**<.001**
person-mean PA (SD-scaled)	0.09	0.05 – 0.12	**<.001**
person-mean-centered PA (SD-scaled)	0.06	0.04 – 0.07	**<.001**
age	0.00	-0.00 – 0.00	.847
sex [female]	-0.04	-0.08 – -0.01	**.001**
sex [non-binary]	-0.06	-0.96 – 0.85	.870
person-mean-centered PA (SD-scaled) × age	-0.00	-0.00 – -0.00	**<.001**
Random Effects			
σ^2	0.25		
$\tau_{00\ id:study}$	0.22		
$\tau_{00\ study}$	0.12		
$\tau_{11\ id:study.mc\_PA\_bp\_zwm}$	0.00		
$\tau_{11\ study.mc\_PA\_bp\_zwm}$	0.00		
$\rho_{01\ id:study}$	-0.09		
$\rho_{01\ study}$	-0.38		
ICC	0.58		
N_{id}	6,159		
N_{study}	44		
Observations	225,932		

Table S12.1b: Antecedent moderation analysis of age negative affective states

	negative affective states		
Predictors	*Estimates*	*99.2% CI*	*p*
(Intercept)	1.57	1.44 – 1.69	**<.001**
person-mean PA (SD-scaled)	-0.02	-0.05 – 0.00	.023
person-mean-centered PA (SD-scaled)	-0.01	-0.02 – 0.00	.023
age	-0.00	-0.00 – -0.00	**<.001**
sex [female]	0.02	-0.00 – 0.04	.026
sex [non-binary]	0.14	-0.28 – 0.55	.387
person-mean-centered PA (SD-scaled) × age	0.00	-0.00 – 0.00	.163
Random Effects			
σ^2	0.13		
$\tau_{00\ id:study}$	0.12		
$\tau_{00\ study}$	0.07		
$\tau_{11\ id:study.mc\_PA\_bp\_zwm}$	0.00		
$\tau_{11\ study.mc\_PA\_bp\_zwm}$	0.00		
$\rho_{01\ id:study}$	-0.13		
$\rho_{01\ study}$	-0.38		
ICC	0.59		
N_{id}	6,429		
N_{study}	45		
Observations	235,537		

Table S12.1c: Antecedent moderation analysis of age valence

	valence		
Predictors	*Estimates*	*99.2% CI*	*p*
(Intercept)	3.01	2.84 – 3.18	**<.001**
person-mean PA (SD-scaled)	0.08	0.01 – 0.15	**.001**
person-mean-centered PA (SD-scaled)	0.04	0.02 – 0.06	**<.001**
age	0.00	-0.00 – 0.00	.042
sex [female]	-0.05	-0.10 – 0.00	.013
person-mean-centered PA (SD-scaled) × age	-0.00	-0.00 – 0.00	.111
Random Effects			
σ^2	0.22		
τ_{00} id:study	0.15		
τ_{00} study	0.04		
τ_{11} id:study.mc_PA_bp_zwm	0.00		
τ_{11} study.mc_PA_bp_zwm	0.00		
ρ_{01} id:study	-0.09		
ρ_{01} study	-0.91		
ICC	0.48		
N id	1,947		
N study	22		
Observations	53,274		

Table S12.1d: Antecedent moderation analysis of age energetic arousal

	energetic arousal		
Predictors	*Estimates*	*99.2% CI*	*p*
(Intercept)	2.65	2.53 – 2.78	**<.001**
person-mean PA (SD-scaled)	0.10	0.03 – 0.16	**<.001**
person-mean-centered PA (SD-scaled)	0.09	0.03 – 0.15	**<.001**
age	0.01	0.00 – 0.01	**<.001**
sex [female]	-0.12	-0.17 – -0.07	**<.001**
person-mean-centered PA (SD-scaled) × age	0.00	-0.00 – 0.00	.290
Random Effects			
σ^2	0.32		
τ_{00} id:study	0.14		
τ_{00} study	0.01		
τ_{11} id:study.mc_PA_bp_zwm	0.01		
τ_{11} study.mc_PA_bp_zwm	0.00		
ρ_{01} id:study	-0.06		
ρ_{01} study	-0.43		
ICC	0.32		
N id	1,751		
N study	19		
Observations	50,105		

Table S12.1e: Antecedent moderation analysis of age calmness

	calmness		
Predictors	*Estimates*	*99.2% CI*	*p*
(Intercept)	3.03	2.89 – 3.18	**<.001**
person-mean PA (SD-scaled)	0.02	-0.05 – 0.09	.446
person-mean-centered PA (SD-scaled)	-0.01	-0.05 – 0.03	.577
age	0.00	-0.00 – 0.00	.383
sex [female]	-0.07	-0.12 – -0.01	**.002**
person-mean-centered PA (SD-scaled) × age	-0.00	-0.00 – 0.00	.037
Random Effects			
σ^2	0.24		
$\tau_{00\ id:study}$	0.15		
$\tau_{00\ study}$	0.01		
$\tau_{11\ id:study.mc\_PA\_bp\_zwm}$	0.01		
$\tau_{11\ study.mc\_PA\_bp\_zwm}$	0.00		
$\rho_{01\ id:study}$	0.02		
$\rho_{01\ study}$	-0.14		
ICC	0.42		
N_{id}	1,641		
N_{study}	18		
Observations	48,399		

Table S12.2a: Consequent moderation analysis of age positive affective states

	PA (SD-scaled, square-root-transformed)		
Predictors	*Estimates*	*99.2% CI*	*p*
(Intercept)	0.84	0.56 – 1.11	**<.001**
person-mean positive affective states	0.04	0.03 – 0.06	**<.001**
person-mean-centered positive affective states	0.06	0.04 – 0.09	**<.001**
age	-0.00	-0.00 – -0.00	**<.001**
sex [female]	-0.01	-0.03 – 0.00	.065
person-mean-centered positive affective states × age	-0.00	-0.00 – -0.00	**.003**
Random Effects			
σ^2	0.21		
τ_{00} id:study	0.02		
τ_{00} study	0.22		
τ_{11} id:study.mc_posaff	0.01		
τ_{11} study.mc_posaff	0.00		
ρ_{01} id:study	0.13		
ρ_{01} study	-0.03		
ICC	0.53		
N id	3,864		
N study	25		
Observations	140,170		

Table S12.2b: Consequent moderation analysis of age negative affective states

	PA (SD-scaled, square-root-transformed)		
Predictors	*Estimates*	*99.2% CI*	*p*
(Intercept)	1.05	0.77 – 1.32	**<.001**
person-mean negative affective states	-0.03	-0.05 – -0.01	**<.001**
person-mean-centered negative affective states	-0.03	-0.06 – -0.00	**.005**
age	-0.00	-0.00 – -0.00	**<.001**
sex [female]	-0.01	-0.03 – 0.00	.043
sex [non-binary]	0.03	-0.22 – 0.28	.755
person-mean-centered negative affective states × age	0.00	-0.00 – 0.00	.051
Random Effects			
σ^2	0.21		
$\tau_{00\ id:study}$	0.02		
$\tau_{00\ study}$	0.24		
$\tau_{11\ id:study.mc\_negaff}$	0.01		
$\tau_{11\ study.mc\_negaff}$	0.00		
$\rho_{01\ id:study}$	-0.04		
$\rho_{01\ study}$	-0.63		
ICC	0.56		
N_{id}	4,179		
N_{study}	27		
Observations	150,118		

Table S12.2c: Consequent moderation analysis of age valence

	PA (SD-scaled, square-root-transformed)		
Predictors	*Estimates*	*99.2% CI*	*p*
(Intercept)	0.75	0.51 – 0.99	**<.001**
person-mean valence	0.03	0.01 – 0.06	**.001**
person-mean-centered valence	0.05	0.01 – 0.10	**.003**
age	-0.00	-0.00 – 0.00	.098
sex [female]	0.01	-0.02 – 0.03	.540
person-mean-centered valence × age	-0.00	-0.00 – 0.00	.089
Random Effects			
σ^2	0.16		
$\tau_{00\ id:study}$	0.02		
$\tau_{00\ study}$	0.05		
$\tau_{11\ id:study.mc\_valence}$	0.01		
$\tau_{11\ study.mc\_valence}$	0.00		
$\rho_{01\ id:study}$	-0.05		
$\rho_{01\ study}$	-0.37		
ICC	0.31		
N_{id}	1,354		
N_{study}	11		
Observations	44,251		

Table S12.2d: Consequent moderation analysis of age energetic arousal

	PA (SD-scaled, square-root-transformed)		
Predictors	*Estimates*	*99.2% CI*	*p*
(Intercept)	0.82	0.56 – 1.09	**<.001**
person-mean energetic arousal	0.01	-0.02 – 0.04	.275
person-mean-centered energetic arousal	0.10	0.05 – 0.16	**<.001**
age	-0.00	-0.00 – 0.00	.151
sex [female]	0.01	-0.02 – 0.03	.341
person-mean-centered energetic arousal × age	-0.00	-0.00 – 0.00	.640
Random Effects			
σ^2	0.15		
$\tau_{00\ id:study}$	0.01		
$\tau_{00\ study}$	0.06		
$\tau_{11\ id:study.mc\_energetic}$	0.01		
$\tau_{11\ study.mc\_energetic}$	0.00		
$\rho_{01\ id:study}$	0.25		
$\rho_{01\ study}$	-0.09		
ICC	0.33		
N_{id}	1,271		
N_{study}	10		
Observations	43,270		

Table S12.2e: Consequent moderation analysis of age calmness

	PA (SD-scaled, square-root-transformed)		
Predictors	*Estimates*	*99.2% CI*	*p*
(Intercept)	0.85	0.58 – 1.12	**<.001**
person-mean calmness	0.01	-0.02 – 0.03	.598
person-mean-centered calmness	-0.06	-0.12 – 0.00	.010
age	-0.00	-0.00 – 0.00	.988
sex [female]	0.01	-0.01 – 0.03	.204
person-mean-centered calmness × age	0.00	-0.00 – 0.00	.978
Random Effects			
σ^2	0.16		
$\tau_{00\ id:study}$	0.01		
$\tau_{00\ study}$	0.05		
$\tau_{11\ id:study.mc\_energetic}$	0.02		
$\tau_{11\ study.mc\_energetic}$	0.00		
$\rho_{01\ id:study}$	-0.20		
$\rho_{01\ study}$	0.77		
ICC	0.31		
N_{id}	1,161		
N_{study}	9		
Observations	41,561		

Table S12.3a: Antecedent moderation analysis of sex/gender for positive affective states

	positive affective states		
Predictors	*Estimates*	*99.2% CI*	*p*
(Intercept)	2.59	2.42 – 2.75	**<.001**
person-mean PA (SD-scaled)	0.09	0.05 – 0.12	**<.001**
person-mean-centered PA (SD-scaled)	0.04	0.02 – 0.05	**<.001**
age	-0.00	-0.00 – 0.00	.945
sex [female]	-0.04	-0.08 – -0.01	**<.001**
sex [non-binary]	-0.06	-0.96 – 0.84	.857
person-mean-centered PA (SD-scaled) × sex [female]	0.01	-0.00 – 0.01	.060
person-mean-centered PA (SD-scaled) × sex [non-binary]	0.34	-0.34 – 1.02	.180
Random Effects			
σ^2	0.25		
$\tau_{00\ id:study}$	0.22		
$\tau_{00\ study}$	0.12		
$\tau_{11\ id:study.mc\_PA\_bp\_zwm}$	0.00		
$\tau_{11\ study.mc\_PA\_bp\_zwm}$	0.00		
$\rho_{01\ id:study}$	-0.09		
$\rho_{01\ study}$	-0.41		
ICC	0.58		
N_{id}	6,159		
N_{study}	44		
Observations	225,932		

Table S12.3b: Antecedent moderation analysis of sex/gender negative affective states

	negative affective states		
Predictors	*Estimates*	*99.2% CI*	*p*
(Intercept)	1.56	1.44 – 1.69	**<.001**
person-mean PA (SD-scaled)	-0.02	-0.05 – 0.00	.024
person-mean-centered PA (SD-scaled)	-0.00	-0.01 – 0.00	.242
age	-0.00	-0.00 – -0.00	**<.001**
sex [female]	0.02	-0.00 – 0.05	.021
person-mean-centered PA (SD-scaled) × sex [female]	-0.00	-0.01 – 0.00	.126
**Random Effects**			
σ^2	0.13		
$\tau_{00\ id:study}$	0.12		
$\tau_{00\ study}$	0.07		
$\tau_{11\ id:study.mc\_PA\_bp\_zwm}$	0.00		
$\tau_{11\ study.mc\_PA\_bp\_zwm}$	0.00		
$\rho_{01\ id:study}$	-0.13		
$\rho_{01\ study}$	-0.40		
ICC	0.59		
N_{id}	6,424		
N_{study}	45		
Observations	235,343		

Table S12.3c: Antecedent moderation analysis of sex/gender valence

	valence		
Predictors	*Estimates*	*99.2% CI*	*p*
(Intercept)	3.02	2.85 – 3.20	**<.001**
person-mean PA (SD-scaled)	0.08	0.01 – 0.15	**.001**
person-mean-centered PA (SD-scaled)	0.02	0.01 – 0.04	**<.001**
age	0.00	-0.00 – 0.00	.084
sex [female]	-0.05	-0.10 – 0.00	.010
person-mean-centered PA (SD-scaled) × sex [female]	0.01	-0.01 – 0.02	.186
**Random Effects**			
σ^2	0.22		
$\tau_{00\ id:study}$	0.15		
$\tau_{00\ study}$	0.04		
$\tau_{11\ id:study.mc\_PA\_bp\_zwm}$	0.00		
$\tau_{11\ study.mc\_PA\_bp\_zwm}$	0.00		
$\rho_{01\ id:study}$	-0.10		
$\rho_{01\ study}$	-0.89		
ICC	0.48		
N_{id}	1,947		
N_{study}	22		
Observations	53,274		

Table S12.3d: Antecedent moderation analysis of sex/gender energetic arousal

	energetic arousal		
Predictors	*Estimates*	*99.2% CI*	*p*
(Intercept)	2.65	2.53 – 2.78	**<.001**
person-mean PA (SD-scaled)	0.10	0.03 – 0.16	**<.001**
person-mean-centered PA (SD-scaled)	0.09	0.05 – 0.13	**<.001**
age	0.01	0.00 – 0.01	**<.001**
sex [female]	-0.12	-0.17 – -0.07	**<.001**
person-mean-centered PA (SD-scaled) × sex [female]	0.03	0.00 – 0.05	**.002**
Random Effects			
σ^2	0.32		
$\tau_{00\ id:study}$	0.14		
$\tau_{00\ study}$	0.01		
$\tau_{11\ id:study.mc\_PA\_bp\_zwm}$	0.01		
$\tau_{11\ study.mc\_PA\_bp\_zwm}$	0.00		
$\rho_{01\ id:study}$	-0.07		
$\rho_{01\ study}$	-0.42		
ICC	0.32		
N_{id}	1,751		
N_{study}	19		
Observations	50,105		

Table S12.3e: Antecedent moderation analysis of sex/gender calmness

	calmness		
Predictors	*Estimates*	*99.2% CI*	*p*
(Intercept)	3.03	2.89 – 3.18	**<.001**
person-mean PA (SD-scaled)	0.02	-0.05 – 0.09	.458
person-mean-centered PA (SD-scaled)	-0.04	-0.07 – -0.01	**.002**
age	0.00	-0.00 – 0.00	.393
sex [female]	-0.07	-0.12 – -0.01	**.002**
person-mean-centered PA (SD-scaled) × sex [female]	0.00	-0.02 – 0.02	.929
Random Effects			
σ^2	0.24		
$\tau_{00\ id:study}$	0.15		
$\tau_{00\ study}$	0.01		
$\tau_{11\ id:study.mc\_PA\_bp\_zwm}$	0.01		
$\tau_{11\ study.mc\_PA\_bp\_zwm}$	0.00		
$\rho_{01\ id:study}$	0.01		
$\rho_{01\ study}$	-0.07		
ICC	0.42		
N_{id}	1,641		
N_{study}	18		
Observations	48,399		

Table S12.4a: Consequent moderation analysis of sex/gender positive affective states

	PA (SD-scaled, square-root-transformed)		
Predictors	*Estimates*	*99.2% CI*	*p*
(Intercept)	0.84	0.56 – 1.11	**<.001**
person-mean positive affective states	0.04	0.03 – 0.06	**<.001**
person-mean-centered positive affective states	0.05	0.02 – 0.08	**<.001**
age	-0.00	-0.00 – -0.00	**<.001**
sex [female]	-0.01	-0.03 – 0.00	.057
person-mean-centered positive affective states × sex [female]	-0.01	-0.03 – 0.01	.097
Random Effects			
σ^2	0.21		
$\tau_{00\ id:study}$	0.02		
$\tau_{00\ study}$	0.22		
$\tau_{11\ id:study.mc\_posaff}$	0.01		
$\tau_{11\ study.mc\_posaff}$	0.00		
$\rho_{01\ id:study}$	0.13		
$\rho_{01\ study}$	0.00		
ICC	0.53		
N_{id}	3,864		
N_{study}	25		
Observations	140,170		

Table S12.4b: Consequent moderation analysis of sex/gender negative affective states

	PA (SD-scaled, square-root-transformed)		
Predictors	*Estimates*	*99.2% CI*	*p*
(Intercept)	1.05	0.77 – 1.32	**<.001**
person-mean negative affective states	-0.03	-0.05 – -0.01	**<.001**
person-mean-centered negative affective states	-0.02	-0.05 – 0.01	.037
age	-0.00	-0.00 – -0.00	**<.001**
sex [female]	-0.01	-0.03 – 0.00	.043
person-mean-centered × sex [female]	0.01	-0.01 – 0.03	.471
Random Effects			
σ^2	0.21		
$\tau_{00\ id:study}$	0.02		
$\tau_{00\ study}$	0.24		
$\tau_{11\ id:study.mc\_negaff}$	0.01		
$\tau_{11\ study.mc\_negaff}$	0.00		
$\rho_{01\ id:study}$	-0.04		
$\rho_{01\ study}$	-0.60		
ICC	0.56		
N_{id}	4,176		
N_{study}	27		
Observations	149,994		

Table S12.4c: Consequent moderation analysis of sex/gender valence

	PA (SD-scaled, square-root-transformed)		
Predictors	*Estimates*	*99.2% CI*	*p*
(Intercept)	0.75	0.51 – 0.99	**<.001**
person-mean valence	0.03	0.01 – 0.06	**.001**
person-mean-centered valence	0.01	-0.02 – 0.04	.341
age	-0.00	-0.00 – 0.00	.093
sex [female]	0.00	-0.02 – 0.03	.579
person-mean-centered valence × sex [female]	0.03	-0.00 – 0.06	.013
Random Effects			
σ^2	0.16		
$\tau_{00\ id:study}$	0.02		
$\tau_{00\ study}$	0.05		
$\tau_{11\ id:study.mc\_valence}$	0.01		
$\tau_{11\ study.mc\_valence}$	0.00		
$\rho_{01\ id:study}$	-0.06		
$\rho_{01\ study}$	0.16		
ICC	0.31		
N_{id}	1,354		
N_{study}	11		
Observations	44,251		

Table S12.4d: Consequent moderation analysis of sex/gender energetic arousal

	PA (SD-scaled, square-root-transformed)		
Predictors	*Estimates*	*99.2% CI*	*p*
(Intercept)	0.82	0.55 – 1.09	**<.001**
person-mean energetic arousal	0.01	-0.02 – 0.04	.275
person-mean-centered energetic arousal	0.09	0.05 – 0.13	**<.001**
age	-0.00	-0.00 – 0.00	.162
sex [female]	0.01	-0.01 – 0.03	.334
person-mean-centered energetic arousal × sex [female]	0.00	-0.02 – 0.03	.822
Random Effects			
σ^2	0.15		
$\tau_{00\ \mathrm{id:study}}$	0.01		
$\tau_{00\ \mathrm{study}}$	0.06		
$\tau_{11\ \mathrm{id:study.mc\_energetic}}$	0.01		
$\tau_{11\ \mathrm{study.mc\_energetic}}$	0.00		
$\rho_{01\ \mathrm{id:study}}$	0.25		
$\rho_{01\ \mathrm{study}}$	-0.03		
ICC	0.33		
N_{id}	1,271		
N_{study}	10		
Observations	43,270		

Table S12.4e: Consequent moderation analysis of sex/gender calmness

	PA (SD-scaled, square-root-transformed)		
Predictors	*Estimates*	*99.2% CI*	*p*
(Intercept)	0.85	0.58 – 1.13	**<.001**
person-mean calmness	0.01	-0.02 – 0.03	.603
person-mean-centered calmness	-0.09	-0.13 – -0.04	**<.001**
age	-0.00	-0.00 – 0.00	.995
sex [female]	0.01	-0.02 – 0.03	.339
person-mean-centered calmnness × sex [female]	0.04	0.01 – 0.08	**<.001**
Random Effects			
σ^2	0.16		
$\tau_{00\ id:study}$	0.01		
$\tau_{00\ study}$	0.05		
$\tau_{11\ id:study.mc\_energetic}$	0.02		
$\tau_{11\ study.mc\_energetic}$	0.00		
$\rho_{01\ id:study}$	-0.21		
$\rho_{01\ study}$	0.81		
ICC	0.31		
N_{id}	1,161		
N_{study}	9		
Observations	41,561		

Table S12.5a: Antecedent moderation analysis of Body Mass Index for positive affective states

	positive affective states		
Predictors	*Estimates*	*99.2% CI*	*p*
(Intercept)	2.43	2.23 – 2.64	**<.001**
person-mean PA (SD-scaled)	0.08	0.03 – 0.13	**<.001**
person-mean-centered PA (SD-scaled)	0.03	0.00 – 0.06	**.006**
age	0.00	0.00 – 0.01	**<.001**
sex [female]	-0.04	-0.08 – -0.00	**.007**
BMI of adults	0.00	-0.00 – 0.01	.436
person-mean-centered PA (SD-scaled) × BMI of adults	0.00	-0.00 – 0.00	.863
Random Effects			
σ^2	0.24		
$\tau_{00\ id:study}$	0.17		
$\tau_{00\ study}$	0.07		
$\tau_{11\ id:study.mc\_PA\_bp\_zwm}$	0.00		
$\tau_{11\ study.mc\_PA\_bp\_zwm}$	0.00		
$\rho_{01\ id:study}$	-0.10		
$\rho_{01\ study}$	-0.32		
ICC	0.51		
N_{id}	3,479		
N_{study}	24		
Observations	136,737		

Table S12.5b: Antecedent moderation analysis of Body Mass Index negative affective states

	negative affective states		
Predictors	*Estimates*	*99.2% CI*	*p*
(Intercept)	1.64	1.48 – 1.81	**<.001**
person-mean PA (SD-scaled)	-0.02	-0.06 – 0.02	.195
person-mean-centered PA (SD-scaled)	-0.01	-0.03 – 0.01	.067
age	-0.00	-0.01 – -0.00	**<.001**
sex [female]	-0.00	-0.03 – 0.03	.964
BMI of adults	-0.00	-0.00 – 0.00	.214
person-mean-centered PA (SD-scaled) × BMI of adults	0.00	-0.00 – 0.00	.253
Random Effects			
σ^2	0.13		
$\tau_{00\ id:study}$	0.10		
$\tau_{00\ study}$	0.05		
$\tau_{11\ id:study.mc\_PA\_bp\_zwm}$	0.00		
$\tau_{11\ study.mc\_PA\_bp\_zwm}$	0.00		
$\rho_{01\ id:study}$	-0.22		
$\rho_{01\ study}$	-0.26		
ICC	0.53		
N_{id}	3,495		
N_{study}	24		
Observations	134,951		

Table S12.5c: Antecedent moderation analysis of Body Mass Index valence

	valence		
Predictors	*Estimates*	*99.2% CI*	*p*
(Intercept)	2.93	2.70 – 3.16	**<.001**
person-mean PA (SD-scaled)	0.06	-0.03 – 0.15	.067
person-mean-centered PA (SD-scaled)	0.07	0.03 – 0.11	**<.001**
age	0.00	-0.00 – 0.01	**.008**
sex [female]	-0.04	-0.10 – 0.03	.141
BMI of adults	0.00	-0.01 – 0.01	.799
person-mean-centered PA (SD-scaled) × BMI of adults	-0.00	-0.00 – -0.00	**.004**
controlled for: study			
Random Effects			
σ^2	0.22		
$\tau_{00\ id}$	0.15		
$\tau_{11\ id.mc\_PA\_bp\_zwm}$	0.00		
$\rho_{01\ id}$	-0.07		
ICC	0.41		
N $_{id}$	1,368		
Observations	43,833		

Note. Due to singularity in the three-level model (measurements nested in participants nested in studies), the third level (study) was dropped and a two-level model (measurements nested in participants) employed. We introduced study as a fixed effect control variable.

Table S12.5d: Antecedent moderation analysis of Body Mass Index energetic arousal

	energetic arousal		
Predictors	*Estimates*	*99.2% CI*	*p*
(Intercept)	2.60	2.37 – 2.83	**<.001**
person-mean PA (SD-scaled)	0.08	-0.02 – 0.18	.026
person-mean-centered PA (SD-scaled)	0.05	-0.03 – 0.13	.093
age	0.01	0.00 – 0.01	**<.001**
sex [female]	-0.11	-0.17 – -0.04	**<.001**
BMI of adults	0.00	-0.00 – 0.01	.394
person-mean-centered PA (SD-scaled) × BMI of adults	0.00	-0.00 – 0.00	.131
Random Effects			
σ^2	0.33		
$\tau_{00\ id:study}$	0.13		
$\tau_{00\ study}$	0.01		
$\tau_{11\ id:study.mc\_PA\_bp\_zwm}$	0.01		
$\tau_{11\ study.mc\_PA\_bp\_zwm}$	0.00		
$\rho_{01\ id:study}$	-0.10		
$\rho_{01\ study}$	-0.08		
ICC	0.31		
N_{id}	1,214		
N_{study}	14		
Observations	41,043		

Table S12.5e: Antecedent moderation analysis of Body Mass Index calmness

	calmness		
Predictors	*Estimates*	*99.2% CI*	*p*
(Intercept)	2.88	2.60 – 3.16	**<.001**
person-mean PA (SD-scaled)	-0.01	-0.12 – 0.10	.794
person-mean-centered PA (SD-scaled)	0.02	-0.06 – 0.10	.526
age	0.00	-0.00 – 0.01	.082
sex [female]	-0.05	-0.12 – 0.02	.052
BMI of adults	0.00	-0.00 – 0.01	.184
person-mean-centered PA (SD-scaled) × BMI of adults	-0.00	-0.01 – 0.00	.047
Random Effects			
σ^2	0.24		
τ_{00} id:study	0.16		
τ_{00} study	0.01		
τ_{11} id:study.mc_PA_bp_zwm	0.01		
τ_{11} study.mc_PA_bp_zwm	0.00		
ρ_{01} id:study	0.04		
ρ_{01} study	-0.41		
ICC	0.42		
N id	1,106		
N study	13		
Observations	39,371		

Table S12.6a: Consequent moderation analysis of Body Mass Index positive affective states

	PA (SD-scaled, square-root-transformed)		
Predictors	*Estimates*	*99.2% CI*	*p*
(Intercept)	0.66	0.39 – 0.93	**<.001**
person-mean positive affective states	0.03	0.01 – 0.05	**<.001**
person-mean-centered positive affective states	0.07	0.01 – 0.13	**.003**
age	0.00	0.00 – 0.00	**<.001**
sex [female]	0.00	-0.01 – 0.02	.467
BMI of adults	-0.00	-0.00 – -0.00	.045
person-mean-centered positive affective states × BMI of adults	-0.00	-0.00 – -0.00	.115
Random Effects			
σ^2	0.23		
τ_{00} id:study	0.02		
τ_{00} study	0.11		
τ_{11} id:study.mc_posaff	0.01		
τ_{11} study.mc_posaff	0.00		
ρ_{01} id:study	0.10		
ρ_{01} study	-0.13		
ICC	0.36		
N id	2,610		
N study	15		
Observations	103,791		

Table S12.6b: Consequent moderation analysis of Body Mass Index negative affective states

	PA (SD-scaled, square-root-transformed)		
Predictors	*Estimates*	*99.2% CI*	*p*
(Intercept)	0.86	0.53 – 1.19	**<.001**
person-mean negative affective states	-0.02	-0.05 – 0.00	.022
person-mean-centered negative affective states	-0.06	-0.13 – 0.01	.023
age	0.00	0.00 – 0.00	**<.001**
sex [female]	0.00	-0.02 – 0.02	.698
BMI of adults	-0.00	-0.00 – 0.00	.069
person-mean-centered negative affective states × BMI of adults	0.00	-0.00 – 0.00	.052
Random Effects			
σ^2	0.23		
$\tau_{00\ id:study}$	0.02		
$\tau_{00\ study}$	0.18		
$\tau_{11\ id:study.mc\_negaff}$	0.01		
$\tau_{11\ study.mc\_negaff}$	0.00		
$\rho_{01\ id:study}$	-0.10		
$\rho_{01\ study}$	-0.59		
ICC	0.47		
N_{id}	2,671		
N_{study}	16		
Observations	102,334		

Table S12.6c: Consequent moderation analysis of Body Mass Index valence

	**PA (SD-scaled, square-root-transformed)**		
Predictors	*Estimates*	*99.2% CI*	*p*
(Intercept)	0.81	0.65 – 0.96	**<.001**
person-mean valence	0.03	-0.00 – 0.06	.009
person-mean-centered valence	0.02	-0.05 – 0.09	.388
age	-0.00	-0.00 – 0.00	.446
sex [female]	0.01	-0.02 – 0.03	.557
BMI of adults	-0.00	-0.01 – -0.00	**.002**
person-mean-centered valence × BMI of adults	-0.00	-0.00 – 0.00	.968
controlled for: study			
Random Effects			
σ^2	0.16		
$\tau_{00\ id}$	0.01		
$\tau_{11\ id.mc\_valence}$	0.01		
$\rho_{01\ id}$	-0.04		
ICC	0.09		
N_{id}	1,056		
Observations	37,887		

Note. Due to singularity in the three-level model (measurements nested in participants nested in studies), the third level (study) was dropped and a two-level model (measurements nested in participants) employed. We introduced study as a fixed effect control variable.

Table S12.6d: Consequent moderation analysis of Body Mass Index energetic arousal

	PA (SD-scaled, square-root-transformed)		
Predictors	*Estimates*	*99.2% CI*	*p*
(Intercept)	0.82	0.66 – 0.98	**<.001**
person-mean energetic arousal	0.01	-0.02 – 0.04	.296
person-mean-centered energetic arousal	0.12	0.04 – 0.20	**<.001**
age	-0.00	-0.00 – 0.00	.577
sex [female]	0.01	-0.02 – 0.03	.409
BMI of adults	-0.00	-0.01 – -0.00	**.001**
person-mean-centered energetic arousal × BMI of adults	-0.00	-0.00 – 0.00	.405
Random Effects			
σ^2	0.16		
$\tau_{00\ id:study}$	0.01		
$\tau_{00\ study}$	0.01		
$\tau_{11\ id:study.mc\_energetic}$	0.01		
$\tau_{11\ study.mc\_energetic}$	0.00		
$\rho_{01\ id:study}$	0.20		
$\rho_{01\ study}$	0.83		
ICC	0.16		
N_{id}	973		
N_{study}	9		
Observations	36,904		

Table S12.6e: Consequent moderation analysis of Body Mass Index calmness

	PA (SD-scaled, square-root-transformed)		
Predictors	*Estimates*	*99.2% CI*	*p*
(Intercept)	0.79	0.65 – 0.92	**<.001**
person-mean calmness	0.01	-0.02 – 0.04	.234
person-mean-centered calmness	-0.13	-0.21 – -0.05	**<.001**
age	0.00	-0.00 – 0.00	.381
sex [female]	0.02	-0.01 – 0.04	.130
BMI of adults	-0.00	-0.01 – 0.00	.022
person-mean-centered calmness × BMI of adults	0.00	-0.00 – 0.01	.034
controlled for: study			
Random Effects			
σ^2	0.16		
$\tau_{00\ id}$	0.01		
$\tau_{11\ id.mc\_calmness}$	0.02		
$\rho_{01\ id}$	-0.16		
ICC	0.09		
N_{id}	865		
Observations	35,228		

Note. Due to singularity in the three-level model (measurements nested in participants nested in studies), the third level (study) was dropped and a two-level model (measurements nested in participants) employed. We introduced study as a fixed effect control variable.

Table S12.7a: Antecedent moderation analysis of weekday vs. weekend for positive affective states

	positive affective states		
Predictors	*Estimates*	*99.2% CI*	*p*
(Intercept)	2.65	2.48 – 2.82	**<.001**
person-mean PA (SD-scaled)	0.08	0.04 – 0.12	**<.001**
person-mean-centered PA (SD-scaled)	0.04	0.03 – 0.06	**<.001**
age	0.00	-0.00 – 0.00	.903
sex [female]	-0.06	-0.09 – -0.02	**<.001**
sex [non-binary]	-0.06	-0.96 – 0.83	.849
weekday [weekday]	-0.07	-0.08 – -0.07	**<.001**
person-mean-centered PA (SD-scaled) × weekday [weekday]	-0.00	-0.01 – 0.00	.321
Random Effects			
σ^2	0.25		
$\tau_{00\ id:study}$	0.21		
$\tau_{00\ study}$	0.12		
$\tau_{11\ id:study.mc\_PA\_bp\_zwm}$	0.00		
$\tau_{11\ study.mc\_PA\_bp\_zwm}$	0.00		
$\rho_{01\ id:study}$	-0.10		
$\rho_{01\ study}$	-0.49		
ICC	0.57		
N_{id}	5,757		
N_{study}	42		
Observations	212,265		

Table S12.7b: Antecedent moderation analysis of weekday vs. weekend negative affective states

	negative affective states		
Predictors	*Estimates*	*99.2% CI*	*p*
(Intercept)	1.54	1.41 – 1.67	**<.001**
person-mean PA (SD-scaled)	-0.02	-0.05 – 0.00	.025
person-mean-centered PA (SD-scaled)	-0.00	-0.01 – 0.01	.331
age	-0.00	-0.00 – -0.00	**<.001**
sex [female]	0.02	-0.01 – 0.04	.056
sex [non-binary]	0.13	-0.28 – 0.55	.393
weekday [weekday]	0.05	0.05 – 0.06	**<.001**
person-mean-centered PA (SD-scaled) × weekday [weekday]	-0.00	-0.01 – 0.00	.026
Random Effects			
σ^2	0.13		
$\tau_{00\ id:study}$	0.12		
$\tau_{00\ study}$	0.07		
$\tau_{11\ id:study.mc\_PA\_bp\_zwm}$	0.00		
$\tau_{11\ study.mc\_PA\_bp\_zwm}$	0.00		
$\rho_{01\ id:study}$	-0.15		
$\rho_{01\ study}$	-0.38		
ICC	0.59		
N_{id}	6,027		
N_{study}	43		
Observations	221,872		

Table S12.7c: Antecedent moderation analysis of weekday vs. weekend valence

	valence		
Predictors	*Estimates*	*99.2% CI*	*p*
(Intercept)	3.07	2.90 – 3.24	**<.001**
person-mean PA (SD-scaled)	0.08	0.02 – 0.15	**.001**
person-mean-centered PA (SD-scaled)	0.03	0.01 – 0.04	**<.001**
age	0.00	-0.00 – 0.00	.092
sex [female]	-0.05	-0.10 – 0.00	.012
weekday [weekday]	-0.06	-0.08 – -0.05	**<.001**
person-mean-centered PA (SD-scaled) × weekday [weekday]	0.00	-0.01 – 0.01	.985
Random Effects			
σ^2	0.21		
$\tau_{00\ id:study}$	0.15		
$\tau_{00\ study}$	0.04		
$\tau_{11\ id:study.mc\_PA\_bp\_zwm}$	0.00		
$\tau_{11\ study.mc\_PA\_bp\_zwm}$	0.00		
$\rho_{01\ id:study}$	-0.10		
$\rho_{01\ study}$	-0.92		
ICC	0.48		
N_{id}	1,947		
N_{study}	22		
Observations	53,274		

Table S12.7d: Antecedent moderation analysis of weekday vs. weekend energetic arousal

	energetic arousal		
Predictors	*Estimates*	*99.2% CI*	*p*
(Intercept)	2.67	2.55 – 2.80	**<.001**
person-mean PA (SD-scaled)	0.09	0.03 – 0.16	**<.001**
person-mean-centered PA (SD-scaled)	0.12	0.08 – 0.16	**<.001**
age	0.01	0.00 – 0.01	**<.001**
sex [female]	-0.12	-0.17 – -0.07	**<.001**
weekday [weekday]	-0.03	-0.04 – -0.01	**<.001**
person-mean-centered PA (SD-scaled) × weekday [weekday]	-0.02	-0.04 – -0.00	**.001**
Random Effects			
σ^2	0.32		
$\tau_{00\ id:study}$	0.14		
$\tau_{00\ study}$	0.01		
$\tau_{11\ id:study.mc\_PA\_bp\_zwm}$	0.01		
$\tau_{11\ study.mc\_PA\_bp\_zwm}$	0.00		
$\rho_{01\ id:study}$	-0.06		
$\rho_{01\ study}$	-0.42		
ICC	0.32		
N_{id}	1,751		
N_{study}	19		
Observations	50,105		

Table S12.7e: Antecedent moderation analysis of weekday vs. weekend calmness

	calmness		
Predictors	*Estimates*	*99.2% CI*	*p*
(Intercept)	3.09	2.94 – 3.24	**<.001**
person-mean PA (SD-scaled)	0.02	-0.05 – 0.09	.478
person-mean-centered PA (SD-scaled)	-0.04	-0.07 – -0.01	**.002**
age	0.00	-0.00 – 0.00	.392
sex [female]	-0.07	-0.12 – -0.01	**.001**
weekday [weekday]	-0.08	-0.09 – -0.06	**<.001**
person-mean-centered PA (SD-scaled) × weekday [weekday]	0.00	-0.01 – 0.02	.617
Random Effects			
σ^2	0.23		
$\tau_{00\ id:study}$	0.15		
$\tau_{00\ study}$	0.01		
$\tau_{11\ id:study.mc\_PA\_bp\_zwm}$	0.01		
$\tau_{11\ study.mc\_PA\_bp\_zwm}$	0.00		
$\rho_{01\ id:study}$	0.02		
$\rho_{01\ study}$	-0.05		
ICC	0.42		
N_{id}	1,641		
N_{study}	18		
Observations	48,399		

Table S12.8a: Consequent moderation analysis of weekday vs. weekend positive affective states

	PA (SD-scaled, square-root-transformed)		
Predictors	*Estimates*	*99.2% CI*	*p*
(Intercept)	0.82	0.54 – 1.11	**<.001**
person-mean positive affective states	0.04	0.02 – 0.05	**<.001**
person-mean-centered positive affective states	0.05	0.02 – 0.07	**<.001**
age	-0.00	-0.00 – -0.00	**.004**
sex [female]	-0.01	-0.03 – 0.00	.029
weekday [weekday]	-0.01	-0.01 – 0.00	.018
person-mean-centered positive affective states × weekday [weekday]	-0.00	-0.02 – 0.01	.747
Random Effects			
σ^2	0.22		
$\tau_{00\ id:study}$	0.02		
$\tau_{00\ study}$	0.23		
$\tau_{11\ id:study.mc\_posaff}$	0.01		
$\tau_{11\ study.mc\_posaff}$	0.00		
$\rho_{01\ id:study}$	0.15		
$\rho_{01\ study}$	0.06		
ICC	0.53		
N_{id}	3,721		
N_{study}	24		
Observations	137,665		

Table S12.8b: Consequent moderation analysis of weekday vs. weekend negative affective states

	PA (SD-scaled, square-root-transformed)		
Predictors	*Estimates*	*99.2% CI*	*p*
(Intercept)	1.03	0.75 – 1.32	**<.001**
person-mean negative affective states	-0.03	-0.05 – -0.01	**<.001**
person-mean-centered negative affective states	-0.01	-0.04 – 0.02	.252
age	-0.00	-0.00 – -0.00	**.002**
sex [female]	-0.01	-0.03 – 0.00	.018
sex [non-binary]	0.03	-0.22 – 0.27	.769
weekday [weekday]	-0.01	-0.01 – -0.00	**.003**
person-mean-centered negative affective states × weekday [weekday]	-0.01	-0.03 – 0.01	.288
Random Effects			
σ^2	0.21		
$\tau_{00\ id:study}$	0.02		
$\tau_{00\ study}$	0.25		
$\tau_{11\ id:study.mc\_negaff}$	0.01		
$\tau_{11\ study.mc\_negaff}$	0.00		
$\rho_{01\ id:study}$	-0.07		
$\rho_{01\ study}$	-0.60		
ICC	0.56		
N_{id}	4,034		
N_{study}	26		
Observations	147,606		

Table S12.8c: Consequent moderation analysis of weekday vs. weekend valence

	PA (SD-scaled, square-root-transformed)		
Predictors	*Estimates*	*99.2% CI*	*p*
(Intercept)	0.76	0.52 – 1.00	**<.001**
person-mean valence	0.03	0.01 – 0.06	**.001**
person-mean-centered valence	0.03	0.00 – 0.06	**.004**
age	-0.00	-0.00 – 0.00	.092
sex [female]	0.01	-0.02 – 0.03	.549
weekday [weekday]	-0.01	-0.02 – 0.00	.012
person-mean-centered valence × weekday [weekday]	-0.00	-0.03 – 0.02	.686
Random Effects			
σ^2	0.16		
$\tau_{00\ id:study}$	0.02		
$\tau_{00\ study}$	0.05		
$\tau_{11\ id:study.mc\_valence}$	0.01		
$\tau_{11\ study.mc\_valence}$	0.00		
$\rho_{01\ id:study}$	-0.05		
$\rho_{01\ study}$	-0.04		
ICC	0.30		
N_{id}	1,354		
N_{study}	11		
Observations	44,250		

Table S12.8d: Consequent moderation analysis of weekday vs. weekend energetic arousal

	PA (SD-scaled, square-root-transformed)		
Predictors	*Estimates*	*99.2% CI*	*p*
(Intercept)	0.83	0.56 – 1.09	**<.001**
person-mean energetic arousal	0.01	-0.02 – 0.04	.280
person-mean-centered energetic arousal	0.12	0.08 – 0.16	**<.001**
age	-0.00	-0.00 – 0.00	.164
sex [female]	0.01	-0.02 – 0.03	.364
weekday [weekday]	-0.01	-0.02 – 0.00	.054
person-mean-centered energetic arousal × weekday [weekday]	-0.03	-0.05 – -0.01	**<.001**
Random Effects			
σ^2	0.15		
$\tau_{00\ id:study}$	0.01		
$\tau_{00\ study}$	0.06		
$\tau_{11\ id:study.mc\_energetic}$	0.01		
$\tau_{11\ study.mc\_energetic}$	0.00		
$\rho_{01\ id:study}$	0.25		
$\rho_{01\ study}$	-0.10		
ICC	0.33		
N $_{id}$	1,271		
N $_{study}$	10		
Observations	43,269		

Table S12.8e: Consequent moderation analysis of weekday vs. weekend calmness

	PA (SD-scaled, square-root-transformed)		
Predictors	*Estimates*	*99.2% CI*	*p*
(Intercept)	0.86	0.59 – 1.13	<.001
person-mean calmness	0.01	-0.02 – 0.03	.603
person-mean-centered calmness	-0.06	-0.10 – -0.02	<.001
age	-0.00	-0.00 – 0.00	.982
sex [female]	0.01	-0.01 – 0.03	.212
weekday [weekday]	-0.01	-0.03 – -0.00	.002
person-mean-centered calmness × weekday [weekday]	0.01	-0.02 – 0.03	.544
Random Effects			
σ^2	0.16		
$\tau_{00\ id:study}$	0.01		
$\tau_{00\ study}$	0.05		
$\tau_{11\ id:study.mc\_energetic}$	0.02		
$\tau_{11\ study.mc\_energetic}$	0.00		
$\rho_{01\ id:study}$	-0.20		
$\rho_{01\ study}$	0.80		
ICC	0.31		
N_{id}	1,161		
N_{study}	9		
Observations	41,560		

S13: Low Base effects in one-stage Individual Participant Data models

We found considerable differences in the direction and strength of PA-AWB associations between individuals. To gain more insights into this heterogeneity, we visualised individual intercepts and slopes using Scatter-Plots and explored potential low-base effects (i.e., steeper PA-AWB slopes for individuals with on average low AWB/little PA; Figure S13.1, Figure S13.2). To test for these potential low-base effects statistically, we inspected the random effects correlations of the multilevel models (i.e., the correlation of individual intercepts and slopes; Table S13.1, Table S13.2)

Figure S13.1

Scatter plots of individual intercepts and slopes from antecedent one-stage IPD models

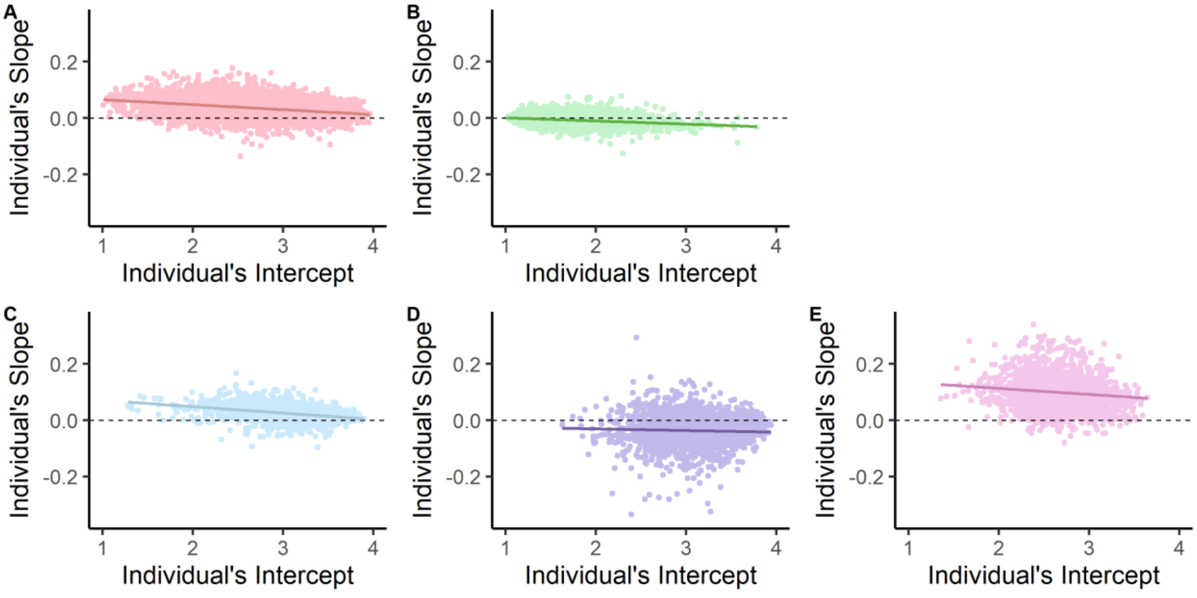

Note. n indicates the number of participants and *i* the number of ratings. Scatter plots depicting the association of participants' random intercepts and slopes drawn from the multilevel model predicting A) positive affective states (*n* = 6,159, *i* = 225,932), B) negative affective states (*n* = 6,429, *i* = 235,537), C) valence (*n* = 1,947, *i* = 53,274), D) energetic arousal (*n* = 1,751, *i* = 50,105), and E) calmness (*n* = 1,641, *i* = 48,399) based on preceding physical activity (controlled for gender/sex and age). We depicted individual intercepts of participants on the x-axis and their slopes on the y-axis.

Table S13.1: Random effects correlations and 99.2% confidence intervals in one-stage IPD antecedent models

	Random Effects Correlation	
Outcome	*Estimates*	*99.2% CI*
positive affective states	-.09	-.17 – -.01
negative affective states	-.13	-.23 – -.04
valence	-.10	-.25 – .06
energetic arousal	-.06	-.20 – .08
calmness	.01	-.10 – .13

Figure S13.2

Scatter plots of individual intercepts and slopes from consequent one-stage IPD models

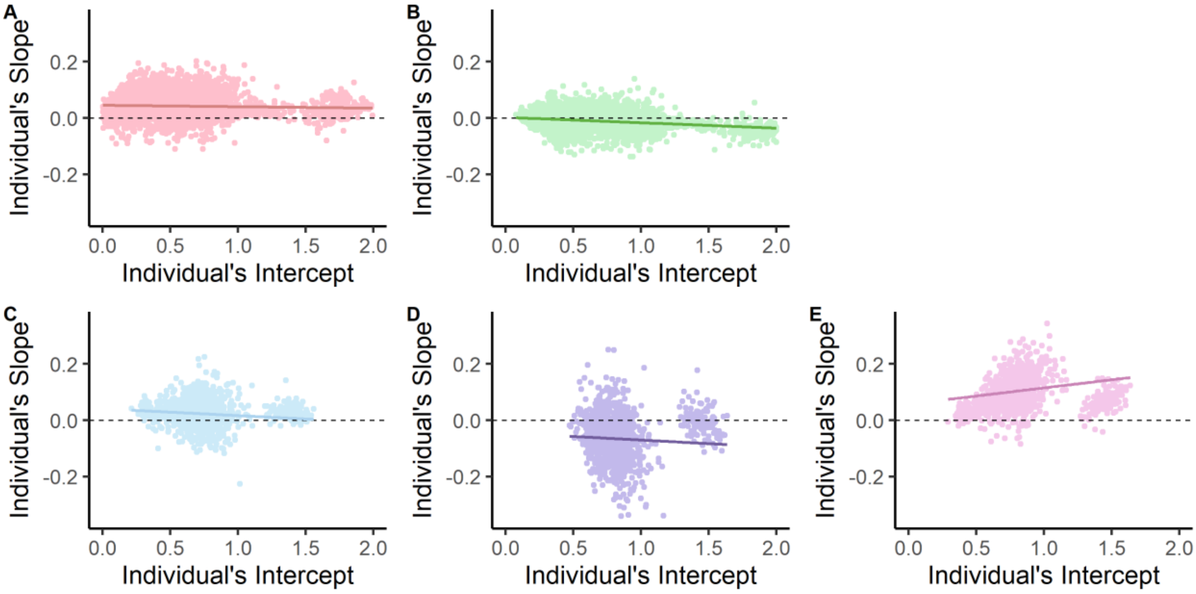

Note. n indicates the number of participants and *i* the number of ratings. Scatter plots depicting the association of participants' random intercepts and slopes drawn from the multilevel model predicting PA based on preceding A) positive affective states (*n* = 3,864, *i* = 140,170), B) negative affective states (*n* = 4,179, *i* = 150,118), C) valence (*n* = 1,354, *i* = 44,251), D) energetic arousal (*n* = 1,271, *i* = 43,270), and E) calmness (*n* = 1,161, *i* = 41,561) (controlled for gender/sex and age). We depicted individual intercepts of participants on the x-axis and their slopes on the y-axis.

Table S13.2: Random effects correlations and 99.2% confidence intervals in one-stage IPD consequent models

	Random Effects Correlation	
Predictor	*Estimates*	*99.2% CI*
positive affective states	.13	.01 – .26
negative affective states	-.04	-.20 – .11
valence	-.06	-.25 – .14
energetic arousal	.25	.09 – .41
calmness	-.20	-.35 – -.05

S14: PRISMA Checklist

PRISMA-IPD Checklist of items to include when reporting a systematic review and meta-analysis of individual participant data (IPD)

PRISMA-IPD Section/topic	Item No	Checklist item	Location where item is reported
**Title**			
Title	1	Identify the report as a systematic review and meta-analysis of individual participant data.	Title (p. 1)
**Abstract**			
Structured summary	2	Provide a structured summary including as applicable:	Abstract (p. 3)
		**Background**: state research question and main objectives, with information on participants, interventions, comparators and outcomes.	
		**Methods**: report eligibility criteria; data sources including dates of last bibliographic search or elicitation, noting that IPD were sought; methods of assessing risk of bias.	
		**Results**: provide number and type of studies and participants identified and number (%) obtained; summary effect estimates for main outcomes (benefits and harms) with confidence intervals and measures of statistical heterogeneity. Describe the direction and size of summary effects in terms meaningful to those who would put findings into practice.	
		**Discussion:** state main strengths and limitations of the evidence, general interpretation of the results and any important implications.	
		**Other:** report primary funding source, registration number and registry name for the systematic review and IPD meta-analysis.	
**Introduction**			
Rationale	3	Describe the rationale for the review in the context of what is already known.	Introduction (p. 4)
Objectives	4	Provide an explicit statement of the questions being addressed with reference, as applicable, to participants, interventions, comparisons, outcomes and study design (PICOS). Include any hypotheses that relate to particular types of participant-level subgroups.	Introduction (p. 5)
**Methods**			
Protocol and registration	5	Indicate if a protocol exists and where it can be accessed. If available, provide registration information including registration number and registry name. Provide publication details, if applicable.	Methods (p. 14)
Eligibility criteria	6	Specify inclusion and exclusion criteria including those relating to participants, interventions, comparisons, outcomes, study design and characteristics (e.g., years when conducted, required minimum follow-up). Note whether these were applied at the study or individual level i.e. whether eligible participants were included (and ineligible participants excluded) from a study that included a wider population than specified by the review inclusion criteria. The rationale for criteria should be stated.	Methods Data sources and study selection (p. 14)
Identifying studies - information sources	7	Describe all methods of identifying published and unpublished studies including, as applicable: which bibliographic databases were searched with dates of coverage; details of any hand searching including of conference proceedings; use of study registers and agency or company databases; contact with the original research team and experts in the field; open adverts and surveys. Give the date of last search or elicitation.	Methods Data sources and study selection (p. 14)
Identifying studies - search	8	Present the full electronic search strategy for at least one database, including any limits used, such that it could be repeated.	S15
Study selection processes	9	State the process for determining which studies were eligible for inclusion.	Methods Data sources and study selection (p. 14)
Data collection processes	10	Describe how IPD were requested, collected and managed, including any processes for querying and confirming data with investigators. If IPD were not sought from any eligible study, the reason for this should be stated (for each such study).	Methods Data synthesis (p. 14); Figure 1
		If applicable, describe how any studies for which IPD were not available were dealt with. This should include whether, how and what aggregate data were sought or extracted from study reports and publications (such as extracting data independently in duplicate) and any processes for obtaining and confirming these data with investigators.	
Data items	11	Describe how the information and variables to be collected were chosen. List and define all study level and participant level data that were sought, including baseline and follow-up information. If applicable, describe methods of standardising or translating variables within the IPD datasets to ensure common scales or measurements across studies.	Methods Data synthesis (p. 14); S1

IPD integrity	A1	Describe what aspects of IPD were subject to data checking (such as sequence generation, data consistency and completeness, baseline imbalance) and how this was done.	S1
Risk of bias assessment in individual studies.	12	Describe methods used to assess risk of bias in the individual studies and whether this was applied separately for each outcome. If applicable, describe how findings of IPD checking were used to inform the assessment. Report if and how risk of bias assessment was used in any data synthesis.	Methods<br>Risk of bias (p. 16); S16
Specification of outcomes and effect measures	13	State all treatment comparisons of interests. State all outcomes addressed and define them in detail. State whether they were pre-specified for the review and, if applicable, whether they were primary/main or secondary/additional outcomes. Give the principal measures of effect (such as risk ratio, hazard ratio, difference in means) used for each outcome.	Methods<br>Outcomes (pp. 14-15); S2; S3
Synthesis methods	14	Describe the meta-analysis methods used to synthesise IPD. Specify any statistical methods and models used. Issues should include (but are not restricted to):<br>• Use of a one-stage or two-stage approach.<br>• How effect estimates were generated separately within each study and combined across studies (where applicable).<br>• Specification of one-stage models (where applicable) including how clustering of patients within studies was accounted for.<br>• Use of fixed or random effects models and any other model assumptions, such as proportional hazards.<br>• How (summary) survival curves were generated (where applicable).<br>• Methods for quantifying statistical heterogeneity (such as I^2 and τ^2).<br>• How studies providing IPD and not providing IPD were analysed together (where applicable).<br>• How missing data within the IPD were dealt with (where applicable).	Methods<br>Data synthesis (p. 14), Analysis (p. 15-16); S1; S2
Exploration of variation in effects	A2	If applicable, describe any methods used to explore variation in effects by study or participant level characteristics (such as estimation of interactions between effect and covariates). State all participant-level characteristics that were analysed as potential effect modifiers, and whether these were pre-specified.	Methods<br>Data analysis (pp. 15-16); S11; S12
Risk of bias across studies	15	Specify any assessment of risk of bias relating to the accumulated body of evidence, including any pertaining to not obtaining IPD for particular studies, outcomes or other variables.	Methods<br>Risk of bias (p. 16), Additional analyses (p. 16); S19
Additional analyses	16	Describe methods of any additional analyses, including sensitivity analyses. State which of these were pre-specified.	Methods<br>Additional analyses (p. 16)
**Results**			
Study selection and IPD obtained	17	Give numbers of studies screened, assessed for eligibility, and included in the systematic review with reasons for exclusions at each stage. Indicate the number of studies and participants for which IPD were sought and for which IPD were obtained. For those studies where IPD were not available, give the numbers of studies and participants for which aggregate data were available. Report reasons for non-availability of IPD. Include a flow diagram.	Results,<br>Figure 1
Study characteristics	18	For each study, present information on key study and participant characteristics (such as description of interventions, numbers of participants, demographic data, unavailability of outcomes, funding source, and if applicable duration of follow-up). Provide (main) citations for each study. Where applicable, also report similar study characteristics for any studies not providing IPD.	Results (p. 5)<br>Table 1, S1, S19
IPD integrity	A3	Report any important issues identified in checking IPD or state that there were none.	Methods Data synthesis (p. 14); S1
Risk of bias within studies	19	Present data on risk of bias assessments. If applicable, describe whether data checking led to the up-weighting or down-weighting of these assessments. Consider how any potential bias impacts on the robustness of meta-analysis conclusions.	S16; S17
Results of individual studies	20	For each comparison and for each main outcome (benefit or harm), for each individual study report the number of eligible participants for which data were obtained and show simple summary data for each intervention group (including, where applicable, the number of events), effect estimates and confidence intervals. These may be tabulated or included on a forest plot.	Figures 3-7; S5-S8
Results of syntheses	21	Present summary effects for each meta-analysis undertaken, including confidence intervals and measures of statistical heterogeneity. State whether the analysis was pre-specified, and report the numbers of studies and participants and, where applicable, the number of events on which it is based.<br>When exploring variation in effects due to patient or study characteristics, present summary interaction estimates for each characteristic examined, including confidence intervals and measures of statistical heterogeneity. State whether the analysis was pre-specified. State whether any interaction is consistent across trials.	Results (pp. 6-9), Figures 3-7;<br>S5-S8, S11, S12

		Provide a description of the direction and size of effect in terms meaningful to those who would put findings into practice.	
Risk of bias across studies	22	Present results of any assessment of risk of bias relating to the accumulated body of evidence, including any pertaining to the availability and representativeness of available studies, outcomes or other variables.	S11
Additional analyses	23	Give results of any additional analyses (e.g. sensitivity analyses). If applicable, this should also include any analyses that incorporate aggregate data for studies that do not have IPD. If applicable, summarise the main meta-analysis results following the inclusion or exclusion of studies for which IPD were not available.	S4; S11; S13; S18; S19
**Discussion**			
Summary of evidence	24	Summarise the main findings, including the strength of evidence for each main outcome.	Discussion (pp. 10-11)
Strengths and limitations	25	Discuss any important strengths and limitations of the evidence including the benefits of access to IPD and any limitations arising from IPD that were not available.	Discussion (pp. 10-12)
Conclusions	26	Provide a general interpretation of the findings in the context of other evidence.	Discussion (pp. 10-12)
Implications	A4	Consider relevance to key groups (such as policy makers, service providers and service users). Consider implications for future research.	Discussion (pp. 10-12)
**Funding**			
Funding	27	Describe sources of funding and other support (such as supply of IPD), and the role in the systematic review of those providing such support.	Funding (pp. 17-18)

A1 – A3 denote new items that are additional to standard PRISMA items. A4 has been created as a result of re-arranging content of the standard PRISMA statement to suit the way that systematic review IPD meta-analyses are reported.

© Reproduced with permission of the PRISMA IPD Group, which encourages sharing and reuse for non-commercial purposes

S15: Comprehensive search term

Database	Complete search term
Scopus:	TITLE-ABS-KEY ("physical activit*" OR exercis* OR "sedentary behav*" OR sedentar* OR "physical inactivit*") AND TITLE-ABS-KEY (mood* OR emotion* OR affect OR "affec* stat*" OR valence OR calmness OR "energetic arousal") AND TITLE-ABS-KEY ("ambulatory assessment" OR "ecological momentary assessment" OR "experience sampling method*" OR "electronic sampling method" OR "ambulatory monitoring" OR acceler* OR "physical activity monitoring" OR "interactive assessment" OR "e*diar*" OR "electronic diar*") AND (LIMIT-TO (LANGUAGE , "English"))
Web of Science:	((TS=("physical activit*" OR exercis* OR "sedentary behav*" OR sedentar* OR "physical inactivit*") AND TS=(mood* OR emotion* OR affect OR "affec* stat*" OR valence OR calmness OR "energetic arousal") AND TS=("ambulatory assessment" OR "ecological momentary assessment" OR "experience sampling method*" OR "electronic sampling method" OR "ambulatory monitoring" OR acceler* OR "physical activity monitoring" OR "interactive assessment" OR "e*diar*" OR "electronic diar*"))) AND LANGUAGE: (English)
PubMed:	((physical activity[Title/Abstract] OR exercise[Title/Abstract] OR exercises[Title/Abstract] OR sedentary behavior[Title/Abstract] OR sedentary behaviour[Title/Abstract] OR sedentariness[Title/Abstract] OR physical inactivity[Title/Abstract]) AND (mood[Title/Abstract] OR moods[Title/Abstract] OR emotion[Title/Abstract] OR emotions[Title/Abstract] OR affect[Title/Abstract] OR affects[Title/Abstract] OR affective state[Title/Abstract] OR affective states[Title/Abstract] OR affective-states[Title/Abstract] OR valence[Title/Abstract] OR calmness[Title/Abstract] OR energetic arousal[Title/Abstract])) AND (ambulatory assessment[Title/Abstract] OR ecological momentary assessment[Title/Abstract] OR experience sampling method[Title/Abstract] OR (("electronics"[MeSH Terms] OR "electronics"[All Fields] OR "electronic"[All Fields]) AND sampling method[Title/Abstract]) OR ambulatory monitoring[Title/Abstract] OR accelerometry[Title/Abstract] OR accelerometer[Title/Abstract] OR physical activity monitoring[Title/Abstract] OR interactive assessment[Title/Abstract] OR e-diary[Title/Abstract] OR ediary[Title/Abstract] OR ediaries[Title/Abstract] OR electronic diary[Title/Abstract] OR electronic diaries[Title/Abstract]) AND English[lang]
PsychINFO	(TI physical activity OR AB physical activity OR TI exercise OR AB exercise OR TI exercises OR AB exercises OR TI sedentary behavior OR AB sedentary behavior OR TI sedentariness OR AB sedentariness OR TI physical inactivity OR AB physical inactivity) AND (TI mood OR AB mood OR TI emotion OR AB emotion OR TI emotions OR AB emotions OR TI moods OR AB moods OR TI affect OR AB affect OR TI affects OR AB affects OR TI affective state OR AB affective state OR TI affective-states OR AB affective-states OR TI valence OR AB valence OR TI calmness OR AB calmness OR TI energetic arousal OR AB energetic arousal) AND (TI ambulatory assessment OR AB ambulatory assessment OR TI ecological momentary assessment OR AB ecological momentary assessment OR TI experience sampling method OR AB experience sampling method OR TI electronic sampling method OR AB electronic sampling method OR TI ambulatory monitoring OR AB ambulatory monitoring OR TI accelerometry OR AB accelerometry OR TI accelerometer OR AB accelerometer OR TI physical activity monitoring OR AB physical activity monitoring OR TI interactive assessment OR AB interactive assessment OR TI e-diary OR AB e-diary OR TI ediary OR AB ediary OR TI ediaries OR AB ediaries OR TI electronic diary OR AB electronic diary OR TI electronic diaries OR AB electronic diaries)
SPORT DISCUS	((physical activity[Title/Abstract] OR exercise[Title/Abstract] OR exercises[Title/Abstract] OR sedentary behavior[Title/Abstract] OR sedentary behaviour[Title/Abstract] OR sedentariness[Title/Abstract] OR physical inactivity[Title/Abstract]) AND (mood[Title/Abstract] OR moods[Title/Abstract] OR emotion[Title/Abstract] OR emotions[Title/Abstract] OR affect[Title/Abstract] OR affects[Title/Abstract] OR affective state[Title/Abstract] OR affective states[Title/Abstract] OR affective-states[Title/Abstract] OR valence[Title/Abstract] OR calmness[Title/Abstract] OR energetic arousal[Title/Abstract])) AND (ambulatory assessment[Title/Abstract] OR ecological momentary assessment[Title/Abstract] OR experience sampling method[Title/Abstract] OR (("electronics"[MeSH Terms] OR "electronics"[All Fields] OR "electronic"[All Fields]) AND sampling method[Title/Abstract]) OR ambulatory monitoring[Title/Abstract] OR accelerometry[Title/Abstract] OR accelerometer[Title/Abstract] OR physical activity monitoring[Title/Abstract] OR interactive assessment[Title/Abstract] OR e-diary[Title/Abstract] OR ediary[Title/Abstract] OR ediaries[Title/Abstract] OR electronic diary[Title/Abstract] OR electronic diaries[Title/Abstract]) AND English[lang]

Note. We investigated the associations of PA and AWB in daily life. We used AWB as an established umbrella term for different affective phenomena such as mood, emotions, and affect, which cannot be used interchangeably. We chose a broad search term to achieve the best coverage of evidence.

S16: Risk of Bias assessment of individual studies using ROBINS-E

Publication	Domain 1: Confounding	Domain 2: Exposure	Domain 3: Participants	Domain 4: Interventions	Domain 5: Missing Data	Domain 6: Outcome	Domain 7: Reported Result	Overall Rating
Berli et al. (2021) [2]	NA	NA	Some concerns	Low risk of bias	NA	Low risk of bias	NA	NA
Bermudez et al. (2021) [4]	Some concerns	Some concerns	Some concerns	Low risk of bias	Some concerns	Low risk of bias	Some concerns	Some concerns
Bossmann et al. (2013) [5]	Some concerns	Low risk of bias	Some concerns	Low risk of bias	Some concerns	Low risk of bias	Some concerns	Some concerns
Bourke et al. (2021) [7]	Low risk of bias	Low risk of bias	Some concerns	Low risk of bias	Some concerns	Low risk of bias	Some concerns	Some concerns
Bourke et al. (2022) [8]	NA	Low risk of bias	Some concerns	Low risk of bias	NA	Low risk of bias	NA	NA
Bourke et al. (2023) [9]	Some concerns	Low risk of bias	Some concerns	Low risk of bias	Some concerns	Low risk of bias	Some concerns	Some concerns
Cabrita et al. (2017) [10]	Some concerns	Low risk of bias	Low risk of bias	Low risk of bias	Some concerns	Low risk of bias	Some concerns	Some concerns
Cook et al. (2022) [11]	Some concerns	Low risk of bias	Low risk of bias	Low risk of bias	Some concerns	Low risk of bias	Some concerns	Some concerns
Curtiss et al. (2022) [13]	High risk of bias	Some concerns	Some concerns	Low risk of bias	Some concerns	Low risk of bias	Some concerns	High risk of bias
Dickman et al. (2020) [15]	NA	Low risk of bias	Some concerns	Low risk of bias	NA	Low risk of bias	NA	NA
Dunton et al. (2011) [16]	Low risk of bias	NA	Some concerns	Low risk of bias	NA	Low risk of bias	NA	NA
Dunton et al. (2014) [19]	Very high risk of bias	Some concerns	Some concerns	Low risk of bias	Some concerns	Low risk of bias	Some concerns	Very high risk of bias
Elavsky et al. (2016) [20]	Some concerns	Low risk of bias	Some concerns	Low risk of bias	Some concerns	Low risk of bias	Some concerns	Some concerns
Elavsky et al. (2021) [21]	/	/	/	/	/	/	/	Data from other source
Elavsky et al. (2024) [22]	/	/	/	/	/	/	/	Data from other source
Gallagher & Carr (2021) [23]	High risk of bias	Low risk of bias	Low risk of bias	Low risk of bias	Some concerns	Low risk of bias	Some concerns	High risk of bias
Giurgiu et al. (2019) [25]	Low risk of bias	Low risk of bias	Some concerns	Low risk of bias	Some concerns	Low risk of bias	Some concerns	Some concerns
Giurgiu et al. (2020) [26]	Low risk of bias	Low risk of bias	Low risk of bias	Low risk of bias	Some concerns	Low risk of bias	Some concerns	Some concerns
Giurgiu et al. (2020) [27]	Low risk of bias	Low risk of bias	Some concerns	Low risk of bias	Some concerns	Low risk of bias	Some concerns	Some concerns
Giurgiu et al. (2022) [28]	Some concerns	Low risk of bias	Some concerns	Low risk of bias	Some concerns	Low risk of bias	Some concerns	Some concerns

Giurgiu et al. (2023) [29]	Some concerns	Low risk of bias	Some concerns	Low risk of bias	Some concerns	Low risk of bias	Some concerns	Some concerns
Timm et al. (2023) [30]	Some concerns	Low risk of bias	Low risk of bias	Low risk of bias	Some concerns	Low risk of bias	Some concerns	Some concerns
von Haaren et al. (2013) [31]	Very high risk of bias	Low risk of bias	Low risk of bias	Low risk of bias	High risk of bias	Low risk of bias	Some concerns	Very high risk of bias
Hachenberger et al. (2023) [32]	Some concerns	Some concerns	Some concerns	Low risk of bias	Some concerns	Low risk of bias	Some concerns	Some concerns
Haucke et al. (2022) [34]	Some concerns	Some concerns	Some concerns	Low risk of bias	Some concerns	Low risk of bias	Some concerns	Some concerns
Hevel et al. (2021) [35]	Some concerns	Low risk of bias	Some concerns	Low risk of bias	Some concerns	Low risk of bias	Some concerns	Some concerns
Hollands et al. (2020) [36]	Some concerns	Some concerns	Some concerns	Low risk of bias	Some concerns	Low risk of bias	Some concerns	Some concerns
Jeckel & Sudeck (2016) [37]	Some concerns	Low risk of bias	Low risk of bias	Low risk of bias	Some concerns	Low risk of bias	Some concerns	Some concerns
Jeckel & Sudeck (2018) [38]	High risk of bias	Low risk of bias	Low risk of bias	Low risk of bias	Some concerns	Low risk of bias	Some concerns	High risk of bias
Kanning (2009)	/	/	/	/	/	/	/	Data from other source
Kanning (2010)	/	/	/	/	/	/	/	Data from other source
Kanning et al. (2012)* [39]	Some concerns	Low risk of bias	Low risk of bias	Low risk of bias	Some concerns	Low risk of bias	Some concerns	Some concerns
Kanning (2013)* [40]	High risk of bias	Low risk of bias	Low risk of bias	Low risk of bias	Some concerns	Low risk of bias	Some concerns	High risk of bias
Kanning & Schoebi (2016)* [41]	Some concerns	Low risk of bias	Low risk of bias	Low risk of bias	Some concerns	Low risk of bias	Some concerns	Some concerns
Kanning et al. (2015)* [42]	Some concerns	Low risk of bias	Some concerns	Low risk of bias	Some concerns	Low risk of bias	Some concerns	Some concerns
Kanning & Hansen, (2017)* [43]	Some concerns	Low risk of bias	Some concerns	Low risk of bias	Some concerns	Low risk of bias	Some concerns	Some concerns
Kim et al. (2013) [44]	Very high risk of bias	Low risk of bias	Some concerns	Low risk of bias	Some concerns	Low risk of bias	Some concerns	Very high risk of bias
Kim et al. (2020) [46]	High risk of bias	Some concerns	Some concerns	Low risk of bias	Some concerns	Low risk of bias	Some concerns	High risk of bias
Kim et al. (2021) [47]	Some concerns	Low risk of bias	Some concerns	Low risk of bias	Some concerns	Low risk of bias	Some concerns	Some concerns
Koch et al. (2018) [48]	Some concerns	Low risk of bias	Some concerns	Low risk of bias	Some concerns	Low risk of bias	Some concerns	Some concerns
Koch et al. (2020) [50]	Some concerns	Low risk of bias	Some concerns	Low risk of bias	Some concerns	Low risk of bias	Some concerns	Some concerns

Koch et al. (2022) [51]	Some concerns	Some concerns	Some concerns	Low risk of bias	Some concerns	Low risk of bias	Some concerns	Some concerns
Kracht et al. (2021) [53]	Some concerns	Low risk of bias	Some concerns	Low risk of bias	Some concerns	Low risk of bias	Some concerns	Some concerns
Kuehnhausen et al. (2013) [55]	Some concerns	Low risk of bias	Some concerns	Low risk of bias	Some concerns	Low risk of bias	Some concerns	Some concerns
Leger et al. (2023) [56]	Low risk of bias	Low risk of bias	Some concerns	Low risk of bias	Some concerns	Low risk of bias	Some concerns	Some concerns
Li et al. (2022) [58]	High risk of bias	Low risk of bias	Some concerns	Low risk of bias	Some concerns	Low risk of bias	Some concerns	High risk of bias
Liao et al. (2017) [63]	Some concerns	Some concerns	Low risk of bias	Low risk of bias	Some concerns	Low risk of bias	Some concerns	Some concerns
Liao et al. (2017) [64]	High risk of bias	Some concerns	Some concerns	Low risk of bias	High risk of bias	Low risk of bias	Some concerns	High risk of bias
Maher et al. (2017) [65]	Low risk of bias	Low risk of bias	Some concerns	Low risk of bias	Some concerns	Low risk of bias	Some concerns	Some concerns
Madden et al. (2020) [66]	Some concerns	Low risk of bias	Some concerns	Low risk of bias	Some concerns	Low risk of bias	Some concerns	Some concerns
Maher (2022)	/	/	/	/	/	/	/	Data from other source
McCormick et al. (2008) [67]	High risk of bias	Low risk of bias	Some concerns	Low risk of bias	Some concerns	Low risk of bias	Some concerns	High risk of bias
Michalak et al. (2022) [68]	Some concerns	Some concerns	Some concerns	Low risk of bias	Some concerns	Low risk of bias	Some concerns	Some concerns
Olfermann et al. (2024) [69]	/	/	/	/	/	/	/	Data from other source
Pannicke et al. (2020) [70]	High risk of bias	Low risk of bias	Some concerns	Low risk of bias	Some concerns	Low risk of bias	Some concerns	High risk of bias
Pham et al. (2023) [72]	Some concerns	Low risk of bias	Some concerns	Low risk of bias	Some concerns	Low risk of bias	Some concerns	Some concerns
Poppe et al. (2021) [79]	Some concerns	Low risk of bias	Some concerns	Low risk of bias	Some concerns	Low risk of bias	Some concerns	Some concerns
Reichert et al. (2016) [80]	Some concerns	Low risk of bias	Some concerns	Low risk of bias	Some concerns	Low risk of bias	Some concerns	Some concerns
Reichert at al. (2017) [81]	Some concerns	Low risk of bias	Some concerns	Low risk of bias	Some concerns	Low risk of bias	Some concerns	Some concerns
Reichert et al. (2018) [82]	/	/	/	/	/	/	/	Data from other source
Reininghaus et al. (2023) [83]	/	/	/	/	/	/	/	Data from other source
Ruissen et al. (2022) [84]	Some concerns	Some concerns	Low risk of bias	Low risk of bias	Some concerns	Low risk of bias	Some concerns	Some concerns
Schwerdtfeger & Mai, (2009) [88]	Low risk of bias	Low risk of bias	Low risk of bias	Low risk of bias	Some concerns	Low risk of bias	Some concerns	Some concerns

Schwerdtfeger et al. (2015) [89]	Some concerns	Low risk of bias	Some concerns	Low risk of bias	Some concerns	Low risk of bias	Some concerns	Some concerns
Schwerdtfeger et al. (2010) [90]	Some concerns	Some concerns	Low risk of bias	Low risk of bias	Some concerns	Low risk of bias	Some concerns	Some concerns
Schwerdtfeger & Scheel (2012) [91]	/	/	/	/	/	/	/	Data from other source
Schwerdtfeger & Gerteis (2014) [92]	/	/	/	/	/	/	/	Data from other source
Schwerdtfeger & Rathner (2016) [94]	/	/	/	/	/	/	/	Data from other source
Schwerdtfeger & Dick (2019) [95]	/	/	/	/	/	/	/	Data from other source
Seiferth et al. (2023) [97]	/	/	/	/	/	/	/	Data from other source
Smith et al. (2020) [98]	Low risk of bias	Low risk of bias	Some concerns	Low risk of bias	Some concerns	Low risk of bias	Some concerns	Some concerns
Stevenson et al. (2022) [100]	Some concerns	Low risk of bias	Some concerns	Low risk of bias	Some concerns	Low risk of bias	Some concerns	Some concerns
Sudeck et al. (2018) [101]	Some concerns	Low risk of bias	Some concerns	Low risk of bias	Some concerns	Low risk of bias	Some concerns	Some concerns
Takano et al. (2013) [102]	Some concerns	Low risk of bias	Some concerns	Low risk of bias	Some concerns	Low risk of bias	Some concerns	Some concerns
Vetrovsky et al. (2021) [103]	Some concerns	Low risk of bias	Some concerns	Low risk of bias	Some concerns	Low risk of bias	Some concerns	Some concerns
Williams et al. (2020) [105]	Some concerns	Low risk of bias	Some concerns	Low risk of bias	Some concerns	Low risk of bias	Some concerns	Some concerns
Yang et al. (2020) [106]	Some concerns	Low risk of bias	Low risk of bias	Low risk of bias	Some concerns	Low risk of bias	Some concerns	Some concerns
Wen et al. (2018) [107]	Some concerns	Low risk of bias	Some concerns	Low risk of bias	Some concerns	Low risk of bias	Some concerns	Some concerns
Dunton et al. (2022) [108]	Some concerns	Low risk of bias	Some concerns	Low risk of bias	Some concerns	Low risk of bias	Some concerns	Some concerns
Kanning et al. (2020) [109]	Low risk of bias	Low risk of bias	Some concerns	Low risk of bias	Some concerns	Low risk of bias	Some concerns	Some concerns

Zarbo et al. (2023) [110]	Some concerns	Low risk of bias	Some concerns	Low risk of bias	Some concerns	Low risk of bias	Some concerns	Some concerns
Zhaoyang & Martire (2019) [111]	Low risk of bias	Low risk of bias	Some concerns	Low risk of bias	Some concerns	Low risk of bias	Some concerns	Some concerns

Notes. NA = not applicable, some studies did not analyse associations of physical activity and affective well-being but included physical activity or affective well-being as control variable.

S17: Quality Assessment of individual studies

Publication	Title	Rationale	Training	AA tech-nology	ACC tech-nology	Assess-ment duration	AA prompt design	AA prompt fre-quency	Para-metri-zation	Design features	Statistical methods	Defining non-wear	Latency	Delay possi-bility	Com-pliance/ missing data	Limi-tations	Overall
Berli et al. (2021) [2]	0	0	0.5	0	0.5	0.5	1	1	1	1	1	0.5	0	0	0	0	7
Bermudez et al. (2021) [4]	0	0	0	0	0.5	0.5	1	1	0.5	1	1	0	0	0	0	1	6.5
Bossmann et al. (2013) [5]	1	1	0.5	1	1	0.5	1	0	1	0	1	0	0	1	0	1	10
Bourke et al. (2021) [7]	1	0	0.5	0.5	1	1	1	1	1	0	1	0.5	0	1	0.5	1	11
Bourke et al. (2022) [8]	1	1	1	1	1	1	0	1	1	1	1	1	1	0	0	0.5	12.5
Bourke et al. (2023) [9]	1	0	0.5	0.5	1	1	1	1	1	1	1	1	1	1	1	1	14
Cabrita et al. (2017) [10]	0	1	1	0.5	0.5	0.5	1	1	0.5	1	0	0	0	0	0.5	1	8.5
Cook et al. (2022) [11]	0	1	1	1	0.5	0.5	0	1	1	0	1	0	0	0	0	1	8
Curtiss et al. (2022) [13]	0	0	0.5	1	0	0.5	0	1	0	0	0	0	0	0	0.5	1	4.5
Dickman et al. (2020) [15]	0	0	1	0.5	0.5	1	1	1	0.5	0	0	0	1	0	0	0	6.5
Dunton et al. (2011) [16]	1	0	0	1	0.5	1	1	1	1	1	0	0.5	1	1	0	0.5	10.5
Dunton et al. (2014) [19]	1	0	0	1	0	1	1	1	1	1	1	0.5	0	0	0.5	1	10
Elavsky et al. (2016) [20]	0	1	0.5	1	0.5	0.5	1	1	1	0	1	1	0	0	0.5	1	10
Elavsky et al. (2021) [21]	NA	NA	NA	NA	NA	NA	NA	NA	NA	NA	NA	NA	NA	NA	NA	NA	Data from other source
Elavsky et al. (2024) [22]	NA	NA	NA	NA	NA	NA	NA	NA	NA	NA	NA	NA	NA	NA	NA	NA	Data from other source
Gallagher & Carr (2021) [23]	0	0.5	0	1	0.5	1	1	1	1	0	0	0.5	0	0	0	0	6.5
Giurgiu et al. (2019) [25]	1	1	1	1	1	1	1	1	1	0	1	0.5	0	1	0.5	1	13

Giurgiu et al. (2020) [26]	0	1	1	1	1	1	1	1	1	1	1	0	0	1	0.5	1	12.5
Giurgiu et al. (2020) [27]	0	1	1	1	1	1	1	1	1	1	1	1	0	1	1	1	14
Giurgiu et al. (2022) [28]	0	1	1	1	1	1	1	1	1	1	1	1	0	1	0.5	0	12.5
Giurgiu et al. (2023) [29]	1	1	1	1	1	1	1	1	1	1	1	1	1	1	0.5	1	15.5
Timm et al. (2023) [30]	1	1	1	1	1	1	1	1	1	1	1	1	0	1	1	1	15
von Haaren et al. (2013) [31]	1	1	1	1	1	0.5	1	1	1	0	0	0	1	1	0	1	11.5
Hachenberger et al. (2023) [32]	1	1	1	1	1	0.5	1	1	1	0	1	0.5	1	0	0	1	12
Haucke et al. (2022) [34]	0	1	0	1	1	0.5	1	1	0	0	0	0.5	0	0	0	1	7
Hevel et al. (2021) [35]	1	1	1	1	0	0.5	1	1	1	0	0.5	0.5	0	1	0	1	10.5
Hollands et al. (2020) [36]	1	1	0	1	1	0.5	1	1	1	1	1	1	0	0	0.5	1	12
Jeckel & Sudeck (2016) [37]	0	0	1	1	0.5	1	1	1	1	1	1	1	0	1	0.5	1	12
Jeckel & Sudeck (2018) [38]	0	1	1	1	0.5	1	1	1	1	0	1	0	0	1	0.5	1	11
Kanning (2009)	NA	NA	NA	NA	NA	NA	NA	NA	NA	NA	NA	NA	NA	NA	NA	NA	Data from other source
Kanning (2010)	NA	NA	NA	NA	NA	NA	NA	NA	NA	NA	NA	NA	NA	NA	NA	NA	Data from other source
Kanning et al. (2012)* [39]	1	1	0	1	1	0.5	1	1	1	0	1	0	0	0	0	1	9.5
Kanning (2013)* [40]	0	1	0	0.5	1	1	1	1	1	1	1	0	0	1	0	1	10.5
Kanning & Schoebi (2016)* [41]	0	1	0	0.5	1	1	1	1	1	0	0.5	0.5	1	1	0	1	10.5
Kanning et al. (2015)* [42]	0	1	1	1	1	1	1	1	1	1	1	0	0	0	1	0	11
Kanning & Hansen, (2017)* [43]	1	1	0.5	1	1	1	1	1	1	1	1	0	0	0	0	1	11.5

Kim et al. (2013) [44]	1	1	1	0.5	1	1	1	1	1	0	0.5	0	0	1	0.5	1	11.5
Kim et al. (2020) [46]	0	1	1	0	0	1	1	1	0.5	1	1	0.5	0	0	0.5	1	9.5
Kim et al. (2021) [47]	0	1	0	0	0.5	1	1	1	1	0	1	0.5	0	0	0	0	7
Koch et al. (2018) [48]	1	1	1	1	1	1	1	1	1	0	1	0.5	0	1	1	1	13.5
Koch et al. (2020) [50]	0	1	1	1	1	0.5	1	1	1	1	1	1	0	1	0.5	0	12
Koch et al. (2022) [51]	0	1	0	0.5	1	0.5	1	1	1	0	1	0.5	0	0	0	0	7.5
Kracht et al. (2021) [53]	1	1	1	1	0	1	1	1	1	1	1	1	0	1	0.5	1	13.5
Kuehnhausen et al. (2013) [55]	0	1	0	0	1	0.5	0	1	0.5	1	0.5	0.5	0	0	0.5	0	6.5
Leger et al. (2023) [56]	0	1	0	0.5	0.5	1	1	1	0	0	1	0.5	0	0	0	1	7.5
Li et al. (2022) [58]	1	0	0.5	1	1	0.5	1	1	1	1	1	0	0	0	0	1	10
Liao et al. (2017) [63]	1	1	1	1	0	1	1	1	1	1	1	1	0	1	0.5	1	13.5
Liao et al. (2017) [64]	1	1	0	0	0	1	1	1	0.5	0	0	0.5	0	0	0	0	6
Maher et al. (2017) [65]	0	1	1	1	0	0.5	1	1	0	1	1	0.5	1	1	1	1	12
Madden et al. (2020) [66]	0	1	1	1	0	0.5	1	1	1	1	1	0.5	0	1	1	1	12
Maher (2022)	NA	NA	NA	NA	NA	NA	NA	NA	NA	NA	NA	NA	NA	NA	NA	NA	Data from other source
McCormick et al. (2008) [67]	0	1	0	0.5	0.5	0.5	1	1	0.5	0	0.5	1	0	0	0	0	6.5
Michalak et al. (2022) [68]	0	0	0	1	1	0.5	1	1	1	1	1	0	0	0	0	1	8.5
Olfermann et al. (2024) [69]	NA	NA	NA	NA	NA	NA	NA	NA	NA	NA	NA	NA	NA	NA	NA	NA	Data from other source
Pannicke et al. (2020) [70]	0	1	1	0	0	1	1	1	1	1	1	1	0	1	0.5	1	11.5
Pham et al. (2023) [72]	0	1	0.5	1	0.5	0.5	1	1	1	1	1	0.5	0	0	0	0	9

Poppe et al. (2021) [79]	0	1	0.5	1	0.5	0.5	1	1	1	1	1	1	0	1	0	1	11.5
Reichert et al. (2016) [80]	1	1	1	1	1	0.5	1	1	1	0	1	0.5	0	1	1	1	13
Reichert at al. (2017) [81]	0	1	1	1	1	0.5	1	1	1	0	1	0.5	0	1	0.5	1	11.5
Reichert et al. (2018) [82]	NA	NA	NA	NA	NA	NA	NA	NA	NA	NA	NA	NA	NA	NA	NA	NA	Data from other source
Reininghaus et al. (2023) [83]	NA	NA	NA	NA	NA	NA	NA	NA	NA	NA	NA	NA	NA	NA	NA	NA	Data from other source
Ruissen et al. (2022) [84]	0	0	1	1	0.5	0.5	1	1	1	1	1	0.5	1	0	0	1	10.5
Schwerdtfeger & Mai, (2009) [88]	1	0	1	0.5	1	0.5	1	0	0	0	1	0	0	0	0	0	6
Schwerdtfeger et al. (2015) [89]	0	1	1	0.5	1	1	1	1	0	1	1	0	0	0	0	0	8.5
Schwerdtfeger et al. (2010) [90]	1	1	1	1	1	1	1	1	1	1	1	0.5	0	1	0	1	13.5
Schwerdtfeger & Scheel (2012) [91]	NA	NA	NA	NA	NA	NA	NA	NA	NA	NA	NA	NA	NA	NA	NA	NA	Data from other source
Schwerdtfeger & Gerteis (2014) [92]	NA	NA	NA	NA	NA	NA	NA	NA	NA	NA	NA	NA	NA	NA	NA	NA	Data from other source
Schwerdtfeger & Rathner (2016) [94]	NA	NA	NA	NA	NA	NA	NA	NA	NA	NA	NA	NA	NA	NA	NA	NA	Data from other source
Schwerdtfeger & Dick (2019) [95]	NA	NA	NA	NA	NA	NA	NA	NA	NA	NA	NA	NA	NA	NA	NA	NA	Data from other source
Seiferth et al. (2023) [97]	NA	NA	NA	NA	NA	NA	NA	NA	NA	NA	NA	NA	NA	NA	NA	NA	Data from other source
Smith et al. (2020) [98]	0	1	1	0.5	0.5	1	1	1	1	0	0.5	0.5	0	0	0.5	1	9.5

Stevenson et al. (2022) [100]	1	0	1	0.5	0.5	0.5	1	1	1	1	1	0	0	0	1	1	10.5
Sudeck et al. (2018) [101]	0	1	1	1	1	0.5	1	1	1	0	1	0	0	1	1	1	11.5
Takano et al. (2013) [102]	0	0	1	0	1	1	1	1	1	1	1	0.5	1	0	0.5	1	11
Vetrovsky et al. (2021) [103]	0	1	1	0.5	1	1	1	1	1	0	0.5	1	0	1	0.5	1	11.5
Williams et al. (2020) [105]	0	1	0.5	0	0	0.5	1	1	1	1	1	0	0	0	0	0	7
Yang et al. (2020) [106]	1	1	0	1	0	1	1	1	1	1	1	0.5	0	1	0	1	11.5
Wen et al. (2018) [107]	0	1	1	1	0.5	0.5	1	1	1	1	1	0.5	0	1	0	1	11.5
Dunton et al. (2022) [108]	0	1	1	1	0.5	1	1	1	1	1	1	1	1	0	0.5	1	13
Kanning et al. (2020) [109]	0	1	0.5	1	0	0.5	1	1	1	0	1	0.5	0	1	0	1	9.5
Zarbo et al. (2023) [110]	1	1	1	0	0.5	0.5	1	1	1	0	0.5	0.5	0	0	0	1	9
Zhaoyang & Martire (2019) [111]	0	1	0.5	0	0.5	0.5	0	1	0.5	0	1	1	0	0	0.5	1	7.5

S18: Sensitivity analyses of two-stage Individual Participant Data models

Table S18.1: Sensitivity analyses of antecedent two-stage Individual Participant Data models

			rho = 0.00			rho = 0.25			rho = 0.50			rho = 0.75			rho = 1.00		
	Level	Association	Beta	99.2% CI LB	99.2% CI UB	Beta	99.2% CI LB	99.2% CI UB	Beta	99.2% CI LB	99.2% CI UB	Beta	99.2% CI LB	99.2% CI UB	Beta	99.2% CI LB	99.2% CI UB
Overall Model	Antecedent Overall	Within	0.05	0.03	0.06	0.05	0.03	0.06	0.05	0.03	0.06	0.05	0.03	0.06	0.05	0.03	0.06
	Antecedent Overall	Between	0.08	0.04	0.12	0.08	0.04	0.12	0.08	0.04	0.12	0.08	0.04	0.12	0.08	0.04	0.12
AWB Concepts	Antecedent positive affective states	Within	0.07	0.04	0.10	0.07	0.04	0.10	0.07	0.04	0.10	0.07	0.04	0.10	0.07	0.04	0.10
	Antecedent positive affective states	Between	0.09	0.03	0.15	0.09	0.03	0.15	0.09	0.02	0.15	0.07	-0.00	0.15	0.05	-0.06	0.15
	Antecedent negative affective states	Within	-0.01	-0.03	0.01	-0.01	-0.03	0.01	-0.01	-0.03	0.01	-0.01	-0.03	0.01	-0.01	-0.03	0.01
	Antecedent negative affective states	Between	-0.07	-0.13	-0.01	-0.07	-0.13	-0.01	-0.06	-0.13	0.00	-0.05	-0.12	0.02	-0.04	-0.12	0.04
	Antecedent valence	Within	0.06	0.03	0.08	0.06	0.03	0.08	0.06	0.03	0.08	0.06	0.03	0.09	0.06	0.03	0.09
	Antecedent valence	Between	0.10	0.02	0.18	0.10	0.02	0.18	0.09	0.00	0.19	0.09	-0.02	0.19	0.09	-0.02	0.20
	Antecedent energetic arousal	Within	0.15	0.09	0.20	0.15	0.09	0.20	0.14	0.09	0.20	0.14	0.08	0.20	0.15	0.09	0.20
	Antecedent energetic arousal	Between	0.11	0.00	0.22	0.10	-0.01	0.22	0.10	-0.02	0.22	0.10	-0.03	0.22	0.11	-0.02	0.23
	Antecedent calmness	Within	-0.05	-0.09	-0.01	-0.05	-0.10	-0.01	-0.06	-0.10	-0.01	-0.06	-0.10	-0.01	-0.06	-0.10	-0.01
	Antecedent calmness	Between	0.02	-0.09	0.12	0.01	-0.11	0.12	-0.00	-0.13	0.12	-0.01	-0.14	0.13	-0.01	-0.15	0.12
Physical Activity Type	Antecedent Intensity	Within	0.05	0.03	0.07	0.05	0.04	0.07	0.05	0.04	0.07	0.05	0.04	0.07	0.05	0.04	0.07
	Antecedent Intensity	Between	0.08	0.03	0.12	0.08	0.04	0.12	0.08	0.04	0.12	0.08	0.04	0.13	0.08	0.03	0.13
	Antecedent Time	Within	-0.01	-0.03	0.01	-0.01	-0.02	0.01	-0.01	-0.02	0.01	-0.01	-0.03	0.01	-0.01	-0.04	0.01
	Antecedent Time	Between	0.10	-0.01	0.21	0.10	-0.02	0.21	0.10	-0.02	0.21	0.09	-0.04	0.22	0.08	-0.07	0.23
Age Group	Antecedent Adults	Within	0.05	0.03	0.07	0.05	0.03	0.07	0.05	0.04	0.07	0.05	0.04	0.07	0.05	0.04	0.07
	Antecedent Adults	Between	0.07	0.02	0.12	0.07	0.03	0.12	0.07	0.02	0.12	0.07	0.02	0.13	0.07	0.02	0.13
	Antecedent Non-adults	Within	0.03	-0.01	0.08	0.04	-0.01	0.08	0.04	-0.00	0.08	0.04	-0.01	0.08	0.04	-0.01	0.09
	Antecedent Non-adults	Between	0.08	-0.04	0.20	0.09	-0.02	0.20	0.09	-0.02	0.21	0.10	-0.02	0.21	0.09	-0.03	0.22

	Antecedent Older Adults	Within	0.02	-0.01	0.06	0.02	-0.01	0.06	0.02	-0.01	0.06	0.02	-0.01	0.06	0.03	-0.01	0.06
	Antecedent Older Adults	Between	0.11	-0.01	0.23	0.11	-0.01	0.23	0.11	-0.00	0.23	0.11	-0.00	0.23	0.12	-0.00	0.24
**Region**	Antecedent Asia	Within	0.09	0.05	0.13	0.09	0.05	0.13	0.09	0.04	0.13	0.09	0.04	0.13	0.09	0.04	0.14
	Antecedent Asia	Between	0.03	-0.11	0.16	0.03	-0.11	0.16	0.02	-0.12	0.16	0.02	-0.13	0.17	0.02	-0.14	0.18
	Antecedent Europe	Within	0.05	0.03	0.07	0.05	0.03	0.07	0.05	0.03	0.07	0.05	0.03	0.07	0.05	0.03	0.07
	Antecedent Europe	Between	0.08	0.02	0.13	0.08	0.02	0.14	0.08	0.03	0.14	0.09	0.03	0.15	0.09	0.03	0.15
	Antecedent North America	Within	0.03	0.00	0.05	0.03	0.00	0.05	0.03	0.00	0.06	0.03	0.01	0.06	0.03	0.00	0.06
	Antecedent North America	Between	0.08	0.02	0.14	0.08	0.02	0.15	0.09	0.02	0.15	0.08	0.02	0.15	0.08	0.00	0.16
**Sensor Position**	Antecedent Distal	Within	0.05	0.03	0.08	0.06	0.03	0.08	0.06	0.03	0.08	0.06	0.03	0.08	0.06	0.03	0.09
	Antecedent Distal	Between	0.08	0.02	0.14	0.07	0.02	0.13	0.07	0.02	0.13	0.07	0.02	0.13	0.07	0.01	0.13
	Antecedent Proximal	Within	0.04	0.02	0.06	0.04	0.03	0.06	0.05	0.03	0.06	0.05	0.03	0.07	0.05	0.03	0.07
	Antecedent Proximal	Between	0.08	0.02	0.13	0.08	0.03	0.14	0.09	0.03	0.14	0.09	0.03	0.15	0.09	0.02	0.15
**Prompts**	Prompts	Both	-0.00	-0.01	0.00	-0.00	-0.01	0.00	-0.00	-0.01	0.00	-0.00	-0.01	0.00	-0.00	-0.01	0.00
**Days of Sampling**	Days of Sampling	Both	0.00	-0.00	0.00	0.00	-0.00	0.00	0.00	-0.00	0.00	0.00	-0.00	0.00	0.00	-0.00	0.00
**Short Interval**	Short Interval	Both	0.00	-0.00	0.01	0.00	-0.00	0.01	0.00	-0.00	0.01	0.00	-0.00	0.01	0.00	-0.00	0.01
**Long Interval**	Long Interval	Both	-0.00	-0.00	0.00	-0.00	-0.00	0.00	-0.00	-0.00	0.00	-0.00	-0.00	0.00	-0.00	-0.00	0.00

Note. AWB = affective well-being. CI = confidence interval. LB = lower bound. UP = upper bound

Table S18.2: Sensitivity analyses of consequent two-stage Individual Participant Data models

			rho = 0.00			rho = 0.25			rho = 0.50			rho = 0.75			rho = 1.00		
	Level	Association	Beta	99.2% CI LB	99.2% CI UB	Beta	99.2% CI LB	99.2% CI UB	Beta	99.2% CI LB	99.2% CI UB	Beta	99.2% CI LB	99.2% CI UB	Beta	99.2% CI LB	99.2% CI UB
**Overall Model**	Consequent Overall	Within	0.03	0.02	0.04	0.04	0.02	0.04	0.04	0.03	0.05	0.04	0.03	0.05	0.04	0.03	0.05
	Consequent Overall	Between	0.08	0.04	0.12	0.08	0.04	0.13	0.08	0.04	0.13	0.08	0.04	0.13	0.08	0.04	0.13
**AWB Concepts**	Consequent positive affective states	Within	0.04	0.01	0.08	0.05	0.01	0.08	0.05	0.01	0.09	0.05	0.01	0.10	0.05	0.01	0.10
	Consequent positive affective states	Between	0.13	0.06	0.21	0.13	0.05	0.21	0.13	0.05	0.21	0.12	0.02	0.21	0.08	-0.07	0.23
	Consequent negative affective states	Within	-0.01	-0.03	0.01	-0.01	-0.04	0.01	-0.02	-0.04	0.01	-0.02	-0.04	0.01	-0.02	-0.04	0.01
	Consequent negative affective states	Between	-0.09	-0.18	-0.00	-0.09	-0.18	-0.00	-0.09	-0.18	0.01	-0.08	-0.18	0.03	-0.06	-0.18	0.07
	Consequent valence	Within	0.02	0.00	0.04	0.03	0.01	0.05	0.03	0.01	0.05	0.03	0.00	0.06	0.03	0.00	0.06
	Consequent valence	Between	0.06	-0.08	0.20	0.06	-0.07	0.19	0.05	-0.09	0.18	0.04	-0.10	0.18	0.04	-0.11	0.20
	Consequent energetic arousal	Within	0.11	0.08	0.15	0.12	0.08	0.16	0.12	0.08	0.16	0.12	0.08	0.16	0.12	0.08	0.16
	Consequent energetic arousal	Between	0.03	-0.11	0.16	0.02	-0.11	0.15	0.01	-0.13	0.14	0.00	-0.14	0.15	0.01	-0.15	0.16
	Consequent calmness	Within	-0.07	-0.10	-0.03	-0.06	-0.09	-0.03	-0.06	-0.10	-0.03	-0.06	-0.10	-0.02	-0.06	-0.10	-0.02
	Consequent calmness	Between	-0.03	-0.19	0.13	-0.04	-0.22	0.13	-0.06	-0.25	0.14	-0.06	-0.26	0.13	-0.05	-0.23	0.13
**Physical Activity Type**	Consequent Intensity	Within	0.04	0.02	0.05	0.04	0.03	0.05	0.04	0.03	0.06	0.05	0.03	0.06	0.05	0.03	0.07
	Consequent Intensity	Between	0.07	0.01	0.14	0.08	0.01	0.15	0.08	0.01	0.15	0.08	0.01	0.15	0.08	0.01	0.16
	Consequent Time	Within	-0.01	-0.03	0.01	-0.01	-0.02	0.01	-0.01	-0.02	0.01	-0.01	-0.02	0.01	-0.01	-0.04	0.02
	Consequent Time	Between	0.12	0.00	0.23	0.12	-0.00	0.24	0.11	-0.01	0.24	0.11	-0.04	0.25	0.09	-0.08	0.26
**Age Group**	Consequent Adults	Within	0.04	0.02	0.05	0.04	0.03	0.06	0.04	0.03	0.06	0.05	0.03	0.07	0.05	0.03	0.07
	Consequent Adults	Between	0.06	-0.03	0.15	0.06	-0.02	0.15	0.06	-0.02	0.15	0.06	-0.02	0.15	0.05	-0.04	0.15
	Consequent Non-adults	Within	0.04	0.00	0.07	0.04	0.00	0.07	0.04	0.00	0.08	0.04	-0.00	0.08	0.04	-0.01	0.09
	Consequent Non-adults	Between	0.08	-0.04	0.21	0.09	-0.03	0.22	0.10	-0.03	0.23	0.10	-0.04	0.24	0.11	-0.05	0.26
	Consequent Older Adults	Within	0.00	-0.03	0.03	0.00	-0.03	0.03	0.00	-0.03	0.04	0.01	-0.03	0.04	0.01	-0.03	0.05

	Consequent Older Adults	Between	0.18	0.07	0.29	0.18	0.07	0.29	0.18	0.08	0.29	0.19	0.08	0.29	0.19	0.07	0.31
**Region**	Consequent Asia	Within	0.10	0.06	0.14	0.10	0.06	0.15	0.11	0.06	0.15	0.11	0.06	0.15	0.11	0.06	0.15
	Consequent Asia	Between	0.05	-0.07	0.18	0.05	-0.06	0.17	0.06	-0.04	0.15	0.06	-0.02	0.14	0.06	-0.02	0.14
	Consequent Europe	Within	0.03	0.01	0.04	0.03	0.02	0.04	0.03	0.02	0.05	0.03	0.02	0.05	0.03	0.02	0.05
	Consequent Europe	Between	0.06	-0.01	0.13	0.06	-0.01	0.13	0.06	-0.01	0.14	0.07	-0.01	0.14	0.07	-0.01	0.14
	Consequent North America	Within	0.02	-0.01	0.04	0.02	-0.00	0.05	0.03	-0.00	0.06	0.03	-0.00	0.07	0.03	-0.00	0.08
	Consequent North America	Between	0.12	0.02	0.23	0.13	0.02	0.23	0.12	0.02	0.23	0.12	0.01	0.24	0.12	-0.01	0.24
**Sensor Position**	Consequent Distal	Within	0.05	0.02	0.08	0.05	0.02	0.08	0.05	0.01	0.08	0.05	0.01	0.09	0.05	0.01	0.10
	Consequent Distal	Between	0.08	0.01	0.16	0.08	0.01	0.15	0.08	0.01	0.15	0.08	-0.00	0.16	0.08	-0.02	0.17
	Consequent Proximal	Within	0.03	0.01	0.04	0.03	0.02	0.04	0.03	0.02	0.05	0.04	0.02	0.05	0.04	-0.00	0.05
	Consequent Proximal	Between	0.08	-0.01	0.16	0.08	-0.00	0.17	0.09	0.00	0.17	0.09	0.00	0.18	0.09	-0.00	0.18
**Prompts**	Prompts	Both	-0.01	-0.03	0.01	-0.01	-0.02	0.01	-0.01	-0.02	0.01	-0.01	-0.03	0.00	-0.01	-0.03	0.00
**Days of Sampling**	Days of Sampling	Both	0.01	0.00	0.01	0.01	0.00	0.01	0.01	0.00	0.01	0.01	0.00	0.01	0.01	0.00	0.01
**Short Interval**	Short Interval	Both	0.01	-0.00	0.02	0.01	-0.01	0.02	0.01	-0.01	0.02	0.01	-0.01	0.02	0.01	-0.01	0.02
**Long Interval**	Long Interval	Both	0.00	-0.00	0.00	0.00	-0.00	0.00	0.00	-0.00	0.00	0.00	-0.00	0.00	0.00	-0.00	0.00

Note. AWB = affective well-being. CI = confidence interval. LB = lower bound. UP = upper bound

S19: Predicting data provision by study characteristics

To explore systematic differences between studies that provided data and studies that did not provide data for our meta-analysis, we extracted relevant information from publications. We set up a logistic regression model and predicted data provision by study characteristics. The analysis included the predictors mean age, percentage of females, number of study participants, study location, prompts per day, days of sampling as well as antecedent and consequent models. In the studies included, physical activity was more often investigated prior to affective well-being than in studies that did not provide data (p = .01). None of the other variables predicted data provision.

	Data provision		
Predictors	*Odds Ratios*	*95% CI*	*p*
(Intercept)	0.05	0.00 – 1.82	.119
continent [Australia]	0.37	0.01 – 7.82	.526
continent [Europe]	4.93	0.75 – 33.64	.092
continent [Mixed]	10609681.11	0.00 – NA	.992
continent [Northamerica]	1.16	0.17 – 8.12	.877
sample size	1.00	1.00 – 1.01	.416
days of sampling	0.98	0.94 – 1.00	.194
prompts per day	1.06	0.92 – 1.25	.456
percentage female	0.11	0.01 – 1.46	.107
mean age	1.01	0.98 – 1.05	.485
PA assessed before AWB [1]	25.52	2.87 – 619.50	**.011**
PA assessed after AWB [1]	2.94	1.01 – 9.22	.053
Observations	101		

Note. k = 11 studies were excluded from this analysis, as not all relevant information could not be extracted from the publication.

References

1. Viechtbauer, W. Structuring, checking, and preparing the data (Center for Research on Experience Sampling and Ambulatory Methods Leuven2021), pp. 137–152.
2. Berli, C., Schwaninger, P. & Scholz, U. "We Feel Good": Daily Support Provision, Health Behavior, and Well-Being in Romantic Couples. *Frontiers in Psychology* **11,** 622492; 10.3389/FPSYG.2020.622492/FULL (2021).
3. Thompson, E. R. Development and Validation of an Internationally Reliable Short-Form of the Positive and Negative Affect Schedule (PANAS). *Journal of Cross-Cultural Psychology* **38,** 227–242; 10.1177/0022022106297301 (2007).
4. Bermudez, T. *et al.* Physical activity after cardiac rehabilitation: Explicit and implicit attitudinal components and ambivalence. *Health psychology* **40,** 491–501; 10.1037/HEA0001109 (2021).
5. Bossmann, T., Kanning, M., Koudela-Hamila, S., Hey, S. & Ebner-Priemer, U. The Association between Short Periods of Everyday Life Activities and Affective States: A Replication Study Using Ambulatory Assessment. *Frontiers in Psychology* **4,** 102; 10.3389/FPSYG.2013.00102 (2013).
6. Wilhelm, P. & Schoebi, D. Assessing mood in daily life: Structural validity, sensitivity to change, and reliability of a short-scale to measure three basic dimensions of mood. *European Journal of Psychological Assessment* **23,** 258–267; 10.1027/1015-5759.23.4.258 (2007).
7. Bourke, M., Hilland, T. A. & Craike, M. Contextual influences on the within-person association between physical activity and affect in adolescents: an ecological momentary assessment study. *Journal of behavioral medicine* **44,** 296–309; 10.1007/s10865-020-00197-4 (2021).
8. Bourke, M., Hilland, T. A. & Craike, M. Daily Physical Activity and Satisfaction with Life in Adolescents: An Ecological Momentary Assessment Study Exploring Direct Associations and the Mediating Role of Core Affect. *Journal of Happiness Studies* **23,** 949–968; 10.1007/S10902-021-00431-Z (2022).
9. Bourke, M., Hilland, T. A. & Craike, M. Domain specific association between physical activity and affect in adolescents' daily lives: an ecological momentary assessment study. *Psychology & health* **38,** 369–388; 10.1080/08870446.2021.1965603 (2023).
10. Cabrita, M., Lousberg, R., Tabak, M., Hermens, H. J. & Vollenbroek-Hutten, M. M. R. An exploratory study on the impact of daily activities on the pleasure and physical activity of older adults. *European review of aging and physical activity : official journal of the European Group for Research into Elderly and Physical Activity* **14,** 1; 10.1186/s11556-016-0170-2 (2017).
11. Cook, P. *et al.* Low- and High-Intensity Physical Activity Among People with HIV: Multilevel Modeling Analysis Using Sensor- and Survey-Based Predictors. *JMIR mHealth and uHealth* **10**; 10.2196/33938 (2022).
12. Kamarck, T. W. *et al.* The Diary of Ambulatory Behavioral States: A new approach to the assessment of psychosocial influences on ambulatory cardiovascular activity. In *Technology*

and methods in behavioral medicine, edited by D. S. Krantz & A. Baum (Lawrence Erlbaum Associates Publishers, Mahwah, NJ, 1998), pp. 163–193.

13. Curtiss, J. E., Pinaire, M., Fulford, D., McNally, R. J. & Hofmann, S. G. Temporal and contemporaneous network structures of affect and physical activity in emotional disorders. *Journal of affective disorders* **315,** 139–147; 10.1016/j.jad.2022.07.061 (2022).
14. van de Leemput, I. A. *et al.* Critical slowing down as early warning for the onset and termination of depression. *Proceedings of the National Academy of Sciences of the United States of America* **111,** 87–92; 10.1073/pnas.1312114110 (2014).
15. Dickman, K. D., Thomas, M. C., Anderson, B., Manuck, S. B. & Kamarck, T. W. Social Integration and Diurnal Cortisol Decline: The Role of Psychosocial and Behavioral Pathways. *Psychosomatic Medicine* **82,** 568; 10.1097/PSY.0000000000000825 (2020).
16. Dunton, G. F., Liao, Y., Intille, S., Wolch, J. & Pentz, M. A. Physical and social contextual influences on children's leisure-time physical activity: an ecological momentary assessment study. *Journal of physical activity & health* **8 Suppl 1,** 103–108; 10.1123/JPAH.8.S1.S103 (2011).
17. Ebesutani, C. *et al.* The 10-Item Positive and Negative Affect Schedule for Children, Child and Parent Shortened Versions: Application of Item Response Theory for More Efficient Assessment. *J Psychopathol Behav Assess* **34,** 191–203; 10.1007/s10862-011-9273-2 (2012).
18. Laurent, J. *et al.* A measure of positive and negative affect for children: Scale development and preliminary validation. *Psychological assessment* **11,** 326–338; 10.1037/1040-3590.11.3.326 (1999).
19. Dunton, G. F. *et al.* Momentary assessment of affect, physical feeling states, and physical activity in children. *Health psychology : official journal of the Division of Health Psychology, American Psychological Association* **33,** 255–263; 10.1037/a0032640 (2014).
20. Elavsky, S., Kishida, M. & Mogle, J. A. Concurrent and lagged relations between momentary affect and sedentary behavior in middle-aged women. *Menopause (New York, N.Y.)* **23,** 919–923; 10.1097/GME.0000000000000645 (2016).
21. Elavsky, S. *et al.* Feasibility of Real-time Behavior Monitoring Via Mobile Technology in Czech Adults Aged 50 Years and Above: 12-Week Study With Ecological Momentary Assessment. *JMIR* **4,** e15220; 10.2196/15220 (2021).
22. Elavsky, S. *et al.* Physical activity in an air-polluted environment: behavioral, psychological and neuroimaging protocol for a prospective cohort study (Healthy Aging in Industrial Environment study – Program 4). *BMC Public Health* **21,** 1–14; 10.1186/S12889-021-10166-4/FIGURES/2 (2021).
23. Gallagher, J. & Carr, L. J. Leisure but Not Occupational Physical Activity and Sedentary Behavior Associated With Better Health. *Journal of occupational and environmental medicine* **63,** E774-E782; 10.1097/JOM.0000000000002365 (2021).
24. Mayer, J. D. & Gaschke, Y. N. The experience and meta-experience of mood. *Journal of personality and social psychology* **55,** 102–111; 10.1037/0022-3514.55.1.102 (1988).

25. Giurgiu, M. *et al.* Sedentary behavior in everyday life relates negatively to mood: An ambulatory assessment study. *Scandinavian Journal of Medicine & Science in Sports* **29,** 1340–1351; 10.1111/sms.13448 (2019).

26. Giurgiu, M., Koch, E. D., Plotnikoff, R. C., Ebner-Priemer, U. W. & Reichert, M. Breaking Up Sedentary Behavior Optimally to Enhance Mood. *Medicine and Science in Sports and Exercise* **52,** 457–465; 10.1249/MSS.0000000000002132 (2020).

27. Giurgiu, M. *et al.* Momentary mood predicts upcoming real-life sedentary behavior. *Scandinavian Journal of Medicine & Science in Sports* **30,** 1276–1286; 10.1111/SMS.13652 (2020).

28. Giurgiu, M., Ebner-Priemer, U. W. & Dumuid, D. Compositional insights on the association between physical activity and sedentary behavior on momentary mood in daily life. *Psychology of Sport and Exercise* **58**; 10.1016/J.PSYCHSPORT.2021.102102 (2022).

29. Giurgiu, M. & Ebner-Priemer, U. W. Momentary associations between sedentary bouts, cognitive load and mood in daily life: An ambulatory assessment study. *Mental Health and Physical Activity* **25**; 10.1016/J.MHPA.2023.100540 (2023).

30. Timm, I., Reichert, M., Ebner-Priemer, U. W. & Giurgiu, M. Momentary within-subject associations of affective states and physical behavior are moderated by weather conditions in real life: an ambulatory assessment study. *International Journal of Behavioral Nutrition and Physical Activity* **20**; 10.1186/S12966-023-01507-0 (2023).

31. Haaren, B. v. *et al.* Characteristics of the activity-affect association in inactive people: an ambulatory assessment study in daily life. *Frontiers in Psychology* **4,** 163; 10.3389/fpsyg.2013.00163 (2013).

32. Hachenberger, J. *et al.* Investigating associations between physical activity, stress experience, and affective wellbeing during an examination period using experience sampling and accelerometry. *Scientific Reports 2023 13:1* **13,** 1–10; 10.1038/s41598-023-35987-8 (2023).

33. Das-Friebel, A. *et al.* Bedtime social media use, sleep, and affective wellbeing in young adults: an experience sampling study. *Journal of child psychology and psychiatry, and allied disciplines* **61,** 1138–1149; 10.1111/jcpp.13326 (2020).

34. Haucke, M., Heinz, A., Liu, S. & Heinzel, S. The Impact of COVID-19 Lockdown on Daily Activities, Cognitions, and Stress in a Lonely and Distressed Population: Temporal Dynamic Network Analysis. *Journal of Medical Internet Research* **24,** e32598; 10.2196/32598 (2022).

35. Hevel, D. J., Dunton, G. F. & Maher, J. P. Acute Bidirectional Relations Between Affect, Physical Feeling States, and Activity-Related Behaviors Among Older Adults: An Ecological Momentary Assessment Study. *Annals of behavioral medicine : a publication of the Society of Behavioral Medicine* **55,** 41–54; 10.1093/abm/kaaa027 (2021).

36. Hollands, L., Lambert, J., Price, L., Powell, D. & Greaves, C. Ecological momentary assessment of mood and physical activity in people with depression. *Journal of affective disorders* **271,** 293–299; 10.1016/J.JAD.2020.03.085 (2020).

37. Jeckel, S. & Sudeck, G. Physical Activity and Affective Well-Being in Everyday Life: Comparing sport activities and dailyphysical activities regardings acute and sustainable

associations. *Zeitschrift für Gesundheitspsychologie* **24,** 130–144; 10.1026/0943-8149/a000163 (2016).

38. Jeckel, S. & Sudeck, G. Sport activities in daily routine. *German Journal of Exercise and Sport Research* **48,** 26–39; 10.1007/s12662-017-0469-9 (2018).
39. Kanning, M., Ebner-Priemer, U. & Brand, R. Autonomous Regulation Mode Moderates the Effect of Actual Physical Activity on Affective States: An Ambulant Assessment Approach to the Role of Self-Determination. *Journal of Sport and Exercise Psychology* **34,** 260–269; 10.1123/JSEP.34.2.260 (2012).
40. Kanning, M. Using objective, real-time measures to investigate the effect of actual physical activity on affective States in everyday life differentiating the contexts of working and leisure time in a sample with students. *Frontiers in Psychology* **3,** 602; 10.3389/FPSYG.2012.00602 (2012).
41. Kanning, M. K. & Schoebi, D. Momentary Affective States Are Associated with Momentary Volume, Prospective Trends, and Fluctuation of Daily Physical Activity. *Frontiers in Psychology* **7,** 744; 10.3389/fpsyg.2016.00744 (2016).
42. Kanning, M., Ebner-Priemer, U. & Schlicht, W. Using activity triggered e-diaries to reveal the associations between physical activity and affective states in older adult's daily living. *International Journal of Behavioral Nutrition and Physical Activity* **12,** 1–10; 10.1186/s12966-015-0272-7 (2015).
43. Kanning, M. & Hansen, S. Need Satisfaction Moderates the Association Between Physical Activity and Affective States in Adults Aged 50+: an Activity-Triggered Ambulatory Assessment. *Annals of Behavioral Medicine* **51,** 18–29; 10.1007/S12160-016-9824-6 (2017).
44. Kim, J., Nakamura, T., Kikuchi, H., Sasaki, T. & Yamamoto, Y. Co-variation of depressive mood and locomotor dynamics evaluated by ecological momentary assessment in healthy humans. *PloS one* **8,** e74979; 10.1371/journal.pone.0074979 (2013).
45. Fukui, I. The Depression and Anxiety Mood Scale (DAMS): Scale Development and Validation. *Japanese Journal of Behavior Therapy,* 83–93 (1997).
46. Kim, J., Conroy, D. E. & Smyth, J. M. Bidirectional Associations of Momentary Affect with Physical Activity and Sedentary Behaviors in Working Adults. *Annals of behavioral medicine : a publication of the Society of Behavioral Medicine* **54,** 268–279; 10.1093/abm/kaz045 (2020).
47. Kim, J., Murata, T., Foo, J. C., Hossain, B. M. A. & Togo, F. A Pilot Study of Temporal Associations Between Psychological Stress and Cardiovascular Response. *Annu Int Cong IEEE Eng Med Bill Soc* **2021,** 7040–7043; 10.1109/EMBC46164.2021.9630872 (2021).
48. Koch, E. D. *et al.* Mood Dimensions Show Distinct Within-Subject Associations With Non-exercise Activity in Adolescents: An Ambulatory Assessment Study. *Frontiers in Psychology* **9,** 268; 10.3389/fpsyg.2018.00268 (2018).
49. Leonhardt, A., Könen, T., Dirk, J. & Schmiedek, F. How differentiated do children experience affect? An investigation of the within- and between-person structure of children's affect. *Psychological assessment* **28,** 575–585; 10.1037/pas0000195 (2016).

50. Koch, E. D. *et al.* Relationships between incidental physical activity, exercise, and sports with subsequent mood in adolescents. *Scandinavian Journal of Medicine & Science in Sports* **30,** 2234–2250; 10.1111/sms.13774 (2020).

51. Koch, E. D. *et al.* The dynamical association between physical activity and affect in the daily life of individuals with ADHD. *European neuropsychopharmacology : the journal of the European College of Neuropsychopharmacology* **57,** 69–74; 10.1016/j.euroneuro.2022.01.110 (2022).

52. Myin-Germeys, I. *et al.* Emotional reactivity to daily life stress in psychosis and affective disorder: an experience sampling study. *Acta psychiatrica Scandinavica* **107,** 124–131; 10.1034/j.1600-0447.2003.02025.x (2003).

53. Kracht, C. L., Beyl, R. A., Maher, J. P., Katzmarzyk, P. T. & Staiano, A. E. Adolescents' sedentary time, affect, and contextual factors: An ecological momentary assessment study. *The international journal of behavioral nutrition and physical activity* **18,** 53; 10.1186/s12966-021-01121-y (2021).

54. Ebesutani, C., Okamura, K., Higa-McMillan, C. & Chorpita, B. F. A psychometric analysis of the Positive and Negative Affect Schedule for Children-Parent Version in a school sample. *Psychological assessment* **23,** 406–416; 10.1037/a0022057 (2011).

55. Kühnhausen, J., Leonhardt, A., Dirk, J. & Schmiedek, F. Physical activity and affect in elementary school children's daily lives. *Frontiers in Psychology* **4,** 456; 10.3389/fpsyg.2013.00456 (2013).

56. Leger, K. A., Charles, S. T., Brown, C. J. & Fingerman, K. L. Physical Activity and Daily Stress Processes in Older Adulthood. *The journals of gerontology. Series B, Psychological sciences and social sciences* **78,** 20–29; 10.1093/GERONB/GBAC113 (2023).

57. Fingerman, K. L., Kim, K., Birditt, K. S. & Zarit, S. H. The Ties That Bind: Midlife Parents' Daily Experiences With Grown Children. *Journal of marriage and the family* **78,** 431–450; 10.1111/jomf.12273 (2016).

58. Li, Y.-M., Konstabel, K., Mõttus, R. & Lemola, S. Temporal associations between objectively measured physical activity and depressive symptoms: An experience sampling study. *Frontiers in psychiatry* **13,** 920580; 10.3389/fpsyt.2022.920580 (2022).

59. Kroenke, K., Spitzer, R. L. & Williams, J. B. The PHQ-9: validity of a brief depression severity measure. *Journal of general internal medicine* **16,** 606–613; 10.1046/j.1525-1497.2001.016009606.x (2001).

60. ZUNG, W. W. A Self-Rating Depression Scale. *Archives of general psychiatry* **12,** 63–70; 10.1001/archpsyc.1965.01720310065008 (1965).

61. Zigmond, A. S. & Snaith, R. P. The hospital anxiety and depression scale. *Acta psychiatrica Scandinavica* **67,** 361–370; 10.1111/j.1600-0447.1983.tb09716.x (1983).

62. Lovibond, S. H. & Lovibond, P. F. *PsycTESTS Dataset* (1995).

63. Liao, Y., Chou, C. P., Huh, J., Leventhal, A. & Dunton, G. Examining acute bi-directional relationships between affect, physical feeling states, and physical activity in free-living situations using electronic ecological momentary assessment. *Journal of Behavioral Medicine 2016 40:3* **40,** 445–457; 10.1007/S10865-016-9808-9 (2016).

64. Liao, Y., Chou, C.-P., Huh, J., Leventhal, A. & Dunton, G. Associations of Affective Responses During Free-Living Physical Activity and Future Physical Activity Levels: an Ecological Momentary Assessment Study. *International journal of behavioral medicine* **24,** 513–519; 10.1007/s12529-016-9626-z (2017).

65. Maher, J. P. *et al.* Momentary assessment of physical activity intention-behavior coupling in adults. *Translational behavioral medicine* **7,** 709–718; 10.1007/S13142-017-0472-6 (2017).

66. Madden, D. R. *et al.* Real-Time Data Collection to Examine Relations Between Physical Activity and Affect in Adults With Mental Illness. *Journal of sport 5&6 exercise psychology,* 1–8; 10.1123/jsep.2019-0035 (2020).

67. McCormick, B. P. *et al.* Predicting transitory mood from physical activity level among people with severe mental illness in two cultures. *The International journal of social psychiatry* **54,** 527–538; 10.1177/0020764008091423 (2008).

68. Michalak, J. *et al.* Subjective and Objective Measures of Activity in Depressed and Non-depressed Individuals in Everyday Life. *Journal of Experimental Psychopathology* **13,** 204380872210925; 10.1177/20438087221092582 (2022).

69. Olfermann, R. *et al.* Relationship between nonexercise activity and mood in patients with eating disorders. *Acta psychiatrica Scandinavica*; 10.1111/ACPS.13757 (2024).

70. Pannicke, B., Reichenberger, J., Schultchen, D., Pollatos, O. & Blechert, J. Affect Improvements and Measurement Concordance Between a Subjective and an Accelerometric Estimate of Physical Activity. *European Journal of Health Psychology* **27,** 66–75; 10.1027/2512-8442/a000050 (2020).

71. Breyer, B. & Bluemke, M. Deutsche Version der Positive and Negative Affect Schedule PANAS (GESIS Panel), 2016.

72. Pham, L. T., Hernandez, R., Spruijt-Metz, D., Gonzalez, J. S. & Pyatak, E. A. Movement matters: short-term impacts of physical activity on mood and well-being. *Journal of behavioral medicine* **46,** 781–790; 10.1007/S10865-023-00407-9 (2023).

73. Broderick, J. E., Schwartz, J. E., Schneider, S. & Stone, A. A. Can End-of-day reports replace momentary assessment of pain and fatigue? *The journal of pain* **10,** 274–281; 10.1016/j.jpain.2008.09.003 (2009).

74. Crawford, J. R. & Henry, J. D. The positive and negative affect schedule (PANAS): construct validity, measurement properties and normative data in a large non-clinical sample. *The British journal of clinical psychology* **43,** 245–265; 10.1348/0144665031752934 (2004).

75. Dunton, G. F., Berrigan, D., Ballard-Barbash, R., Graubard, B. I. & Atienza, A. A. Social and physical environments of sports and exercise reported among adults in the American Time Use Survey. *Preventive Medicine* **47,** 519–524; 10.1016/j.ypmed.2008.07.001 (2008).

76. Laurenceau, J.-P. Using mobile devices to study daily diabetes management in marital context (Grant No. R21 DK098679). National Institute of Diabetes and Digestive and Kidney Diseases, 2013.

77. Merwin, R. M. *et al.* Momentary Predictors of Insulin Restriction Among Adults With Type 1 Diabetes and Eating Disorder Symptomatology. *Diabetes care* **38,** 2025–2032; 10.2337/dc15-0753 (2015).

78. Scott, S. B., Ram, N., Smyth, J. M., Almeida, D. M. & Sliwinski, M. J. Age differences in negative emotional responses to daily stressors depend on time since event. *Developmental psychology* **53,** 177–190; 10.1037/dev0000257 (2017).

79. Poppe, L. *et al.* The impact of mental and somatic stressors on physical activity and sedentary behaviour in adults with type 2 diabetes mellitus: a diary study. *PeerJ* **9,** e11579; 10.7717/peerj.11579 (2021).

80. Reichert, M. *et al.* Within-Subject Associations between Mood Dimensions and Non-exercise Activity: An Ambulatory Assessment Approach Using Repeated Real-Time and Objective Data. *Frontiers in Psychology* **0,** 918; 10.3389/FPSYG.2016.00918 (2016).

81. Reichert, M. *et al.* Exercise versus nonexercise activity: E-diaries unravel distinct effects on mood. *Medicine and Science in Sports and Exercise* **49,** 763–773; 10.1249/MSS.0000000000001149 (2017).

82. Reichert, M. *et al.* Erste Schritte zu Smartphone-basierten Sportinterventionen im Alltag? *Zeitschrift für Sportpsychologie* **25,** 156; 10.1026/1612-5010/A000243 (2018).

83. Reininghaus, U. *et al.* Effects of a Novel, Transdiagnostic Ecological Momentary Intervention for Prevention, and Early Intervention of Severe Mental Disorder in Youth (EMIcompass): Findings From an Exploratory Randomized Controlled Trial. *Schizophrenia bulletin* **49,** 592–604; 10.1093/SCHBUL/SBAC212 (2023).

84. Ruissen, G. R. *et al.* Continuous-Time Modeling of the Bidirectional Relationship Between Incidental Affect and Physical Activity. *Annals of behavioral medicine : a publication of the Society of Behavioral Medicine* **56,** 1284–1299; 10.1093/abm/kaac024 (2022).

85. Watson, D., Clark, L. A. & Tellegen, A. Development and validation of brief measures of positive and negative affect: the PANAS scales. *Journal of personality and social psychology* **54,** 1063–1070; 10.1037//0022-3514.54.6.1063 (1988).

86. Watson, D. & Clark, L. The PANAS-X: Manual for the positive and negative affect schedule-expanded form. *Psychology Publications* (1999).

87. Sperry, S. H., Sharpe, B. M. & Wright, A. G. C. Momentary dynamics of emotion-based impulsivity: Exploring associations with dispositional measures of externalizing and internalizing psychopathology. *Journal of abnormal psychology* **130,** 815–828; 10.1037/abn0000720 (2021).

88. Schwerdtfeger, A. & Friedrich-Mai, P. Social interaction moderates the relationship between depressive mood and heart rate variability: evidence from an ambulatory monitoring study. *Health psychology* **28,** 501–509; 10.1037/A0014664 (2009).

89. Schwerdtfeger, A. R., Friedrich-Mai, P. & Gerteis, A. K. S. Daily Positive Affect and Nocturnal Cardiac Activation. *International journal of behavioral medicine* **22,** 132–138; 10.1007/S12529-014-9396-4 (2015).

90. Schwerdtfeger, A., Eberhardt, R., Chmitorz, A. & Schaller, E. Momentary affect predicts bodily movement in daily life: an ambulatory monitoring study. *Journal of sport & exercise psychology* **32,** 674–693; 10.1123/JSEP.32.5.674 (2010).

91. Schwerdtfeger, A. R. & Scheel, S. M. Self-esteem fluctuations and cardiac vagal control in everyday life. *International journal of psychophysiology* **83,** 328–335; 10.1016/J.IJPSYCHO.2011.11.016 (2012).

92. Schwerdtfeger, A. R. & Gerteis, A. K. S. The manifold effects of positive affect on heart rate variability in everyday life: distinguishing within-person and between-person associations. *Health psychology* **33,** 1065–1073; 10.1037/hea0000079 (2014).

93. Krohne, H. W., Egloff, B., Kohlmann, C.-W. & Tausch, A. Positive and Negative Affect Schedule--German Version (PANAS) . *APA PsycTests*; 10.1037/t49650-000 (1996).

94. Schwerdtfeger, A. R. & Rathner, E. M. The ecological validity of the autonomic-subjective response dissociation in repressive coping. *Anxiety, stress, and coping* **29,** 241–258; 10.1080/10615806.2015.1048237 (2016).

95. Schwerdtfeger, A. R. & Dick, K. Episodes of momentary resilience in daily life are associated with HRV reductions to stressful operations in firefighters: An ambulatory assessment approach using bayesian multilevel modeling. *The Journal of Positive Psychology,* 593–602 (2019).

96. Wagnild, G. M. & Young, H. M. Development and psychometric evaluation of the Resilience Scale. *Journal of nursing measurement* **1,** 165–178 (1993).

97. Seiferth, C. *et al.* Bi-directional associations of core affect and physical activity in adults with higher body weight: An ecological momentary assessment study. *Journal of Health Psychology* **29,** 1115–1128; 10.1177/13591053241228202 (2024).

98. Smith, K. E. *et al.* Associations between naturalistically assessed physical activity patterns, affect, and eating in youth with overweight and obesity. *Journal of behavioral medicine* **43,** 916–931; 10.1007/s10865-020-00152-3 (2020).

99. Watson, D., Wiese, D., Vaidya, J. & Tellegen, A. The two general activation systems of affect: Structural findings, evolutionary considerations, and psychobiological evidence. *Journal of personality and social psychology* **76,** 820–838; 10.1037/0022-3514.76.5.820 (1999).

100. Stevenson, B. L. *et al.* Using Ecological Momentary Assessments and Fitbit Data to Examine Daily Associations Between Physical Activity, Affect and Alcohol Cravings in Patients with Alcohol Use Disorder. *International journal of behavioral medicine* **29,** 543–552; 10.1007/s12529-021-10039-5 (2022).

101. Sudeck, G., Jeckel, S. & Schubert, T. Individual Differences in the Competence for Physical-Activity-Related Affect Regulation Moderate the Activity-Affect Association in Real-Life Situations. *Journal of sport 5&6 exercise psychology* **40,** 196–205; 10.1123/jsep.2018-0017 (2018).

102. Takano, K., Sakamoto, S. & Tanno, Y. Ruminative self-focus in daily life: associations with daily activities and depressive symptoms. *Emotion (Washington, D.C.)* **13,** 657–667; 10.1037/a0031867 (2013).

103. Vetrovsky, T. *et al.* Morning fatigue and structured exercise interact to affect non-exercise physical activity of fit and healthy older adults. *BMC geriatrics* **21,** 179; 10.1186/s12877-021-02131-y (2021).

104. Mendoza, T. R. *et al.* The rapid assessment of fatigue severity in cancer patients. *Cancer* **85,** 1186–1196; 10.1002/(SICI)1097-0142(19990301)85:5<1186::AID-CNCR24>3.0.CO;2-N (1999).

105. Williams, D. R., Martin, S. R., Liu, S. & Rast, P. Bayesian Multivariate Mixed-Effects Location Scale Modeling of Longitudinal Relations Among Affective Traits, States, and Physical Activity. *European journal of psychological assessment : official organ of the European Association of Psychological Assessment* **36,** 981–997; 10.1027/1015-5759/a000624 (2020).

106. Yang, C.-H. *et al.* Mother-child dyadic influences of affect on everyday movement behaviors: evidence from an ecological momentary assessment study. *The international journal of behavioral nutrition and physical activity* **17,** 56; 10.1186/s12966-020-00951-6 (2020).

107. Wen, C. K. F. *et al.* Relationships among affective states, physical activity, and sedentary behavior in children: Moderation by perceived stress. *Health psychology : official journal of the Division of Health Psychology, American Psychological Association* **37,** 904–914; 10.1037/hea0000639 (2018).

108. Dunton, G. F. *et al.* How acute affect dynamics impact longitudinal changes in physical activity among children. *Journal of behavioral medicine* **45,** 451–460; 10.1007/S10865-022-00282-W/METRICS (2022).

109. Kanning, M. *et al.* Doing exercise or sport together with one's child is positively associated with mothers' momentary affect in daily life, but not with higher levels of overall physical activity. *BMC Public Health* **20,** 715; 10.1186/s12889-020-08864-6 (2020).

110. Zarbo, C. *et al.* Ecological monitoring of physical activity, emotions and daily life activities in schizophrenia: the DiAPAson study. *BMJ mental health* **26**; 10.1136/BMJMENT-2023-300836 (2023).

111. Zhaoyang, R. & Martire, L. M. Daily Sedentary Behavior Predicts Pain and Affect in Knee Arthritis. *Annals of behavioral medicine : a publication of the Society of Behavioral Medicine* **53,** 642–651; 10.1093/abm/kay073 (2019).

112. Thomas, D. L. & Diener, E. Memory accuracy in the recall of emotions. *Journal of personality and social psychology* **59,** 291–297; 10.1037/0022-3514.59.2.291 (1990).

113. Rosseel, Y. lavaan: An R Package for Structural Equation Modeling. *Journal of Statistical Software* **48,** 1–36; 10.18637/JSS.V048.I02 (2012).

114. Viechtbauer, W. Conducting Meta-Analyses in R with the metafor Package. *Journal of Statistical Software* **36**; 10.18637/JSS.V036.I03 (2010).

115. Pustejovsky, J. E. & Tipton, E. Meta-analysis with Robust Variance Estimation: Expanding the Range of Working Models. *Prevention Science* **23,** 425–438; 10.1007/S11121-021-01246-3/METRICS (2022).

116. Nakagawa, S. *et al.* orchaRd 2.0: An R package for visualising meta-analyses with orchard plots. *Methods in Ecology and Evolution* **14,** 2003–2010; 10.1111/2041-210X.14152 (2023).

117. Olfermann, R. Accelerometer Data. Available at https://olfermann.shinyapps.io/accel/.

118. Burchartz, A. *et al.* Assessing physical behavior through accelerometry – State of the science, best practices and future directions. *Psychology of Sport and Exercise* **49**; 10.1016/J.PSYCHSPORT.2020.101703 (2020).

119. Bolger, N. & Laurenceau, J.-P. *Intensive Longitudinal Methods: An Introduction to Diary and Experience Sampling Research* (The Guilford Press, 2013).

120. Hox, J. J., Moerbeek, M. & van de Schoot, R. *Multilevel Analysis: Techniques and Applications* (2017).

121. Ben-Shachar, M. S., Lüdecke, D. & Makowski, D. effectsize: Estimation of Effect Size Indices and Standardized Parameters. *Journal of Open Source Software* **5,** 2815; 10.21105/JOSS.02815 (2020).

122. Wickham, H. *ggplot2. Elegant Graphics for Data Analysis* (Springer International Publishing, 2016).

123. Lüdecke, D. ggeffects: Tidy Data Frames of Marginal Effects from Regression Models. *Journal of Open Source Software* **3,** 772; 10.21105/JOSS.00772 (2018).

124. Liddell, T. M. & Kruschke, J. K. Analyzing ordinal data with metric models: What could possibly go wrong? *Journal of Experimental Social Psychology* **79,** 328–348; 10.1016/J.JESP.2018.08.009 (2018).

125. Christensen, R. H. B. *Regression Models for Ordinal Data [R package ordinal version 2023.12-4.1]*. Available at https://CRAN.R-project.org/package=ordinal (Comprehensive R Archive Network (CRAN), 2024).

126. Modugno, L. & Giannerini, S. The Wild Bootstrap for Multilevel Models. *Communications in Statistics - Theory and Methods* **44,** 4812–4825; 10.1080/03610926.2013.802807 (2015).

127. Loy, A., Steele, S. & Korobova, J. *lmeresampler: Bootstrap Methods for Nested Linear Mixed-Effects Models_. R package version 0.2.4*. Available at https://cran.r-project.org/web/packages/lmeresampler/index.html (2023).

128. Killingsworth, M. A. & Gilbert, D. T. A wandering mind is an unhappy mind. *Science* **330,** 932; 10.1126/SCIENCE.1192439 (2010).

129. Russel, J. A. Core affect and the psychological construction of emotion. *Psychological Review* **110,** 145–172 (2003).

130. Barrett, L. F. Valence is a basic building block of emotional life. *Journal of Research in Personality* **40,** 35–55; 10.1016/j.jrp.2005.08.006 (2006).

131. Scherer, K. R. What are emotions? And how can they be measured? *Social Science Information* **44,** 695–729; 10.1177/0539018405058216 (2005).

132. Cunningham, W. A., Dunfield, K. A. & Stillman, P. E. Emotional States from Affective Dynamics. *Emotion Review* **5,** 344–355; 10.1177/1754073913489749 (2013).

133. Ekkekakis, P. The measurement of affect, mood, and emotion: A guide for health-behavioral research. *The Measurement of Affect, Mood, and Emotion: A Guide for Health-Behavioral Research*; 10.1017/CBO9780511820724 (2013).

134. Williams, D. M. & Evans, D. R. Current Emotion Research in Health Behavior Science. *Emotion Review* **6,** 277–287; 10.1177/1754073914523052 (2014).

135. Barrett, L. F. The theory of constructed emotion: an active inference account of interoception and categorization. *Social cognitive and affective neuroscience* **12,** 1833; 10.1093/scan/nsx060 (2017).

136. Kuppens, P. & Verduyn, P. Emotion dynamics. *Current opinion in psychology* **17,** 22–26; 10.1016/j.copsyc.2017.06.004 (2017).

137. Scarantino, A. (ed.). *Emotion Theory: The Routledge Comprehensive Guide* (Routledge, New York, 2024).

138. Russell, J. A. & Barrett, L. F. Core affect, prototypical emotional episodes, and other things called emotion: dissecting the elephant. *Journal of personality and social psychology* **76,** 805–819; 10.1037//0022-3514.76.5.805 (1999).

139. Buecker, S., Simacek, T., Ingwersen, B., Terwiel, S. & Simonsmeier, B. A. Physical activity and subjective well-being in healthy individuals: a meta-analytic review. *Health Psychology Review* **15,** 574–592; 10.1080/17437199.2020.1760728 (2021).

140. Dukes, D. *et al.* The rise of affectivism. *Nature Human Behaviour 2024 8:6* **5,** 816–820; 10.1038/s41562-021-01130-8 (2021).

141. Russell, J. A. Core affect and the psychological construction of emotion. *Psychological Review* **110,** 145–172; 10.1037/0033-295X.110.1.145 (2003).

142. Thayer, R. E. *The biopsychology of mood and activation* (Oxford University Press., New York, 1989).

143. Watson, D. & Tellegen, A. Toward a consensual structure of mood. *Psychological bulletin* **98,** 219–235; 10.1037//0033-2909.98.2.219 (1985).

144. Morris, W. N. *Mood. The Frame of Mind* (Springer New York, New York, NY, 1989).

145. Russell, J. A. A circumplex model of affect. *Journal of personality and social psychology* **39,** 1161–1178; 10.1037/H0077714 (1980).

146. Matthews, G., Jones, D. M. & Chamberlain, A. G. Refining the measurement of mood: The UWIST Mood Adjective Checklist. *British J of Psychology* **81,** 17–42; 10.1111/j.2044-8295.1990.tb02343.x (1990).

147. Steyer, R., Schwenkmezger, P., Notz, P. & Eid, M. Testtheoretische Analysen des Mehrdimensionalen Befindlichkeitsfragebogen (MDBF). *Diagnostica* (1994).

148. Schimmack, U. & Grob, A. Dimensional models of core affect: a quantitative comparison by means of structural equation modeling. *Eur. J. Pers.* **14,** 325–345; 10.1002/1099-0984(200007/08)14:4<325::AID-PER380>3.0.CO;2-I (2000).

149. Ekman, P. Basic Emotions. In *Handbook of Cognition and Emotion,* edited by T. Dalgleish & M. J. Power (Wiley1999), pp. 45–60.

150. Lange, J. & Zickfeld, J. H. Emotions as Overlapping Causal Networks of Emotion Components: Implications and Methodological Approaches. *Emotion Review* **13,** 157–167; 10.1177/1754073920988787 (2021).

151. Scherer. Klaus R, Schorr, A. & Johnstone, T. (eds.). *Appraisal Processes in Emotion: Theory, Methods, Research* (Oup Usa, 2001).

152. Moors, A., Boddez, Y. & Houwer, J. de. The Power of Goal-Directed Processes in the Causation of Emotional and Other Actions. *Emotion Review* **9,** 310–318; 10.1177/1754073916669595 (2017).

153. Barrett, L. F. & Russell, J. A. (eds.). *The psychological construction of emotion* (Guilford Press, New York, London, 2015).

154. Mesquita, B. & Parkinson, B. Social Constructionist Theories of Emotions. In *Emotion Theory: The Routledge Comprehensive Guide,* edited by A. Scarantino (Routledge, New York, 2024), pp. 388–407.

155. Larsen, R. J., Diener, E. Promises and problems with the circumplex model of emotion. *Emotion: Review of Personality and Social Psychology (Band 13),* 25–59 (1992).

156. Brose, A., Schmiedek, F., Gerstorf, D. & Voelkle, M. C. The measurement of within-person affect variation. *Emotion (Washington, D.C.)* **20,** 677–699; 10.1037/emo0000583 (2020).

157. Cloos, L., Ceulemans, E. & Kuppens, P. Development, validation, and comparison of self-report measures for positive and negative affect in intensive longitudinal research. *Psychological assessment* **35,** 189–204; 10.1037/pas0001200 (2023).

158. Ekkekakis, P. & Zenko, Z. Measurement of Affective Responses to Exercise. In *Emotion Measurement* (Elsevier2016), pp. 299–321.

159. Kanning, M. K., Ebner-Priemer, U. W. & Schlicht, W. M. How to Investigate Within-Subject Associations between Physical Activity and Momentary Affective States in Everyday Life: A Position Statement Based on a Literature Overview. *Frontiers in Psychology* **4,** 187; 10.3389/fpsyg.2013.00187 (2013).

160. Russell, J. A. Emotion, core affect, and psychological construction. *Cognition and Emotion* **23,** 1259–1283; 10.1080/02699930902809375 (2009).

161. Yik, M. S. M., Russell, J. A. & Barrett, L. F. Structure of self-reported current affect: Integration and beyond. *Journal of personality and social psychology* **77,** 600–619; 10.1037/0022-3514.77.3.600 (1999).

162. Steyer, R., Schwenkmezger, P., Notz, P. & Eid, M. Entwicklung des Mehrdimensionalen Befindlichkeitsfragebogens (MDBF). Primärdatensatz, 2003.

163. Packheiser, J. *et al.* A systematic review and multivariate meta-analysis of the physical and mental health benefits of touch interventions. *Nature Human Behaviour 2024 8:6* **8,** 1088–1107; 10.1038/s41562-024-01841-8 (2024).
