# Supplementary material for: An individual participant data meta-analysis of how physical activity relates to affective well-being in daily life
Source: Nat Hum Behav. 2026 May 6;10(7):1297–315. doi: 10.1038/s41562-026-02427-2 (PMC13388107; doi:10.1038/s41562-026-02427-2)
Supplement: Supplementary file 2 — Reporting Summary [file 41562_2026_2427_MOESM2_ESM.pdf]

nature portfolio

Corresponding author(s): Markus Reichert

Last updated by author(s): Feb 8, 2026

Reporting Summary

Nature Portfolio wishes to improve the reproducibility of the work that we publish. This form provides structure for consistency and transparency in reporting. For further information on Nature Portfolio policies, see our Editorial Policies and the Editorial Policy Checklist.

Statistics

For all statistical analyses, confirm that the following items are present in the figure legend, table legend, main text, or Methods section.

n/a	Confirmed	
☐	☒	The exact sample size (n) for each experimental group/condition, given as a discrete number and unit of measurement
☐	☒	A statement on whether measurements were taken from distinct samples or whether the same sample was measured repeatedly
☐	☒	The statistical test(s) used AND whether they are one- or two-sided<br>*Only common tests should be described solely by name; describe more complex techniques in the Methods section.*
☐	☒	A description of all covariates tested
☐	☒	A description of any assumptions or corrections, such as tests of normality and adjustment for multiple comparisons
☐	☒	A full description of the statistical parameters including central tendency (e.g. means) or other basic estimates (e.g. regression coefficient) AND variation (e.g. standard deviation) or associated estimates of uncertainty (e.g. confidence intervals)
☐	☒	For null hypothesis testing, the test statistic (e.g. F, t, r) with confidence intervals, effect sizes, degrees of freedom and P value noted<br>*Give P values as exact values whenever suitable.*
☒	☐	For Bayesian analysis, information on the choice of priors and Markov chain Monte Carlo settings
☐	☒	For hierarchical and complex designs, identification of the appropriate level for tests and full reporting of outcomes
☒	☐	Estimates of effect sizes (e.g. Cohen's d, Pearson's r), indicating how they were calculated

Our web collection on statistics for biologists contains articles on many of the points above.

Software and code

Policy information about availability of computer code

Data collection	n/a
Data analysis	All preprocessing, harmonization, checking, merging, and analyzing was performed using R (version 4.4.0 and version 4.5.2) and the interface RStudio. Used packages were tidyverse (version 2.0.0), esmpack (version 0.1.20), lme4 (version 1.1.35.3), lmerTest (version 3.1.3), effectsize (0.8.7), lavaan (0.6.17), metafor (version 4.8.0), RoBMA (version 2.3.3), orchaRd (version 2.1), ordinal (2023.12.4), lmeresampler (version 0.2.4), ggeffects (version 1.5.2), sjPlot (version 2.8.15), performance (version 0.11.0), ggpubr (version 0.6.0), janitor (version 2.2.0), rio (version 1.0.1), gridExtra (version 2.3). Analysis code can be accessed at https://osf.io/2tn8u

For manuscripts utilizing custom algorithms or software that are central to the research but not yet described in published literature, software must be made available to editors and reviewers. We strongly encourage code deposition in a community repository (e.g. GitHub). See the Nature Portfolio guidelines for submitting code & software for further information.

Data

Policy information about availability of data

All manuscripts must include a data availability statement. This statement should provide the following information, where applicable:

- Accession codes, unique identifiers, or web links for publicly available datasets
- A description of any restrictions on data availability
- For clinical datasets or third party data, please ensure that the statement adheres to our policy

For license and ethical reasons, IPD cannot be made publicly available. Data from the individual studies needs to be requested from the respective data contributor. To facilitate data requests, a list of included datasets is publicly available (https://osf.io/2tn8u). Further, we are happy to assist in establishing contact with the data contributors.

Research involving human participants, their data, or biological material

Policy information about studies with human participants or human data. See also policy information about sex, gender (identity/presentation), and sexual orientation and race, ethnicity and racism.

Reporting on sex and gender	If available from primary studies, we included gender (instead of sex) and a non-binary category in our analyses (covariate, moderator). However, if there was no information on gender in primary studies, we relied on information on participants' sex.
Reporting on race, ethnicity, or other socially relevant groupings	We used the study location as a proxy for cultural background and included it as moderator in our analyses. Factors such as race or ethnicity were not consistently available for all datasets, so that it was not possible to conduct a moderator analysis on race, ethnicity, or other socially relevant groupings in the present individual participant data meta-analysis.
Population characteristics	See below. Consistently available characteristics of samples/participants were extracted and used as moderators in the present individual participant data meta-analysis, see Table 1 and Supplementary Materials S1.
Recruitment	For the present analysis, no participants were recruited but previously collected data was combined.
Ethics oversight	An ethics exemption was obtained from the ethics committee at the Faculty of Sport Science, Ruhr University Bochum, Germany, and based on the ethics approvals of individual studies included in the meta-analysis (reference: EKS S 03/2022).

Note that full information on the approval of the study protocol must also be provided in the manuscript.

Field-specific reporting

Please select the one below that is the best fit for your research. If you are not sure, read the appropriate sections before making your selection.

☐ Life sciences ☒ Behavioural & social sciences ☐ Ecological, evolutionary & environmental sciences

For a reference copy of the document with all sections, see nature.com/documents/nr-reporting-summary-flat.pdf

Behavioural & social sciences study design

All studies must disclose on these points even when the disclosure is negative.

Study description	The present study is a quantitative individual participant data meta-analysis with three analysis levels (two-stage individual participant data meta-analysis, one-stage individual participant data meta-analysis, and subgroup one-stage individual participant data meta-analysis) investigating the bidirectional associations of physical activity and affective well-being in everyday life.
Research sample	Individual participant data from k = 67 existing datasets including n = 8,223 participants (mean age = 36.33, SD = 18.11; 54.93% female, 0.06% non-binary) from 14 countries world wide was combined. We included participants of all ages and independent of their clinical status in our analyses. We only restricted the sample to participants living in their natural conditions, i.e., interventions or in-patient treatments were considered as exclusion criteria. Hence, we consider our sample to be highly representative. We investigated heterogeneity by moderator analyses. The individual datasets are described in detail in Supplementary Materials S1 and a list of dataset sources can be found on OSF (https://osf.io/2tn8u)
Sampling strategy	n/a, we did not collect data from participants ourselves but combined existing datasets in an individual participant data meta-analysis, thus, there was no sample size calculation or sampling procedure for participants; To identify published studies for the meta-analysis, the databases Web of Science, PubMed, Scopus, SPORTDiscus, and PsycINFO were searched till December 2023. The five databases were systematically searched by the terms “ecological momentary assessment”, “mood”, “physical activity” and “sedentary behavior” as well as their respective synonyms: "physical activity" or "exercise" or "sedentary behavior" or "sedentariness" or "physical inactivity" plus "mood" or "emotion" or "affect" or "affective states" or "valence" or "calmness" or "energetic arousal" plus "ambulatory assessment" or "ecological momentary assessment" or "experience sampling method" or "electronic sampling method" or "ambulatory monitoring" or "accelerometry" or "physical activity monitoring" or "interactive assessment" or "e-diary" or "electronic diary".

Data collection

na/, we did not collect data from participants ourselves but combined existing datasets in an individual participant data meta-analysis. Corresponding authors of selected studies were contacted via email for data contribution. If authors did not respond within six weeks after first contact, no further reminders were sent. Authors interested in contributing their data received an exemplary data sheet, a letter of understanding, and a link to a password-secured file-drop folder. However, other formats of data transfer were also accepted. If necessary, university agreements for data sharing were arranged.
The variables to be included on study, participant, and measurement level were chosen upon intensive discussion in the coordinating team (JR, IT, GB, MG, JP, MR). For variables on study level, a data extraction template was custom-developed to extract data on: authors, country, detailed participant characteristics, AWB assessment, PA assessment, assessment duration (number of days participants were instructed to wear accelerometers and fill out e-diaries) and assessment frequency (number of e-diary prompts sent per day). Two researchers (IT, MG) independently extracted the relevant information from included records. Thereafter, the two files were merged. Any discrepancies were discussed among the two authors until an agreement was reached, and in case of persistent non-agreement between the two researchers (IT, MG) a third reviewer (MR) was consulted to reach a decision. For participant and measurement characteristics, all contributed data files were inspected individually by JR. Relevant variables were renamed and recoded if necessary in order to harmonize all data sets following a predefined data mask.

Timing

The databases were searched until December 2023. Data collection (i.e., requesting data from authors and including it in our analysis) was carried out from March 2022 until October 2024.

Data exclusions

Exclusion criteria were detailed in the pre-registration prior to study onset. Study exclusions are listed in detail in the flowchart and the Methods section. Specifically, two datasets needed to be excluded. In one dataset, PA and AWB were only available for exercise bouts, not for time slots preceding or following the e-diary prompt. The other only included one aggregate measure of PA per participant so that the within-associations of PA and AWB could not be investigated. During preprocessing, we excluded all rows of data with neither PA nor AWB data, reducing the dataset from i = 380,349 to i = 369,828. We also excluded participants with a standard deviation of 0 in all AWB scores which we used as an indicator of careless responding to EMA prompts. This was not pre-specified. This led to the exclusion of 27 participants (0.33%)/477 measurements (0.13%).

Non-participation

No participants were involved in the present study as we conducted an individual participant data meta-analysis of existing data.

Randomization

n/a; no intervention but correlative design

Reporting for specific materials, systems and methods

We require information from authors about some types of materials, experimental systems and methods used in many studies. Here, indicate whether each material, system or method listed is relevant to your study. If you are not sure if a list item applies to your research, read the appropriate section before selecting a response.

Materials & experimental systems

n/a	Involved in the study
☒	☐ Antibodies
☒	☐ Eukaryotic cell lines
☒	☐ Palaeontology and archaeology
☒	☐ Animals and other organisms
☒	☐ Clinical data
☒	☐ Dual use research of concern
☒	☐ Plants

Methods

n/a	Involved in the study
☒	☐ ChIP-seq
☒	☐ Flow cytometry
☒	☐ MRI-based neuroimaging

Plants

Seed stocks

Report on the source of all seed stocks or other plant material used. If applicable, state the seed stock centre and catalogue number. If plant specimens were collected from the field, describe the collection location, date and sampling procedures.

Novel plant genotypes

Describe the methods by which all novel plant genotypes were produced. This includes those generated by transgenic approaches, gene editing, chemical/radiation-based mutagenesis and hybridization. For transgenic lines, describe the transformation method, the number of independent lines analyzed and the generation upon which experiments were performed. For gene-edited lines, describe the editor used, the endogenous sequence targeted for editing, the targeting guide RNA sequence (if applicable) and how the editor was applied.

Authentication

Describe any authentication procedures for each seed stock used or novel genotype generated. Describe any experiments used to assess the effect of a mutation and, where applicable, how potential secondary effects (e.g. second site T-DNA insertions, mosiacism, off-target gene editing) were examined.
