# Supplementary material for: An individual participant data meta-analysis of how physical activity relates to affective well-being in daily life
Source: Nat Hum Behav. 2026 May 6;10(7):1297–315. doi: 10.1038/s41562-026-02427-2 (PMC13388107; doi:10.1038/s41562-026-02427-2)
Supplement: Supplementary file 3 — Peer Review File [file 41562_2026_2427_MOESM3_ESM.pdf]

Peer Review File

An individual participant data meta-analysis of how physical activity relates to affective well-being in daily life

Corresponding Author: Professor Markus Reichert

Version 0:

Decision Letter:

18th August 2025

Dear Professor Reichert,

Thank you once again for your manuscript, entitled "Physical activity and affective well-being in daily life: An individual participant data meta-analysis of 321,345 ambulatory ratings and accelerometry", and for your patience during the peer review process.

Your Article has now been evaluated by 3 referees. You will see from their comments copied below that, although they find your work of considerable potential interest, they have raised quite substantial concerns. In light of these comments, we cannot accept the manuscript for publication, but would be interested in considering a revised version if you are willing and able to fully address reviewer and editorial concerns.

We hope you will find the referees' comments useful as you decide how to proceed. If you wish to submit a substantially revised manuscript, please bear in mind that we will be reluctant to approach the referees again in the absence of major revisions. We are committed to providing a fair and constructive peer-review process. Do not hesitate to contact us if there are specific requests from the reviewers that you believe are technically impossible or unlikely to yield a meaningful outcome.

To guide the scope of the revisions, the editors discuss the referee reports in detail within the team, including with the chief editor, with a view to (1) identifying key priorities that should be addressed in revision and (2) overruling referee requests that are deemed beyond the scope of the current study. We hope that you will find the prioritised set of referee points to be useful when revising your study. Please do not hesitate to get in touch if you would like to discuss these issues further.

Reviewer 2 raises a major concern about the merging of conceptually dissimilar concepts, and the absence of a discussion / consideration of measures and their downsides, or a clear conceptual framework that guides the work. These are fundamental concerns that need to be addressed. We ask you, however, not to remove your current pre-registered analyses. Rather, we want you to present additional analyses that supplement your existing ones and address the concerns Reviewer 2 raises, alongside all other reviewer feedback.

Finally, your revised manuscript must comply fully with our editorial policies and formatting requirements. Failure to do so will result in your manuscript being returned to you, which will delay its consideration. To assist you in this process, I have attached a checklist that lists all of our requirements. If you have any questions about any of our policies or formatting, please don't hesitate to contact me.

If you wish to submit a suitably revised manuscript, we would hope to receive it within 4 months. I would be grateful if you could contact us as soon as possible if you foresee difficulties with meeting this target resubmission date.

With your revision, please:

• Include a “Response to the editors and reviewers” document detailing, point-by-point, how you addressed each editor and referee comment. If no action was taken to address a point, you must provide a compelling argument. When formatting this document, please respond to each reviewer comment individually, including the full text of the reviewer comment verbatim followed by your response to the individual point. This response will be used by the editors to evaluate your revision and sent back to the reviewers along with the revised manuscript.

• Highlight all changes made to your manuscript or provide us with a version that tracks changes.

Please use the link below to submit your revised manuscript and related files:

Link Redacted

Note: This URL links to your confidential home page and associated information about manuscripts you may have submitted, or

that you are reviewing for us. If you wish to forward this email to co-authors, please delete the link to your homepage.

Thank you for the opportunity to review your work. Please do not hesitate to contact me if you have any questions or would like to discuss the required revisions further.

Sincerely,

[redacted]

[redacted]
Nature Human Behaviour

Reviewer expertise:

Reviewer #1: physical activity and well-being

Reviewer #2: physical activity and well-being ; accelerometry

Reviewer #3: physical activity and well-being ; accelerometry ; IPD- MA

REVIEWER COMMENTS:

Reviewer #1 (Remarks to the Author):

This paper is a substantial amount of work, and the authors are to be congratulated. Below are some comments for the authors to consider.

Methods
I am curious about the rationale to do complete-case analysis (for PA and AWB) rather than impute. The range of missing data (described around ln 578) seems relatively small and seems like a good candidate for imputation?

I have two concerns regarding the risk of bias/study quality assessment.
First, I'm unconvinced that a tool specific to EMA is needed. The more general tools (e.g., those by JBI, Cochrane) already have items regarding the quality of the measure, and you could have simply included specific rules as part of the operationalisation of these items. Using standard assessment tools provides better information than a more niche tool.
My second concern is that the tool is not an assessment of risk of bias, but instead of study quality. This seems to be an error in the original paper, but the tool used could not accurately be described as a 'risk of bias' tool. It is a quality assessment tool. Many of the items measured present no obvious link to a potential biases (e.g., titles, rationales), and so it would be inaccurate to describe the tool as assessing bias. I'd also note that Cochrane specifically warn against aggregate scores for RoB or quality assessment tools, because it infers that each item in the tool is weighted equally or is equally likely to cause a bias with an equal likelihood of impacting the results. This clearly untrue, and so aggregate scores should be avoided. I appreciate that the original paper cited describes thresholds, but this is a mistake in my opinion and not one that needs to be propagated through the literature.
So I have two specific suggestions: switch to a general purpose tool which is more widely used OR change the reporting to more clearly be described as quality assessment and provide the ratings within each item, removing the aggregate scores.

I'm trying to understand why in the antecedent model for the one-stage IPD that you would need to include both a mean centered PA variable and the person-mean PA variable. The first gives you how much their PA varies from their usual PA for the next AWB measure, and the latter the persons overall average PA. I'm a little confused by the hypothesis the inclusion of the second variable is testing: if a person is more active than their normal level you'd expect their AWB to be higher, but this effect my depend on their total physical activity? I have no doubt that the authors have thought deeply about this, and I'd just like a little additional explanation.

For the most part, I am impressed by the analyses. They seem robust, and aligned with the registered plan.

Results
I may have missed it, but the PRISMA-IPD checklists suggests "Where applicable, also report similar study characteristics for any studies not providing IPD" (item 18). It would be interesting to know if there are any systematic differences between the studies you were able to get data from vs not.

I found Figure 6 quite confusing. I'm not completely sure it adds anything.

I am struggling to see where the numbers from lines 342 onwards come from. The part I am having trouble reconciling is how we have gone from a correlation of 0.05 (Fig 2) or a standardised beta of 0.05 (Fig 3) for positive affect, to an effect size which is described as "considerable". Part of the reason I think I am finding it hard to parse this, is because the results given in the text are somehow on a scale of 0-100, which the numbers used in Table 1 are 1-4. If I am reading table S2.2a correctly, a full standard deviation increase in person-mean physical activity (which seems large) would be associated with a 0.09 unit increase on the 1-4 positive affect scale. That does not seem "considerable". The comparisons made also don't really suggest very large effects: it's consistent with other 'daily life activities'. This would not suggest that physical activity is a panacea. I'd appreciate either clearer explanation for why the authors feel that these are considerable effects sizes, or a tempering of the language.

Reviewer #2 (Remarks to the Author):

This is an interesting submission that entailed an individual participant data (IPD) meta-analysis into physical activity and affective responses. There is a lot to like about the paper, such as the pre-registered PRISMA-informed methodology, the use of device-based accelerometry data and the associated one- and two-stage IPD analyses. While the topic of the paper is likely to be of interest to Nature Human Behavior's readership, I have the following concerns about the submission.

A limitation of affective psychology research is that the terms core affect, emotion, and mood are often used interchangeably without conceptual differentiation (Russell & Feldman Barrett, 1999). Unfortunately, it appears that you often fall into this trap by conflating key affective phenomena terminology. The rationale for including "five dimensions of affective well-being that were most frequently assessed in real-life studies on AWB-PA associations" is somewhat problematic – just because a set of constructs are frequently measured, it does necessarily mean that they coherently tap the underlying affective phenomena of interest. It is currently unclear, for example, how positive/negative affect differs from valence, which is typically conceptualized as a bipolar dimension ranging from pleasure to displeasure (Ekkekakis & Zenko, 2016). Likewise, you mentioned that fatigue was "assigned to negative affect" and that "mood [was assigned] to valence". Importantly, these are very different affective constructs, mood being more diffuse/low intensity and valence being part of core affect, constantly accessible to consciousness (Russell, 2009). In addition, mood is suggested to last for days, weeks, or months (Ekkekakis & Zenko, 2016), so it would have been useful if you provided a rationale for why you think mood is amenable to change in relation to a single session of physical activity.

The studies included in the analysis comprise a smorgasbord of affective phenomena without any clear guiding conceptual framework and this is further evidenced by the associated search terms in S12 ("mood* OR emotion* OR affect*"). A related problem, is a lack of consideration toward the measures used in the included studies. S1 reveals that many studies administered the Positive and Negative Affect Schedule (PANAS; Watson et al., 1988), which has been heavily criticized for only tapping high-activation states (Barrett & Russell, 1999). Similarly, other studies appear to have employed a measure of mood proposed by Wilhelm and Schoebi (2007), who note that "moods are not necessarily linked to an obvious cause that can be related to an event and its specific appraisal" (p. 259). Again, a rationale is needed as to why the construct mood is of interest in relation to physical activity.

Casting the net far and wide is good for retaining studies and associated data (indeed, I commend you for amalgamating almost 1 million hours of accelerometry), but these data are considerably messy in relation to the affective phenomena that they tap and your associated categories that you have enforced on them. Unfortunately, this perpetuates conceptual ambiguity. An alternative strategy, would have been to employ the three-step approach advocated by Ekkekakis and Zenko (2016). First, you initially decide upon an appropriate affective construct (core affect, emotion, or mood). Second, you choose and clearly explain the most appropriate theoretical framework for the chosen construct (distinct states vs. dimensional approaches; Ekkekakis, 2013). It looks as though you have a mixture of the two approaches at present (positive affect, negative affect, and valence). Finally, you select the psychometrically strongest measure based on the chosen theoretical framework. Basing your inclusion criteria on psychometrically strong measures would have resulted in far fewer studies, but would have put you on much firmer ground when drawing conclusions from the data.

Reviewer #3 (Remarks to the Author):

This study presents a robust and comprehensive Individual Participant Data (IPD) meta-analysis on the bidirectional relationship between physical activity (PA) and affective well-being (AWB) in people's daily lives. The work is notable for its scope synthesizing 67 unique datasets from 14 countries, covering 8,223 participants, over 321,000 mood ratings, and nearly 1 million hours of accelerometer-measured PA. The paper addresses a key gap in health psychology and behavioral science: understanding the dynamic, within-person effects of physical activity on momentary well-being in real-world contexts. Below are a series of comments intended to help the authors strengthen their manuscript:

1. While heterogeneity is flagged and some moderators are tested (age, gender/sex, BMI, weekday/weekend), the explanation of why certain effects vary remains largely descriptive. Where data permit, the authors could expand discussion of why certain subgroups may respond differently — to guide precision intervention development. The authors could also strengthen interpretation of heterogeneity by linking findings more explicitly to theoretical models (e.g., motivational psychology, social determinants of health).
2. In the discussion section, the authors should more clearly discuss the nuances of important limitations that readers should consider when drawing their own independent interpretations of the results beyond listing them in the limitations section. Primarily around the observational nature, residual confounding potential, between-study variability in instruments/definitions, and limited coverage of certain AWB dimensions in some datasets.
3. The methods section is very detailed, with some elements that could be streamlined or moved to the Supplementary Materials. In its current form, the sheer length risks losing non-specialist readers and obscuring the central methodological insights that broad Nature Human Behaviour readers might seek. The authors may want to consider keeping in the main text only the conceptual and rationale-level description of methods, highlighting why each approach was chosen and how it advances the field. This will improve readability for a multi-disciplinary audience.
4. The manuscript occasionally uses language implying causal inference (e.g., "PA is bi-directionally related," "PA leads to increases"), even though the observational nature of the IPD data precludes definitive causal claims. The authors could replace or qualify causal-sounding terms with wording that reflects association rather than causation (e.g., "is associated with").
5. While the paper is methodologically sound, the framing currently reads as if written primarily for behavioral medicine or exercise psychology specialists. The broader readership of the journal might not immediately see the transdisciplinary implications. The

authors could use the introduction and conclusion to explicitly link how these microtemporal processes contribute to general models of human decision-making and public health strategies.
6. Some figures (e.g., orchard plots, spaghetti plots) are complex and may be challenging for non-specialists to parse quickly. The authors could add simplified visual summaries with plain-language captions alongside the more technical plots. For key results, the authors may want to also consider including a single integrated conceptual diagram showing the bidirectional associations, their relative strength for each AWB dimension, and identified moderators.

Version 1:

Decision Letter:

Our ref: NATHUMBEHAV-25062905A

8th January 2026

Dear Dr. Reichert,

Thank you for submitting your revised manuscript "An individual participant data meta-analysis of physical activity and affective well-being in daily life" (NATHUMBEHAV-25062905A). It has now been seen by the original referees and their comments are below. As you can see, the reviewers find that the paper has improved in revision. We will therefore be happy in principle to publish it in Nature Human Behaviour, pending revisions and compliance with our editorial and formatting guidelines.

Specifially, we ask that you update your search period to be inclusive until the current point in time.

We are now performing detailed checks on your paper and will send you a checklist detailing our editorial and formatting requirements within two weeks. Please do not upload the final materials and make any revisions until you receive this additional information from us.

Please do not hesitate to contact me if you have any questions.

Sincerely,

[redacted]

[redacted]
Nature Human Behaviour

Reviewer #1 (Remarks to the Author):

The authors have done a commendable job responding to my queries. I have no remaining concerns for this paper.

Reviewer #2 (Remarks to the Author):

Thank you for taking the time to comprehensively address my comments from the previous review. I fully appreciate the addition of the sensitivity analyses and feel that the manuscript has been substantially strengthened with greater coverage concerning the difficulties in differentiating between key affective phenomena terminology. Congratulations on a well-executed revision.

Reviewer #3 (Remarks to the Author):

I thank the authors for their thorough and diligent revisions and responses. I have no further comments.

Version 2:

Decision Letter:

Dear Professor Reichert,

We are pleased to inform you that your Article "An individual participant data meta-analysis of how physical activity relates to

affective well-being in daily life", has now been accepted for publication in Nature Human Behaviour.

Authors may need to take specific actions to achieve compliance with funder and institutional open access mandates. If your research is supported by a funder that requires immediate open access (e.g. according to Plan S principles or the NIH public access policy) then you should select the gold OA route, and we will direct you to the compliant route where possible. Because authors warrant under our subscription licensing terms that they haven't committed to licensing any version of their article under a licence inconsistent with the terms of our agreement – including the applicable embargo period – publication under the subscription model isn't suitable for authors whose funders require no embargo.

Once your manuscript is typeset and you have completed the appropriate grant of rights, you will receive a link to your electronic proof via email with a request to make any corrections within 48 hours. If, when you receive your proof, you cannot meet this deadline, please inform us at rjsproduction@springernature.com immediately. Once your paper has been scheduled for online publication, the Nature press office will be in touch to confirm the details.

Acceptance of your manuscript is conditional on all authors' agreement with our publication policies (see http://www.nature.com/nathumbehav/info/gta). In particular your manuscript must not be published elsewhere and there must be no announcement of the work to any media outlet until the publication date (the day on which it is uploaded onto our web site).

If you have posted a preprint on any preprint server, please ensure that the preprint details are updated with a publication reference, including the DOI and a URL to the published version of the article on the journal website.

An online order form for reprints of your paper is available at https://www.nature.com/reprints/author-reprints.html. All co-authors, authors' institutions and authors' funding agencies can order reprints using the form appropriate to their geographical region.

We welcome the submission of potential cover material (including a short caption of around 40 words) related to your manuscript; suggestions should be sent to Nature Human Behaviour as electronic files (the image should be 300 dpi at 210 x 297 mm in either TIFF or JPEG format). Please note that such pictures should be selected more for their aesthetic appeal than for their scientific content, and that colour images work better than black and white or grayscale images. Please do not try to design a cover with the Nature Human Behaviour logo etc., and please do not submit composites of images related to your work. I am sure you will understand that we cannot make any promise as to whether any of your suggestions might be selected for the cover of the journal.

You can now use a single sign-on for all your accounts, view the status of all your manuscript submissions and reviews, access usage statistics for your published articles and download a record of your refereeing activity for the Nature journals.

To assist our authors in disseminating their research to the broader community, our SharedIt initiative provides you with a unique shareable link that will allow anyone (with or without a subscription) to read the published article. Recipients of the link with a subscription will also be able to download and print the PDF.

As soon as your article is published, you will receive an automated email with your shareable link.

In approximately 10 business days you will receive an email with a link to choose the appropriate publishing options for your paper and our Author Services team will be in touch regarding any additional information that may be required.

If you have any questions about our publishing options, costs, Open Access requirements, or our legal forms, please contact ASJournals@springernature.com

We look forward to publishing your paper.

With best regards,

[redacted]

[redacted]
Nature Human Behaviour

P.S. Click on the following link if you would like to recommend Nature Human Behaviour to your librarian http://www.nature.com/subscriptions/recommend.html#forms

** Visit the Springer Nature Editorial and Publishing website at www.springernature.com/editorial-and-publishing-jobs for more information about our career opportunities. If you have any questions please click here.**

Open Access This Peer Review File is licensed under a Creative Commons Attribution 4.0 International License, which permits use, sharing, adaptation, distribution and reproduction in any medium or format, as long as you give appropriate credit to the original author(s) and the source, provide a link to the Creative Commons license, and indicate if changes were made.
In cases where reviewers are anonymous, credit should be given to 'Anonymous Referee' and the source.
The images or other third party material in this Peer Review File are included in the article's Creative Commons license, unless indicated otherwise in a credit line to the material. If material is not included in the article's Creative Commons license and your intended use is not permitted by statutory regulation or exceeds the permitted use, you will need to obtain permission directly from the copyright holder.
To view a copy of this license, visit https://creativecommons.org/licenses/by/4.0/

Dear Editors, Dear Reviewers,

Thank you for the constructive feedback and the time and effort you invested in improving our manuscript. Below you will find a point-by-point response to all comments. The Reviewers' comments are written in blue, our responses in black, and excerpts from the manuscript in grey. Changes in the manuscript are highlighted in orange. We also wish to inform you that we noticed an error in the preprocessing of physical activity data in k = 2 out of k = 67 individual datasets during the revision process. This error led to the wrongful exclusion of some participants in a subset of our analyses. We have since corrected the error. The only significant changes concern associations of valence with physical activity, for which we now found even stronger evidence. Further, we now revised the manuscript to comply with the revision checklist. Accordingly, we also added a shortened abstract (150 words) to the original PRISMA-compliant abstract. All changes are tracked in the manuscript and supplementary materials. We would like to thank the Editorial team and the Reviewers again for the careful review and the constructive feedback that has helped us substantially in improving our manuscript.

REVIEWER COMMENTS:

Reviewer #1 (Remarks to the Author):

This paper is a substantial amount of work, and the authors are to be congratulated. Below are some comments for the authors to consider.

We thank the Reviewer for acknowledging the substantial amount of work associated with our meta-analysis. We are grateful for the Reviewer's thoughtful comments and suggestions, which helped us greatly in revising and improving our manuscript.

Methods

I am curious about the rationale to do complete-case analysis (for PA and AWB) rather than impute. The range of missing data (described around ln 578) seems relatively small and seems like a good candidate for imputation?

We thank the Reviewer for their inquiry. We are happy to present our rationale and the associated considerations for forgoing imputations in our analyses. First, we collected data from Ecological Momentary Assessment (EMA) studies. Time series data from EMA studies crucially deviates from cross-sectional or longitudinal data. Participants in EMA studies are prompted once or multiple times per day to fill out queries. It is highly likely that participants miss some of the prompts or are unable to answer prompts, for example, because they are driving their car. Hence, perfect compliance to all prompts is practically impossible and missing data is expected. Second, the datasets we received were heterogeneous with regard to missing data: Some datasets did not include any information on missed or ignored prompts. Others still contained all missing data. While

the datasets we received can undoubtedly be combined in a complete-case analysis, we assume that imputing missing data in some datasets but not in others might bias the results.

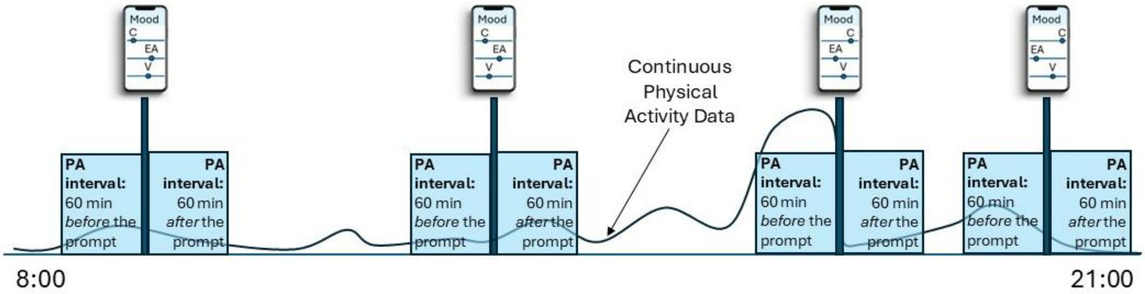

Figure 1. Study design of Ecological Momentary Assessment (EMA) studies investigating the relation of affective well-being (AWB) and physical activity (PA) in daily life. AWB is assessed at multiple discrete time points across a day/multiple days. PA is measured continuously and aggregated over specific time intervals preceding or following the AWB rating.

Third, it is not possible to impute missing data simultaneously across all datasets, as datasets varied in the included variables. All studies provided basic sociodemographic information on participants (i.e., sex/gender and age), for which we controlled in our analyses. Also, they included items on affective well-being in the EMA queries (inclusion criterion). Studies varied, however, in the additional EMA items they queried. That is, there is little consistency with regard to time-varying variables on the within-person level (e.g., time, weekday, social contact, positive or negative events, being indoors versus being outdoors). To adequately impute missing outcome or predictor data on the within-person level, however, we need relevant auxiliary variables. That is, we need to identify time-varying variables that are associated with momentary physical activity and affective well-being in order to set up valid imputation models. The adequacy of imputations heavily depends on the quality of these imputation models. Due to the lack of consistent auxiliary variables across studies, it is not possible to set up an imputation model across datasets.

Setting up individual imputation models for each dataset will introduce critical bias. Due to the different amount and quality of auxiliary variables that can be included in the imputation models of individual studies, the quality of the resulting imputations will tremendously vary across studies. We fear that for some studies the data provided is insufficient to set up adequate imputation models, as relevant predictors of missingness are not included in these datasets. Also, we assume that jointly analysing data based on varying imputation models of variable quality will introduce bias and, thus, weaken our analyses instead of strengthening them.

However, as an alternative to imputation models, we conducted our original analysis using mixed-effects models with restricted maximum likelihood. Maximum likelihood (ML) is a close competitor to multiple imputation (MI). Under identical assumptions, both methods produce estimates that are consistent, asymptotically efficient, and asymptotically normal. Both methods are based on the assumption that data are missing

at random. That is, both assume that missingness is not associated with the true value. Then, the missing data mechanism is said to be ignorable (e.g.,[1]).

Of note, our procedure is in line with prominent prior work: None of the included individual studies of our meta-analysis applied multiple imputations of missing data at prompt-level. Additionally, to our knowledge, there are only two similar IPD meta-analyses on time series data (on alcoholism[2], on self-injury[3]). While Kühn et al. did not discuss the imputation of missing data at all, Dora et al. clearly argue against imputing missing data in individual EMA studies and also report the robustness of MLM in case of missing data.

I have two concerns regarding the risk of bias/study quality assessment.

First, I'm unconvinced that a tool specific to EMA is needed. The more general tools (e.g., those by JBI, Cochrane) already have items regarding the quality of the measure, and you could have simply included specific rules as part of the operationalisation of these items. Using standard assessment tools provides better information than a more niche tool. My second concern is that the tool is not an assessment of risk of bias, but instead of study quality. This seems to be an error in the original paper, but the tool used could not accurately be described as a 'risk of bias' tool. It is a quality assessment tool. Many of the items measured present no obvious link to a potential biases (e.g., titles, rationales), and so it would be inaccurate to describe the tool as assessing bias. I'd also note that Cochrane specifically warn against aggregate scores for RoB or quality assessment tools, because it infers that each item in the tool is weighted equally or is equally likely to cause a bias with an equal likelihood of impacting the results. This clearly untrue, and so aggregate scores should be avoided. I appreciate that the original paper cited describes thresholds, but this is a mistake in my opinion and not one that needs to be propagated through the literature.

So I have two specific suggestions: switch to a general purpose tool which is more widely used OR change the reporting to more clearly be described as quality assessment and provide the ratings within each item, removing the aggregate scores.

We agree with the Reviewer that the tool we applied assesses study quality, not risk of bias, and apologise for mixing this up in our manuscript. Based on the Reviewer's justified criticism, we now present the ratings within each item of the quality assessment tool (see S17). Further, we emphasise in our manuscript that the quality assessment tool should not be confused with a tool for assessing the risk of bias. It is intended as a supplementary and EMA-specific assessment of study quality, focusing more closely on the reporting in publications of EMA studies.

Second, we thank the Reviewer for their assessment that a general risk of bias tool can be applied to our data. We followed the Reviewer's suggestion and applied the ROBINS-E risk of bias tool to the included studies. Results can be found in S16.

Methods (p. 16): To assess risk of bias in individual studies, we applied the ROBINS-E tool[155]. Ratings were conducted and discussed by three researchers (IT, JR, MG) and are presented in S16. Further, we also employed the modified Quality Assessment (QA) tool developed by Timm et al.[16] as a quality assessment tool specifically developed for ambulatory assessment studies on physical activity and affective well-being. The modified QA tool includes categories such as accelerometer technology employed, e-diary sampling schema applied, and compliance rates received. It consists of 16 questions (i.e., the total score equals 16). For the current meta-analysis, all included records' quality was assessed independently by two researchers (IT, MG) and divergent ratings were rated by a third author (MR). Ratings can be found in S17.

I'm trying to understand why in the antecedent model for the one-stage IPD that you would need to include both a mean centered PA variable and the person-mean PA variable. The first gives you how much their PA varies from their usual PA for the next AWB measure, and the latter the persons overall average PA. I'm a little confused by the hypothesis the inclusion of the second variable is testing: if a person is more active than their normal level you'd expect their AWB to be higher, but this effect my depend on their total physical activity? I have no doubt that the authors have thought deeply about this, and I'd just like a little additional explanation.

Thanks for the opportunity to clarify our rationale to include both a mean-centered PA as well as a person-mean PA variable. As the Reviewer correctly recognised, we divided the predictor variables in the one-stage IPD meta-analysis in a person-mean-centered predictor – that is, how much the momentary physical activity differs from the average physical activity of a person, and a person-mean predictor – that is, the person's average amount of physical activity before all e-diary prompts).

In our one-stage IPD meta-analysis, we included both variables in the analyses to test two separate hypotheses: Physical activity is associated with affective well-being (1) on a momentary within-person level and (2) on a between-person level. That is, the person-mean-centered part indicates whether being more or less physically active than usual is associated with affective well-being (within-person association). The person-mean, on the other hand, serves as an indicator of whether people that are on average more or less active than others also exhibit higher or lower affective well-being (between-person association).

This is an established procedure to differentiate between- and within-person effects in mixed effects models of EMA studies[4–6]. This distinction is crucial to avoid the pitfall of the ecological fallacy, a phenomenon describing that within-person and between-person processes differ methodologically, statistically, and empirically[7–11]. Hamaker[7] (p. 3) illustrates the ecological fallacy using the example of typing speed and typos:

> *"Suppose we are interested in the relationship between typing speed (i.e., number of words typed per minute), and the percentage of typos that are made. If we look at the cross-sectional relationship (i.e., the population level), we may well find a negative*

relationship, in that people who type faster make fewer mistakes (this may be reflective of the fact that people with better developed typing skills and more experience both type faster and make fewer mistakes). [...] If we were to generalise this result to the within-person level, we would conclude that if a particular person types faster, (s)he will make fewer mistakes. Clearly, this is not what we expect: In fact, we are fairly certain that for any particular individual the number of typos will increase if (s)he tries to type faster. This implies a positive—rather than a negative—relationship at the within-person level.“

Translated to affective well-being and physical activity: That people feel relatively more energetic after being more physically active than their normal level does not necessarily imply that people who are more active on average than others also feel more energetic than others. Indeed, in our analyses we predominantly found support for a within-person association but only little support for a between-person association between daily life physical activity and affective well-being, again emphasizing the crucial step to separate within-person effects (the effect of a time-varying predictor centered on the person mean) from between-person effects (the effect of the person average of a time-varying predictor).

For the most part, I am impressed by the analyses. They seem robust, and aligned with the registered plan.

We thank the Reviewer for the positive feedback on our analyses. We greatly appreciate the Reviewer's recognition of the robustness of results and of the alignment with preregistration.

Results

I may have missed it, but the PRISMA-IPD checklists suggests "Where applicable, also report similar study characteristics for any studies not providing IPD" (item 18). It would be interesting to know if there are any systematic differences between the studies you were able to get data from vs not.

We thank the Reviewer for bringing up this suggestion. Following the Reviewers suggestion, we now extracted relevant information from studies not providing data to compare them to studies that provided data. We set up a logistic regression model and predicted data provision by study characteristics that were extracted from publications. The analysis included the predictors age, gender, number of study participants, study location, prompts per day, days of sampling as well as antecedent and consequent models. In the studies included, physical activity was more often investigated prior to affective well-being than in studies that did not provide data (p = .01). None of the other variables predicted data provision. We now included this analysis in the revised version of the supplement, see S19.

Methods (p. 16): Finally, we set up a logistic regression model and predicted data provision by study characteristics to explore systematic differences between studies that provided data and studies that did not provide data for our meta-analysis (see S19). In the studies included, physical activity was more often investigated prior to affective well-being than in studies that did not provide data (p = .01).

I found Figure 6 quite confusing. I'm not completely sure it adds anything.

We thank the Reviewer for their important remark. We included Figure 6 to inform the general audience of our results by means of practical effect sizes and to simplify our results. The Reviewers' comments made us realise, however, that the figure needed to be clarified. That is why we have substantially revised Figure 6.

Further, given that the practical effect sizes are now also included in the graphical summary of findings (Figure 2; motivated by Reviewer 3, see below), we moved Figure 6 to the supplementary materials (see Figure S6).

I am struggling to see where the numbers from lines 342 onwards come from. The part I am having trouble reconciling is how we have gone from a correlation of 0.05 (Fig 2) or a standardised beta of 0.05 (Fig 3) for positive affect, to an effect size which is described as "considerable". Part of the reason I think I am finding it hard to parse this, is because the results given in the text are somehow on a scale of 0-100, which the numbers used in Table 1 are 1-4. If I am reading table S2.2a correctly, a full standard deviation increase in person-mean physical activity (which seems large) would be associated with a 0.09 unit increase on the 1-4 positive affect scale. That does not seem "considerable". The comparisons made also don't really suggest very large effects: it's consistent with other 'daily life activities'. This would not suggest that physical activity is a panacea. I'd appreciate either clearer explanation for why the authors feel that these are considerable effects sizes, or a tempering of the language.

We sincerely apologise for the unclear description of findings. Our analysis comprised three analysis steps: First, we conducted a (multivariate) meta-analysis based on multilevel within- and between-person correlations *r* (two-stage IPD). Second, we built a series of multilevel models including standardised PA values and distinct AWB dimensions as well the control variables age and sex/gender (one-stage IPD). Finally, we built another series of multilevel models with the same structure, but restricted the data to studies containing measures of raw PA data (Movement Acceleration Intensity, MAI, in milli-g) and added PA aggregation interval as a control variable (MAI one-stage IPD).

By means of this final analysis step, we present practical effect sizes (also referred to as benchmarking, e.g.,[12–17]). That is, we inserted MAI values for sitting versus walking into the regression formula. Originally, we rescaled the AWB outcomes to 0-100 (i.e., we divided the results by 4 and multiplied by 101). Our aim was to show how sitting versus walking is

associated with affective well-being on a scale from 0-100 to make it easier for the general audience to understand and compare the effects with other daily life activities (e.g., with relaxing, eating, or conversation[15]). However, based on the Reviewer's comment, we realised that this only introduced confusion, so we now present the practical effect sizes from analysis step 3 (mixed effects models of studies reporting MAI as a physical activity metric) on the scale of 1-4.

Regarding the second aspect, the depiction of the magnitude of effect sizes, we agree with the Reviewer: a 0.09 unit increase on a 1-4 scale for positive affect is, in absolute terms, not considerable. We would also like to emphasize that we do not wish to claim that physical activity is a panacea. We agree with the Reviewer that the effect sizes are consistent with other daily life activities. The only aspect of affective well-being for which we found larger effect sizes is energetic arousal. Here, we assume that effects of physical activity on energetic arousal are larger than many previously investigated effects in daily life (e.g., [15]), so that we assume that this association deserves special attention. For the other dimensions of AWB, we found consistent effects comparable to other activities of daily life. The Reviewer's comment made us realise that we, indeed, needed to temper the language throughout the manuscript. That is why we took extra care to find more adequate wordings in our revised manuscript and made the following changes:

Abstract (p. 3): The practical effect sizes are comparable to other daily life activities, with energetic arousal evincing the strongest relation to PA.

Results (p. 8): When translating these effect sizes into practice to estimate their relevance in humans' everyday life, their magnitude is comparable to other daily life activities. Changing one's activity in the range of, for example, from sitting to walking is associated with an increase in AWB […] and exercising to an increase of approximately 0.36 points when transferring effects to a scale from 1 to 4 (see S6).

Discussion (p. 10): The practical effect sizes are comparable to other daily life activities, with energetic arousal evincing the strongest within-person association with PA.

Discussion (p. 10): Overall, the data show that PA is robustly associated with humans' AWB as humans go about their daily routines.

Discussion (p. 11): Accordingly, PA–energetic arousal ~~and PA–positive affective states~~ relations present themselves as particularly salient targets to shape theories of health behaviour change and develop future precision medicine approaches.

Discussion (p. 11): Overall, the practical effect sizes of PA–AWB relations derived from our IPD meta-analysis are comparable to other daily life activities (e.g., reading, listening to music eating) that were investigated in a large-scale study by Killingsworth and Gilbert[63]. For feelings of energy, they appear considerable.

Discussion (p. 11): First, given its considerable effect size, interactions of PA with energetic arousal ~~and positive affect~~ appear as important future research targets.

We would like to express our sincere gratitude to the Reviewer for the time and effort dedicated to reviewing and enhancing our manuscript. The Reviewer's suggestions allowed us to clarify our analyses and findings and to apply a standard Risk of Bias tool to EMA studies, which makes the Risk of Bias assessments accessible to a wider audience.

Reviewer #2 (Remarks to the Author):

This is an interesting submission that entailed an individual participant data (IPD) meta-analysis into physical activity and affective responses. There is a lot to like about the paper, such as the pre-registered PRISMA-informed methodology, the use of device-based accelerometry data and the associated one- and two-stage IPD analyses. While the topic of the paper is likely to be of interest to Nature Human Behavior's readership, I have the following concerns about the submission. A limitation of affective psychology research is that the terms core affect, emotion, and mood are often used interchangeably without conceptual differentiation (Russell & Feldman Barrett, 1999). Unfortunately, it appears that you often fall into this trap by conflating key affective phenomena terminology. The rationale for including "five dimensions of affective well-being that were most frequently assessed in real-life studies on AWB-PA associations" is somewhat problematic – just because a set of constructs are frequently measured, it does necessarily mean that they coherently tap the underlying affective phenomena of interest. It is currently unclear, for example, how positive/negative affect differs from valence, which is typically conceptualized as a bipolar dimension ranging from pleasure to displeasure (Ekkekakis & Zenko, 2016). Likewise, you mentioned that fatigue was "assigned to negative affect" and that "mood [was assigned] to valence". Importantly, these are very different affective constructs, mood being more diffuse/low intensity and valence being part of core affect, constantly accessible to consciousness (Russell, 2009). In addition, mood is suggested to last for days, weeks, or months (Ekkekakis & Zenko, 2016), so it would have been useful if you provided a rationale for why you think mood is amenable to change in relation to a single session of physical activity.

The studies included in the analysis comprise a smorgasbord of affective phenomena without any clear guiding conceptual framework and this is further evidenced by the associated search terms in S12 ("mood* OR emotion* OR affect*"). A related problem, is a lack of consideration toward the measures used in the included studies. S1 reveals that many studies administered the Positive and Negative Affect Schedule (PANAS; Watson et al., 1988), which has been heavily criticized for only tapping high-activation states (Barrett & Russell, 1999). Similarly, other studies appear to have employed a measure of mood proposed by Wilhelm and Schoebi (2007), who note that "moods are not necessarily linked to an obvious cause that can be related to an event and its specific appraisal" (p. 259). Again, a rationale is needed as to why the construct mood is of interest in relation to physical activity.

Casting the net far and wide is good for retaining studies and associated data (indeed, I commend you for amalgamating almost 1 million hours of accelerometry), but these data are considerably messy in relation to the affective phenomena that they tap and your associated categories that you have enforced on them. Unfortunately, this perpetuates conceptual ambiguity. An alternative strategy, would have been to employ the three-step approach advocated by Ekkekakis and Zenko (2016). First, you initially decide upon an appropriate affective construct (core affect, emotion, or mood). Second, you choose and clearly explain the most appropriate theoretical framework for the chosen construct (distinct states vs. dimensional approaches; Ekkekakis, 2013). It looks as though you have a mixture of the two approaches at present (positive affect, negative affect, and valence). Finally, you select the psychometrically strongest measure based on the chosen theoretical framework. Basing your inclusion criteria on psychometrically strong measures would have resulted in far fewer studies, but would have put you on much firmer ground when drawing conclusions from the data.

We thank the Reviewer for acknowledging our PRISMA-informed methodology, the use of device-based accelerometry, and the application of one- and two-stage IPD analyses. We are delighted that the Reviewer deems our manuscript to be of interest to *Nature Human Behaviour*'s readership.

We fully agree with the Reviewer that a clear conceptual framework is indispensable, both in original research and in meta-analyses, and that there is inconsistent and muddled use of affective terminology in the field more generally. The conceptual framework of our meta-analysis is guided by daily life research on the short-term association of physical activity and affective well-being. Research in this area is relatively novel[18]. It differs from laboratory studies not only with regard to the experimental control but also the type of physical activity being researched. In short, physical activities in everyday life (e.g., gardening or climbing stairs) are generally characterised by relatively low energy expenditure and differ from volitional and structured activities induced in a laboratory and/or clinical setting (for detailed discussions see, e.g.,[18,19]). For example, although individuals will likely consciously decide to engage in exercise and perceive it as a temporary event, the majority of physical activity in daily life occurs incidentally, most likely without any conscious processing of the physical activity involved. The conceptual framework including behavioural, psychological, and (neuro)biological mechanisms how incidental physical activity relates to affective well-being may therefore plausibly differ from those linking exercise activities to affective well-being.

Accordingly, Researchers of daily life studies put forth different hypotheses about the relationship of physical activity and diverse affective phenomena within persons on a micro-temporal time scale; that is, some assumed physical activity to relate to core affect (e.g., [20–27]), others investigated the association of physical activity with mood (e.g.,[28]), yet others were interested in the relationship of physical activity with concrete emotions (e.g.,[29]).

In the present meta-analysis, we aim to summarise all available evidence on associations of physical activity and affective well-being in everyday life. To detect all available evidence despite the heterogeneous affective terminology across individual daily life studies, we indeed chose broad search terms including "affect", "emotion", and "mood".

Importantly, following the assumed hierarchical structure of the affective domain referred to by the Reviewer (e.g.,[30]), all affective phenomena share commonalities, for example, in comprising positive or negative valence. Thus, they can be summarised under the umbrella term "affective well-being" (for prominent work illustrating this approach to summarise affective phenomena see e.g.,[31–34]). We agree that the summary of distinct affective phenomena under the umbrella term AWB had not been stated clearly enough in the original version of the manuscript and have revised accordingly.

Of course, we fully agree with the Reviewer on the lack of conceptual differentiation in research on affective well-being and the key challenge this poses to the field of affective psychology. A recent comment in *Nature Human Behaviour* authored by numerous leading researchers in affective sciences stated[32]:

> *"The definition of affective processes, either as a whole or individually, is subject to debate. For example, questions continue concerning how definitions of emotion should accommodate the fact that we continuously evaluate events around us and how our central and peripheral nervous systems allow the emergence of expressions, physiological arousal and bodily reactions, action tendencies, and felt subjective experiences. Nonetheless, it seems that affective processes are typically understood to relate to the notion of (dis)pleasure or valence, to not necessarily be consciously felt, and to mobilize the organism to deal with events that may be important to that organism."*

In short: Despite various efforts to standardise affective terminology (e.g.,[30,35,36]), terms such as core affect, feelings, emotions, and moods remain inconsistently used[30,36]. Core affect as a construct introduced by Russell and Barrett[35] comprises "the most elementary consciously accessible affective feelings". It is a continuous and dimensional phenomenon, which is most apparent in moods and emotions[37]. Russell[37] argues that Thayer's[38] activation, Watson's and Tellegen's[39] affect, Morris'[40] mood, and the commonly used term "feeling" all tap into core affect but use different terminology. Core affect has been modeled as a two-dimensional (e.g.,[38,39,41]) and three-dimensional construct (e.g.,[42–44]). For both there is empirical evidence[44,45]. In contrast, emotions involve stimulus-related changes in multiple components (e.g., facial expressions, action tendencies, physiology, core affect) and cognitive processes such as appraisal or constructionism[30,36,46]. Various emotion theories persist, including Ekman's[47] basic emotion theory, Lange's[48] network theory, Scherer's[49] appraisal theory, Moors's[50] goal-directed theory, Barrett and Russell's[51] psychological construction theories, and Mesquita's and Parkinson's[52] social constructionist theories (see Scarantino[53] for an overview). Definitions of mood appear to exhibit the largest heterogeneity. Although some

conceptualizations appear similar to core affect (e.g., [20–22,27,40,45]), others differentiate mood from core affect, for example, in terms of permanence, duration, and cognitive appraisal[30,37].

Accordingly, it is not surprising that the terminology for distinct AWB constructs differs between daily life studies on PA–AWB. For example, although mood and valence are very different constructs following the definition highlighted by the Reviewer (e.g.,[54,55]), Wilhelm and Schoebi's[45] definition of mood shows a conceptual overlap with core affect[20–23,27]. Both describe a continuous, dimensional construct rather unrelated to specific events, objects, and appraisal processes[45,54,55]. Particularly, many daily life studies that declare to assess mood are not measuring something that is largely stable over days and weeks. Instead, their assessments of mood exhibit substantial within-person variability over short time scales. As only a few[20–23,26,27,56,57] out of 69 daily life studies referred to Russell's[37] definitions of affective phenomena, there is room for interpretation on whether researchers intended to investigate the associations of physical activity with core affect, mood, or emotions.

Indeed, this conceptual debate was insufficiently discussed in the originally submitted manuscript. We think that the meta-analysis is ideally suited to draw attention to this challenge and accordingly included paragraphs throughout the revised manuscript.

We want to particularly emphasise our efforts to directly avoid the simple conflation of key affective phenomena. To this end, we split the affective phenomena in our meta-analysis into the distinct constructs of valence, calmness, energetic arousal, positive and negative affect. We thus intentionally summarised the key affective phenomena researched in daily life studies on PA–AWB. Thereby, we hope to advance the science in this domain (to the degree possible given the constraints of the extant data sources) by not only differentiating between phenomena but also following logic analogous to that suggested by the Reviewer.

In particular, as stated by the Reviewer, there is a difference between unipolar and bipolar affective conceptualizations. Valence is typically conceptualised as a bipolar dimension ranging from pleasure to displeasure. Positive and negative affective states are unipolar. Hence, we summarised bipolar affective constructs in valence, calmness, and energetic arousal. Unipolar affective constructs were summarised in positive/negative affect; although it is important to note that this conceptualization does not necessarily correspond to the PANAS-PA and PANAS-NA but may combine high and low activation items, i.e., items are assigned to positive or negative affect according to their unipolar valence.

For example, for datasets that did not refer to valence, energetic arousal, calmness, positive affect, or negative affect, the „mood" operationalizations that we assigned to valence were: „Overall, my mood is: Very unpleasant (-10) to Very pleasant (10)", „Please rate your current mood on a scale of 1 to 10, where 1 = sad and 10 = happy", and „How did

you feel during the last hour? Very negative (-2) to Very positive (2)“. Following Ekkekakis[30] and Russell[37] , these items appear to capture valence as a dimension of core affect. We have clarified and amended the original description of our procedure in the revised manuscript and thank the Reviewer for highlighting this point.

Given the lack of consensus in affective psychology, we agree with the Reviewer that the decisions necessary to conduct a meta-analysis could have been made differently on this very detailed level. This motivates sensitivity analyses.

Following the Editors’ recommendations, we did not remove our pre-registered analyses but amended our analyses by a set of sensitivity tests. In particular, we ran two sensitivity analyses. The sensitivity analyses focus on a three-dimensional and two-dimensional operationalization of core affect. The first sensitivity analysis only includes studies with uniformly assessed valence, calmness, and energetic arousal. This approach builds upon a clearly defined, psychometrically validated, and highly established framework[45,58]. The second sensitivity analysis focuses on positive and negative affect as operationalised in the PANAS. Thus, the second sensitivity analysis only includes studies which summarised high-activation states according to their valence in positive and negative affect. Please note that, although we agree with the Reviewer that this operationalization may only questionably cover the full spectrum of positive and negative affect[30,35,59], it is the best solution for a second sensitivity analysis given the data available to establish uniformity among the various operationalizations in studies focusing on unipolar affective constructs. Sensitivity analyses confirm our main results, even though they substantially reduce the number of studies meta-analysed as correctly anticipated by the Reviewer. This strongly supports our approach to summarise affective phenomena in the meta-analysis.

Fortunately, the extensive scientific discourse on the operationalization of AWB and the persisting disagreement does not hinder our meta-analysis from making scientific progress, which is fully in line with the call of numerous leading researchers in affective sciences as published in *Nature Human Behaviour*[32]:

> *“In any case, scientific study [of affective phenomena] is beset by questions of terminology: persistent difficulties in formally defining ‘cognition’ did not prevent the transition from behaviourism to cognitivism, and the fact that there is no consensus concerning a formal definition of other important constructs, such as intelligence, religion, culture, and even life, does not preclude fruitful scientific study of them.”*

We want to thank the Reviewer again for their valuable input. The comments and suggestions helped us to focus our discussion regarding the heterogeneity of affective well-being definitions in affective science, to refine explanations of the purpose of our analyses, to add a set of sensitivity analysis that shows the robustness of our main results, and to point out future directions in the field.

We have taken the Reviewer's feedback into account by comprehensively revising and significantly amending our manuscript as suggested by the Reviewer and the Editors, adding clarity to our manuscript and analyses.

In line with this line of reasoning, we have comprehensively revised our manuscript as detailed in the following:

1. Throughout the manuscript, we adapted our wording to resolve ambiguity (e.g., "AWB concepts" instead of "AWB dimensions" to also include affective states, "positive/negative affective states" instead of "positive/negative affect").

2. We included definitions of the affective terminology used (e.g., "affective well-being" as an umbrella term that comprises diverse affective well-being concepts).

   Introduction (p. 5): To extensively summarise all evidence, we chose to focus on AWB as a superordinate term of affective constructs[12,29,43]. Further, […]

3. We have explicitly pointed out the existing debate regarding terminology in the field of affective sciences.

   Introduction (p. 5): In sum, although intensive longitudinal data on AWB–PA associations are rapidly increasing, evidence remains ambiguous. The concepts and terminology of AWB strongly differ between studies (e.g., mood, emotions, affect, core affect) and there is little consistency regarding the items employed.

   Discussion (p. 11): Of note, our meta-analysis revealed a pronounced variability between studies in the instruments applied for the assessment of AWB, as well as a limited coverage of certain AWB dimensions. This appears as a challenge to daily life studies.

   Discussion (p. 12): Third, studies varied in the AWB concepts investigated and, within concepts, the specific items employed. We streamlined our analyses across unipolar (positive affective states, negative affective states) and bipolar (valence, calmness, energetic arousal) AWB concepts, resulting in the most comprehensive existing summary of evidence on PA–AWB associations across AWB concepts. Despite the heterogeneity of AWB assessments, our meta-analysis reveals that PA can explain meaningful variance across AWB concepts.

   Methods (p. 14-15): In the antecedent models, AWB served as the primary outcome. There is an ongoing debate on how to conceptualise, define, and operationalise AWB[29,58,79,141–147]. A variety of terms such as core affect, feelings, emotions, and moods, exists, which are, to date, not consistently used (for an extensive discussion, see[29] and S9). Prominent models of AWB include Russell's[148] circumplex model, Watson et al.'s two-dimensional approach operationalised within the PANAS[51] and Thayer's concept of activation comprising tension and wakefulness[141,149,150]. Recently, three-dimensional theories of AWB, which include the dimensions of valence, calmness, and energetic arousal[48,151], have gained increasing interest. To extensively summarise the heterogeneous literature on PA and AWB, we focused on five constructs from two- and three-dimensional models of AWB that were frequently assessed in real-life studies on AWB–PA associations[16]: positive affective states, negative affective states, valence, energetic arousal, and calmness.

We emphasise the lack of consistent affective terminology as an important research frontier in the discussion:

Discussion (p. 11): We conducted sensitivity analyses revealing that our main analyses are robust against assignments of AWB constructs to positive affective states, negative affective states, valence, energetic arousal, and calmness (see S4.5, S4.6, S9). We recommend future studies to provide clear conceptual rationales and rely on psychometrically evaluated measures when conducting a study on PA-AWB associations in daily life, for example guided by a framework from affective psychology[52]. The development of formalised and testable psychological theories[92-94] as well as item pools (e.g., the ESM Item Repository[95]) for daily life research can further contribute to greater consensus.

4. We summarised our sensitivity analysis to show robustness against the assignment of AWB constructs in the Results and Methods sections:

Results (p. 8): Drawing on a conceptual framework from affective psychology[52], we conducted sensitivity analyses for AWB operationalizations. For bipolar conceptualizations of AWB, we jointly analysed studies (k_{ant} = 17, k_{con} = 8) assessing valence, energetic arousal, and calmness with the psychometrically evaluated short-version of the MDMQ[48]. The short-version of the MDMQ has been referenced as a measure of core affect[53-57]. For unipolar conceptualizations of AWB, there is no similarly established and psychometrically evaluated measure in daily life research and the collected data. We concentrated our analysis on all studies that followed the PANAS[51] and summarised high-activation items in positive and negative affect (k_{ant} = 10, k_{con} = 4). It needs to be kept in mind that this operationalization is not undisputed (see, e.g.,[58,59] for a critical discussion of the PANAS). The sensitivity analyses support our main findings. For a more detailed description of the theoretical considerations and the resulting analyses, see S4.5, S4.6, and S9.

Methods (p. 16): Additionally, to ensure robustness of AWB operationalizations, we conducted sensitivity analyses in the one-stage IPD analysis. These analyses followed the same procedure as our main analysis but were restricted to a subset of studies that followed a narrowly defined assessment of AWB (see S4.5, S4.6, S9).

We extensively described our theoretical considerations and analyses in the supplementary material S4.5, S4.6, and S9 (see below).

5. We changed our wording regarding the search term to emphasise that the terms mood, emotion, and affect are not synonyms but potentially capture different affective phenomena.

Methods (p. 14): We searched the databases Web of Science, PubMed, Scopus, SPORTDiscus, and PsycINFO until December 2023 using the search terms "ecological momentary assessment", "physical activity", and "sedentary behavior" as well as their synonyms (see S12 for the adapted Boolean operators) and search terms for the umbrella term AWB; these comprise "mood" or "emotion" or "affect".

We amended the Boolean operators in S12 by the information that the three affective terms (i.e., mood, emotion, affect) were included jointly to achieve the

best coverage of research on affective well-being and physical activity in daily life and that they are not interchangeable but, depending on the definition of affective terms, may refer to different affective constructs.

Supplement: *Note.* We investigated the associations of PA and AWB in daily life. We used AWB as an established umbrella term for different affective phenomena such as mood, emotions, and affect, which cannot be used interchangeably. We chose a broad search term to achieve the best coverage of evidence.

6. We detailed the measures individual studies employed (supplement Table S1).

   We detailed our procedure to differentiate between the affective constructs of valence, calmness, energetic arousal, positive affective states, and negative affective states.

   Results (p. 6): We next investigated associations of PA with distinct AWB concepts. Here, we differentiated between two unipolar AWB concepts (positive affective states, negative affective states) and three bipolar AWB concepts (valence, energetic arousal, calmness). We found…

   Methods (p. 15): We differentiated positive and negative affective states (as unipolar AWB concepts) from bipolar AWB concepts (i.e., valence, energetic arousal, and calmness).

   Further, to transparently convey how we assigned constructs to valence, calmness, energetic arousal, positive affective states, and negative affective states, we now include the operationalizations and a detailed description of our procedure in the methods section:

   Methods (p. 15): If studies included none of the predefined AWB dimensions, the assessed AWB measures were assigned to positive affective states, negative affective states, valence, energetic arousal, and calmness by five researchers independently (MR, MG, GB, IT, JR); a unipolar item of "fatigue" was, e.g., assigned to negative affective states, a unipolar item of "pleasure" (i.e., "not at all pleasurable" to "totally pleasurable") to positive affective states. "Mood" was depending on whether it was assessed unipolarly (e.g., DABS Mood[152]) or bipolarly (i.e., "very unpleasant" to "very pleasant"; "sad" to "happy"; "very negative" to "very positive") assigned to positive affective states and negative affective states (unipolar) or to valence (bipolar).

We elaborated on these paragraphs in-depth in the supplementary material S9, which comprehensively focuses on measuring affective well-being in daily life studies on physical activity:

Supplementary materials S9: There is an ongoing debate on how to conceptualise, define, and operationalise affective phenomena[129–137]. Despite various efforts to standardise affective terminology (e.g.,[131,133,138]), terms such as core affect, feelings, emotions, and moods remain inconsistently used[131,133]. We use the term affective well-being (AWB) as a superordinate term that comprises all types of affective phenomena[139,140].

A prominent approach to standardise affective terminology differentiates between core affect, emotions, and mood (e.g., [133,138,141]). Russell and Barrett[138] define core affect as "the most elementary consciously accessible affective feelings". Russell[141] argues that concepts such as Thayer's[142] *activation*, Watson's and Tellegen's[143] *affect*, Morris'[144] *mood*, and the commonly used term *feeling* also tap into core affect, although they employ different terminology. Core affect is a continuous and dimensional phenomenon, which is most apparent in moods and emotions[141]. It has been modelled both as a two-dimensional (e.g.,[142,143,145]) and a three-dimensional concept (e.g.,[146–148]). For both there is empirical evidence[6,148]. Emotions, on the other hand, involve stimulus-related changes in multiple components (e.g., facial expressions, action tendencies, physiology, core affect) and cognitive processes, such as appraisal or constructionism[131,133,135]. Various emotion theories persist such as Ekman's[149] basic emotion theory, Lange's[150] network theory, Scherer's[151] appraisal theory, Moors's[152] goal-directed theory, Barrett and Russell's[153] psychological construction theories, and Mesquita and Parkinson's[154] social constructionist theories (see Scarantino[137] for an overview). Finally, definitions of mood appear to exhibit the largest heterogeneity. Russell[141] describes mood as "prolonged core affect" and a free-floating "fuzzy [concept] because neither duration nor degree of stability is defined". Ekkekakis[133] summarises that mood further involves appraisal processes, evolving around more existential topics compared to emotions. Other conceptualization of mood, however, appear similar to core affect (e.g.,[6–9,30,97,144]). Further, it needs to be noted that mood and core affect are sometimes not easily separated as changes in core affect may condense in changes in mood ratings[133].

Given the ongoing debate on basic affective terminology, several procedures for assessing AWB in daily life are currently in use: Some researchers employ self-developed items tailored to their research questions but without psychometric evaluations. Others select items from established questionnaires from cross-sectional studies, such as the Positive and Negative Affect Schedule (PANAS[85,3]; for a critical discussion of the PANAS see, e.g.,[133,155]). Both approaches commonly result in a list of affective states (e.g., content, happy, calm, excited, sad, angry, upset, anxious), which is summarised in scores for positive and negative affect. In many studies the composition of the item list varies and is not theoretically justified[156,157]. The third approach are questionnaires based on a solid theoretical framework that have demonstrated good psychometric properties in daily life[6,133,157]. To date, there is only a limited number of affective measures meeting these criteria[157], with the short-version of the MDMQ[6] being a prominent example.

To test for the robustness of our findings in the current meta-analysis against different AWB operationalizations, we draw on a theoretical framework from affective psychology[158].

Different affective phenomena, such as core affect, mood, or emotions, may be of relevance for PA in daily life. All studies identified in our literature search assessed PA continuously. Hence, all kinds of PA in everyday life were assessed. For our sensitivity analysis, we assume that the majority of physical activities in daily life (e.g., walking to the bus station) are not perceived as events but occurs incidentally without any conscious reflection on the PA involved[50,159]. Hence, we do not presume that PA in daily life elicits emotions that, per definition, are reactions to specific events or objects and comprise psychological construction[160] or appraisal[131] processes. Whether PA in daily life may be linked to core affect or mood is more difficult to theorise. As outlined above, the predominant differentiation between mood and core affect appears to lie in the time course (with mood being transient and lasting for hours, days, or weeks and core affect being a continuous phenomenon) and the presumed underlying appraisal processes of mood.

Following Ekkekakis'[133] line of argument that "brief interventions (i.e., those lasting for just a few minutes) may not be long or powerful enough to change mood per se. What probably changes in such cases is core affect and it is the core affect that is reflected in self-reports of mood." (p. 51), we chose to focus our sensitivity analysis on short-term associations of PA and core affect.

We assume PA as an integral part of daily life to be associated with the global domain of core affect rather than specific affective states. Therefore, we adopt a dimensional approach to core affect instead of relying on distinct states. Both two-dimensional and three-dimensional models of core affect exist. The most prominent theoretical model of core affect are different versions of a circumplex model[142,143,145,155,161]. Russell[145] explains core affect by the two dimensions of valence and activation. Specific affective states can be arranged along those two dimensions. Watson and Tellegen[143] identified two dimensions that combine valence and activation: positive affect/activation, which ranges from low activated negative affect to high activated positive affect, and negative affect/activation, which ranges from low activated positive affect to high activated negative affect.

Three-dimensional models of core affect[146–148], on the other hand, use a different terminology than the circumplex model. Instead of referring to core affect, three-dimensional models refer to mood, which is defined as "a current inner experience and inner perception of an individual [that] is not bound to specific objects (persons, things, events, institutions) or situations and is not tied to specific, experientially evident causes"[162] and, therefore, appears to largely overlap with Russell's core affect. Three-dimensional models of core affect differentiate between the dimensions of valence or hedonic tone, tense arousal or calmness (its opposite), and energetic arousal[146,147]. Thus, they differentiate two dimensions of arousal. With regard to PA, studies adapting a three-dimensional approach, have suggested differential effects of PA on valence, calmness, and energetic arousal[5,7,37,50,80].

Direct psychometric comparisons of two-dimensional and three-dimensional models appear to favour three-dimensional models of core affect[148], at least within-persons[6]. Since we are predominantly interested in within-person associations, we therefore adopt a three-dimensional model of core affect for our sensitivity analysis.

Three-dimensional measures of core affect[146–148] exhibited good psychometric properties in cross-sectional studies. However, the psychometric quality cannot be directly transferred from cross-sectional to daily life studies[6]. Thus, Wilhelm and Schoebi[6] developed a short-version (6 items) of the Multidimensional Mood Questionnaire (MDMQ[147]) explicitly for the application in daily life studies. The short version of the MDMQ demonstrated acceptable within- and between-person reliability and sensibility to change in daily life[6]. Hence, to demonstrate robustness of findings for a narrowly defined AWB operationalization, we will jointly analyse only those studies using the short-version of the MDMQ in the following sensitivity analysis.

As a second sensitivity analysis, we adopted the PANAS conceptualization of positive and negative affect. Thus, we jointly analysed all studies that used items from the PANAS to derive scores for positive and negative activation, i.e., that used high-activation items only. A sensitivity analysis based on the circumplex model would also be desirable. However, there are only few daily life studies that directly assess the dimensions of the circumplex model to date (e.g.,[46]), making it impossible to conduct a sensitivity analysis on the dimensions of valence and activation.

Reviewer #3 (Remarks to the Author):

This study presents a robust and comprehensive Individual Participant Data (IPD) meta-analysis on the bidirectional relationship between physical activity (PA) and affective well-being (AWB) in people's daily lives. The work is notable for its scope synthesizing 67 unique datasets from 14 countries, covering 8,223 participants, over 321,000 mood ratings, and nearly 1 million hours of accelerometer-measured PA. The paper addresses a key gap in health psychology and behavioral science: understanding the dynamic, within-person effects of physical activity on momentary well-being in real-world contexts. Below are a series of comments intended to help the authors strengthen their manuscript:

We thank the Reviewer for their positive assessment of our work. We are especially delighted that the Reviewer noted the scope of our work and confirmed that it addresses a key research gap in health psychology and behavioural science. We thank the Reviewer for their thoughtful suggestions for improving our manuscript.

1. While heterogeneity is flagged and some moderators are tested (age, gender/sex, BMI, weekday/weekend), the explanation of why certain effects vary remains largely descriptive. Where data permit, the authors could expand discussion of why certain subgroups may respond differently — to guide precision intervention development. The authors could also strengthen interpretation of heterogeneity by linking findings more explicitly to theoretical models (e.g., motivational psychology, social determinants of health).

We thank the Reviewer for this important feedback. In response to the Reviewer's comment, we have expanded our discussion on the potential sources of heterogeneity and discussed interpretations in light of theoretical models. We appreciate the Reviewer raising this point. In the following, we detail the explanations and interpretations and describe how we condensed them into the revised version of the manuscript.

In our meta-analysis, we explored potential moderations of PA-AWB associations by prominent sociodemographic and measurement variables. Although we received no overarching evidence for uniform moderation patterns across AWB concepts, distinct interactions emerged.

First, we found that the association of physical activity with positive affect and valence was partially moderated by age and BMI. That is, high-BMI and older individuals exhibited weaker associations between physical activity and positive affect and valence.

Against the background of theories from motivational psychology, particularly hedonic theories[60], the epidemiological observation of reduced PA in older adults and high-BMI populations[61–64] plausibly fits the blunted relation of physical activity to positive affect and valence observed in our meta-analysis. Hedonic theories postulate that individuals strive

to maximise their well-being by engaging in activities that bring them pleasure[60]. For physical activity behaviour, there is evidence that affect during exercise predicts future physical activity engagement[65,66]. Thus, it is tempting to speculate that high-BMI and older adults face affective barriers hindering PA engagement: PA does not sufficiently enhance pleasure to reinforce future PA engagement, and the threshold for AWB to trigger PA is high.

As the altered PA–AWB association plausibly links to the reduced PA engagement in vulnerable groups, research needs to identify readily accessible ways to enhance PA-AWB associations in these groups for precision interventions. Ultimately, PA-AWB associations may serve as an intervention target to improve physical and mental health. Here, sociodemographic, biological, and contextual factors are putative catalysts. Building upon the recent discourse on the intertwinement of behaviour change and policymaking, interventions are particularly promising in facilitating environments without structural constraints, as outlined most recently by Hofmann et al.[67] in *Nature Human Behaviour*. These may comprise important social determinants of health[68–72]. For example, two recent prominent large-scale studies published in *Nature* support the causal relationship between built environments and health-enhancing PA[73,74]. They suggest that favourable environments such as cities with increased walkability can reduce PA inequality and obesity[73,74].

For AWB-PA associations, there is indeed also evidence that environmental factors such as green spaces are able to evoke additional AWB benefit[17,75–79]. In practice, there is a wealth of promising modifiable candidates to catalyse effects of PA on AWB[13,23].

The moderation pattern of blunted PA-AWB responses in high-BMI populations is also in line with findings from laboratory studies evidencing a poorer affective response to exercise in overweight and obese individuals (e.g.,[80–82]). Further, taking a neurobiological perspective, Jackson et al.[83] observed reduced activation of reward centres and heightened activation in regions associated with negative affect regulation when presenting overweight women with images of exercise in a fMRI study. For older individuals, Ingram[84] suggests reduced dopamine release or loss of dopamine receptors as a mechanism of age-related activity decline. Here, it is tempting to speculate that neurobiological processes may be influenced by intensity levels of physical activity, a candidate for future precision interventions that has already been tackled by some studies that yielded mixed evidence[81,85,86] a proposal that needs to be substantiated in future trials.

Second, we found that, for men, calmness is more strongly associated with subsequent physical activity than for women, yet women feel more energetically aroused after being physically active than men. Although the assessment of gender/sex differed between the studies included in our analysis and only few studies included a non-binary gender option, this differential pattern of physical activity – affective well-being associations may

plausibly link to distinct autonomic nervous system properties seen in people with female vs. male biology, such as differences in hormones[87] or heart rate variability (HRV;[88,89]), which has been linked to emotion regulation and stress[90,91]. It suggests that biologically-informed prevention and treatment approaches may be particularly salient for physical activity engagement and affective well-being.

Third, on weekends, the temporally bidirectional association of physical activity and energetic arousal is stronger than on weekdays, which partially matches findings on differences in work-related versus leisure-time PA impacts on both mental[92,93] and physical[94–96] health. Particularly, studies have found that while leisure time physical activity is consistently linked with reduced cardiovascular risk and depression, occupational physical activity showed negative health effects[92–96]. Hence, accounting for the day of week context appears of particular importance for future precision medicine approaches.

Indeed, as pointed out by the Reviewer, explanations of the findings from the moderator analyses were rather descriptive in the originally submitted manuscript. We now revised the discussion section as follows:

Discussion (pp. 11-12): Younger and low-BMI-individuals exhibit stronger associations of PA with subsequent valence or positive affective states. In light of motivational and particularly hedonic theories, which postulate that individuals strive to maximise their well-being, the less positive affective response to PA in high-BMI and older individuals plausibly fits epidemiological observations of reduced PA in these groups[3,99-101]. High-BMI and older adults may have weaker associations between PA and subsequent positive affective states and valence because PA may trigger more feelings of discomfort, pain, heat, stain, or other unpleasant physiological states in these groups. To enhance PA engagement, it thus appears especially important to identify putative catalysts of the PA-AWB associations in vulnerable groups[102]: Sociodemographic, biological, and contextual factors may facilitate effects of PA on pleasure to reinforce future PA engagement and lower the threshold for AWB to evoke PA. This may comprise aspects such as walkability[103,104], green spaces[105-107], or the social context[108-110]. Given neurobiological[111] and laboratory[112-114] findings of blunted PA-AWB responses in high-BMI individuals, it is also tempting to speculate that the intensity of PA differentially affects neurobiological processes and, thus, constitutes a candidate for precision intervention[114,115]. Next, for men, calmness is more strongly associated with subsequent PA than for women, yet women feel more energetically aroused after being physically active than men. Although the assessment of gender/sex differed between studies and only few studies included a non-binary gender option, this differential pattern of PA-AWB associations may relate to distinct autonomic nervous system properties seen in people with female vs. male biology, such as differences in hormones[116] or heart rate variability (HRV;[117,118]). If substantiated in future proposals, this suggests that biologically-informed prevention and treatment approaches may be particularly salient for PA engagement and AWB. Finally, on weekends, the bidirectional association of PA and energetic arousal is stronger than on weekdays, which partially matches findings on differences in work-related versus leisure-time PA impacts on both mental[119,120] and physical[121] health. Particularly, studies have found that while leisure time physical activity is consistently linked with reduced cardiovascular risk and depression, occupational physical activity appears to have negative health effects. Hence, accounting for the day of week context appears of particular importance for future precision medicine approaches.

2. In the discussion section, the authors should more clearly discuss the nuances of important limitations that readers should consider when drawing their own independent interpretations of the results beyond listing them in the limitations section. Primarily around the observational nature, residual confounding potential, between-study variability in instruments/definitions, and limited coverage of certain AWB dimensions in some datasets.

We fully agree with the Reviewer and appreciate the Reviewer's very helpful advice. We followed the Reviewer's suggestion and now discuss the implications of important limitations, such as the observational nature of the data, the potential of residual confounding, variability in instruments and definitions, as well as the limited coverage of AWB dimensions throughout the discussion. Particularly, we made the following amendments:

Discussion (p. 18): However, these implications need to be considered against the background of the observational nature of the data meta-analysed and the resulting potential for residual confounding. While this meta-analysis on intensive longitudinal data of daily life studies on PA and AWB comes with highest ecological validity, the designs of PA-AWB studies included are observational. That is, they do not include experimental manipulation to control the impact of confounding variables (i.e., variables impacting the PA-AWB association). As a result, unmeasured (time-varying) confounding variables (e.g., weather conditions) could have affected the findings. Ideally, future EMA studies consider the impact of these variables more thoroughly. For studies focusing on the PA-AWB association, a quasi-experimental design could be adopted[84,85]. For studies examining the AWB-PA association, potential confounding variables should be systematically identified and accounted for in the EMA design and the analyses[86,87].

Discussion (p. 11): Of note, our meta-analysis revealed a pronounced variability between studies in the instruments applied for the assessment of AWB, as well as a limited coverage of certain AWB dimensions. This appears as a conceptual challenge to daily life studies. We conducted sensitivity analyses revealing that our main analyses are robust against assignments of AWB constructs to positive affective states, negative affective states, valence, energetic arousal, and calmness (see S4.5, S4.6, S9). We recommend future studies to provide a conceptual rational and rely on psychometrically evaluated measures when conducting a study on PA-AWB associations in daily life, for example guided by a framework from affective psychology[52]. The development of formalised and testable psychological theories[92-94] as well as item pools (e.g., the ESM Item Repository[95]) for daily life research can further contribute to greater consensus.

3. The methods section is very detailed, with some elements that could be streamlined or moved to the Supplementary Materials. In its current form, the sheer length risks losing non-specialist readers and obscuring the central methodological insights that broad Nature Human Behaviour readers might seek. The authors may want to consider keeping in the main text only the conceptual and rationale-level description of methods, highlighting why each approach was chosen and how it advances the field. This will improve readability for a multi-disciplinary audience.

We thank the Reviewer for the valuable feedback and suggestions regarding our Methods section. Following the Reviewer's comment, we have extensively revised and simplified the Methods section and moved non-central paragraphs to the Supplementary Materials (S1, S2, S3, S11, S12).

4. The manuscript occasionally uses language implying causal inference (e.g., "PA is bi-directionally related," "PA leads to increases"), even though the observational nature of the IPD data precludes definitive causal claims. The authors could replace or qualify causal-sounding terms with wording that reflects association rather than causation (e.g., "is associated with").

We thank the Reviewer for their remarks on causal inference. We fully agree with the Reviewer that cause and effect relationships cannot be derived from our analyses of observational data but that, inter alia, experimental designs are needed for drawing conclusions on causal physical activity and affective well-being relationships. We have emphasised this in the discussion of the revised manuscript (see our response to comment 2). Additionally, we have comprehensively revised all wordings that might have implied causality and have made it clear that all references to directionality refer solely to the temporal order of the affective well-being and physical activity assessments, e.g.: "temporally bidirectional PA-AWB associations" and "associated with both prior and subsequent AWB" instead of "bidirectionally related", "variations" instead of "modulations".

5. While the paper is methodologically sound, the framing currently reads as if written primarily for behavioral medicine or exercise psychology specialists. The broader readership of the journal might not immediately see the transdisciplinary implications. The authors could use the introduction and conclusion to explicitly link how these microtemporal processes contribute to general models of human decision-making and public health strategies.

We thank the Reviewer for their valuable feedback. We highly appreciate their recognition of our methods and their suggestions for amending the paper. The Reviewer's comment has prompted us to reflect on how we can better transfer the transdisciplinary significance of our findings to a broad readership.

Towards this aim, we incorporated the general sentiments of rising "affectivism", describing that in the last decades, the focus of research has shifted from cognitive to affective processes[32] . In essence, there is a general consensus that affective processes play a substantial role in shaping our experiences and behaviours, both on a macro-(societal-) and on a micro-(individual)-level and across various disciplines[32]. Affect has been found to influence key cognitive processes, such as attention, learning, and decision-making[97–99].

As suggested by the Reviewer, we revised the introduction and conclusion accordingly to make the transdisciplinary implications of our findings more explicit. We highlighted how the microtemporal associations of physical activity and affective well-being may contribute to general models of human decision-making and public health strategies, ensuring that our paper is accessible and relevant to a broader readership. Further, we have adapted and simplified paragraphs that might not have been understandable to the general reader.

Introduction (p. 4): In general, AWB is increasingly recognised as a key driver of human behaviour and experience; for example, AWB is not only shown to be involved in PA engagement but also in attention, learning, memory, and decision-making processes[29].

Introduction (p. 4): These findings, however, are constrained by low ecological validity due to artificial settings, recall biases, and a focus on differences between individuals[30–32]. Critically, between-person differences cannot be employed as a proxy for intra-individual processes[33], a well-known limitation referred to as the ecological fallacy[34–39]. Yet, to promote PA in daily life, it is of crucial importance to understand the within-person associations of PA and AWB in everyday life. In general, variations in AWB in everyday life plausibly affect how humans act and think and are currently suggested to drive key health behaviours such as PA and diet[8]. Insights into these daily life processes can personalise and tailor prevention and treatment by identifying affective facilitators and barriers therewith contributing to public health strategies[8,13].

Introduction (p. 4): […] that is, structured exercise sessions in laboratory/interventional studies versus incidental PA, comprising exercises but also low-threshold non-exercise activity as PA facets, in daily life studies.

Discussion (p. 10): The evidence on pronounced within-person PA-AWB relations substantiates the general sentiment of cutting-edge health behaviour theory development. Traditional health behaviour theories emphasising the rational and cognitive determination of health behaviour[68-72] show limited explanatory power[9,24,27,73]. To tackle the vital issue of health behaviour decision making and public health, novel models of health behaviour build on hedonic theories of behaviour[24-27,74-79] and take into account that affective, impulsive processes shape health behaviours including PA engagement. These affective, impulsive processes contrast against rational, reflective processes and regained attention subsequent to Kahnemann's "thinking slow, thinking fast" proposal[80]. Put simply, novel health behaviour models emphasise that it is not only the knowledge on positive health effects that drives PA engagement but actual experiences and affective feelings related to PA.

Discussion (p. 11): Ultimately, the micro-temporal processes promise to allow for individually- and context-tailored real-time support to prevent and intervene on a broad range of non-communicable diseases and thereby contribute to public health. Since variations in AWB are not only thought to be linked to PA engagement but also to human experiences and behaviours in general, it is tempting to speculate that PA may indirectly also influence other aspects of daily life, such as attention, memory, decision making, or learning, via fluctuations in AWB. General models of decision making, for example, postulate that incidental factors, such as lingering moods and emotions, can impact decisions[82,83]. Hence, PA-related changes in AWB unrelated to the object of decisions may nevertheless subtly affect how individuals make decisions in their daily lives[82].

Conclusion (p. 13): It supports the general sentiment of "affectivism"[29] and of motivational theories that highlight the relevance of affective processes. The findings argue for a dominant role of momentary

AWB fluctuations within humans to shape behaviour and experiences. The heterogeneity of PA-AWB associations underlines the importance of considering contextual factors, especially in vulnerable groups. Considering aspects of the social and physical environment of individuals, such as walkability or urban green spaces, appears of utmost importance for human decision making and public health strategies. The collected data provide an evidence base for precision medicine interventions, that may ultimately alleviate the inactivity pandemic and enhance human health around the globe.

6. Some figures (e.g., orchard plots, spaghetti plots) are complex and may be challenging for non-specialists to parse quickly. The authors could add simplified visual summaries with plain-language captions alongside the more technical plots. For key results, the authors may want to also consider including a single integrated conceptual diagram showing the bidirectional associations, their relative strength for each AWB dimension, and identified moderators.

We are grateful for the Reviewer's highly valuable feedback. We followed the Reviewer's suggestion and included a simplified visual summary of our findings in the revised manuscript. This summary includes the main findings on the bidirectional associations of physical activity and affective well-being, the relative strength of effects, and identified moderators.

Results (p. 6): For a graphical overview of findings, see Figure 2.

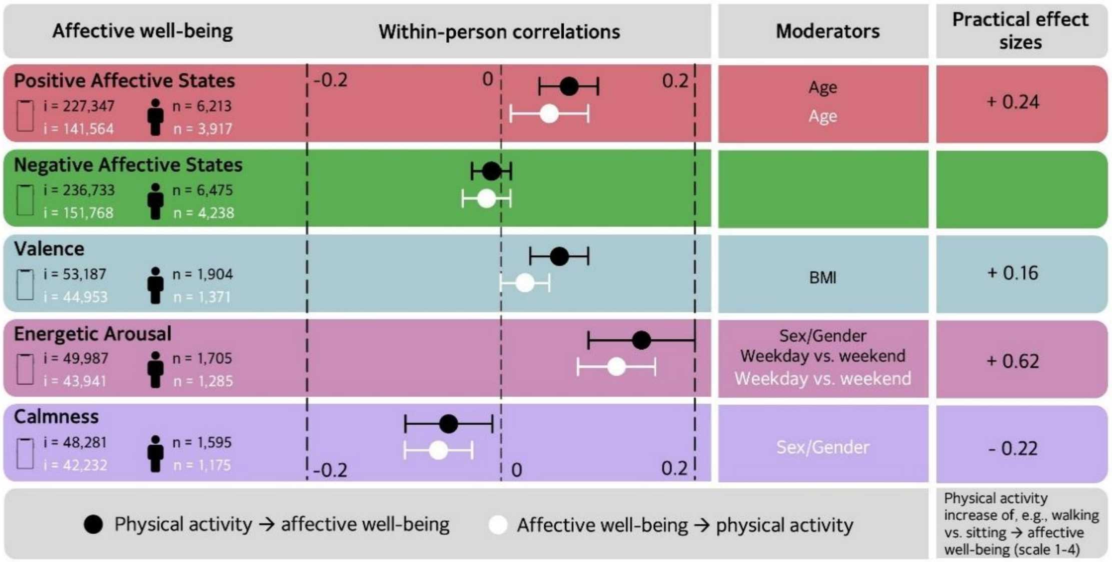

Figure 2. Graphical overview of findings. Summary of findings from all three analysis steps. First and in the left panel, within-person correlations of affective well-being (AWB) with prior (black; antecedent model) and subsequent (white; consequent) physical activity (PA) derived from two-stage individual participant data (IPD) meta-analysis (see S2) are displayed. Second and in the mid panel, moderating influences on associations between AWB and PA derived from one-stage IPD (see S3, S12) models are illustrated. Finally and in the right panel, practical effect sizes are depicted for significant associations of AWB and PA which were derived from Movement Acceleration Intensity (MAI) one-stage IPD models (see S3, S6). Practical effect sizes are exemplified for a PA increase in the range of walking at 5.4km/h versus sitting.

We would like to thank the Reviewer again for the time and effort they invested in reviewing and improving our manuscript. The Reviewer's suggestions allowed us to clarify the implications of our work and to improve the accessibility for a wider audience.

References

1. Molenberghs, G. & Kenward, M. G. *Missing Data in Clinical Studies* (Wiley, 2007).
2. Dora, J. *et al.* The daily association between affect and alcohol use: A meta-analysis of individual participant data. *Psychological bulletin* **149,** 1–24; 10.1037/bul0000387 (2023).
3. Kuehn, K. S. *et al.* A meta-analysis on the affect regulation function of real-time self-injurious thoughts and behaviours. *Nature Human Behaviour 2024 8:6* **6,** 964–974; 10.1038/s41562-022-01340-8 (2022).
4. Bolger, N. & Laurenceau, J.-P. *Intensive Longitudinal Methods: An Introduction to Diary and Experience Sampling Research* (The Guilford Press, 2013).
5. Hox, J. & van de Schoot, R. The SAGE Handbook of Multilevel Modeling, edited by M. Scott, J. S. Simonoff & B. D. Marx (sage publications2013), pp. 387–402.
6. Hox, J. J., Moerbeek, M. & van de Schoot, R. *Multilevel Analysis: Techniques and Applications* (2017).
7. Hamaker, E. L. Why researchers should think" within-person": A paradigmatic rationale (The Guilford Press2012), pp. 43–61.
8. Robinson, W. S. Ecological Correlations and the Behavior of Individuals. *American Sociological Review* **15,** 351–357; 10.1093/ije/dyn357 (1950).
9. Zawadzki, M. J., Smyth, J. M., Sliwinski, M. J., Ruiz, J. M. & Gerin, W. Revisiting the lack of association between affect and physiology: Contrasting between-person and within-person analyses. *Health psychology* **36,** 811–818; 10.1037/HEA0000466 (2017).
10. Kamarck, T. W., Schwartz, J. E., Janicki, D. L., Shiffman, S. & Raynor, D. A. Correspondence between laboratory and ambulatory measures of cardiovascular reactivity: A multilevel modeling approach. *Psychophysiology* **40,** 675–683; 10.1111/1469-8986.00069 (2003).
11. Reichert, M. *et al.* The association of stress and physical activity: Mind the ecological fallacy. *German Journal of Exercise and Sport Research* **52,** 282–289; 10.1007/S12662-022-00823-0/TABLES/1 (2022).
12. Wright, C. E., Rhodes, R. E., Ruggiero, E. W. & Sheeran, P. Benchmarking the effectiveness of interventions to promote physical activity: A metasynthesis. *Health psychology* **40,** 811–821; 10.1037/hea0001118 (2021).
13. Benedyk, A. *et al.* Real-life behavioral and neural circuit markers of physical activity as a compensatory mechanism for social isolation. *Nature Mental Health 2024 2:3* **2,** 337–342; 10.1038/s44220-024-00204-6 (2024).

14. Reichert, M. *et al.* A neural mechanism for affective well-being: Subgenual cingulate cortex mediates real-life effects of nonexercise activity on energy. *Science Advances* **6,** eaaz8934; 10.1126/SCIADV.AAZ8934 (2020).

15. Killingsworth, M. A. & Gilbert, D. T. A wandering mind is an unhappy mind. *Science* **330,** 932; 10.1126/SCIENCE.1192439 (2010).

16. Reichert, M. *et al.* Ambulatory assessment for physical activity research: State of the science, best practices and future directions. *Psychology of Sport and Exercise* **50,** 101742; 10.1016/j.psychsport.2020.101742 (2020).

17. Tost, H. *et al.* Neural correlates of individual differences in affective benefit of real-life urban green space exposure. *Nature neuroscience* **22,** 1389–1393; 10.1038/s41593-019-0451-y (2019).

18. Timm, I., Giurgiu, M., Ebner-Priemer, U. W. & Reichert, M. The within-subject association of physical behavior and affective well-being in everyday life: a systematic literature review. *Sports Medicine,* 1667–1705; 10.1007/s40279-024-02016-1 (2024).

19. Kanning, M. K., Ebner-Priemer, U. W. & Schlicht, W. M. How to Investigate Within-Subject Associations between Physical Activity and Momentary Affective States in Everyday Life: A Position Statement Based on a Literature Overview. *Frontiers in Psychology* **4,** 187; 10.3389/fpsyg.2013.00187 (2013).

20. Bourke, M., Hilland, T. A. & Craike, M. Contextual influences on the within-person association between physical activity and affect in adolescents: an ecological momentary assessment study. *Journal of behavioral medicine* **44,** 296–309; 10.1007/s10865-020-00197-4 (2021).

21. Bourke, M., Hilland, T. A. & Craike, M. Daily Physical Activity and Satisfaction with Life in Adolescents: An Ecological Momentary Assessment Study Exploring Direct Associations and the Mediating Role of Core Affect. *Journal of Happiness Studies* **23,** 949–968; 10.1007/S10902-021-00431-Z (2022).

22. Bourke, M., Hilland, T. A. & Craike, M. Domain specific association between physical activity and affect in adolescents' daily lives: an ecological momentary assessment study. *Psychology & health* **38,** 369–388; 10.1080/08870446.2021.1965603 (2023).

23. Timm, I., Reichert, M., Ebner-Priemer, U. W. & Giurgiu, M. Momentary within-subject associations of affective states and physical behavior are moderated by weather conditions in real life: an ambulatory assessment study. *International Journal of Behavioral Nutrition and Physical Activity* **20**; 10.1186/S12966-023-01507-0 (2023).

24. Liao, Y., Chou, C.-P., Huh, J., Leventhal, A. & Dunton, G. Associations of Affective Responses During Free-Living Physical Activity and Future Physical Activity Levels: an Ecological Momentary Assessment Study. *International journal of behavioral medicine* **24,** 513–519; 10.1007/s12529-016-9626-z (2017).

25. Maher, J. P. *et al.* Momentary assessment of physical activity intention-behavior coupling in adults. *Translational behavioral medicine* **7,** 709–718; 10.1007/S13142-017-0472-6 (2017).

26. Hevel, D. J., Dunton, G. F. & Maher, J. P. Acute Bidirectional Relations Between Affect, Physical Feeling States, and Activity-Related Behaviors Among Older Adults: An Ecological Momentary Assessment Study. *Annals of behavioral medicine : a publication of the Society of Behavioral Medicine* **55,** 41–54; 10.1093/abm/kaaa027 (2021).

27. Seiferth, C. *et al.* Bi-directional associations of core affect and physical activity in adults with higher body weight: An ecological momentary assessment study. *Journal of Health Psychology* **29,** 1115–1128; 10.1177/13591053241228202 (2024).

28. Kim, J., Nakamura, T., Kikuchi, H., Sasaki, T. & Yamamoto, Y. Co-variation of depressive mood and locomotor dynamics evaluated by ecological momentary assessment in healthy humans. *PloS one* **8,** e74979; 10.1371/journal.pone.0074979 (2013).

29. Haucke, M., Heinz, A., Liu, S. & Heinzel, S. The Impact of COVID-19 Lockdown on Daily Activities, Cognitions, and Stress in a Lonely and Distressed Population: Temporal Dynamic Network Analysis. *Journal of Medical Internet Research* **24,** e32598; 10.2196/32598 (2022).

30. Ekkekakis, P. The measurement of affect, mood, and emotion: A guide for health-behavioral research. *The Measurement of Affect, Mood, and Emotion: A Guide for Health-Behavioral Research*; 10.1017/CBO9780511820724 (2013).

31. Buecker, S., Simacek, T., Ingwersen, B., Terwiel, S. & Simonsmeier, B. A. Physical activity and subjective well-being in healthy individuals: a meta-analytic review. *Health Psychology Review* **15,** 574–592; 10.1080/17437199.2020.1760728 (2021).

32. Dukes, D. *et al.* The rise of affectivism. *Nature Human Behaviour 2024 8:6* **5,** 816–820; 10.1038/s41562-021-01130-8 (2021).

33. Kam, J. W. Y. *et al.* On the relationship between unprompted thought and affective well-being: A systematic review and meta-analysis. *Psychological bulletin* **150,** 621–641; 10.1037/bul0000428 (2024).

34. Williams, M. D., Rhodes, R. E. & T, C. M. (eds.). *Affective determinants of health behavior* (Oxford University Press, 2018).

35. Russell, J. A. & Barrett, L. F. Core affect, prototypical emotional episodes, and other things called emotion: dissecting the elephant. *Journal of personality and social psychology* **76,** 805–819; 10.1037//0022-3514.76.5.805 (1999).

36. Scherer, K. R. What are emotions? And how can they be measured? *Social Science Information* **44,** 695–729; 10.1177/0539018405058216 (2005).

37. Russell, J. A. Core affect and the psychological construction of emotion. *Psychological Review* **110,** 145–172; 10.1037/0033-295X.110.1.145 (2003).

38. Thayer, R. E. *The biopsychology of mood and activation* (Oxford University Press., New York, 1989).

39. Watson, D. & Tellegen, A. Toward a consensual structure of mood. *Psychological bulletin* **98,** 219–235; 10.1037//0033-2909.98.2.219 (1985).

40. Morris, W. N. *Mood. The Frame of Mind* (Springer New York, New York, NY, 1989).

41. Russell, J. A. A circumplex model of affect. *Journal of personality and social psychology* **39,** 1161–1178; 10.1037/H0077714 (1980).

42. Matthews, G., Jones, D. M. & Chamberlain, A. G. Refining the measurement of mood: The UWIST Mood Adjective Checklist. *British J of Psychology* **81,** 17–42; 10.1111/j.2044-8295.1990.tb02343.x (1990).

43. Steyer, R., Schwenkmezger, P., Notz, P. & Eid, M. Testtheoretische Analysen des Mehrdimensionalen Befindlichkeitsfragebogen (MDBF). *Diagnostica* (1994).

44. Schimmack, U. & Grob, A. Dimensional models of core affect: a quantitative comparison by means of structural equation modeling. *Eur. J. Pers.* **14,** 325–345; 10.1002/1099-0984(200007/08)14:4<325::AID-PER380>3.0.CO;2-I (2000).

45. Wilhelm, P. & Schoebi, D. Assessing mood in daily life: Structural validity, sensitivity to change, and reliability of a short-scale to measure three basic dimensions of mood. *European Journal of Psychological Assessment* **23,** 258–267; 10.1027/1015-5759.23.4.258 (2007).

46. Barrett, L. F. The theory of constructed emotion: an active inference account of interoception and categorization. *Social cognitive and affective neuroscience* **12,** 1833; 10.1093/scan/nsx060 (2017).

47. Ekman, P. Basic Emotions. In *Handbook of Cognition and Emotion,* edited by T. Dalgleish & M. J. Power (Wiley1999), pp. 45–60.

48. Lange, J. & Zickfeld, J. H. Emotions as Overlapping Causal Networks of Emotion Components: Implications and Methodological Approaches. *Emotion Review* **13,** 157–167; 10.1177/1754073920988787 (2021).

49. Scherer. Klaus R, Schorr, A. & Johnstone, T. (eds.). *Appraisal Processes in Emotion: Theory, Methods, Research* (Oup Usa, 2001).

50. Moors, A., Boddez, Y. & Houwer, J. de. The Power of Goal-Directed Processes in the Causation of Emotional and Other Actions. *Emotion Review* **9,** 310–318; 10.1177/1754073916669595 (2017).

51. Barrett, L. F. & Russell, J. A. (eds.). *The psychological construction of emotion* (Guilford Press, New York, London, 2015).

52. Mesquita, B. & Parkinson, B. Social Constructionist Theories of Emotions. In *Emotion Theory: The Routledge Comprehensive Guide,* edited by A. Scarantino (Routledge, New York, 2024), pp. 388–407.

53. Scarantino, A. (ed.). *Emotion Theory: The Routledge Comprehensive Guide* (Routledge, New York, 2024).

54. Russell, J. A. Emotion, core affect, and psychological construction. *Cognition and Emotion* **23,** 1259–1283; 10.1080/02699930902809375 (2009).

55. Ekkekakis, P. & Zenko, Z. Measurement of Affective Responses to Exercise. In *Emotion Measurement* (Elsevier2016), pp. 299–321.

56. Kim, J., Conroy, D. E. & Smyth, J. M. Bidirectional Associations of Momentary Affect with Physical Activity and Sedentary Behaviors in Working Adults. *Annals of behavioral medicine : a publication of the Society of Behavioral Medicine* **54,** 268–279; 10.1093/abm/kaz045 (2020).

57. Schwerdtfeger, A. R. & Gerteis, A. K. S. The manifold effects of positive affect on heart rate variability in everyday life: distinguishing within-person and between-person associations. *Health psychology* **33,** 1065–1073; 10.1037/hea0000079 (2014).

58. Cloos, L., Ceulemans, E. & Kuppens, P. Development, validation, and comparison of self-report measures for positive and negative affect in intensive longitudinal research. *Psychological assessment* **35,** 189–204; 10.1037/pas0001200 (2023).

59. Larsen, R. J., Diener, E. Promises and problems with the circumplex model of emotion. *Emotion: Review of Personality and Social Psychology (Band 13),* 25–59 (1992).

60. Williams, D. M. Psychological hedonism, hedonic motivation, and health behavior. In *Affective determinants of health behavior,* edited by M. D. Williams, R. E. Rhodes & C. M. T (Oxford University Press2018), pp. 204–234.

61. O'Donoghue, G. *et al.* A systematic review of correlates of sedentary behaviour in adults aged 18-65 years: a socio-ecological approach. *BMC Public Health* **16,** 163; 10.1186/s12889-016-2841-3 (2016).

62. Strain, T. *et al.* National, regional, and global trends in insufficient physical activity among adults from 2000 to 2022: a pooled analysis of 507 population-based surveys with 5·7 million participants. *The Lancet Global health* **12,** e1232-e1243; 10.1016/S2214-109X(24)00150-5 (2024).

63. Davis, M. G. *et al.* Objectively measured physical activity in a diverse sample of older urban UK adults. *Medicine and Science in Sports and Exercise* **43,** 647–654; 10.1249/MSS.0b013e3181f36196 (2011).

64. Sun, F., Norman, I. J. & While, A. E. Physical activity in older people: a systematic review. *BMC Public Health* **13,** 449; 10.1186/1471-2458-13-449 (2013).

65. Rhodes, R. E. & Kates, A. Can the Affective Response to Exercise Predict Future Motives and Physical Activity Behavior? A Systematic Review of Published Evidence. *Annals of behavioral medicine : a publication of the Society of Behavioral Medicine* **49,** 715–731; 10.1007/s12160-015-9704-5 (2015).

66. Bryan, A., Hutchison, K. E., Seals, D. R. & Allen, D. L. A transdisciplinary model integrating genetic, physiological, and psychological correlates of voluntary exercise. *Health psychology : official journal of the Division of Health Psychology, American Psychological Association* **26,** 30–39; 10.1037/0278-6133.26.1.30 (2007).

67. Hofmann, W. *et al.* Rethinking behaviour change interventions in policymaking. *Nature Human Behaviour 2024 8:6* **9,** 1765–1767; 10.1038/s41562-025-02284-5 (2025).

68. Braveman, P. & Gottlieb, L. The social determinants of health: it's time to consider the causes of the causes. *Public health reports (Washington, D.C. : 1974)* **129 Suppl 2,** 19–31; 10.1177/00333549141291S206 (2014).

69. Braveman, P., Egerter, S. & Williams, D. R. The social determinants of health: coming of age. *Annual review of public health* **32,** 381–398; 10.1146/annurev-publhealth-031210-101218 (2011).

70. Marmot, M., Allen, J., Bell, R., Bloomer, E. & Goldblatt, P. WHO European review of social determinants of health and the health divide. *Lancet (London, England)* **380,** 1011–1029; 10.1016/S0140-6736(12)61228-8 (2012).

71. Marmot, M. & Wilkinson, R. *Social Determinants of Health* (Oxford University PressOxford, 2005).

72. Donkin, A., Goldblatt, P., Allen, J., Nathanson, V. & Marmot, M. Global action on the social determinants of health. *BMJ global health* **3,** e000603; 10.1136/bmjgh-2017-000603 (2018).

73. Althoff, T. *et al.* Countrywide natural experiment links built environment to physical activity. *Nature* **645,** 407–413; 10.1038/s41586-025-09321-3 (2025).

74. Althoff, T. *et al.* Large-scale physical activity data reveal worldwide activity inequality. *Nature* **547,** 336–339; 10.1038/nature23018 (2017).

75. Berman, M. G. *et al.* Interacting with nature improves cognition and affect for individuals with depression. *Journal of affective disorders* **140,** 300–305; 10.1016/j.jad.2012.03.012 (2012).

76. Mnich, C., Weyland, S., Jekauc, D. & Schipperijn, J. Psychosocial and Physiological Health Outcomes of Green Exercise in Children and Adolescents-A Systematic

Review. *International journal of environmental research and public health* **16**; 10.3390/ijerph16214266 (2019).

77. Lahart, I., Darcy, P., Gidlow, C. & Calogiuri, G. The Effects of Green Exercise on Physical and Mental Wellbeing: A Systematic Review. *International journal of environmental research and public health* **16**; 10.3390/ijerph16081352 (2019).
78. Nigg, C. *et al.* Time to go green? *Sports Psychiatry* **1,** 116–118; 10.1024/2674-0052/a000018 (2022).
79. Lu, Y. *et al.* Wearable data link urban green space to physical activity. *Nat. Health*; 10.1038/s44360-025-00011-y (2025).
80. Elsangedy, H. M. *et al.* Poorer positive affect in response to self-paced exercise among the obese. *Physiology & behavior* **189,** 32–39; 10.1016/J.PHYSBEH.2018.02.031 (2018).
81. Ekkekakis, P. & Lind, E. Exercise does not feel the same when you are overweight: the impact of self-selected and imposed intensity on affect and exertion. *International journal of obesity* **30,** 652–660; 10.1038/SJ.IJO.0803052 (2006).
82. Ekkekakis, P., Lind, E. & Vazou, S. Affective responses to increasing levels of exercise intensity in normal-weight, overweight, and obese middle-aged women. *Obesity* **18,** 79–85; 10.1038/OBY.2009.204 (2010).
83. Jackson, T., Gao, X. & Chen, H. Differences in neural activation to depictions of physical exercise and sedentary activity: an fMRI study of overweight and lean Chinese women. *International journal of obesity (2005)* **38,** 1180–1185; 10.1038/IJO.2013.245 (2014).
84. Ingram, D. K. Age-related decline in physical activity: generalization to nonhumans. *Medicine & Science in Sports & Exercise* **32,** 1623–1629; 10.1097/00005768-200009000-00016 (2000).
85. Williams, D. M. *et al.* Recommending self-paced exercise among overweight and obese adults: a randomized pilot study. *Annals of behavioral medicine : a publication of the Society of Behavioral Medicine* **49,** 280–285; 10.1007/s12160-014-9642-7 (2015).
86. Da Silva, C. E. R. *et al.* Self-selected vs. prescribed aerobic exercise intensity: impacts on pleasure in women with obesity. *Frontiers in Psychology* **16,** 1533785; 10.3389/fpsyg.2025.1533785 (2025).
87. Dart, A. M., Du Jun, X. & Kingwell, B. A. Gender, sex hormones and autonomic nervous control of the cardiovascular system. *Cardiovascular research* **53,** 678–687; 10.1016/S0008-6363(01)00508-9 (2002).

88. Koenig, J. & Thayer, J. F. Sex differences in healthy human heart rate variability: A meta-analysis. *Neuroscience & Biobehavioral Reviews* **64,** 288–310; 10.1016/J.NEUBIOREV.2016.03.007 (2016).

89. Tobaldini, E. *et al.* Depression and cardiovascular autonomic control: a matter of vagus and sex paradox. *Neuroscience & Biobehavioral Reviews* **116,** 154–161; 10.1016/J.NEUBIOREV.2020.06.029 (2020).

90. Thayer, J. F., Ahs, F., Fredrikson, M., Sollers, J. J. & Wager, T. D. A meta-analysis of heart rate variability and neuroimaging studies: implications for heart rate variability as a marker of stress and health. *Neuroscience and biobehavioral reviews* **36,** 747–756; 10.1016/j.neubiorev.2011.11.009 (2012).

91. Kim, H.-G., Cheon, E.-J., Bai, D.-S., Lee, Y. H. & Koo, B.-H. Stress and Heart Rate Variability: A Meta-Analysis and Review of the Literature. *Psychiatry investigation* **15,** 235–245; 10.30773/pi.2017.08.17 (2018).

92. White, R. L. *et al.* Domain-Specific Physical Activity and Mental Health: A Meta-analysis. *American Journal of Preventive Medicine* **52,** 653–666; 10.1016/J.AMEPRE.2016.12.008 (2017).

93. Cho, D., Armeli, S., Weinstock, J. & Tennen, H. Daily- and Person-Level Associations Between Physical Activity and Alcohol Use Among College Students. *Emerging Adulthood* **8,** 428–434; 10.1177/2167696818809760 (2018).

94. Holtermann, A., Schnohr, P., Nordestgaard, B. G. & Marott, J. L. The physical activity paradox in cardiovascular disease and all-cause mortality: the contemporary Copenhagen General Population Study with 104 046 adults. *European heart journal* **42,** 1499–1511; 10.1093/EURHEARTJ/EHAB087 (2021).

95. Clays, E. *et al.* Occupational and leisure time physical activity in contrasting relation to ambulatory blood pressure. *BMC Public Health* **12,** 1002; 10.1186/1471-2458-12-1002 (2012).

96. Prince, S. A. *et al.* The effect of leisure time physical activity and sedentary behaviour on the health of workers with different occupational physical activity demands: a systematic review. *International Journal of Behavioral Nutrition and Physical Activity* **18,** 100; 10.1186/s12966-021-01166-z (2021).

97. Pertl, S. M., Srirangarajan, T. & Urminsky, O. A multinational analysis of how emotions relate to economic decisions regarding time or risk. *Nature Human Behaviour 2024 8:6* **8,** 2139–2155; 10.1038/s41562-024-01927-3 (2024).

98. Blanchette, I. & Richards, A. The influence of affect on higher level cognition: A review of research on interpretation, judgement, decision making and reasoning. *Cognition and Emotion* **24,** 561–595; 10.1080/02699930903132496 (2010).

99. Loewenstein, G. & Lerner, J. S. The Role of Affect in Decision Making. In *Handbook of Affective Sciences,* edited by R. J. Davidson, K. R. Scherer & H. H. Goldsmith (Oxford University PressNew York, NY2002), pp. 619–642.
